# Shortest Enantioselective Total Syntheses of (+)-Isolaurepinnacin and (+)-Neoisoprelaurefucin

**DOI:** 10.1021/acs.orglett.2c01769

**Published:** 2022-07-14

**Authors:** Victoria Sinka, Daniel A. Cruz, Víctor S. Martín, Juan I. Padrón

**Affiliations:** † Instituto de Productos Naturales y Agrobiología (IPNA), CSIC, 38206 Avda. Astrofísico Fco. Sánchez 3, 38206 La Laguna, Tenerife, Spain; ‡ Organic Chemistry Department Avda, Universidad de La Laguna, Instituto Universitario de Bio-Orgánica Antonio González (IUBO), Astrofísico Fco. Sánchez 2, 38206 La Laguna, Tenerife, Spain

## Abstract

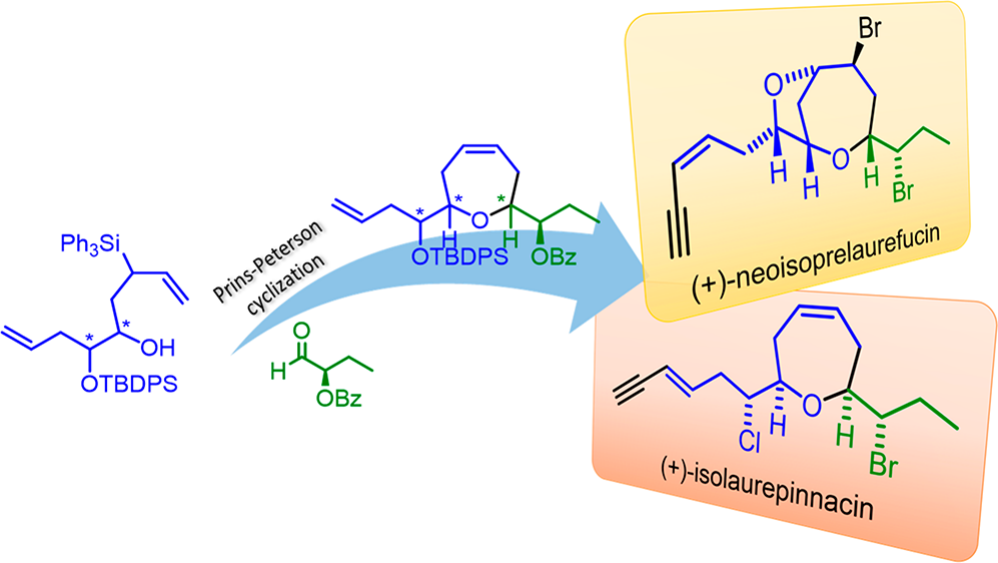

The shortest enantioselective total syntheses of (+)-isolaurepinnacin and (+)-neoisoprelaurefucin have been accomplished. These syntheses were based on a common parallel synthetic strategy using Prins–Peterson cyclization in their core construction. In only one step, a seven-membered ring oxacycle with the correct *cis*-stereochemistry ring closure and the Δ^4^ position of the endocyclic double bond in (+)-isolaurepinnacin was obtained. This unsaturation was also necessary to accede to the bromodioxabicycle on (+)-neoisoprelaurefucin.

(+)-Isolaurepinnacin and (+)-neoisoprelaurefucin are marine natural products, containing a seven-membered cyclic ether unit, isolated from red seaweeds of the genus *Laurencia*, the first from *Laurencia pinnata* Yamada^1^ and the second from *Laurencia nipponica* Yamada.^2^ These macroalgae produce a large number of unique metabolites, such us lauroxanes, a family of nonterpenoid C_15_-metabolites that exhibit a wide range of biological properties ranging from antitumor to antiepileptic activity (Figure 1).^3−6^

**Figure 1:**
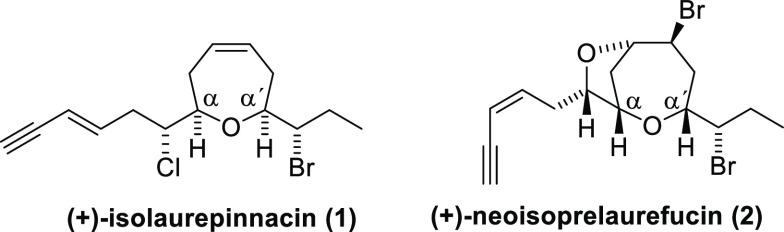
Structures of (+)-isolaurepinnacin (**1**) and (+)-neoisoprelaurefucin (**2**).

Among the members of the lauroxane family, (+)-isolaurepinnacin (**1**) and (+)-neoisoprelaurefucin (**2**) are representative examples of compounds containing an oxepane ring. Both of them share a central seven-membered oxacycle with α,α′-*cis*-disubstitution furnished with two lateral chains, including halogen atoms and a terminal enyne moiety.

The main difference lies in the configuration of the enyne *E* on (+)-isolaurepinnacin (**1**) and *Z* on (+)-neoisoprelaurefucin (**2**), in addition to the fused five-membered oxacycle leading to a bromodioxabicycle on (+)-neoisoprelaurefucin (**2**). The literature only shows one total synthesis performed by Overman et al. in 1993 and a formal approach by Suzuki et al. in 2001 for (+)-isolaurepinnacin (**1**),^7−9^ whereas (+)-neoisoprelaurefucin (**2**) has had only one total synthesis, accomplished by Kim et al. in 2003 (Figure 2).^10^

**Figure 2:**
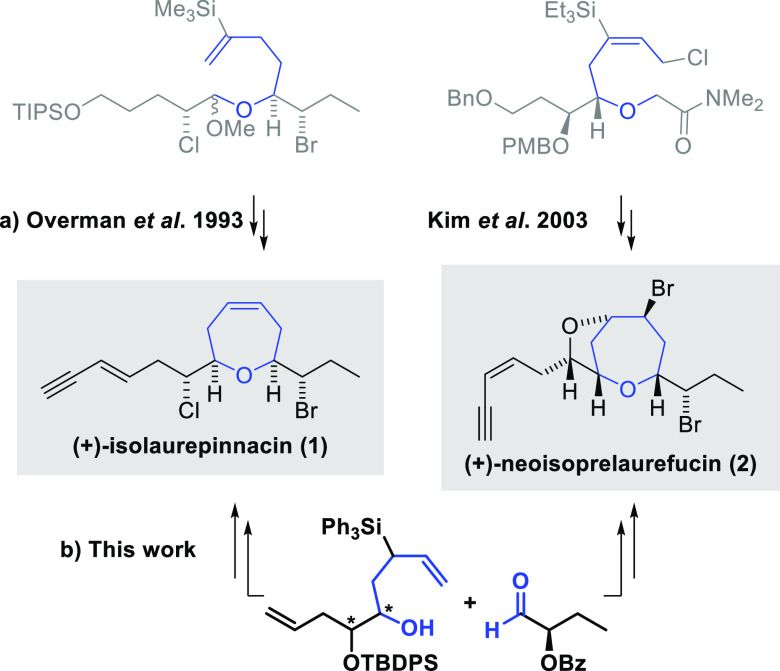
(+)-Isolaurepinnacin and (+)-neoisoprelaurefucin total syntheses. (a) Previous approaches. (b) Our proposal.

Our research group has developed synthetic methodologies that were successfully applied in the preparation of several medium size oxacycles.^11−14^ Moreover, we reported a straightforward method to gain access to *cis*-disubstituted Δ^4^-unsaturated oxepanes in one step that was supported by the excellent previous work of other authors.^15^ Thus, we resolved to apply our experience to the total synthesis of (+)-isolaurepinnacin (**1**) and (+)-neoisoprelaurefucin (**2**) using a common parallel synthetic strategy (Scheme 1).

**Scheme 1:**
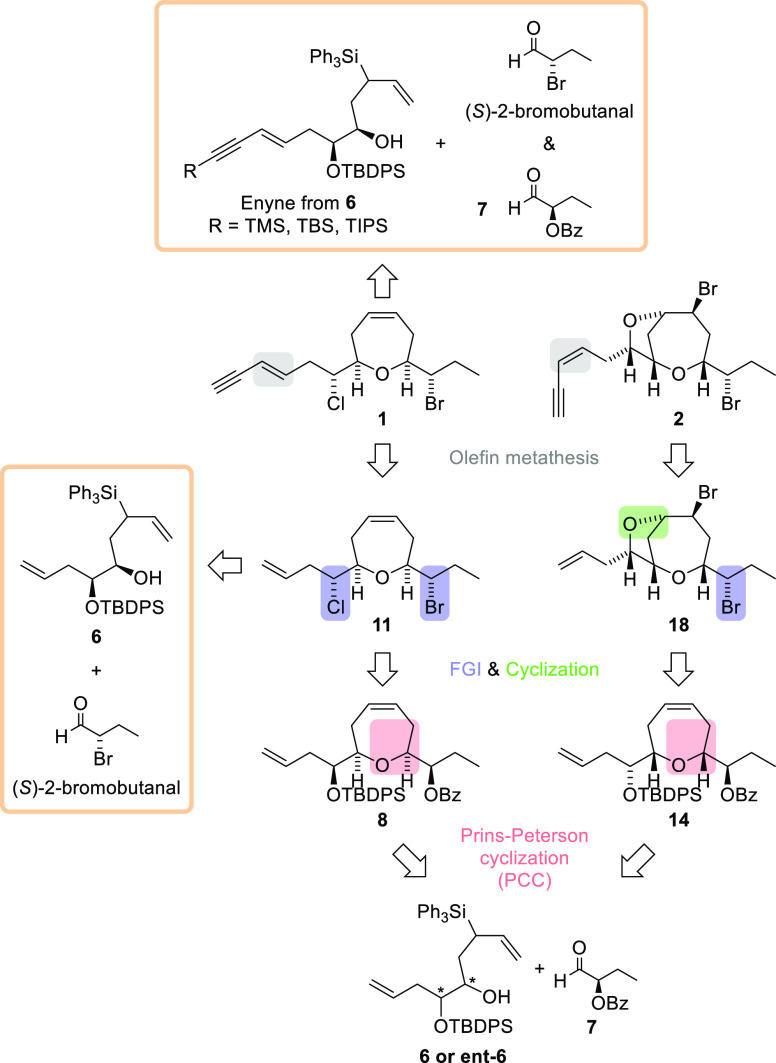
Retrosynthetic Analysis to Access (+)-Isolaurepinnacin (**1**) and (+)-Neoisoprelaurefucin (**2**) from the Bis-homoallylic Silyl Alcohol **6** or *ent*-**6**

In previous work, we demonstrated the ability of iron(III) salts to catalyze the Prins cyclization, which is now a prolific synthetic tool to obtain medium size oxacycles. Among the various types of oxacycles that can be accessed, synthesis of Δ^4^-2,7-disubstituted oxepenes in a single step is one of the most striking applications to date. The Prins–Peterson cyclization (PPC) is highly efficient and stereoselective and only requires a substoichiometric amount of FeBr_3_.^15^

Encouraged by these results, we decided to test the capabilities of PPC as the key and main step in the synthesis of two marine natural products: (+)-isolaurepinnacin (**1**) and (+)-neoisoprelaurefucin (**2**).

We envisioned the preparation of these natural products through this methodology as a great opportunity to achieve more efficient total syntheses by reducing the number of steps. A retrosynthetic analysis for both compounds was designed to use a parallel but common synthetic strategy. This maintained our goal of forging two routes with the highest level of similarity.

As depicted in Scheme 1, the first disconnection was the lateral *E* or *Z* enyne chain. Next, we focused on introducing halogen atoms in the case of (+)-isolaurepinnacin (**11** → **8**) and on inversion of the hydroxyl group and further bromo-etherification to arrive at (+)-neoisoprelaurefucin (**18** → **14**). Finally, the Prins–Peterson cyclization as the key reaction of our retrosynthetic proposal should lead to the functionalized oxepene rings. It has the correct stereochemistry and would start from the unsaturated silyl alcohols **6** and *ent*-**6**.^15^

Equally, more direct PPC disconnections were attempted for the total synthesis of (+)-isolaurepinnacin. The first was the construction of the Prins cyclization precursor with the enyne chain, leaving the generation of the oxepene ring for the last steps of the synthesis. In this case, none of the cyclization conditions tested with aldehyde **7** or (*S*)-2-bromobutanal gave rise to the desired oxepene (Scheme 1). The second focused on obtaining the intermediate oxepene **11** from the cyclization of the alcohol precursor **6** and (*S*)-2-bromobutanal. However, cyclization with this aldehyde proved unsuccessful. In general, the aldehyde with a α-bromo-((*S*)-2-bromobutanal) was unreactive in all the PPC reactions tested (Scheme 1).

The synthesis of (+)-isolaurepinnacin started with the enantioselective epoxidation of the 1,5-hexadien-3-ol (**3**) developed by Sharpless et al. (Scheme 2).^16^ Then we protected the resulting epoxy alcohol with TBDPSCl to obtain the desired epoxide **4** in an excellent yield. Next, the epoxide was opened by nucleophilic attack using the silane **5**, following Corriu et al., which afforded us the unsaturated silyl alcohol **6** in 57% yield.^17,18^ We decided to use this method because of the better ratio between the (desired) α attack versus γ attack in opening the epoxide. In comparison, the analogous strategy developed by Schaumann et al. provided a worse outcome in this reaction (see Supporting Information).^19,20^

**Scheme 2:**
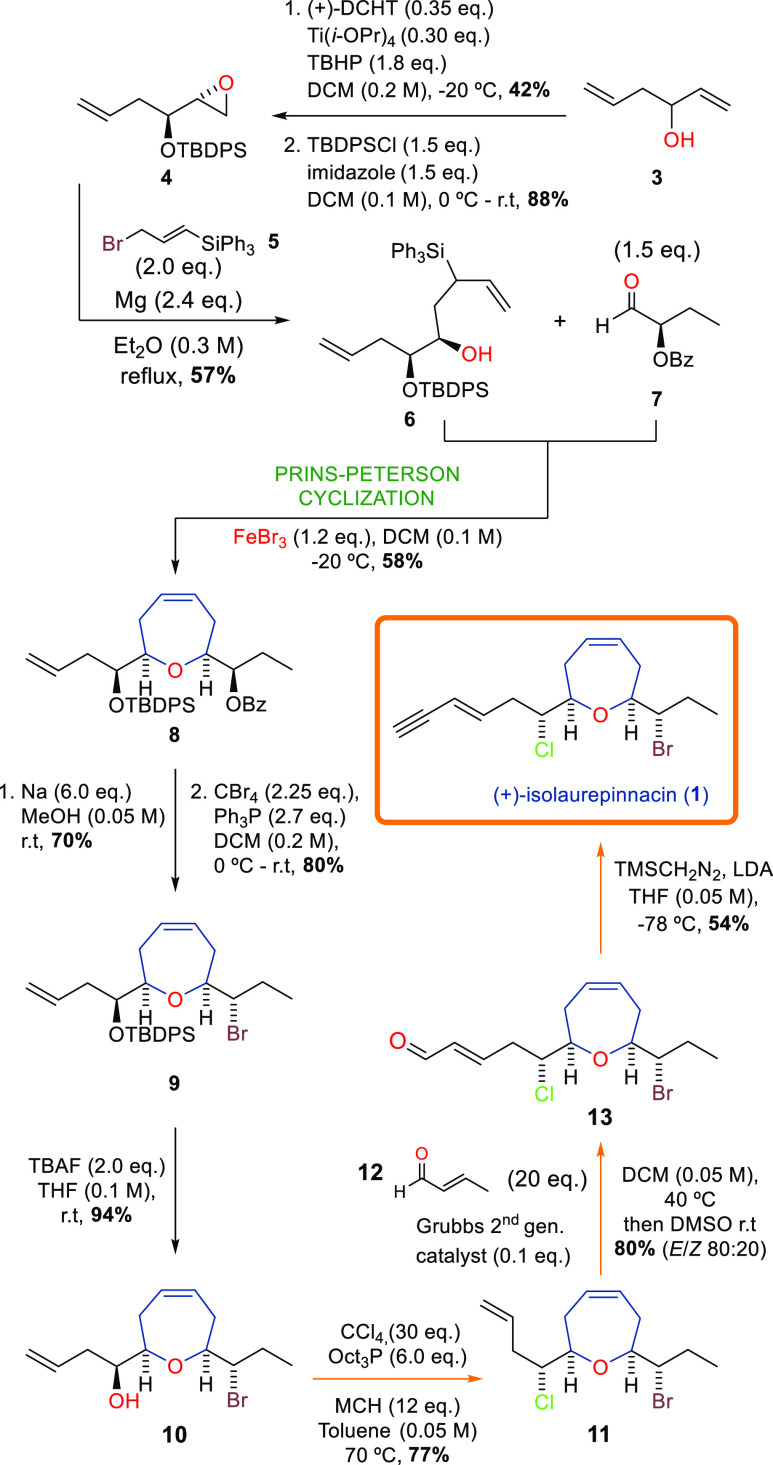
Total Synthesis for (+)-Isolaurepinnacin (**1**)

At this point in the synthesis, we introduced the PPC to achieve the pivotal reaction of the route. Here, a stoichiometric amount of FeBr_3_ and low temperature were essential to enable cyclization with the freshly prepared aldehyde **7** (see Supporting Information). In this case, we obtained the desired Δ^4^-2,7-disubstituted oxepene ring **8** with a 58% yield. A bulky substituent, the TBDPS group, is present at the α-position relative to the hydroxyl group of unsaturated silyl alcohols. This bulky substituent drives the PPC toward the exclusive formation of the *cis*-oxepene.^15^

Once oxepene **8** was ready, the next step was substitution of the benzoate group by a bromine atom, with suitable stereochemistry of the final natural product **1**. We completed this modification in good yield after basic hydrolysis of the ester and the subsequent Appel reaction to obtain **9**.^21^ For this basic hydrolysis of the benzoate group, it was necessary to perform several trials with different bases. The best conditions were with Na/MeOH at room temperature (see Supporting Information). Cleavage of the silyl protecting group of oxepene **9** was facilitated using TBAF, with a yield of 94%.

When the alcohol **10** was prepared, we again took advantage of the Appel reaction by replacing the hydroxyl group with a chlorine atom in good yield (**11**). Next, an olefin metathesis reaction catalyzed by the second-generation Grubbs catalyst led us to the conjugated aldehyde **13** in 80% yield, with an *E*/*Z* ratio of 80:20, which was favorable for the desired compound *E*.^22,23^ Finally, the Colvin rearrangement afforded (+)-isolaurepinnacin (**1**) in 54% yield,^24−26^ completing the total synthesis in 10 steps with an overall yield of 5%.

Next, the synthesis of (+)-neoisoprelaurefucin was designed to use the maximum number of common steps of (+)-isolaurepinnacin (Scheme 3). Therefore, alcohol **3** was epoxidized with the protocol by Sharpless et al. but using (−)-DCHT, followed by protection with TBDPSCl to obtain the epoxide *ent*-**4**. The opening of *ent*-**4** with silane **5** provided the silyl alcohol *ent*-**6** in 56% yield, and the subsequent PPC reaction led us to the oxepene **14** in 52% yield. Now, deprotection of the benzoate group and Appel reaction were conducted to install the bromine atom with suitable stereochemistry in oxepene **15**. The conditions previously used for the cleavage of the TBDPS group were unsuccessful for oxepene **15**, as it led to an undesired elimination reaction. Therefore, we switched to a milder method to avoid the generation of TBAF byproducts using the AcCl/MeOH system to obtain alcohol **16** in 80% yield.^27^ From this step onward, this route went its own way due to structural differences between the natural products (Scheme 3).

**Scheme 3:**
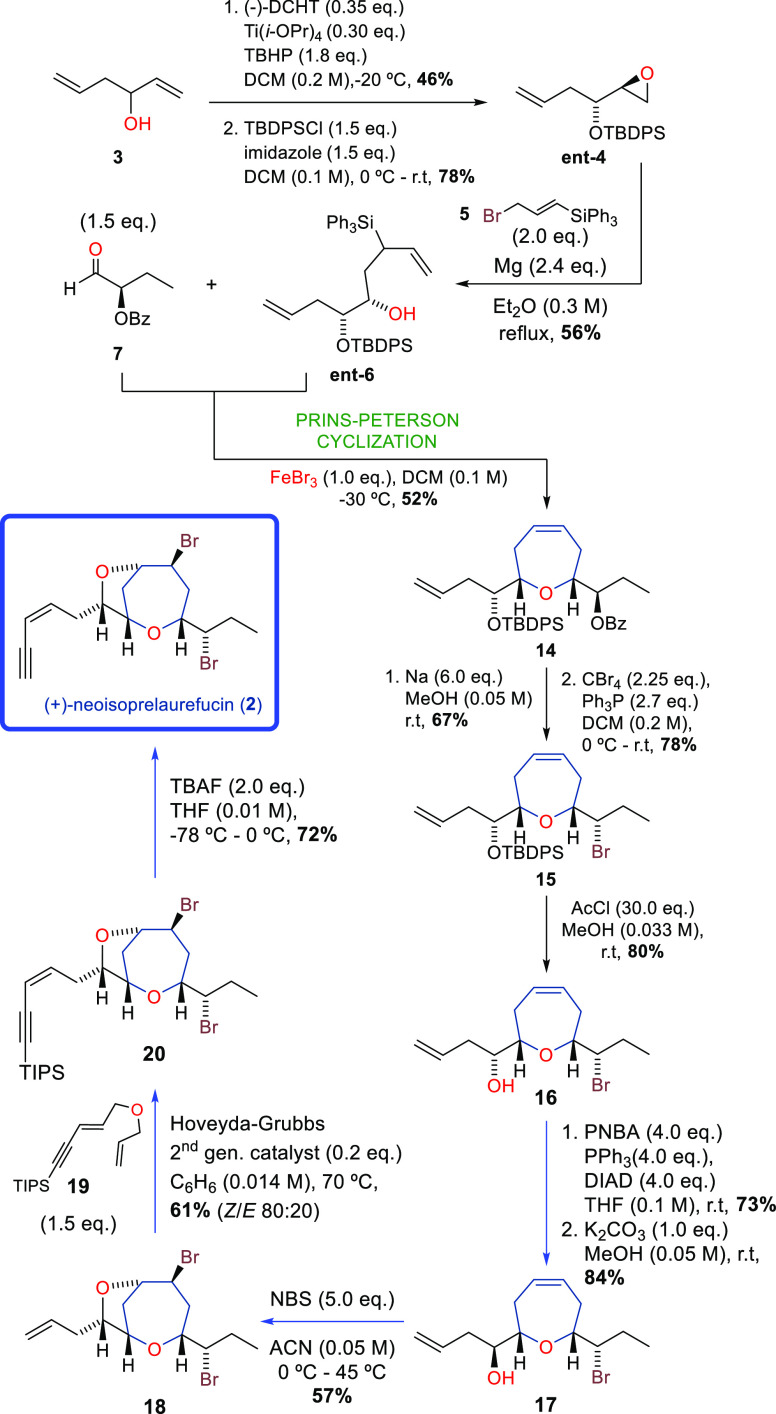
Total Synthesis for (+)-Neoisoprelaurefucin (**2**)

The first specific step in the synthesis of (+)-neoisoprelaurefucin (**2**) was inversion of the hydroxyl group in oxepene **16**. In this case, the Mitsunobu reaction and subsequent hydrolysis of the resulting *p*-NO_2_ benzoate provided the new alcohol **17** in 61% yield (two steps).^28^

At this point, we also considered the possibility of a more direct and convergent approach to access oxepene **17** via a Mitsunobu reaction on the hydroxyl group of precursor **6**. This approach would allow us to save one reaction step and shorten the total synthesis; however, the inversion of the configuration in the secondary alcohol did not work. This attempt failed probably due to the high steric hindrance caused by the presence of the TPS and OTBDPS groups (see Supporting Information).

Next, we used *N*-bromosuccinimide to promote stereospecific cyclization from the hydroxyl group over the bromonium ion to obtain the bromodioxabicycle **18** with a yield of 57%. At this stage, we introduced the alkyne group into the side chain. This required the method developed by Lee et al.,^29^ wherein the allyl enyne ether **19** is essential to afford the dioxabicycle **20** containing the *Z* isomer as the major product (*E*/*Z* = 20:80). Finally, the TIPS group was deprotected at a low temperature to avoid the formation of possible byproducts, providing the (+)-neoisoprelaurefucin (**2**) in 72% yield. Thus, this total synthesis was achieved in 12 steps and 1.3% overall yield (Scheme 3).

In conclusion, we have achieved enantioselective total syntheses for the two marine natural products (+)-isolaurepinnacin (**1**) and (+)-neoisoprelaurefucin (**2**). The design of the synthetic routes depends on a parallel approach to maximize the number of common steps, while taking the Prins–Peterson cyclization methodology as the pivotal synthetic step. This approach allows us to obtain the (+)-isolaurepinnacin in 10 reaction steps with an overall yield of 5% and the (+)-neoisoprelaurefucin in 12 steps with an overall yield of 1.3%. Thus, we report the shortest convergent total syntheses to date for both compounds.

## Supporting Information Available

Experimental procedures and characterization data for new compounds (PDF)

## References

[ref1] Fukuzawa A.; Masamune T. Laurepinnacin and Isolaurepinnacin, New Acetylenic Cyclic Ethers from the Marine Red Alga Laurencia Pinnata Yamada. Tetrahedron Lett. 1981, 22 (41), 4081–4084. 10.1016/S0040-4039(01)82070-0.

[ref2] Suzuki M.; Mizuno Y.; Matsuo Y.; Masuda M. Neoisoprelaurefucin, a Halogenated C15 Non-Terpenoid Compound from Laurencia Nipponica. Phytochemistry 1996, 43 (1), 121–124. 10.1016/0031-9422(96)00237-3.

[ref3] Faulkner D. J. Marine Natural Products. Nat. Prod. Rep. 2002, 19 (1), 1–48. 10.1039/b009029h. 11902436

[ref4] Blunt J. W.; Copp B. R.; Keyzers R. A.; Munro M. H. G.; Prinsep M. R. Marine Natural Products. Nat. Prod. Rep. 2013, 30 (2), 237–323. 10.1039/C2NP20112G. 23263727

[ref5] Wang B.-G.; Gloer J. B.; Ji N.-Y.; Zhao J.-C. Halogenated Organic Molecules of Rhodomelaceae Origin: Chemistry and Biology. Chem. Rev. 2013, 113 (5), 3632–3685. 10.1021/cr9002215. 23448097

[ref6] Dembitsky V. M.; Levitsky D. O.; Gloriozova T. A.; Poroikov V. V. Acetylenic Aquatic Anticancer Agents and Related Compounds. Nat. Prod. Commun. 2006, 10.1177/1934578X0600100914.

[ref7] Berger D.; Overman L. E.; Renhowe P. A. Enantioselective Total Synthesis of (+)-Isolaurepinnacin. J. Am. Chem. Soc. 1993, 115 (20), 9305–9306. 10.1021/ja00073a063.

[ref8] Berger D.; Overman L. E.; Renhowe P. A. Total Synthesis of (+)-Isolaurepinnacin. Use of Acetal-Alkene Cyclizations To Prepare Highly Functionalized Seven-Membered Cyclic Ethers. J. Am. Chem. Soc. 1997, 119 (10), 2446–2452. 10.1021/ja964080b.

[ref9] Suzuki T.; Matsumura R.; Oku K.; Taguchi K.; Hagiwara H.; Hoshi T.; Ando M. Formal Synthesis of (+)-Isolaurepinnacin. Tetrahedron Lett. 2001, 42 (1), 65–67. 10.1016/S0040-4039(00)01880-3.

[ref10] Lee H.; Kim H.; Baek S.; Kim S.; Kim D. Total Synthesis and Determination of the Absolute Configuration of (+)-Neoisoprelaurefucin. Tetrahedron Lett. 2003, 44 (35), 6609–6612. 10.1016/S0040-4039(03)01667-8.

[ref11] Miranda P. O.; Ramírez M. A.; Martín V. S.; Padrón J. I. The Silylalkyne-Prins Cyclization: Stereoselective Synthesis of Tetra- and Pentasubstituted Halodihydropyrans. Org. Lett. 2006, 8 (8), 1633–1636. 10.1021/ol060247m. 16597128

[ref12] Miranda P. O.; Carballo R. M.; Martín V. S.; Padrón J. I. A New Catalytic Prins Cyclization Leading to Oxa- and Azacycles. Org. Lett. 2009, 11 (2), 357–360. 10.1021/ol802593u. 19093859

[ref13] Purino M. A.; Ramírez M. A.; Daranas A. H.; Martín V. S.; Padrón J. I. Iron(III) Catalyzed Direct Synthesis of Cis-2,7-Disubstituted Oxepanes. The Shortest Total Synthesis of (+)-Isolaurepan. Org. Lett. 2012, 14 (23), 5904–5907. 10.1021/ol3028016. 23167915

[ref14] Scoccia J.; Pérez S. J.; Sinka V.; Cruz D. A.; López-Soria J. M.; Fernández I.; Martín V. S.; Miranda P. O.; Padrón J. I. Direct Access to 2,3,4,6-Tetrasubstituted Tetrahydro-2H-Pyrans via Tandem SN2′–Prins Cyclization. Org. Lett. 2017, 19 (18), 4834–4837. 10.1021/acs.orglett.7b02270. 28858515

[ref15] Cruz D. A.; Sinka V.; Martín V. S.; Padrón J. I. Iron-Catalyzed Prins–Peterson Reaction for the Direct Synthesis of Δ4–2,7-Disubstituted Oxepenes. J. Org. Chem. 2018, 83 (20), 12632–12647. and references cited therein 10.1021/acs.joc.8b01978. 30252471

[ref16] Gao Y.; Klunder J. M.; Hanson R. M.; Masamune H.; Ko S. Y.; Sharpless K. B. Catalytic Asymmetric Epoxidation and Kinetic Resolution: Modified Procedures Including in Situ Derivatization. J. Am. Chem. Soc. 1987, 109 (19), 5765–5780. 10.1021/ja00253a032.

[ref17] Corriu R.; Masse J. Organométalliques Allyliques Siliciés: Obtention et Comportement. J. Organomet. Chem. 1973, 57 (1), C5–C8. 10.1016/S0022-328X(00)89640-0.

[ref18] Corriu R. J. P.; Masse J.; Samate D. Syntheses a Partir de Carbanions Allyliques Silicies: I. Carbanions Derives de Monoallylsilanes. J. Organomet. Chem. 1975, 93 (1), 71–80. 10.1016/S0022-328X(00)94145-7.

[ref19] Schaumann E.; Kirschning A. Ring-Opening of Oxiranes by Silyl-Substituted Allyl Anions. A Regiochemical Chameleon. Tetrahedron Lett. 1988, 29 (34), 4281–4284. 10.1016/S0040-4039(00)80474-8.

[ref20] Schaumann E.; Kirschning A.; Narjes F. Synthesis of Vinylcyclopropanes from Epoxides. J. Org. Chem. 1991, 56 (2), 717–723. 10.1021/jo00002a043.

[ref21] Appel R. Tertiary Phosphane/Tetrachloromethane, a Versatile Reagent for Chlorination, Dehydration, and P-N Linkage. Angew. Chem., Int. Ed. Engl. 1975, 14 (12), 801–811. 10.1002/anie.197508011.

[ref22] Kim B.; Lee M.; Kim M. J.; Lee H.; Kim S.; Kim D.; Koh M.; Park S. B.; Shin K. J. Biomimetic Asymmetric Total Synthesis of (−)-Laurefucin via an Organoselenium-Mediated Intramolecular Hydroxyetherification. J. Am. Chem. Soc. 2008, 130 (49), 16807–16811. 10.1021/ja806304s. 19049472

[ref23] Kim B.; Sohn T.; Kim D.; Paton R. S. Asymmetric Total Syntheses and Structure Confirmation of Chlorofucins and Bromofucins.. Chem.–Eur. J. 2018, 24 (11), 2634–2642. 10.1002/chem.201704564. 29222867

[ref24] Colvin E. W.; Hamill B. J. One-Step Conversion of Carbonyl Compounds into Acetylenes. J. Chem. Soc. Chem. Commun. 1973, 5, 151–152. 10.1039/c39730000151.

[ref25] Ohira S.; Okai K.; Moritani T. Generation of Alkylidenecarbenes by the Alkenation of Carbonyl Compounds with Lithiotrimethylsilyldiazomethane. J. Chem. Soc. Chem. Commun. 1992, 9, 721–722. 10.1039/c39920000721.

[ref26] Miwa K.; Aoyama T.; Shioiri T. Extension of the Colvin Rearrangement Using Trimethylsilyldiazomethane. A New Synthesis of Alkynes. Synlett 1994, 1994 (02), 107–108. 10.1055/s-1994-22755.

[ref27] Padrón J. I.; Vázquez J. T. Stereochemical Study of the CD Spectral Differences between Anomers of Alkyl Glucopyranosides. Tetrahedron: Asymmetry 1998, 9 (4), 613–627. 10.1016/S0957-4166(98)00037-8.

[ref28] Mitsunobu O.; Yamada M. Preparation of Esters of Carboxylic and Phosphoric Acid via Quaternary Phosphonium Salts. Bull. Chem. Soc. Jpn. 1967, 40 (10), 2380–2382. 10.1246/bcsj.40.2380.

[ref29] Hansen E. C.; Lee D. Efficient and Z-Selective Cross-Metathesis of Conjugated Enynes. Org. Lett. 2004, 6 (12), 2035–2038. 10.1021/ol049378i. 15176812

