# Supplementary material for: Shortest Enantioselective Total Syntheses of (+)-Isolaurepinnacin and (+)-Neoisoprelaurefucin
Source: Org Lett. 2022 Jul 14;24(29):5271–5. doi: 10.1021/acs.orglett.2c01769 (PMC9344465; doi:10.1021/acs.orglett.2c01769)
Supplement: Supplementary file 1 — ol2c01769_si_001.pdf [file ol2c01769_si_001.pdf]

The Shortest Enantioselective Total Syntheses of (+)-Isolaurepinnacin and (+)-Neoisoprelaurefucin

Victoria Sinka,*[a] Daniel A. Cruz, [a]* Víctor S. Martín[b] and Juan I. Padrón*[a]

[a] Dr. V. Sinka, Dr. D. A. Cruz, Dr. J. I. Padrón. Instituto de Productos Naturales y Agrobiología (IPNA), CSIC, 38206. Avda. Astrofísico Fco. Sánchez 3, 38206 La Laguna, Tenerife (Spain). E-mail: jipadron@ipna.csic.es; vsinka@ipna.csic.es; danielcruz@ipna.csic.es

[b] Prof. V. S. Martín. Universidad de La Laguna, Instituto Universitario de Bio-Orgánica Antonio González (IUBO), Organic Chemistry Department Avda. Astrofísico Fco. Sánchez 2, 38206, La Laguna, Tenerife (Spain).

Table of Contents

1. General Experimental Information S3
1.1. Reagents and solvents S3
1.2. Chromatography S3
1.3. Nuclear magnetic resonance (NMR) S3
1.4. Mass spectrometry S3
1.5. Optical rotation S3
2. Supporting schemes and tables S4
2.1. Epoxide **4** opening reaction S4
2.2. Optimization of benzoate deprotection in **8** S4
2.3. Mitsunobu reaction screening of *bis*-homoallylic alcohol **6** S5
2.4. Unexpected result of epoxide **A‘** opening reaction. S6
2.5. Synthesis of aldehyde **7** S6
2.6. Synthesis of allylic ether **19** S6
3. Experimental Procedures and Compound Data S7
3.1. General procedure for the Katsuki-Sharpless epoxidation of allylic alcohols S7
3.2. General procedure for TBDPS silylation of alcohols S7
3.3. General procedure for the preparation of *bis*-homoallylic alcohols S7
3.4. General procedure for Prins-Peterson cyclization S8

3.5. General procedure for benzoate deprotection S8
3.6. General procedure for bromination of the alcohol S8
4. Comparison of NMR data of natural and synthetic (+)-isolaurepinnacin (1) S26
5. Comparison of NMR data of natural and synthetic (+)-neoisoprelaurefucin (2) S27
6. References S28
7. NMR spectra S29

1. General Experimental Information

1.1. Reagents and solvents

Reagents were obtained from commercial sources (*Sigma-Aldrich*, *Merck*, *Alfa Aesar*), without further purification. *Tert*-butylhydroperoxide (TBHP) was prepared by the method of Sharpless *et al*.[1]

Solvents (THF, DCM, Et_2O, toluene and DMF) were used from the PureSolv system. The dispensing system allows easy access to the anhydrous solvents. Other solvents like MeOH and acetonitrile were purified by distillation and dried following the procedure in the literature.[2]

1.2. Chromatography

Chemical reactions and the separation of the crudes were monitored by thin-layer chromatography (TLC). The TLC was performed on aluminium foil sheets 60 F254 manufactured by MERCK. The solvent or solvent mixture were *n*-hexane/ethyl acetate (EtOAc) in different ratios.
Flash column chromatography was performed using silica gel (0.015-0.04 mm) and *n*-hexane/EtOAc solvent systems.
Automated flash column chromatography was performed using the Biotage® Isolera™ System (Isolera Prime). It includes simultaneous UV-detection on all wavelengths and baseline correction, which enable detection of poor UV absorbing compounds.

1.3. Nuclear magnetic resonance (NMR)

NMR spectra were recorded on Bruker Avance instruments. ^{1}H NMR spectra were recorded at 400, 500 and 600 MHz, and ^{13}C NMR were recorded at 100, 125 and 150 MHz, VTU 298.0 ºK. The residual solvent peak was used as an internal reference ($CDCl_3$: δ_H 7.26, δ_C 77.0).[3]

1.4. Mass spectrometry

High-resolution mass spectra were recorded on an LCT Premier XE mass spectrometer. It is provided with different ionization sources: electrospray (ESI), an atmospheric pressure chemical ionization (APCI) source and an orthogonal acceleration time of flight (oa-TOF) analyzer that provides high sensitivity, resolution and accurate mass measurement.

1.5. Optical rotation

Optical rotations were measured on a Perkin-Elmer 343 plus polarimeter by using a sodium lamp, and a MCP 150 Anton Paar polarimeter equipped with a LED light of 589 nm and a cell with automatic Peltier temperature control was also used. 100 and 10 mm long cells were used. Solutions of the different compounds were prepared, using the solvents and concentrations indicated for each case.

2. Supporting schemes and tables

2.1. Epoxide **4** opening reaction

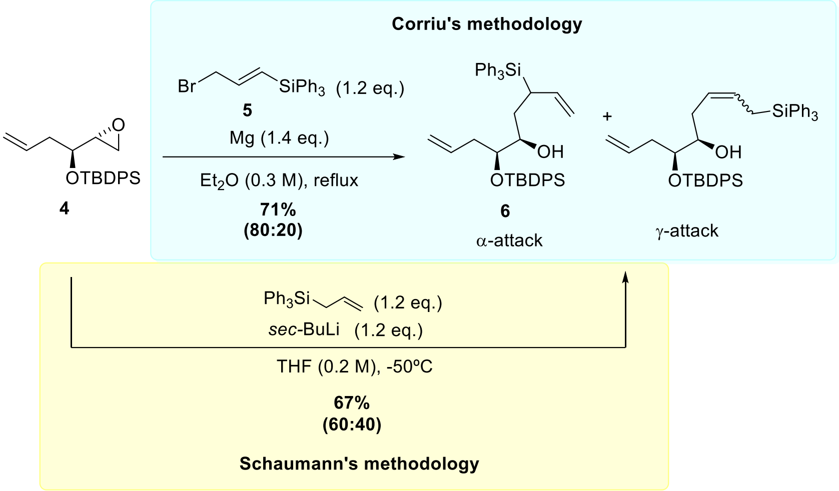

Scheme S1. Comparison of epoxide **4** opening through Corriu's method *vs* Schaumann's methodology.

2.2. Optimization of benzoate deprotection in **8**

TableS1. Optimization of reaction conditions for the deprotection of the benzoate group.

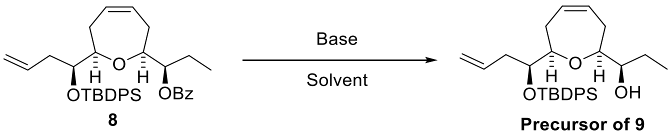

Entry	Base (equiv.)	Solvent (M)	Temperature (ºC)	Yield **Precursor of 9** (%)[a]	Conversion **8** (%)[a]
1	K_2CO_3 (1.0)	MeOH (0.1)	r.t.	40	95
2	K_2CO_3 (5.0)	MeOH (0.05)	40	55	100
3	K_2CO_3 (5.0)	MeOH (0.05)	55	61	100
4	KOH (2.0)	MeOH/H_2O (0.08)	r.t.	9	100
5	LiOH (5.0)	THF/MeOH/H_2O 3:1:1 (0.05)	r.t.	32	48
6	Mg (6.0)	MeOH (0.05)	r.t.	26	63
7	Na (6.0)	MeOH (0.05)	r.t.	70	100

[a] Yield and conversion of the isolated products.

2.3. Mitsunobu reaction screening of *bis*-homoallylic alcohol **6**

Table S2. Different reaction conditions for the inversion of *bis*-homoallylic alcohol **6**.

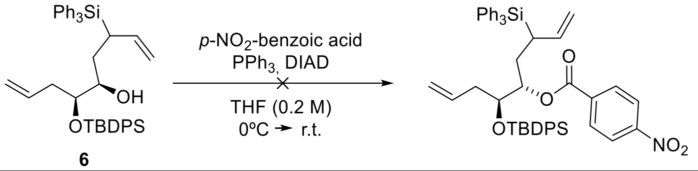

6

Entry	Reagents[a]	Solvent (M)	Temperature	Time (h)	Yield (%)
1	[a] Alcohol **6**, Ph_3P, *p*-NO_2-benzoic acid, DIAD	Toluene (0.2)	0ºC → r.t. Then reflux	48	-
2	[a] Alcohol **6**, Ph_3P, ácido acético, DIAD	Toluene (0.2)	r.t.	48	-
3	[b] Alcohol **6**, Ph_3P, *p*-NO_2-benzoic acid, DIAD[4]	Benzene (0.05)	r.t.	48	-
4	[b] Alcohol **6**, Ph_3P, *p*-NO_2-benzoic acid, DIAD[5]	THF (0.1)	0ºC → r.t.	48	-
5	[c] Alcohol **6**, Ph_3P, *p*-NO_2-benzoic acid, DIAD[6]	THF (3.0)	r.t.[c]	48	-
6	[c] Alcohol **6**, Ph_3P, DIAD, *p*-NO_2-benzoic acid[7]	THF (0.07)	0ºC	24	-

[a]Reaction conditions: alcohol **6** (1.0 equiv.), PPh_3 (1.2 equiv.), acid (1.2 equiv.) and DIAD (1.2 equiv.). [b]Reaction conditions: alcohol **6** (1.0 equiv.), PPh_3 (6.0 equiv.), acid (6.0 equiv.) and DIAD (6.0 equiv.). [c]Reaction conditions: alcohol **6** (1.0 equiv.), PPh_3 (2.0 equiv.), acid (2.0 equiv.) and DIAD (2.0 equiv.), the reaction mixture was sonicated. The addition order agrees with the order in which the reactants appear in the table.

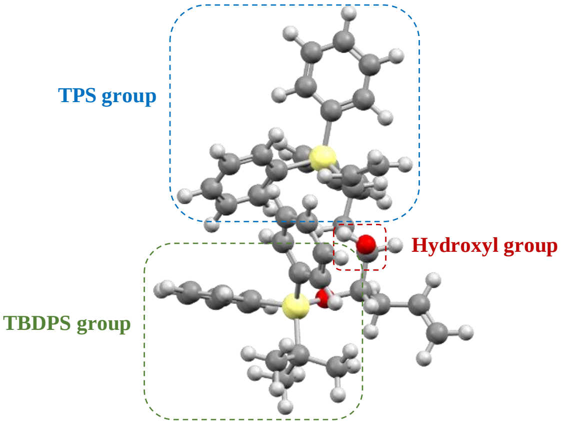

Figure **S1**. Steric hindrance of hydroxyl group of alcohol **6**.

2.4. Unexpected result of epoxide A' opening reaction.

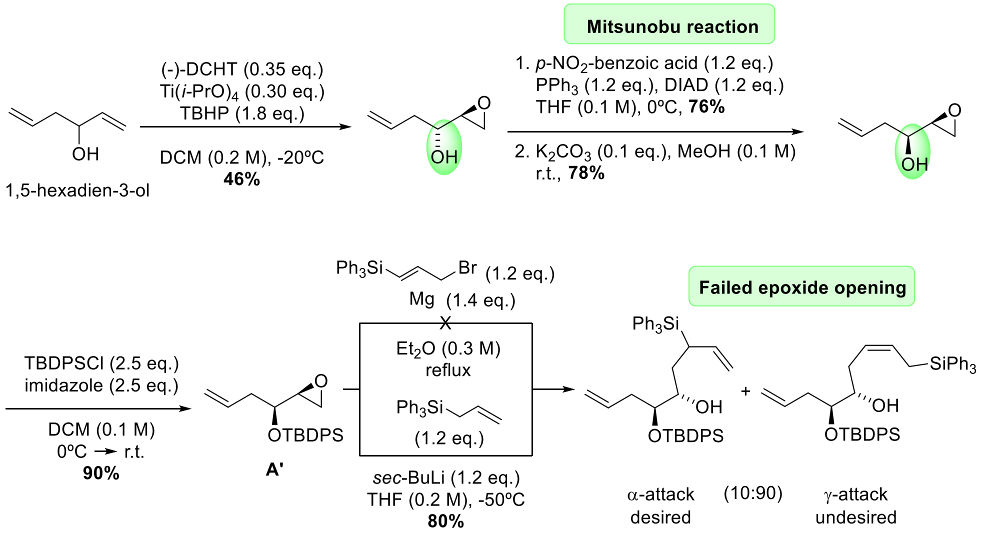

Scheme S2. Unexpected favored opening reaction of γ-attack.

2.5. Synthesis of aldehyde **7**

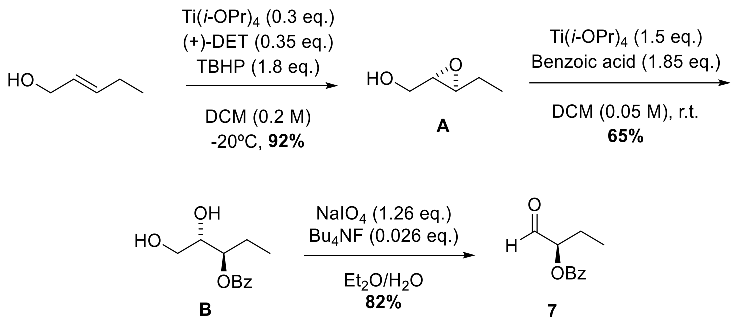

Scheme S3. Synthetic route for aldehyde **7**.

2.6. Synthesis of allylic ether **19**

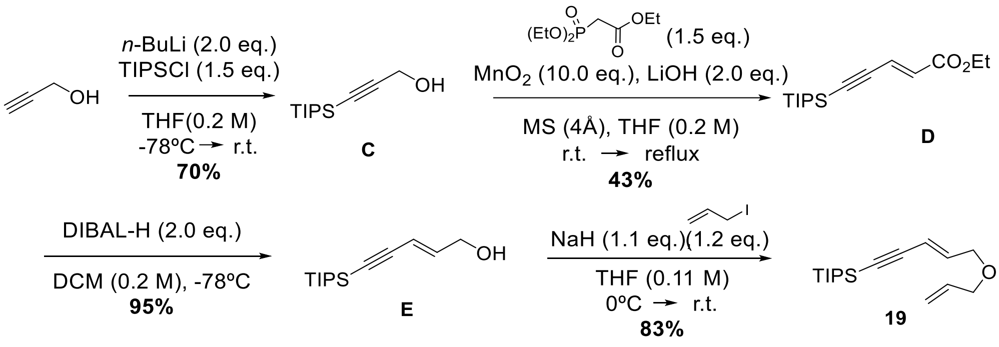

Scheme S4. Synthetic route for allylic ether **19**.

3. Experimental Procedures and Compound Data

3.1. General procedure for the Katsuki-Sharpless epoxidation of allylic alcohols

Following the procedure described by Sharpless *et al.*,[8] 4Å powdered activated molecular sieves (MS) was added into a round-bottom flask equipped with a dropping funnel. Next, DCM (0.2 M) and Ti(*i*-OPr)$_4$ (0.30 equiv.) were added, and the reaction mixture was cooled to -20°C under an inert atmosphere. After 5 minutes, the corresponding tartrate ((+)-dicyclohexyl tartrate (DCHT), (+)- or (-)-diethyltartrate (DET) (0.35 equiv.)) was added and the mixture was stirred, at -20ºC, another 5 min. Then, the allylic alcohol was added, and the mixture was stirred 20 minutes. Finally, the prepared *tert*-butylhydroperoxide (TBHP) 5.8 M in isooctane (1.8 equiv.) was added dropwise, and the flask was placed in the freezer overnight at -20°C. Once the reaction was complete (checked by TLC), the suspension was filtered through a short plug of Celite®. Then, it was poured in a mixture of $Fe_2(SO_4)_3$, aqueous citric acid 1.0 M and water cooled to 0ºC; the mixture was stirred for 30 min. Next, the organic phase was separated and concentrated under reduced pressure. The crude material was dissolved in Et_2O and cooled to 0 °C. Aqueous 15% NaOH was added, and the mixture was stirred for only 1 min. The organic layer was separated and extracted with 2 × Et_2O and 2 × EtOAc, dried over $MgSO_4$, and concentrated under reduced pressure. The reaction mixture was purified by flash silica gel column chromatography (*n*-hexane/EtOAc 70:30 solvent system).

3.2. General procedure for TBDPS silylation of alcohols

To a solution of the corresponding alcohol (1.0 equiv.) in dry DCM (0.1 M), was added imidazole (1.5-2.5 equiv.) and the mixture was stirred until it was totally solved. Then, the mixture was cooled to 0 ºC and TBDPSCl (1.5-2.5 equiv.) was added dropwise. The mixture was allowed to reach room temperature and stirred overnight. The quenching was performed by addition of water. Then, the mixture was extracted with 3 x DCM, dried over $MgSO_4$, filtered and concentrated under reduced pressure. The reaction mixture was purified by flash silica gel column chromatography (*n*-hexane/EtOAc 95:5 solvent system).

3.3. General procedure for the preparation of *bis*-homoallylic alcohols

Following the procedure described by Corriu *et al.*,[9,10] to a solution of Mg (2.4 equiv.) in dry Et_2O (0.3 M) was added allyl-TPS bromide **8** (2.0 equiv.) dissolved in dry Et_2O (1.0 M). The reaction mixture was heated to reflux for 2 h (Heat-On™ Block System). Then, epoxide (1.0 equiv.) was dissolved in Et_2O (1.0 M) and added dropwise at the refluxing temperature. Once the reaction was complete (checked by TLC), it was allowed to cool to room temperature and quenched with saturated aqueous NH_4Cl. The mixture

was extracted with Et_2O and the organic phase was dried over $MgSO_4$, filtered and concentrated under reduced pressure. This crude reaction mixture was purified by flash silica gel column chromatography (*n*-hexane/EtOAc 95:5 solvent system).

3.4. General procedure for Prins-Peterson cyclization

Following the procedure developed in our group,[11] to a solution of *bis*-homoallylsilyl alcohols (1.0 equiv) in dry DCM (0.1 M) at -20ºC or -30ºC were added the corresponding aldehyde (1.5 equiv.) and $FeBr_3$ (1.2 equiv.). The reaction was monitored by TLC, and once it was complete, the process was quenched by the addition of water and DCM. The organic phase was separated and the aqueous layer was extracted with DCM three times. Then, it was dried over $MgSO_4$, filtered and the solvent was removed under reduced pressure. The crude reaction mixture was purified by flash silica gel column chromatography (*n*-hexane/EtOAc 98:2 solvent system).

3.5. General procedure for benzoate deprotection

To a solution of benzoate-oxepene (1.0 equiv.) in dry MeOH (0.05M) at room temperature was added sodium (6.0 eq.). Once the reaction was completed (monitored by TLC), it was concentrated under reduced pressure. Water and DCM were added and then the mixture was extracted with 3 x DCM and 2 x EtOAc, dried over $MgSO_4$ and concentrated under reduced pressure. The reaction mixture was purified by flash silica gel column chromatography (*n*-hexane/EtOAc 95:5 solvent system).

3.6. General procedure for bromination of the alcohol

Following a modified procedure described by Hammond,[12] to a solution of alcohol (1.0 equiv.) and carbon tetrabromide (1.25 equiv.) in dry DCM (0.66 M) at room temperature and under an inert atmosphere was added triphenylphosphine (1.5 equiv.) in small portions during 1.5 h. Then, the solvent was removed under reduced pressure and Et_2O was added. The solid was filtrated through a short plug of Celite®. The solid was washed with Et_2O and the organic phase was concentrated under vacuum. Then, he reaction mixture was purified by flash silica gel column chromatography (*n*-hexane/EtOAc 98:2 solvent system).

3.7. Compound data

(*S*)-1-((*R*)-oxiran-2-yl)but-3-en-1-ol (precursor of 4)

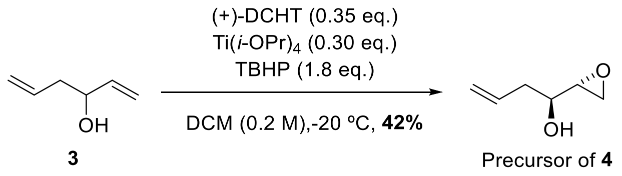

Following the general procedure 3.1, to a solution of Ti(*i*-OPr)$_4$ (1.81 mL, 6.11 mmol, 0.30 equiv.) and (+)-dicyclohexyl tartrate (DCHT) (2.24 g, 7.13 mmol, 0.35 equiv.) were added the allylic alcohol (2.00 g, 20.4 mmol, 1.0 equiv.) and the prepared *tert*-butylhydroperoxide (TBHP) 5.8 M (6.3 mL, 36.7 mmol, 1.8 equiv.) to give 0.978 g of the precursor epoxialcohol of **4** as a yellow oil (8.57 mmol, 42% yield). Spectral data was consistent with the literature.[11,13]

R$_f$ = 0.38 (*n*-hexane/EtOAc 70:30); **[α]$^{25}_D$** = +26.5 (*c* = 1.0, CH_2Cl_2). Literature value: [α]$^{25}_D$ = +30.1 (*c* = 1.0, CH_2Cl_2);[13] **^{1}H-NMR (CDCl$_3$, 400 MHz)** δ = 5.89 (ddt, *J* = 17.2, 10.1 & 7.1 Hz, 1H), 5.22-5.13 (m, 2H), 3.85 (m, 1H), 3.04 (q, *J* = 3.4 Hz, 1H), 2.81 (dd, *J* = 5.1 & 2.8 Hz, 1H), 2.76 (m, 1H), 2.46-2.37 (m, 1H), 2.36-2.27 (m, 1H), 1.88 (brs, 1H). **^{13}C-NMR (CDCl$_3$, 100 MHz)** δ = 133.6 (CH), 118.3 (CH_2), 68.2 (CH), 54.1 (CH), 43.8 (CH_2), 38.1 (CH_2). **HRMS (ESI+):** m/z calcd. for $C_6H_{10}O_2Na^+$: 137.0578 [*M*+Na]$^+$; found: 137.0578.

(*R*)-1-((*S*)-oxiran-2-yl)but-3-en-1-ol (precursor of ent-4)

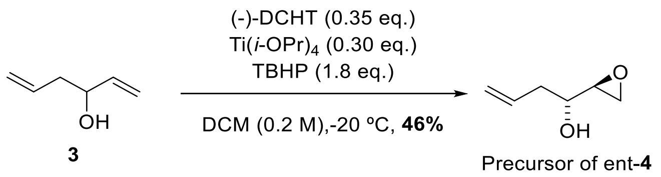

Following the general procedure 3.1, to a solution of Ti(*i*-OPr)$_4$ (0.91 mL, 3.06 mmol, 0.30 equiv.) and (-)-dicyclohexyl tartrate (DCHT) (1.12 g, 3.57 mmol, 0.35 equiv.) in 51 mL of dry DCM (0.2 M) were added the allylic alcohol (1.00 g, 10.2 mmol, 1.0 equiv.) and prepared *tert*-butylhydroperoxide (TBHP) 5.8 M (3.2 mL, 18.4 mmol, 1.8 equiv.) to give 0.535 g of the precursor epoxialcohol of **ent-4** as a yellow oil (4.69 mmol, 46% yield). Spectral data was consistent with the literature.[14,15]

R$_f$ = 0.38 (*n*-hexane/EtOAc 70:30); **[α]$^{25}_D$** = -26.0 (c = 1.00, $CHCl_3$). Literature value: [α]$^{25}_D$ = -28.0 (c = 9.65, CH_2Cl_2);[15] **^{1}H-NMR (CDCl$_3$, 400 MHz)** δ = 5.87 (ddt, *J* = 17.2, 9.9 & 7.1 Hz, 1H), 5.16 (m, 2H), 3.84 (m, 1H), 3.03 (q, *J* = 3.1 Hz, 1H), 2.80 (m, 1H), 2.75 (m, 1H), 2.40 (m, 1H), 2.31 (m, 1H), 1.97 (brs, 1H). **^{13}C-NMR (CDCl$_3$, 100 MHz)** δ = 133.5 (CH), 118.2 (CH_2), 68.1 (CH), 54.0 (CH), 43.8 (CH_2), 38.1 (CH_2). **HRMS (ESI+):** m/z calcd. for $C_6H_{10}O_2Na^+$: 137.0578 [*M*+Na]$^+$; found: 137.0581.

***Tert*-butyl(((*S*)-1-((*R*)-oxiran-2-yl)but-3-en-1-yl)oxy)diphenylsilane (4)**

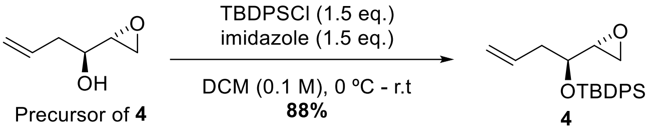

Following the general procedure 3.2, to a solution of the precursor epoxialcohol of **4** (1.14 g, 10.0 mmol, 1.0 equiv.) in dry DCM (100 mL, 0.1 M), was added imidazole (1.70 g, 25.0 mmol, 2.5 equiv.) and TBDPSCl (6.80 mL, 25.0 mmol, 2.5 equiv.) to obtain 3.10 g of epoxide **4** as a colorless oil (8.80 mmol, 88% yield). Spectral data was consistent with the literature.[11]

R_f = 0.64 (*n*-hexane/EtOAc 90:10); **$[\alpha]^{25}_D$** = +29.2 (*c* = 1.30, $CHCl_3$). Literature value: $[\alpha]^{25}_D$ = +32.2 (c = 1.3, $CHCl_3$);[11] **^{1}H-NMR ($CDCl_3$, 400 MHz)** δ = 7.68 (brddd, *J* = 9.5, 7.9 & 1.5 Hz, 4H), 7.47-7.34 (m, 6H), 5.88 (m, 1H), 5.07 (m, 2H), 3.47 (q, *J* = 5.6 Hz, 1H), 2.90 (ddd, *J* = 6.3, 3.6 & 2.8 Hz, 1H), 2.46 (dd, *J* = 5.1 & 3.9 Hz, 1H), 2.36 (m, 2H), 2.15 (dd, *J* = 5.1 & 2.6 Hz, 1H), 1.06 (s, 9H); **^{13}C-NMR ($CDCl_3$, 100 MHz)** δ = 135.9 (4 x CH), 133.8 (C), 133.8 (CH), 133.7 (C), 129.8 (CH), 129.7 (CH), 127.6 (2 x CH), 127.5 (2 x CH), 117.6 (CH_2), 72.8 (CH), 53.9 (CH), 46.2 (CH_2), 39.8 (CH_2), 26.9 (3 x CH_3), 19.4 (C); **HRMS (ESI+):** m/z calcd. for $C_{22}H_{28}O_2SiNa^+$: 375.1756 [*M*+Na]$^+$; found: 375.1758.

***Tert*-butyl(((*R*)-1-((*S*)-oxiran-2-yl)but-3-en-1-yl)oxy)diphenylsilane (ent-4)**

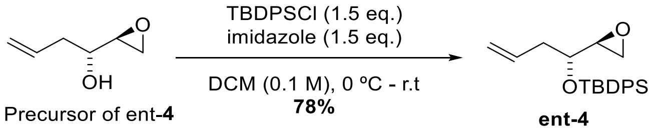

Following the general procedure 3.2, to a solution of the precursor epoxialcohol of **ent-4** (3.23 g, 28.3 mmol, 1.0 equiv.) in 283 mL of dry DCM (0.1 M), was added imidazole (2.89 g, 42.5 mmol, 1.5 equiv.) and TBDPSCl (11.4 mL, 42.5 mmol, 2.5 equiv to obtain 7.79 g of epoxide **ent-4** as a colorless oil (22.1 mmol, 78% yield).

R_f = 0.64 (*n*-hexane/EtOAc 90:10); **$[\alpha]^{25}_D$** = -32.6 (*c* = 0.90, $CHCl_3$); **^{1}H-NMR ($CDCl_3$, 400 MHz)** δ = 7.69 (m, 4H), 7.43 (m, 2H), 7.38 (m, 4H), 5.89 (m, 1H), 5.07 (m, 2H), 3.48 (q, *J* = 5.6 Hz, 1H), 2.91 (m, 1H), 2.46 (dd, *J* = 5.0 & 4.0 Hz, 1H), 2.37 (m, 2H), 2.15 (dd, *J* = 5.2 & 2.6 Hz, 1H), 1.06 (s, 9H); **^{13}C-NMR ($CDCl_3$, 100 MHz)** δ = 135.9 (4 x CH), 133.8 (C), 133.7 (CH), 133.6 (C), 129.8 (CH), 129.7 (CH), 127.6 (2 x CH), 127.5 (2 x CH), 117.6 (CH_2), 72.8 (CH), 53.9 (CH), 46.2 (CH_2), 39.8 (CH_2), 26.9 (3 x CH_3), 19.4 (C); **HRMS (ESI+):** m/z calcd. for $C_{22}H_{28}O_2SiNa^+$: 375.1756 [*M*+Na]$^+$; found: 375.1756.

(*E*)-(3-bromoprop-1-en-1-yl)triphenylsilane (5)

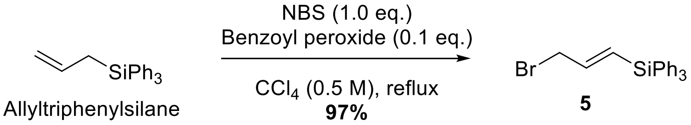

Following the procedure described by Corriu,[9,10] to a solution of commercially available allyltriphenylsilane (6.00 g, 20.0 mmol, 1.0 equiv.) in carbon tetrachloride (CCl_4, 40.0 mL, 0.5 M) at room temperature, were added *N*-bromosuccinimide (NBS, 3.56 g, 20.0 mmol, 1.0 equiv.) and benzoyl peroxide (0.485 g, 2.00 mmol, 0.1 equiv.). The mixture reaction was heated to reflux (Heat-On™ Block System) and monitored by TLC. Then, it was allowed to cool to room temperature and filtered through a short plug of Celite®. The solvent was removed under reduced pressure and the mixture reaction was purified by flash silica gel column chromatography (*n*-hexane/EtOAc 95: 5 solvent system) to afford 7.36 g of allyl-TPS bromide **5** as a white solid (19.4 mmol, 97% yield).

R_f = 0.55 (*n*-hexane/EtOAc 95:5); **^{1}H-NMR ($CDCl_3$, 400 MHz)** δ = 7.51 (m, 6H), 7.46-7.35 (m, 9H), 6.50 (d, *J* = 18.1 Hz, 1H), 6.27 (dt, *J* = 18.2 & 6.7 Hz, 1H), 4.06 (dd, *J* = 6.7 & 0.6 Hz, 2H); **^{13}C-NMR ($CDCl_3$, 125 MHz)** δ = 146.0 (CH), 135.9 (6 x CH), 133.7 (3 x C), 129.7 (3 x CH), 129.6 (CH), 128.0 (6 x CH), 34.7 (CH_2); **HRMS (ESI+):** m/z calcd. for $C_{21}H_{19}BrSiNa^+$: 401.0337 [*M*+Na]$^+$; found: 401.0341.

(5*R*,6*S*)-6-((*tert*-butyldiphenylsilyl)oxy)-3-(triphenylsilyl)nona-1,8-dien-5-ol (6)

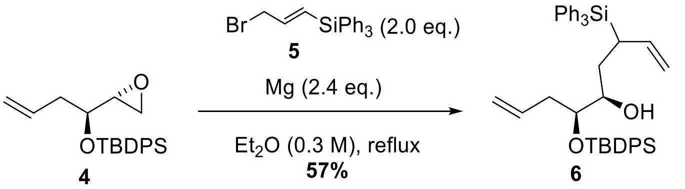

Following the general procedure 3.3, epoxide **4** (3.50 g, 9.93 mmol, 1.0 equiv.) was added to a solution of Mg (0.579 g, 23.8 mmol, 2.4 equiv.) and allyl-TPS bromide **5** (7.55 g, 19.9 mmol, 2.0 equiv.) in 33 mL of dry Et_2O (0.3 M) to give 3.70 g of *bis*-homoallylic alcohol **6** as a colorless oil (5.66 mmol, 57% yield). Spectral data was consistent with the literature.[11]

R_f = 0.44 (*n*-hexane/EtOAc 95:5); **^{1}H-NMR ($CDCl_3$, 400 MHz)** δ = 7.62-7.54 (m, 10H), 7.41-7.31 (m 15H), 5.65-5.55 (dt, *J* = 17.0 & 9.8 Hz, 1H), 5.55-5.47 (m, 1H), 4.93-4.72 (m, 4H), 3.74 (m, 1H), 3.64 (m, 1H), 2.89 (t, *J* = 10.4 Hz, 1H), 2.14 (m, 1H), 2.04 (m, 1H), 1.88-1.82 (d, *J* = 12.3 Hz, 1H), 1.79 (d, *J* = 6.0 Hz, 1H), 1.49 (d, *J* = 12.8 Hz, 1H), 0.99 (s, 9H); **^{13}C-NMR ($CDCl_3$, 100 MHz)** δ = 137.9 (CH), 136.1 (8 x CH), 135.9 (CH), 135.8 (CH), 135.7 (CH), 134.5 (CH), 133.9 (4 x C), 133.6 (C), 129.8 (CH), 129.7 (3 x CH), 127.8 (CH), 127.7 (7 x CH), 127.7 (CH), 127.5 (CH), 117.0 (CH_2), 115.2 (CH_2), 76.3 (CH), 71.5 (CH), 37.3 (CH_2), 30.6 (CH_2), 27.1 (CH), 27.0 (3 x CH_3), 19.4 (C); **HRMS (ESI+):** m/z calcd. for $C_{43}H_{48}O_2Si_2Na^+$: 675.3091 [*M*+Na]$^+$; found: 675.3098.

(5*S*,6*R*)-6-((*tert*-butyldiphenylsilyl)oxy)-3-(triphenylsilyl)nona-1,8-dien-5-ol (ent-6)

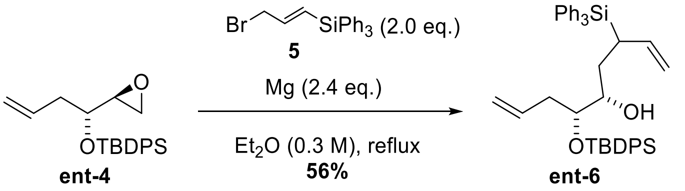

Following the general procedure 3.3, epoxide **ent-4** (1.50 g, 4.25 mmol, 1.0 equiv.) was added to a solution of Mg (0.248 g, 10.2 mmol, 2.4 equiv.) and allyl-TPS bromide **5** (3.22 g, 8.50 mmol, 2.0 equiv.) in 14.2 mL of dry Et_2O (0.3 M) to afford 1.55 g of *bis*-homoallylic alcohol **ent-6** as a colorless oil (2.38 mmol, 56%).

R_f = 0.47 (*n*-hexane/EtOAc 95:5); **^{1}H-NMR ($CDCl_3$, 400 MHz)** δ = 7.63 (t, *J* = 6.7 Hz, 4H), 7.52 (d, *J* = 6.8 Hz, 6H), 7.45-7.30 (m, 15H), 5.77 (dt, *J* = 17.1 & 9.8 Hz, 1H), 5.61 (m, 1H), 4.88-4.72 (m, 4H), 3.78 (m, 2H), 2.33 (brt, *J* = 9.3 Hz, 1H), 2.19-2.16 (d, *J* = 2.4 Hz, 1H), 2.16-2.11 (m, 1H), 2.00 (m, 1H), 1.88 (m, 1H), 1.83-1.74 (m, 1H), 1.03 (s, 9H); **^{13}C-NMR ($CDCl_3$, 100 MHz)** δ = 139.0 (CH), 136.1 (8 x CH), 136.0 (CH), 135.9 (CH), 135.1 (CH), 133.9 (C), 133.6 (C), 133.4 (3 x C), 129.7 (CH), 129.6 (CH), 129.5 (3 x CH), 127.8 (8 x CH), 127.6 (CH), 127.5 (CH), 116.7 (CH_2), 114.7 (CH_2), 75.0 (CH), 74.9 (CH), 35.8 (CH_2), 31.5 (CH_2), 29.7 (CH), 27.1 (3 x CH_3), 19.4 (C); **HRMS (ESI+):** m/z calcd. for $C_{43}H_{48}O_2Si_2Na^+$: 675.3091 [*M*+Na]$^+$; found: 675.3091.

((2*S*,3*S*)-3ethyloxiran-2-yl)methanol (A)

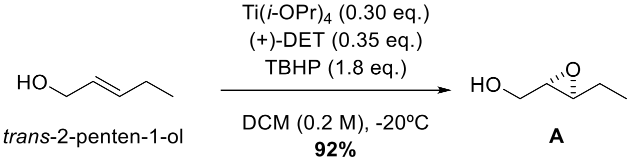

Following the general procedure 3.1, to solution of Ti(*i*-OPr)$_4$ (1.55 mL, 5.22 mmol, 0.3 equiv.), (+)-DET (1.04 mL, 6.09 mmol, 0.35 equiv.), and *trans*-2-penten-1-ol 95% (1.86 mL, 17.4 mmol, 1.0 equiv.) in 87 mL of dry DCM (0.2 M) was added dropwise TBHP 5.8 M in isooctane (5.4 mL, 31.3 mmol, 1.8 equiv.) to obtain 1.63 g of epoxialcohol **A** as a colorless oil (16.0 mmol, 92% yield). Spectral data was consistent with the literature.[16]

R_f = 0.36 (*n*-hexane/acetone 70:30); **$[\alpha]^{25}_D$** = -27.5 (*c* = 1.0, EtOH) Literature value: $[\alpha]^{22}_D$ = -31.3 (c = 0.56, EtOH);[16] **^{1}H-NMR ($CDCl_3$, 400 MHz)** δ = 3.91 (d, *J* = 12.3 Hz, 1H), 3.61 (dd, *J* = 12.5 & 2.9 Hz, 1H), 2.94 (m, 2H), 1.94 (brs, 1H), 1.61 (m, 2H), 1.00 (t, *J* = 7.5 Hz, 3H); **^{13}C-NMR ($CDCl_3$, 100 MHz)** δ = 61.7 (CH_2), 58.1 (CH), 57.0 (CH), 24.6 (CH_2), 9.8 (CH_3); **HRMS (ESI+):** m/z calcd. for $C_5H_{10}O_2Na^+$: 125.0578 [*M*+Na]$^+$; found: 125.0579.

(2*S*,3*R*)-1,2-dihydroxypentan-3-yl benzoate (B)

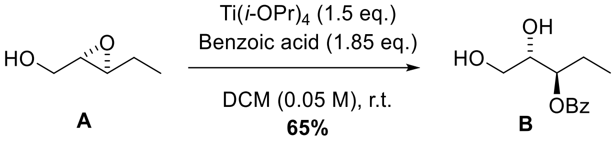

To a solution of Ti(*i*-OPr$_4$) (6.54 mL, 22.1 mmol, 1.5 equiv.) and the epoxialcohol **A** (1.50 g, 14.7 mmol, 1.0 equiv.) in 294 mL of dry DCM (0.05 M) at room temperature, was added benzoic acid (3.32 g, 27.2 mmol, 1.85 equiv.). The mixture reaction was stirred and monitored by TLC. Once it was completed, aqueous 15% tartaric acid was added. Then the mixture was extracted with 3 x DCM and 2 x EtOAc,

dried over $MgSO_4$ and concentrated under reduced pressure. The reaction mixture was purified by flash silica gel column chromatography (*n*-hexane/EtOAc 40:60 solvent system) to obtain 2.14 g of the diol **B** as a colorless oil (9.56 mmol, 65% yield). Spectral data was consistent with the literature.[11]

R$_f$ = 0.45 (*n*-hexane/EtOAc 50:50); **[α]$^{25}_D$** = +2.5 (*c* = 1.02, EtOH). Literature value: $[\alpha]^{25}_D$ = +3.1 (*c* = 1.07, EtOH);[11] **^{1}H-NMR ($CDCl_3$, 400 MHz)** δ = 8.05 (dd, *J* = 8.2 & 1.2 Hz, 2H), 7.59 (m, 1H), 7.46 (m, 2H), 5.03 (ddd, *J* = 8.7, 6.9 & 3.5 Hz, 1H), 3.78-3.73 (m, 1H), 3.73-3.68 (dd, *J* = 11.9 & 3.1 Hz, 1H), 3.62 (dd, *J* = 11.9 & 4.8 Hz, 1H), 2.79 (brs, 1 H), 1.95 (m, 1H), 1.82 (m, 1H), 1.01 (t, *J* = 7.4 Hz, 3H); **^{13}C-NMR ($CDCl_3$, 100 MHz)** δ = 167.4 (C), 133.4 (CH), 129.7 (2 x CH), 128.5 (2 x CH), 76.1 (CH), 72.8 (CH), 62.6 (CH_2), 23.9 (CH_2), 9.8 (CH_3); **HRMS (ESI+):** m/z calcd. for $C_{12}H_{16}O_4Na^+$: 247.0946 [*M*+Na]$^+$; found: 247.0946.

(*R*)-1-oxobutan-2-yl benzoate (7)

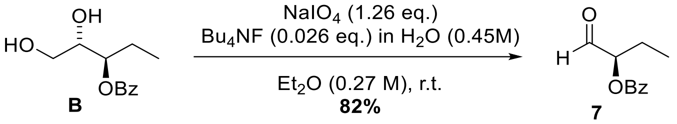

To a solution of previous diol **B** (1.55 g, 6.91 mmol, 1.0 equiv.) in 26 mL of Et_2O (0.27 M) at room temperature was added a solution of $NaIO_4$ (1.86 g, 8.71 mmol, 1.26 equiv.) and Bu_4NF (0.18 mL, 0.18 mmol, 0.026 equiv.) in 15 mL of H_2O (0.45 M). The reaction mixture was stirred and monitored by TLC. The quenching was made diluting the reaction mixture with Et_2O and H_2O. Next, the organic layer was separated, and the aqueous phase was extracted with 3 x Et_2O. The combined organic layers were dried over $MgSO_4$ and concentrated under reduced pressure. The reaction mixture was purified by flash silica gel column chromatography (*n*-hexane/EtOAc 80:20 solvent system) to afford 1.09 g of aldehyde **7** as a pale yellow oil (5.67 mmol, 82% yield). Spectral data was consistent with the literature.[17]

R$_f$ = 0.74 (*n*-hexane/EtOAc 40:60); **[α]$^{25}_D$** = + 39.3 (*c* = 1.00, $CHCl_3$). Literature value: $[\alpha]^{20}_D$ = +41.0 (*c* = 1.00, $CHCl_3$);[17] **^{1}H-NMR ($CDCl_3$, 500 MHz)** δ = 9.65 (d, *J* = 0.6 Hz, 1H), 8.11 (dd, *J* = 8.2 & 1.2 Hz, 2H), 7.61 (m, 1H), 7.48 (m, 2H), 5.18 (ddd, *J* = 7.8, 5.1 & 0.7 Hz, 1H), 2.07-1.99 (m, 1H), 1.98-1.90 (m, 1H), 1.10 (t, *J* = 7.4 Hz, 3H); **^{13}C-NMR ($CDCl_3$, 125 MHz)** δ = 198.5 (C), 166.2 (C), 133.5 (CH), 129.8 (2 x CH), 128.5 (2 x CH), 79.7 (CH), 22.4 (CH_2), 9.4 (CH_3); **HRMS (ESI$^+$):** *m/z* calcd. for $C_{11}H_{12}O_3Na^+$: 215.0684 [*M*+Na]$^+$; found: 215.0686.

(*R*)-1-((2*S*,7*R*)-7-((*S*)-1-((*tert*-butyldiphenylsilyl)oxy)but-3-en-1-yl)-2,3,6,7-tetra hydrooxepin-2-yl)propyl benzoate (8)

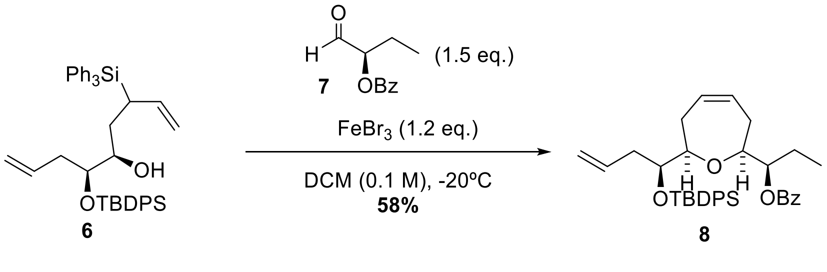

Following the general procedure 3.4, to a solution of *bis*-homoallylic alcohol **6** (0.448 g, 0.69 mmol, 1.0 equiv.) in dry DCM (6.9 mL, 0.1 M) at -20ºC, aldehyde **7** (0.200 g, 1.04 mmol, 1.5 equiv.) and $FeBr_3$ (0.245 g, 0.83 mmol, 1.2 equiv.) were added to obtain 0.228 g of the oxepene **8** as a colorless oil (0.40 mmol, 58% yield). Spectral data was consistent with our own data in the literature.[11]

R$_f$ = 0.59 (*n*-hexane/EtOAc 90:10); **[α]$^{25}_D$** = + 4.9 (*c* = 1.00, $CHCl_3$). Literature value: [α]$^{25}_D$ = +5.6 (*c* = 0.97, $CHCl_3$);[11] **^{1}H-NMR ($CDCl_3$, 400 MHz)** δ = 8.02 (m, 2H), 7.70 (ddd, *J* = 15.7, 8.0 & 1.4 Hz, 4H), 7.55 (m, 1H), 7.45-7.34 (m, 8H), 5.80-5.71 (m, 2H), 5.69-5.57 (ddt, *J* = 17.4, 10.4 & 7.0 Hz, 1H), 5.00 (ddd, *J* = 8.1, 5.7 & 4.1 Hz, 1H), 4.85 (m, 2H), 3.82 (dt, *J* = 6.4 & 4.4 Hz, 1H), 3.49 (ddd, *J* = 8.9, 5.7 & 3.0 Hz, 1H), 3.34 (dt, *J* = 8.2 & 4.0 Hz, 1H), 2.38-2.33 (m, 2H), 2.31-2.26 (m, 3H), 2.17 (m, 1H), 1.73 (m, 2H), 1.06 (s, 9H), 0.89 (t, *J* = 7.4 Hz, 3H). **^{13}C-NMR ($CDCl_3$, 100 MHz)** δ = 166.1 (C), 136.2 (2 x CH), 136.1 (2 x CH), 134.6 (CH), 134.5 (C), 133.8 (C), 132.8 (CH), 130.6 (C), 129.8 (CH), 129.6 (2 x CH), 129.5 (CH), 129.5 (CH), 128.6 (CH), 128.3 (2 x CH), 127.4 (2 x CH), 127.3 (2 x CH), 116.9 (CH_2), 82.0 (CH), 80.4 (CH), 77.9 (CH), 76.1 (CH), 38.3 (CH_2), 33.1 (CH_2), 31.9 (CH_2), 27.1 (3 x CH_3), 23.4 (CH_2), 19.5 (C), 9.8 (CH_3). **HRMS (ESI$^+$):** *m/z* calcd. for $C_{36}H_{44}O_4SiNa^+$: 591.2907 [*M*+Na]$^+$; found: 591.2907.

(*R*)-1-((2*S*,7*R*)-7-((*S*)-1-((*tert*-butyldiphenylsilyl)oxy)but-3-en-1-yl)-2,3,6,7-tetrahydro oxepin-2-yl)propan-1-ol (precursor of 9)

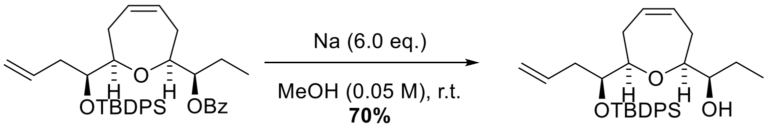

Following the general procedure 3.5, to a solution of oxepene **8** (0.423 g, 0.74 mmol, 1.0 equiv.) in dry MeOH (15 mL, 0.05M) was added sodium (Na, 0.102 g, 4.44 mmol, 6.0 eq.) to obtain 0.242 g of the precursor alcohol of **9** as a colorless oil (0.52 mmol, 70 % yield).

R$_f$ = 0.39 (*n*-hexane/EtOAc 90:10); **[α]$^{25}_D$** = +3.6 (*c* = 1.05, $CHCl_3$); **^{1}H-NMR ($CDCl_3$, 400 MHz)** δ = 7.70 (dt, *J* = 8.1 & 1.4 Hz, 4H), 7.45-7.40 (m, 2H), 7.40-7.35 (m, 4H), 5.80-5.72 (m, 2H), 5.72-5.63 (m, 1H), 4.96-4.87 (m, 2H), 3.77 (m, 1H), 3.46 (m, 1H), 3.33 (brddd, *J* = 10.1, 3.8 & 1.7 Hz, 1H), 3.26 (brddd, *J* = 10.5, 3.2 & 1.7 Hz, 1H), 2.43-2.26 (m, 3H), 2.26-2.14 (m, 2H), 2.09 ((brdd, *J* = 16.4 & 6.3 Hz, 1H), 1.90 (d, *J* = 5.5 Hz, 1H), 1.38 (m, 2H), 1.07 (s, 9H), 0.94 (t, *J* = 7.4 Hz, 3H). **^{13}C-NMR ($CDCl_3$, 100 MHz)** δ = 136.2 (3 x CH), 136.1 (2 x CH), 134.9 (CH), 134.0 (C), 133.9 (C), 129.6 (CH), 129.3 (CH), 129.2 (CH), 127.5 (2 x CH), 127.4 (2 x CH), 116.9 (CH_2), 82.4 (CH), 82.2 (CH), 76.0 (CH), 75.8 (CH), 38.4 (CH_2), 31.8 (CH_2), 30.9 (CH_2), 27.1 (3 x CH_3), 25.0 (CH_2), 19.5 (C), 10.5 (CH_3); **HRMS (ESI$^+$):** *m/z* calcd. for $C_{29}H_{40}O_3SiNa^+$: 487.2644 [*M*+Na]$^+$; found: 487.2641.

(((*S*)-1-((2*R*,7*S*)-7-((*S*)-1-bromopropyl)-2,3,6,7-tetrahydrooxepin-2-yl)but-3-en-1-yl)oxy)(*tert*-butyl)diphenylsilane (9)

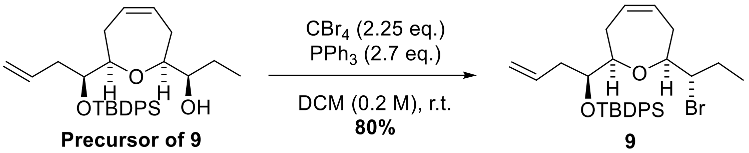

Precursor of 9 **9**

Following the general procedure 3.6, to a solution of the precursor hydroxyoxepene of **9** (0.335 g, 0.72 mmol, 1.0 equiv.) and CBr_4 (0.537 g, 1.62 mmol, 2.25 equiv.) in dry DCM (3.6 mL, 0.2 M) was added PPh_3 (0.509 g, 1.94 mmol, 2.7 equiv.) to give 0.306 g of bromoxepene **9** as a pale yellow oil (0.58 mmol, 80% yield).

R_f = 0.42 (*n*-hexane/EtOAc 98:2); **$[\alpha]^{25}_D$** = -20.4 (*c* = 1.00, $CHCl_3$); **^{1}H-NMR ($CDCl_3$, 400 MHz)** δ = 7.74-7.71 (brdd, *J* = 7.8 & 1.4 Hz, 2H), 7.71-7.67 (brdd, *J* = 7.8 & 1.4 Hz, 2H), 7.43-7.34 (m, 6H), 5.76 (m, 2H), 5.72-5.60 (m, 1H), 4.89 (m, 2H), 3.85 (m, 2H), 3.44 (brddd, *J* = 10.0, 3.2 & 1.5 Hz, 1H), 3.34 (brddd, *J* = 10.1, 3.8 & 1.8 Hz, 1H), 2.52-2.37 (m, 2H), 2.35-2.24 (m, 3H), 2.15 (m, 1H), 1.86 (m, 1H), 1.74 (m, 2H), 1.07 (s, 9H), 1.00 (t, *J* = 7.2 Hz, 3H). **^{13}C-NMR ($CDCl_3$, 100 MHz)** δ = 135.3 (2 x CH), 136.1 (2 x CH), 134.7 (CH), 134.4 (C), 133.9 (C), 129.8 (CH), 129.6 (CH), 129.5 (CH), 128.9 (CH), 127.4 (4 x CH), 117.0 (CH_2), 82.2 (CH), 81.8 (CH), 76.0 (CH), 61.8 (CH), 38.3 (CH_2), 33.6 (CH_2), 31.5 (CH_2), 27.6 (CH_2), 27.1 (3 x CH_3), 19.5 (C), 12.8 (CH_3). **HRMS (ESI$^+$):** *m/z* calcd. for $C_{29}H_{39}BrO_2SiNa^+$: 549.1800 $[M+Na]^+$; found: 549.1806.

(*S*)-1-((2*R*,7*S*)-7-((*S*)-1-bromopropyl)-2,3,6,7-tetrahydrooxepin-2-yl)but-3-en-1-ol (10)

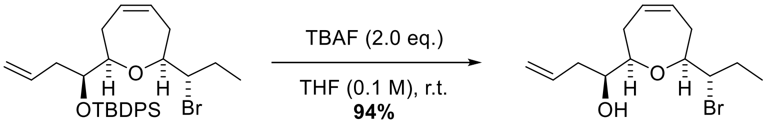

9 **10**

To a solution of bromoxepene **9** (0.100 g, 0.19 mmol, 1.0 equiv.) in dry THF (1.9 mL, 0.1 M) at room temperature was added tetrabutylammonium fluoride (TBAF, 0.38 mmol, 2.0 equiv.). The reaction mixture was stirred and monitored by TLC. Once it was completed, it was quenched with water. The organic phase was separated, and the aqueous layer was extracted with 2 x DCM and 2 x AcOEt. Then, the combined organic layers were dried over $MgSO_4$, filtered and the solvent was removed under reduced pressure. The reaction mixture was purified by flash silica gel column chromatography (*n*-hexane/EtOAc 95:5 solvent system) to obtain 0.052 g of the hydroxyoxepene **10** as a colorless oil (0.18 mmol, 94 % yield).

R_f = 0.37 (*n*-hexane/EtOAc 90:10); **$[\alpha]^{25}_D$** = -13.9 (*c* = 1.00, $CHCl_3$); **^{1}H-NMR ($CDCl_3$, 600 MHz)** δ = 5.92-5.85 (m, 1H), 5.85-5.75 (m, 2H), 5.13 (m, 2H), 3.92 (m, 1H), 3.65 (m, 1H), 3.50 (ddd, *J* = 10.3, 4.4 & 1.4 Hz, 1H), 3.41 (ddd, *J* = 10.0, 4.5 & 2.0 Hz, 1H), 2.49 (m, 1H), 2.41-2.25 (m, 5H), 2.23 (d, *J* = 6.2 Hz, 1H), 1.97 (m, 1H), 1.87 (m, 1H), 1.06 (t, *J* = 7.2 Hz, 3H); **^{13}C-NMR ($CDCl_3$, 100 MHz)** δ = 135.1

(CH), 129.5 (CH), 128.5 (CH), 117.7 (CH_2), 82.9 (CH), 81.7 (CH), 73.3 (CH), 62.2 (CH), 37.1 (CH_2), 34.5 (CH_2), 31.9 (CH_2), 28.4 (CH_2), 12.4 (CH_3); **HRMS (EI$^+$):** *m/z* calcd. for $C_{13}H_{21}BrO_2^+$: 288.0725 [*M*]$^+$; found: 288.0730.

(2*S*,7*R*)-2-((*S*)-1-bromopropyl)-7-((*R*)-1-chlorobut-3-en-1-yl)-2,3,6,7-tetrahydro oxepine (11)

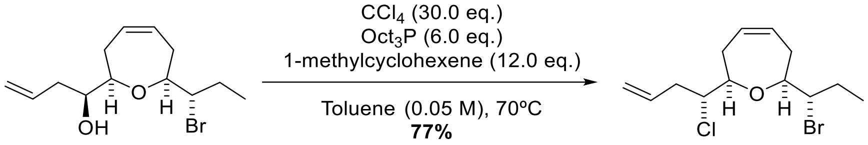

10 11

To a solution of hydroxyoxepene **10** (0.020 g, 0.069 mmol, 1.0 equiv.) in dry toluene (1.4 mL, 0.05M) under inert atmosphere were added CCl_4 (0.20 mL, 2.07 mmol, 30.0 equiv.), tri-*n*-octylphosphine (Oct_3P, 0.20 mL, tech. 90%, 0.41 mmol, 6.0 equiv.) and 1-methylcyclohexene (0.10 mL, 0.83 mmol, 12.0 equiv.) at room temperature. After being stirred at the same temperature for 20 min, the reaction mixture was warmed at 70ºC and was monitored by TLC. Once the reaction was completed, it was allowed to cool to room temperature and concentrated under reduced pressure.[18] The reaction crude was purified by flash silica gel column chromatography (*n*-hexane/EtOAc 98:2 solvent system) to afford 0.016 g of the halogenated oxepene **11** as a colorless oil (0.053 mmol, 77 % yield).

R_f = 0.65 (*n*-hexane/EtOAc 85:15); **$[\alpha]^{25}_D$** = +0.39 (*c* = 1.02, $CHCl_3$); **^{1}H-NMR ($CDCl_3$, 400 MHz)** δ = 5.92-5.83 (m, 1H), 5.83-5.78 (m, 2H), 5.14 (m, 2H), 3.91 (m, 2H), 3.60 (ddd, *J* = 10.3, 3.5 &1.6 Hz, 1H), 3.52 (ddd, *J* = 10.2, 3.7 & 1.6 Hz, 1H), 2.72 (m, 1H), 2.59-2.47 (m, 3H), 2.33 (m, 2H), 2.02 (m, 1H), 1.86 (m, 1H), 1.06 (t, *J* = 7.2 Hz, 3H); **^{13}C-NMR ($CDCl_3$, 100 MHz)** δ = 134.6 (CH), 129.1 (CH), 129.0 (CH), 117.8 (CH_2), 81.9 (CH), 81.2 (CH), 64.2 (CH), 61.3 (CH), 38.5 (CH_2), 33.7 (CH_2), 33.0 (CH_2), 28.0 (CH_2), 12.7 (CH_3); **HRMS (EI$^+$):** *m/z* calcd. for $C_{13}H_{20}BrClO^+$: 306.0386 [*M*]$^+$; found: 306.0387.

(*R*,*E*)-5-((2*R*,7*S*)-7-((*S*)-1-bromopropyl)-2,3,6,7-tetrahydrooxepin-2-yl)-5-chloro pent-2-enal (13)

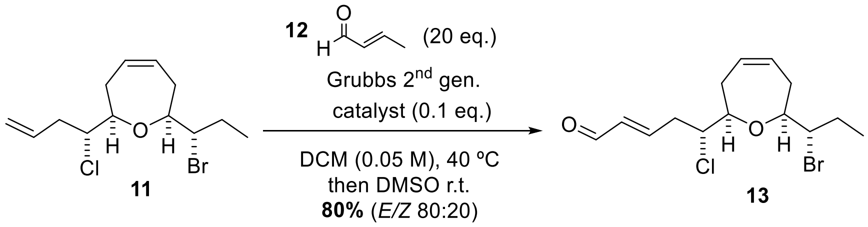

11 13

To a solution of oxepene **11** (0.041 g, 0.13 mmol, 1.0 equiv.) in dry DCM (2.6 mL, 0.05 M) were added crotonaldehyde (**12**) (0.22 mL, 2.6 mmol, 20 equiv.) and Grubbs 2nd generation catalyst ([(H_2IMes)(PCy_3)(Cl_2)Ru=CHPh], 0.011 g, 0.013 mmol, 0.1 equiv.) at room temperature under inert atmosphere. The reaction mixture was heated to 40ºC and refluxed for 1 h (Heat-On™ Block System). Then, DMSO (0.10 mL) was added and the solution was stirred open to air at room temperature overnight.[19] The reaction mixture was concentrated under reduced pressure and it was purified by flash silica gel column chromatography (*n*-hexane/EtOAc 90:10 solvent system) to give 0.028 g of the enal-oxepene **13** as a pale yellow oil (0.083 mmol, 64 % yield).

R_f = 0.46 (*n*-hexane/EtOAc 90:10); **^{1}H-NMR ($CDCl_3$, 500 MHz)** δ = 9.56 (d, *J* = 7.8 Hz, 1H), 6.90 (dt, *J* = 15.7 & 7.0 Hz, 1H), 6.21 (dd, *J* = 15.7 & 7.8 Hz, 1H), 5.83 (m, 2H), 4.03 (dt, *J* = 9.4 & 4.0 Hz, 1H), 3.91 (dt, *J* = 9.3 & 4.2 Hz, 1H), 3.65 (ddd, *J* = 10.1, 3.4 & 1.4 Hz, 1H), 3.53 (ddd, *J* = 10.3, 4.0 & 1.2 Hz, 1H), 2.96 (m, 1H), 2.81 (m, 1H), 2.54 (m, 2H), 2.36 (m, 2H), 1.97 (m, 1H), 1.85 (m, 1H), 1.06 (t, *J* = 7.3 Hz, 3H). **^{13}C-NMR ($CDCl_3$, 125 MHz)** δ = 193.6 (CH), 153.5 (CH), 134.9 (CH), 129.3 (CH), 128.7 (CH), 82.0 (CH), 81.4 (CH), 62.0 (CH), 61.3 (CH), 36.9 (CH_2), 34.0 (CH_2), 32.0 (CH_2), 28.2 (CH_2), 12.5 (CH_3). **HRMS (EI^+):** *m/z* calcd. for $C_{14}H_{20}BrClNaO_2^+$: 357.0233 [*M*]$^+$; found: 357.0231.

(+)-isolaurepinnacin (1)

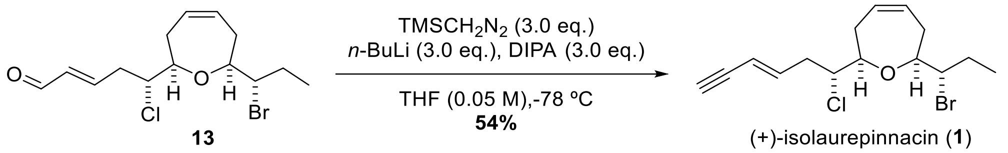

To a solution of diisopropylamine (0.028 mL, 0.20 mmol, 3.0 equiv.) in dry THF (0.4 mL, 0.5 M) at -78ºC under inert atmosphere, was added *n*-BuLi (0.080 mL, 2.5 M solution in hexanes, 0.20 mmol, 3.0 equiv.) and stirred for 1 h at this temperature to generate LDA *in situ*. Then, $TMSCHN_2$ (0.10 mL, 2.0 M solution in Et_2O, 0.20 mmol, 3.0 equiv.) was added dropwise at -78ºC and stirred for 30 min. Then, enal-oxepene **13** (0.022 g, 0.066 mmol, 1.0 equiv.) in dry THF (1.3 mL, 0.05 M) was added dropwise at -78ºC. The reaction mixture was stirred at this temperature and monitored by TLC. It was quenched with saturated aqueous NH_4Cl solution and the layers were separated. The aqueous phase was extracted with 2 x Et_2O. The combined organic phases were washed with saturated brine, dried over $MgSO_4$ and the solvent was removed under reduced pressure.[19] The reaction mixture was purified by flash silica gel column chromatography (*n*-hexane/EtOAc 95:5 solvent system) to afford 0.012 g of the (+)-isolaurepinnacin (**1**) as colorless oil (0.036 mmol, 54% yield). Spectral data was consistent with the literature.[20,21]

R_f = 0.70 (*n*-hexane/EtOAc 90:10); **$[\alpha]^{25}_D$** = + 0.67 (*c* = 0.30, $CHCl_3$). Literature value: $[\alpha]^{23}_D$ = +0.6 (*c* = 1.4, $CHCl_3$);[21] **^{1}H-NMR ($CDCl_3$, 500 MHz)** δ = 6.27 (dt, *J* = 14.7 & 7.2 Hz, 1H), 5.82 (brt, *J* = 4.1 Hz, 2H), 5.59 (ddt, *J* = 15.9, 3.7 & 1.5 Hz, 1H), 3.92 (dt, *J* = 8.0 & 3.9 Hz, 1H), 3.89 (dt, *J* = 4.5 & 3.4 Hz, 1H), 3.59 (ddd, *J* = 10.2, 3.3 & 1.5 Hz, 1H), 3.53 (ddd, *J* = 10.2, 3.9 & 1.5 Hz, 1H), 2.85 (d, *J* = 2.0 Hz, 1H), 2.77 (dddd, *J* = 14.9, 6.5, 4.7 & 1.3 Hz, 1H), 2.56 (m, 1H), 2.55 (m, 2H), 2.36 (m, 1H), 2.30 (m, 1H), 1.99 (ddq, *J* = 14.6, 7.3 & 3.3 Hz, 1H), 1.85 (ddq, *J* = 14.6, 7.3 & 2.2 Hz, 1H), 1.06 (t, *J* = 7.2 Hz, 3H); **^{13}C-NMR ($CDCl_3$, 100 MHz)** δ = 141.8 (CH), 129.1 (CH), 128.9 (CH), 111.7 (CH), 81.9 (CH), 81.8 (C), 81.0 (CH), 76.9 (CH), 63.3 (CH), 61.4 (CH), 37.5 (CH_2), 33.7 (CH_2), 32.7 (CH_2), 28.0 (CH_2), 12.7 (CH_3); **HRMS (ESI^+):** *m/z* calcd. for $C_{15}H_{20}BrClONa^+$: 353.0284 [*M*+Na]$^+$; found: 353.0279.

(*R*)-1-((2*R*,7*S*)-7-((*R*)-1-((*tert*-butyldiphenylsilyl)oxy)but-3-en-1-yl)-2,3,6,7-tetrahydrooxepin-2-yl)propyl benzoate (14)

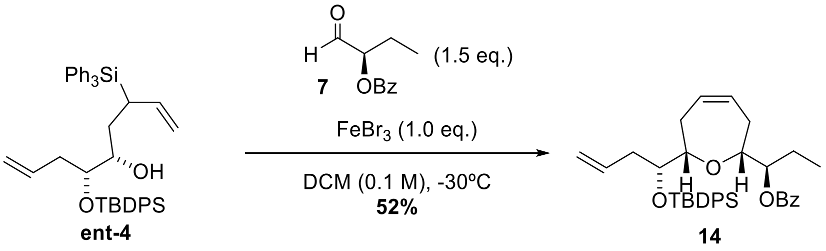

Following the general procedure 3.4, to a solution of *bis*-homoallylsilyl alcohol **ent-4** (1.19 g, 1.82 mmol, 1.0 equiv) in 18.2 mL of dry DCM (0.1 M) at -30ºC were added the aldehyde **7** (0.525 g, 2.73 mmol, 1.5 equiv.) and the $FeBr_3$ (0.549 g, 1.82 mmol, 1.0 equiv to give 0.540 g of oxepene **14** as a colorless oil (0.95 mmol, 52% yield).

$\mathbf{R_f}$ = 0.49 (*n*-hexane/EtOAc 90:10); $[\alpha]^{25}_D$ = +27.2 (*c* = 1.10, $CHCl_3$); **^{1}H-NMR ($CDCl_3$, 600 MHz)** δ = 8.06 (dd, *J* = 8.3 & 1.2 Hz, 2H), 7.75 (dd, *J* = 8.0 & 1.4 Hz, 2H), 7.69 (dd, *J* = 8.1 & 1.4 Hz, 2H), 7.55 (m, 1H), 7.44-7.38 (m, 3H), 7.38-7.33 (m, 4H), 5.75 (m, 2H), 5.63 (m, 1H), 5.06 (m, 1H), 4.91 (d, *J* = 10.3 Hz, 1H), 4.86 (dd, *J* = 17.1 & 1.6 Hz, 1H), 3.88 (m, 1H), 3.52 (ddd, *J* = 10.5, 3.7 & 1.5 Hz, 1H), 3.3 (ddd, *J* = 10.1, 3.6 & 1.6 Hz, 1H), 2.47-2.35 (m, 2H), 2.35-2.28 (m, 1H), 2.24-2.16 (m, 2H), 2.15-2.09 (m, 1H), 1.74 (m, 2H), 1.08 (s, 9H), 0.90 (t, *J* = 7.4 Hz, 3H); **^{13}C-NMR ($CDCl_3$, 150 MHz)** δ = 166.4 (C), 136.2 (2 x CH), 136.0 (2 x CH), 134.6 (CH), 133.8 (2 x C), 132.8 (CH), 130.5 (C), 129.8 (CH), 129.7 (2 x CH), 129.5 (CH), 129.0 (CH), 128.3 (2 x CH), 127.4 (2 x CH), 127.4 (2 x CH), 117.1 (CH_2), 82.2 (CH), 79.5 (CH), 78.0 (CH), 77.2 (CH), 76.0 (CH), 38.4 (CH_2), 32.8 (CH_2), 31.5 (CH_2), 27.1 (3 x CH_3), 23.0 (CH_2), 19.6 (C), 10.2 (CH_3); **HRMS (ESI$^+$):** *m/z* calcd. for $C_{36}H_{44}O_4SiNa^+$: 591.2907 $[M+Na]^+$; found: 591.2916.

(*R*)-1-((2*R*,7*S*)-7-((*R*)-1-((*tert*-butyldiphenylsilyl)oxy)but-3-en-1-yl)-2,3,6,7-tetrahydrooxepin-2-yl)propan-1-ol (precursor of 15)

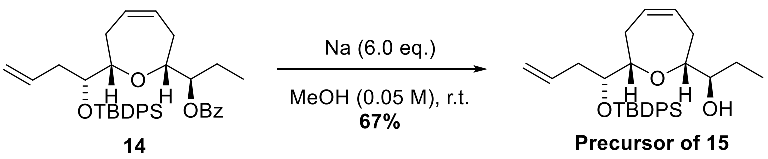

Following the general procedure 3.5, to a solution of oxepene **14** (0.455 g, 0.80 mmol, 1.0 equiv.) in dry MeOH (16 mL, 0.05M) was added sodium (Na, 0.110 g, 4.80 mmol, 6.0 eq.) to obtain 0.251 g of the precursor hydroxyoxepene of **15** as a colorless oil (0.54 mmol, 67 % yield).

$\mathbf{R_f}$ = 0.30 (*n*-hexane/EtOAc 90:10); $[\alpha]^{25}_D$ = +6.2 (*c* = 0.76, $CHCl_3$); **^{1}H-NMR ($CDCl_3$, 500 MHz)** δ = 7.71 (dd, *J* = 7.9 & 1.3 Hz, 2H), 7.67 (dd, *J* = 7.9 & 1.2 Hz, 2H), 7.43 (m, 2H), 7.37 (m, 4H), 5.72 (m, 2H), 5.64 (m, 1H), 4.93 (d, *J* = 1.2 Hz, 1H), 4.90 (dd, *J* = 9.8 & 1.5 Hz, 1H), 3.75 (ddd, *J* = 6.3, 6.3 & 3.4 Hz, 1H), 3.36 (ddd, *J* = 10.8, 2.8 & 1.8 Hz, 1H), 3.28 (m, 1H), 3.13 (ddd, *J* = 10.3, 6.4 & 1.7 Hz, 1H),

2.56 (brs, 1H), 2.41 (m, 1H), 2.33-2.12 (m, 5H), 1.50 (m, 1H), 1.37 (m, 1H), 1.07 (s, 9H), 0.95 (t, *J* = 7.4 Hz, 3H); **^{13}C-NMR ($CDCl_3$, 100 MHz)** δ = 136.1 (2 x CH), 136.0 (2 x CH), 134.7 (CH), 134.0 (C), 133.8 (C), 129.7 (CH), 129.6 (CH), 129.2 (CH), 128.6 (CH), 127.5 (4 x CH), 117.0 (CH_2), 82.5 (CH), 82.3 (CH), 75.8 (CH), 75.3 (CH), 38.2 (CH_2), 33.5 (CH_2), 31.6 (CH_2), 27.1 (3 x CH_3), 26.1 (CH_2), 19.4 (C), 9.8 (CH_3); **HRMS (ESI$^+$):** *m/z* calcd. for $C_{29}H_{40}O_3SiNa^+$: 487.2644 [*M*+Na]$^+$; found: 487.2641.

(((*R*)-1-((2*S*,7*R*)-7-((*S*)-1-bromopropyl)-2,3,6,7-tetrahydrooxepin-2-yl)but-3-en-1-yl)oxy)(*tert*-butyl)diphenylsilane (15)

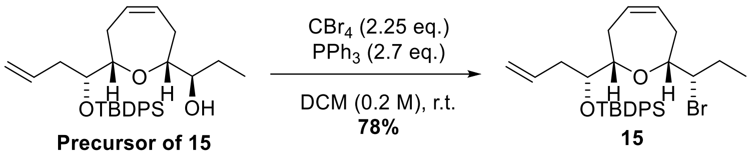

Following the general procedure 3.6, to a solution of the precursor hydroxyoxepene of **15** (0.500 g, 1.08 mmol, 1.0 equiv.) and carbon tetrabromide (0.806 g, 2.43 mmol, 2.25 equiv.) in 5.4 mL of dry DCM (0.2 M) was added PPh_3 (0.587 g, 2.92 mmol, 2.7 equiv.) to afford 0.443 g of the bromoxepene **15** as a pale yellow oil (0.84 mmol, 78 % yield).

R_f = 0.53 (*n*-hexane/EtOAc 98:2); **$[\alpha]^{25}_D$** = -9.9 (c = 1.03, $CHCl_3$); **^{1}H-NMR ($CDCl_3$, 500 MHz)** δ = 7.72 (dd, *J* = 7.9 & 1.4 Hz, 2H), 7.69 (dd, *J* = 7.9 & 1.4 Hz, 2H), 7.45-7.33 (m, 6H). 5.79-5.70 (m, 2H), 5.70-5.60 (m, 1H), 4.91 (m, 2H), 3.84 (m, 2H), 3.39 (m, 2H), 2.50 (m, 1H), 2.34 (m, 4H), 2.17 (m, 1H), 1.90 (m, 1H), 1.68 (m, 1H), 1.06 (s, 9H), 0.98 (t, *J* = 7.2 Hz, 3H); **^{13}C-NMR ($CDCl_3$, 100 MHz)** δ = 136.2 (2 x CH), 136.1 (2 x CH), 134.7 (CH), 134.3 (C), 133.8 (C), 129.7 (CH), 129.6 (CH), 129.5 (CH), 128.3 (CH), 127.4 (2 x CH), 127.3 (2 x CH), 117.0 (CH_2), 82.3 (CH), 82.1 (CH), 75.8 (CH), 62.2 (CH), 38.3 (CH_2), 34.3 (CH_2), 31.6 (CH_2), 27.8 (CH_2), 27.1 (3 x CH_3), 19.5 (C), 12.2 (CH_3); **HRMS (ESI$^+$):** m/z calcd. for $C_{29}H_{39}BrO_2SiNa^+$: 549.1800 [*M*+Na]$^+$; found: 549.1807.

(*R*)-1-((2*S*,7*R*)-7-((*S*)-1-bromopropyl)-2,3,6,7-tetrahydrooxepin-2-yl)but-3-en-1-ol (16)

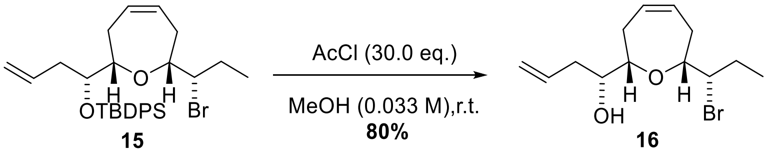

To a solution of acetyl chloride (AcCl) (0.41 mL, 5.70 mmol, 30.0 equiv.) in 5.8 mL of dry MeOH (0.033 M *vs* oxepene **15**) at room temperature was added bromoxepene **15** (0.100 g, 0.19 mmol, 1.0 equiv.) dissolved in 4.8 mL of dry Et_2O (0.04 M). The reaction was stirred for 4 days. Then, the solvent was removed under reduced pressure and the reaction crude was purified by flash silica gel column chromatography (*n*-hexane/EtOAc 90:10 solvent system) to obtain 43 mg of the hydroxyoxepene **16** as a colorless oil (0.15 mmol, 80 % yield).

R_f = 0.36 (*n*-hexane/EtOAc 90:10); **$[\alpha]^{25}_D$** = -25.7 (*c* = 1.00, $CHCl_3$); **^{1}H-NMR ($CDCl_3$, 500 MHz)** δ = 5.94-5.85 (m, 1H), 5.84-5.74 (m, 2H), 5.14 (m, 2H), 3.95 (ddd, *J* = 9.6, 4.7 & 3.2 Hz, 1H), 3.64 (m, 2H),

3.43 (ddd, J = 9.7, 4.4 & 2.4 Hz, 1H), 2.44 (m, 2H), 2.41-2.33 (m, 2H), 2.33-2.24 (m, 2H), 2.22 (m, 1H), 1.95 (m, 1H), 1.86 (m, 1H), 1.07 (t, J = 7.3 Hz, 3H); **^{13}C-NMR ($CDCl_3$, 125 MHz)** δ = 135.1 (CH), 129.5 (CH), 128.5 (CH), 117.7 (CH_2), 82.8 (CH), 82.7 (CH), 73.3 (CH), 62.2 (CH), 37.1 (CH_2), 34.3 (CH_2), 32.0 (CH_2), 26.9 (CH_2), 12.4 (CH_3); **HRMS (ESI$^+$):** *m/z* calcd. for $C_{13}H_{21}BrO_2Na^+$: 311.0623 [M+Na]$^+$; found: 311.0619.

(*S*)-1-((2*S*,7*R*)-7-((*S*)-1-bromopropyl)-2,3,6,7-tetrahydrooxepin-2-yl)but-3-en-1-yl 4-nitrobenzoate (precursor of 17)

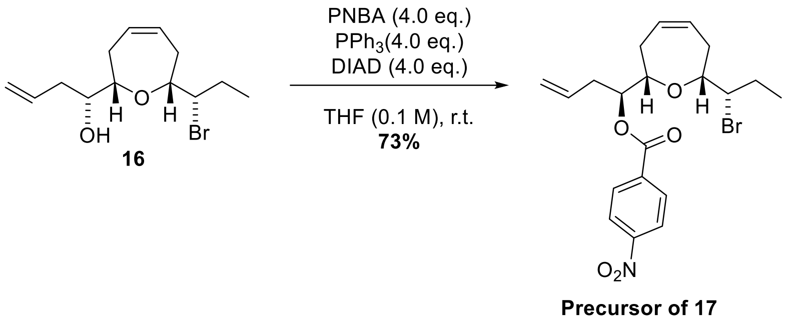

To a solution of hydroxyoxepene **16** (0.132 g, 0.46 mmol, 1.0 equiv.) in 4.6 mL of dry THF (0.1 M) at room temperature under inert atmosphere were added *p*-NO_2-benzoic acid (0.308 g, 1.84 mmol, 4.0 equiv.), PPh_3 (0.483 g, 1.84 mmol, 4.0 equiv.) and DIAD (0.36 mL, 1.84 mmol, 4.0 equiv.). The reaction mixture was stirred overnight at room temperature. The solvent was removed under reduced pressure and *n*-hexane/EtOAc (85:15) mixture was added. The solid of triphenylphosphine oxide was filtered through a pad of celite. The organic phase was concentrated, and the crude reaction was purified by flash silica gel column chromatography (*n*-hexane/EtOAc 90:10 solvent system) to give 0.149 g of the precursor oxepene of **17** as a yellow oil (0.34 mmol, 73 % yield).

R_f = 0.59 (*n*-hexane/EtOAc 90:10); **$[\alpha]^{25}_D$** = -62.6 (c = 1.00, $CHCl_3$); **^{1}H-NMR ($CDCl_3$, 500 MHz)** δ = 8.29 (m, 2H), 8.22 (m, 2H), 5.80 (m, 3H), 5.24 (dt, J = 8.0 & 4.7 Hz, 1H), 5.14 (dq, J = 17.1 & 1.5 Hz, 1H), 5.07 (dd, J = 10.2 & 0.8 Hz, 1H), 3.89 (ddd, J = 8.9, 5.4 & 3.3 Hz, 1H), 3.68 (ddd, J = 10.4, 4.6 & 1.9 Hz, 1H), 3.59 (ddd, J = 9.6, 5.4 & 2.0 Hz, 1H), 2.65 (m, 1H), 2.58-2.48 (m, 2H), 2.48-2.36 (m, 2H), 2.28 (m, 1H), 1.95 (m, 1H), 1.80 (m, 1H), 0.98 (t, J = 7.3 Hz, 3H); **^{13}C-NMR ($CDCl_3$, 125 MHz)** δ = 164.3 (C), 150.5 (C), 135.8 (C), 133.3 (CH), 130.8 (2 x CH), 129.0 (CH), 128.7 (CH), 123.5 (2 x CH), 118.4 (CH_2), 82.9 (CH), 79.6 (CH), 76.7 (CH), 61.5 (CH), 35.2 (CH_2), 34.4 (CH_2), 33.0 (CH_2), 27.2 (CH_2), 12.3 (CH_3); **HRMS (ESI$^+$):** *m/z* calcd. for $C_{20}H_{24}BrNO_5Na^+$: 460.0736 [M+Na]$^+$; found: 460.0733.

(*S*)-1-((2*S*,7*R*)-7-((*S*)-1-bromopropyl)-2,3,6,7-tetrahydrooxepin-2-yl)but-3-en-1-ol (17)

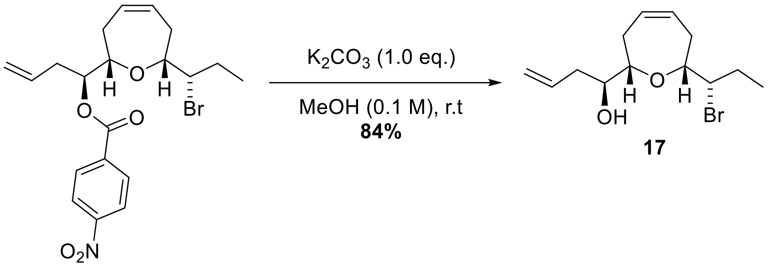

To a solution of the precursor oxepene of **17** (0.284 g, 0.65 mmol, 1.0 equiv.) in 6.5 mL of dry MeOH (0.1 M) was added K_2CO_3 (0.090 g, 0.65 mmol, 1.0 equiv.) at room temperature under inert atmosphere. Once the reaction was completed, 2 drops of water were added. The solvent was removed under reduced pressure and the reaction residue was purified by automated flash chromatography Biotage (DCM 100% solvent system) to give 0.159 g of hydroxyoxepene **17** as a colorless oil (0.55 mmol, 84% yield).

R_f = 0.53 (*n*-hexane/EtOAc 95:5); **$[\alpha]^{25}_D$** = -65.4 (*c* = 0.73, $CHCl_3$); **^{1}H-NMR ($CDCl_3$, 500 MHz)** δ = 5.91 (m, 1H), 5.77 (m, 2H), 5.12 (m, 2H), 3.97 (dt, *J* = 10.0 & 3.7 Hz, 1H), 3.68 (ddd, *J* = 10.2, 4.0 & 1.8 Hz, 1H), 3.57 (m, 1H), 3.32 (ddd, *J* = 10.5, 6.3 & 2.1 Hz, 1H), 2.93 (d, *J* = 3.5 Hz, 1H), 2.47 (m, 1H), 2.37 (m, 3H), 2.25 (m, 2H), 1.90 (m, 2H), 1.07 (t, *J* = 7.3 Hz, 3H); **^{13}C-NMR ($CDCl_3$, 125 MHz)** δ = 134.7 (CH), 129.1 (CH), 128.3 (CH), 117.2 (CH_2). 82.9 (CH), 82.8 (CH), 73.9 (CH), 62.3 (CH), 37.8 (CH_2), 34.2 (CH_2), 33.0 (CH_2), 26.6 (CH_2), 12.6 (CH_3); **HRMS (ESI$^+$):** *m/z* calcd. for $C_{13}H_{21}BrO_2Na^+$: 311.0623 $[M+Na]^+$; found: 311.0623.

(1*S*,3*R*,5*S*,6*S*,8*S*)-8-allyl-5-bromo-3-((*S*)-1-bromopropyl)-2,7-dioxabicyclo[4.2.1] nonane (18)

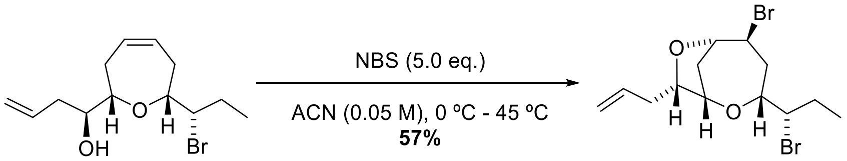

To a solution of hydroxyoxepene **17** (38 mg, 0.13 mmol, 1.0 equiv.). in 2.6 mL of dry acetonitrile (0.05 M) at 0ºC under inert atmosphere, was added NBS (0.112 g, 0.65 mmol, 5.0 equiv.). Then, the ice bath was removed, and the reaction mixture was heated to 45ºC. Once the reaction was complete, it was cooled to room temperature and the solvent was removed under reduced pressure.[22] The residue was purified by flash column chromatography (*n*-hexane/EtOAc 90:10 solvent system) to afford 26.5 mg of bicyclic oxepene **18** (0.072 mmol, 57% yield).

R_f = 0.49 (*n*-hexane/EtOAc 90:10); **$[\alpha]^{25}_D$** = +5.2 (*c* = 0.50, $CHCl_3$); **^{1}H-NMR ($CDCl_3$, 500 MHz)** δ = 5.85 (m, 1H), 5.19 (m, 1H), 5.08 (m, 1H), 4.49 (dd, *J* = 8.4 & 4.6 Hz, 1H), 4.34 (brt, *J* = 3.5 Hz, 1H), 4.29 (m, 1H), 4.08 (m, 1H), 3.92 (dd, *J* = 8.9 & 3.3 Hz, 1H), 3.86 (dt, *J* = 7.2 & 2.7 Hz, 1H), 2.76 (d, *J* = 15.1 Hz, 1H), 2.58 (m, 1H), 2.44 (m, 2H), 2.18-2.13 (m, 1H), 2.13-2.07 (m, 1H), 1.87 (m, 2H), 1.09 (t, *J* = 7.3 Hz, 3H); **^{13}C-NMR ($CDCl_3$, 125 MHz)** δ = 134.5 (CH), 117.3 (CH_2), 86.4 (CH), 80.4 (CH), 75.3

(CH), 71.9 (CH), 62.5 (CH), 51.1 (CH), 36.1 (CH_2), 33.6 (CH_2), 30.3 (CH_2), 28.5 (CH_2), 12.5 (CH_3); **HRMS (ESI$^+$):** *m/z* calcd. for $C_{13}H_{20}Br_2O_2Na^+$: 388.9728 [*M*+Na]$^+$; found: 388.9714.

3-(triisopropylsilyl)prop-2-yn-1-ol (C)

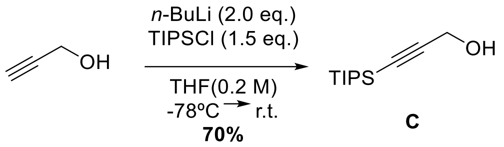

To a solution of propargyl alcohol (2.0 g, 35.7 mmol, 1.0 equiv.) in 179 mL of dry THF (0.2 M) at -78°C under an inert atmosphere, *n*-BuLi 2.5 M in hexanes (28.6 mL, 71.4 mmol, 2.0 equiv.) was added dropwise. After 20 min at -78°C, TIPSCl (11.5 mL, 53.6 mmol, 1.5 equiv.) was added dropwise. The solution was stirred 5 minutes at -78°C and then the bath was removed. It was left to react for 1 hour. The reaction was quenched with water and then with an aqueous solution of 10% HCl, until the complete disappearance of the silylated ether was observed. The reaction mixture was extracted with Et_2O.[23] The organic phase was washed with saturated NaCl, dried over $MgSO_4$, filtered, and concentrated under vacuum. It was purified by column chromatography (*n*-hexane/EtOAc 90:10 solvent system) to give 5.31g of silylated propargyl alcohol **C** as a yellow oil (25.0 mmol, 70% of yield). Spectral data was consistent with the literature. [23,24]

R_f = 0.42 (*n*-hexane/EtOAc 90:10); **^{1}H-NMR ($CDCl_3$, 400 MHz)** δ = 4.30 (d, *J* = 6.2 Hz, 2H), 1.53 (dt, *J* = 6.2 & 1.0 Hz, 1H), 1.11-1.02 (m, 21H); **^{13}C-NMR ($CDCl_3$, 100 MHz)** δ = 105.7 (C), 86.7 (C), 51.7 (CH_2), 18.5 (6 x CH_3), 11.1 (3 x CH); **HRMS (ESI$^+$):** *m/z* calcd. for $C_{12}H_{24}OsiNa^+$: 235.1494 [*M*+Na]$^+$; found: 235.1496.

Ethyl (*E*)-5-(triisopropylsilyl)pent-2-en-4-ynoate (D)

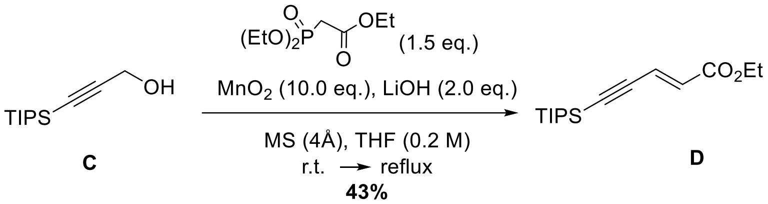

To a solution of silylated propargyl alcohol **C** (2.0 g, 9.42 mmol, 1.0 equiv.) in 47 mL of dry THF were added MnO_2 (8.2 g, 94.2 mmol, 10.0 equiv.), LiOH (0.789 g, 18.8 mmol, 2.0 equiv.), 4Å molecular sieves (4.7 g) and ethyl 2-(diethoxyphosphoryl)acetate (2.80 mL, 14.1 mmol, 1.5 equiv.) at room temperature. Then, the reaction mixture was heated at reflux (Heat-On™ Block System). Once the reaction was complete, it was cooled to room temperature and filter through a pad of celite and wash with EtOAc. The organic phase was neutralized with aq. sat. NH_4Cl solution and extracted with Et_2O. The combined organic layers were dried over $MgSO_4$ and concentrated under reduced pressure.[25] The reaction crude was purified by column chromatography (*n*-hexane/EtOAc 90:10 solvent system) to afford 1.14 g of ester **D** as a yellow oil (4.05 mmol, 43% yield). Spectral data was consistent with the literature.[26]

R_f = 0.69 (*n*-hexane/EtOAc 90:10); **^{1}H-NMR ($CDCl_3$, 400 MHz)** δ = 6.78 (d, *J* = 16.0 Hz, 1H), 6.26 (d, *J* = 16.1 Hz, 1H), 4.21 (q, *J* = 7.2 Hz, 2H), 1.29 (t, *J* = 7.1 Hz, 3H), 1.10-1.06 (m, 21H); **^{13}C-NMR ($CDCl_3$, 100 MHz)** δ = 165.9 (C), 131.1 (CH), 125.1 (CH), 103.3 (C), 101.8 (C), 60.7 (CH_2), 18.5 (6 x CH_3), 14.2 (CH_3), 11.1 (3 x CH); **HRMS (ESI$^+$):** *m/z* calcd. for $C_{16}H_{28}O_2SiNa^+$: 303.1756 [*M*+Na]$^+$; found: 303.1759.

(*E*)-5-(triisopropylsilyl)pent-2-en-4-yn-1-ol (E)

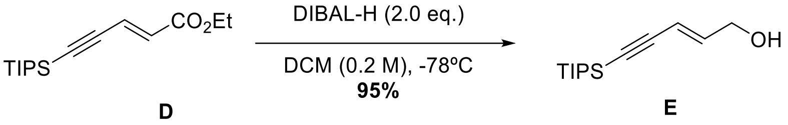

To a solution of ester **D** (1.50 g, 5.35 mmol, 1.0 equiv.) in 26.8 mL of dry DCM (0.2 M) at -78ºC was added DIBAL-H (1.0 M in hexane, 10.7 mL, 10.7 mmol, 2.0 equiv.) dropwise. The reaction mixture was quenched with a 1 M aqueous solution of HCl and then allowed to warm to room temperature. The layers were separated and aqueous phase was extracted with 3 x DCM. The combined organic layers were dried over $MgSO_4$, filtered and concentrated under reduced pressure. The reaction crude was purified by column chromatography (*n*-hexane/EtOAc 90:10 solvent system) to obtain 1.21 g of alcohol **E** as a yellow oil (5.08 mmol, 95% yield). Spectral data was consistent with the literature.[27]

R_f = 0.35 (*n*-hexane/EtOAc 90:10); **^{1}H-NMR ($CDCl_3$, 500 MHz)** δ = 6.31 (dt, *J* = 15.0 & 5.2 Hz, 1H), 5.80 (dt, *J* = 16.0 & 1.8 Hz, 1H), 4.21 (dt, *J* = 5.8 & 1.8 Hz, 2H), 1.10-1.03 (m, 21H); **^{13}C-NMR ($CDCl_3$, 125 MHz)** δ = 142.5 (CH), 110.9 (CH), 104.8 (C), 91.6 (C), 62.9 (CH_2), 18.6 (6 x CH_3), 12.3 (3 x CH); **HRMS (ESI$^+$):** *m/z* calcd. for $C_{14}H_{26}OsiNa^+$: 261.1651 [*M*+Na]$^+$; found: 261.1656.

(*E*)-(5-(allyloxy)pent-3-en-1-yn-1-yl)triisopropylsilane (19)

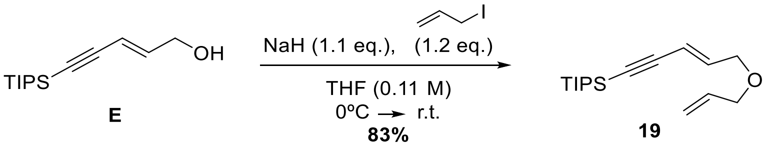

To a solution of alcohol **E** (0.300 g, 1.26 mmol, 1.0 equiv.) in mL 11.5 of dry THF (0.11 M) at 0ºC under inert atmosphere were added NaH 60% (0.056 g, 1.39 mmol, 1.1 equiv.) and allyl iodide (0.14 mL, 1.51 mmol, 1.2 equiv.). The ice bath was removed, and the reaction mixture was stirred at room temperature. Once the reaction was complete, it was quenched with saturated aqueous NH_4Cl. The aqueous layer was extracted with 3 x Et_2O. The combined organic extracts were washed with brine and then with saturated aqueous $NaHCO_3$. The organic layers were dried over $MgSO_4$, filtered and concentrated under reduced pressure. The reaction crude was purified by column chromatography (*n*-hexane/EtOAc 95:5 solvent system) to obtain 0.292 g of ether **19** as a yellow oil (1.05 mmol, 83% yield).

R_f = 0.35 (*n*-hexane/EtOAc 90:10); **^{1}H-NMR ($CDCl_3$, 500 MHz)** δ = 6.23 (dt, *J* = 16.0 & 5.5 Hz, 1H), 5.90 (ddt, *J* = 16.0, 10.4, 5.6 Hz, 1H), 5.79 (dt, *J* = 15.9 & 1.8 Hz, 1H), 5.29 (dq, *J* = 17.2 & 1.6 Hz, 1H), 5.19 (dq, *J* = 10.4 & 1.4 Hz, 1H), 4.03 (dd, *J* = 5.5 & 1.8 Hz, 2H), 3.99 (dt, *J* = 5.6 & 1.4 Hz, 2H), 1.07 (m, 21H); **^{13}C-NMR ($CDCl_3$, 125 MHz)** δ = 140.2 (CH), 134.6 (CH), 117.2 (CH_2), 112.0 (CH), 104.9

(C), 91.4 (C), 71.3 (CH_2), 69.7 (CH_2), 18.6 (6 x CH_3), 11.3 (3 x CH); **HRMS (ESI^+):** *m/z* calcd. for $C_{17}H_{30}OSiNa^+$: 301.1964 [*M*+Na]$^+$; found: 301.1965.

((*Z*)-5-((1*S*,3*R*,5*S*,6*S*,8*S*)-5-bromo-3-((*S*)-1-bromopropyl)-2,7-dioxabicyclo[4.2.1]nonan-8-yl)pent-3-en-1-yn-1-yl)triisopropyl silane (20)

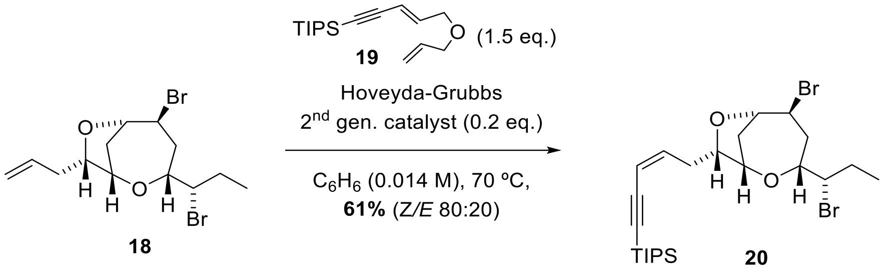

To a solution of bicyclic oxepene **18** (10 mg, 0.027 mmol, 1.0 equiv.) in 1.9 mL of dry benzene (0.014 M) were added TIPS-enyne **19** (11 mg, 0.041 mmol, 1.5 equiv.) in benzene (0.2 mL, 0.2 M) and Hoveyda-Grubbs 2nd catalyst (3.5 mg, $5.6\cdot10^{-3}$ mmol, 0.2 equiv.) in benzene (0.3 mL, 0.016 M) at room temperature under nitrogen atmosphere. The reaction mixture was stirred at 70°C until it was complete. DMSO (3 drops) was added to the solution, and it was stirred open to the air for 12 h. The reaction mixture was concentrated under reduced pressure.[13] The reaction crude was purified by column chromatography (*n*-hexane/EtOAc 98:2 solvent system) to give 8.9 mg of a mixture 80:20 of *Z*/*E*-TIPS-enyne oxepene **20** as a colorless oil (0.0164 mmol, 61% yield).

R_f = 0.68 (*n*-hexane/EtOAc 90:10); **$[\alpha]^{25}_D$** = +20.8 (*c* = 0.39, $CHCl_3$); **^{1}H-NMR ($CDCl_3$, 500 MHz)** δ = 6.05 (m, 1H), 5.64 (d, *J* = 10.9 Hz, 1H), 4.49 (dd, *J* = 8.4 & 4.5 Hz, 1H), 4.34 (brt, *J* = 3.5 Hz, 1H), 4.29 (q, *J* = 3.6 Hz, 1H), 4.03 (dt, *J* = 9.0 & 4.5 Hz, 1H), 3.93 (m, 2H), 2.85 (dt, *J* = 14.0 & 8.0 Hz, 1H), 2.77 (d, *J* = 15.0 Hz, 1H), 2.67 (m, 1H), 2.43 (ddd, *J* = 16.1, 9.3 & 3.6 Hz, 1H), 2.21 (dd, *J* = 16.1 & 2.5 Hz, 1H), 2.09 (ddd, *J* = 14.0, 8.5 & 4.3 Hz, 1H), 1.88 (m, 2H), 1.08 (m, 24H); **^{13}C-NMR ($CDCl_3$, 125 MHz)** δ = 140.2 (CH), 111.7 (CH), 103.3 (C), 95.9 (C), 85.9 (CH), 80.5 (CH), 75.6 (CH), 72.1 (CH), 62.1 (CH), 51.1 (CH), 36.5 (CH_2), 30.5 (CH_2), 30.3 (CH_2), 28.3 (CH_2), 18.7 (6 x CH_3), 12.4 (3 x C), 11.3 (CH_3). **HRMS (ESI^+):** *m/z* calcd. for $C_{24}H_{40}Br_2O_2SiNa^+$: 569.1062 [*M*+Na]$^+$; found: 569.1068.

(+)-neoisoprelaurefucin (2)

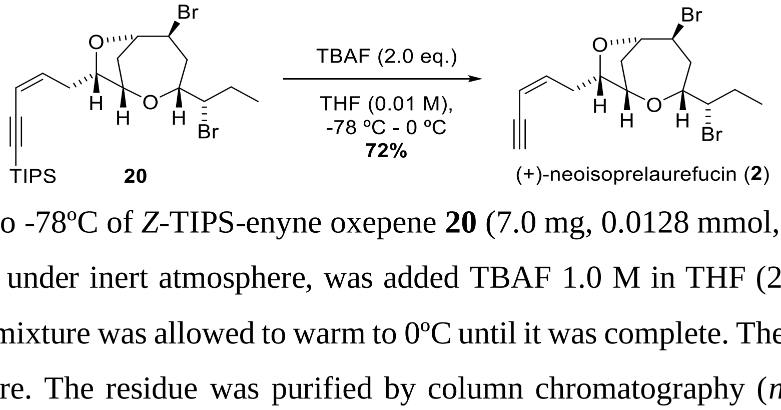

To a solution cooled to -78°C of *Z*-TIPS-enyne oxepene **20** (7.0 mg, 0.0128 mmol, 1.0 equiv.) in 1.3 mL of dry THF (0.01 M) under inert atmosphere, was added TBAF 1.0 M in THF (2.0 equiv., 26 mL, 2.0 equiv.). The reaction mixture was allowed to warm to 0°C until it was complete. The solvent was removed under reduced pressure. The residue was purified by column chromatography (*n*-hexane/EtOAc, 98:2

solvent system) to afford 3.6 mg of (+)-neoisoprelaurefucin (**2**) as a colorless oil ($9.22 \cdot 10^{-3}$ mmol, 72% yield). Spectral data was consistent with the literature.[28]

R$_f$ = 0.54 (*n*-hexane/EtOAc 90:10); **$[\alpha]^{25}_D$** = +16.7 (*c* = 1.02, $CHCl_3$). Literature value: $[\alpha]^{23}_D$ = +17.2 (*c* = 1.30, $CHCl_3$);[28] **^{1}H-NMR ($CDCl_3$, 500 MHz)** δ = 6.12 (ddd, *J* = 10.8, 7.6 & 7.6 Hz, 1H), 5.57 (brd, *J* = 10.8 Hz, 1H), 4.49 (dd, *J* = 8.5 & 4.5 Hz, 1H), 4.36 (dd, *J* = 3.9 & 3.1 Hz, 1H), 4.29 (ddd, *J* = 6.8, 3.2 & 3.2 Hz, 1H), 4.06 (ddd, *J* = 8.6, 4.7 & 3.8 Hz, 1H), 3.94 (dd, *J* = 9.3 & 3.3 Hz, 1H), 3.91 (ddd, *J* = 8.8, 6.3 & 2.9 Hz, 1H), 3.10 (brd, *J* = 1.6 Hz, 1H), 2.93 (ddd, *J* = 14.1, 7.9 & 7.9 Hz, 1H), 2.78 (d, *J* = 15.1 Hz, 1H), 2.66 (ddd, *J* = 13.4, 6.7 & 6.7 Hz, 1H), 2.49 (ddd, *J* = 16.1, 9.2 & 3.5 Hz, 1H), 2.18 (dd, *J* = 16.0 & 2.3 Hz, 1H), 2.12 (ddd, *J* = 14.5, 8.6 & 4.7 Hz, 1H), 1.88 (m, 2H), 1.09 (t, *J* = 7.3 Hz, 3H); **^{13}C-NMR ($CDCl_3$, 125 MHz)** δ 141.5 (CH), 110.3 (CH), 85.7 (CH), 82.0 (CH), 80.5 (CH), 80.2 (C), 75.6 (CH), 72.1 (CH), 62.3 (CH), 51.0 (CH), 36.3 (CH_2), 30.5 (CH_2), 30.3 (CH_2), 28.4 (CH_2), 12.5 (CH_3); **HRMS (ESI$^+$):** *m/z* calcd. for $C_{15}H_{20}Br_2O_2Na^+$: 412.9728; found $[M+Na]^+$: 412.9740.

4. Comparison of NMR data of natural and synthetic (+)-isolaurepinnacin (1)

Table S3. Comparative ^{1}H NMR Data for (+)-isolaurepinnacin (**1**)

Natural (**Masamune**) (400 MHz, $CDCl_3$)	Δδ Nat.-Syn.	Synthetic (**Padrón**) (500 MHz, $CDCl_3$)
6.27 (ddt, *J* = 16, 0.5 & 7 Hz, 1H)	0	6.27 (dt, *J* = 14.7 & 7.2 Hz, 1H)
5.83 (dt, *J* = 8.0 & 4.0 Hz, 1H)<br>5.80 (dt, *J* = 8.0 & 4.0 Hz, 1H)	0.01	5.82 (brt, *J* = 4.1 Hz, 2H)
5.59 (ddt, *J* = 16.0, 2.0, & 1.5 Hz, 1H)	0	5.59 (ddt, *J* = 15.9, 3.7 & 1.5 Hz, 1H)
3.92 (dt, *J* = 9.0 & 4.0 Hz, 1H)	0	3.92 (dt, *J* = 8.0 & 3.9 Hz, 1H)
3.89 (dt, *J* = 5.0 & 4.0 Hz, 1H)	0	3.89 (dt, *J* = 4.5 & 3.4 Hz, 1H)
3.59 (ddd, *J* = 10.0, 4.0 & 2.0 Hz, 1H)	0	3.59 (ddd, *J* = 10.2, 3.3 & 1.5 Hz, 1H)
3.52 (ddd, *J* = 10.0, 4.0 & 2.0 Hz, 1H)	0.01	3.53 (ddd, *J* = 10.2, 3.9 & 1.5 Hz, 1H)
2.83 (dd, *J* = 2.0 & 0.5 Hz, 1H)	0.02	2.85 (d, *J* = 2.0 Hz, 1H)
2.78 (dddd, *J* = 15.0, 7.0, 4.0 & 1.5 Hz, 1H)	0.01	2.77 (dddd, *J* = 14.9, 6.5, 4.7 & 1.3 Hz, 1H)
2.56 (dddd, *J* = 15.0, 9.0, 7.0 & 1.5 Hz, 1H)	0	2.56 (m, 1H)
2.55 (m, 2H)	0	2.55 (m, 2H)
2.36 (dddd, *J* = 15.0, 4.0, 3.0 & 2 Hz, 1H)	0	2.36 (m, 1H)
2.30 (dddd, *J* = 15, 4, 3 & 2 Hz, 1H)	0	2.30 (m, 1H)
1.98 (ddq, *J* = 14, 5 & 7 Hz, 1H)	0.01	1.99 (ddq, *J* = 14.6, 7.3 & 3.3 Hz, 1H)
1.85 (ddq, *J* = 14, 4 & 7 Hz, 1H)	0	1.85 (ddq, *J* = 14.6, 7.3 & 2.2 Hz, 1H)
0.94 (t, *J* = 7 Hz, 3H)	0.12	1.06 (t, *J* = 7.2 Hz, 3H)

Table S4. Comparative ^{13}C NMR Data for (+)-isolaurepinnacin (**1**)

Natural (**Masamune**) (100 MHz, $CDCl_3$)	Δδ Nat.-Syn.	Synthetic (**Padrón**) (150 MHz, $CDCl_3$)
142.0 (d)	0.2	141.8 (CH)
129.2 (d)	0.1	129.1 (CH)
129.0 (d)	0.1	128.9 (CH)
111.8 (d)	0.1	111.7 (CH)
82.5 (d)	0.6	81.9 (CH)
82.0 (s)	0.2	81.8 (C)
81.2 (d)	0.2	81.0 (CH)
77.0 (d)	0.1	76.9 (CH)
63.6 (d)	0.3	63.3 (CH)
61.3 (d)	0.1	61.4 (CH)
37.6 (t)	0.1	37.5 (CH_2)
33.8 (t)	0.1	33.7 (CH_2)
32.7 (t)	0	32.7 (CH_2)
28.0 (t)	0	28.0 (CH_2)
12.7 (q)	0	12.7 (CH_3)

5. Comparison of NMR data of natural and synthetic (+)-neoisoprelaurefucin (2)

Table S5. Comparative ^{1}H NMR Data for (+)-neoisoprelaurefucin (**2**).

Natural (**Suzuki**) (400 MHz, $CDCl_3$)	Δδ Nat.-Syn.	Synthetic (**our lab**) (500 MHz, $CDCl_3$)
6.12 (ddd, *J* = 11.2, 7.3 & 7.3 Hz, 1H)	0	6.12 (ddd, *J* = 10.8, 7.6 & 7.6 Hz, 1H)
5.57 (brd, *J* = 11.2 Hz, 1H)	0	5.57 (brd, *J* = 10.8 Hz, 1H)
4.49 (dd, *J* = 8.3 & 4.4 Hz, 1H)	0	4.49 (dd, *J* = 8.5 & 4.5 Hz, 1H)
4.36 (dd, *J* = 3.9 & 2.9 Hz, 1H)	0	4.36 (dd, *J* = 3.9 & 3.1 Hz, 1H)
4.29 (ddd, *J* = 4.4, 3.4 & 2.4 Hz, 1H)	0	4.29 (ddd, *J* = 6.8, 3.2 & 3.2 Hz, 1H)
4.07 (ddd, *J* = 8.8, 4.9 & 3.4 Hz, 1H)	0.01	4.06 (ddd, *J* = 8.6, 4.7 & 3.8 Hz, 1H)
3.94 (dd, *J* = 9.3 & 3.4 Hz, 1H)	0	3.94 (dd, *J* = 9.3 & 3.3 Hz, 1H)
3.91 (ddd, *J* = 8.3, 5.8 & 2.9 Hz, 1H)	0	3.91 (ddd, *J* = 8.8, 6.3 & 2.9 Hz, 1H)
3.10 (brd, *J* = 2.0 Hz, 1H)	0	3.10 (brd, *J* = 1.6 Hz, 1H)
2.93 (ddd, *J* = 14.2, 7.3 & 5.8 Hz, 1H)	0	2.93 (ddd, *J* = 14.1, 7.9 & 7.9 Hz, 1H)
2.78 (d, *J* = 15.1 Hz, 1H)	0	2.78 (d, *J* = 15.1 Hz, 1H)
2.66 (ddd, *J* = 14.2, 8.3 & 7.3 Hz, 1H)	0	2.66 (ddd, *J* = 13.4, 6.7 & 6.7 Hz, 1H)
2.49 (ddd, *J* = 15.1, 9.3 & 3.4 Hz, 1H)	0	2.49 (ddd, *J* = 16.1, 9.2 & 3.5 Hz, 1H)
2.18 (dd, *J* = 15.1 & 2.4 Hz, 1H)	0	2.18 (dd, *J* = 16.0 & 2.3 Hz, 1H)
2.12 (ddd, *J* = 15.1, 8.3 & 3.9 Hz, 1H)	0	2.12 (ddd, *J* = 14.5, 8.6 & 4.7 Hz, 1H)
1.91 (m, 2H)	0.03	1.88 (m, 2H)
1.09 (t, *J* = 7.3 Hz, 3H)	0	1.09 (t, *J* = 7.3 Hz, 3H)

Tabla S6. Comparative ^{13}C NMR Data for (+)-neoisoprelaurefucin (**2**).

Natural (**Suzuki**) (100 MHz, $CDCl_3$)	Δδ Nat.-Syn.	Synthetic (P**adrón**) (125 MHz, $CDCl_3$)
141.5	0	141.5 (CH)
110.4	0.1	110.3 (CH)
85.7	0	85.7 (CH)
82.0	0	82.0 (CH)
80.6	0.1	80.5 (CH)
80.2	0	80.2 (C)
75.6	0	75.6 (CH)
72.1	0	72.1 (CH)
62.3	0	62.3 (CH)
51.1	0.1	51.0 (CH)
36.4	0.1	36.3 (CH_2)
30.5	0	30.5 (CH_2)
30.3	0	30.3 (CH_2)
28.5	0.1	28.4 (CH_2)
12.5	0	12.5 (CH_3)

6. References

[1] K. B. Sharpless, K. Akashi, *J. Am. Chem. Soc.* **1976**, *98*, 1986–1987.
[2] W. Perrin, D.; Armarego, *Purification of Laboratory Chemicals*, Buterworth-Heinemann, **1996**.
[3] H. E. Gottlieb, V. Kotlyar, A. Nudelman, *J. Org. Chem.* **1997**, *62*, 7512–7515.
[4] S. F. Martin, J. A. Dodge, *Tetrahedron Lett.* **1991**, *32*, 3017–3020.
[5] J. A. Dodge, J. I. Trujillo, M. Presnell, *J. Org. Chem.* **1994**, *59*, 234–236.
[6] S. D. Lepore, Y. He, *J. Org. Chem.* **2003**, *68*, 8261–8263.
[7] L. J. Baird, M. S. M. Timmer, P. H. Teesdale-Spittle, J. E. Harvey, *J. Org. Chem.* **2009**, *74*, 2271–2277.
[8] Y. Gao, J. M. Klunder, R. M. Hanson, H. Masamune, S. Y. Ko, K. B. Sharpless, *J. Am. Chem. Soc.* **1987**, *109*, 5765–5780.
[9] R. Corriu, J. Masse, *J. Organomet. Chem.* **1973**, *57*, C5–C8.
[10] R. J. P. Corriu, J. Masse, D. Samate, *J. Organomet. Chem.* **1975**, *93*, 71–80.
[11] D. A. Cruz, V. Sinka, V. S. Martín, J. I. Padrón, *J. Org. Chem.* **2018**, *83*, 12632–12647.
[12] Z. Wang, Y. Gu, A. J. Zapata, G. B. Hammond, *J. Fluor. Chem.* **2001**, *107*, 127–132.
[13] B. S. Dyson, J. W. Burton, T. Sohn, B. Kim, H. Bae, D. Kim, *J. Am. Chem. Soc.* **2012**, *134*, 11781–11790.
[14] M. T. Crimmins, M. T. Powell, *J. Am. Chem. Soc.* **2003**, *125*, 7592–7595.
[15] M. T. Crimmins, A. C. DeBaillie, *Org. Lett.* **2003**, *5*, 3009–3011.
[16] K. Miyashita, T. Tsunemi, T. Hosokawa, M. Ikejiri, T. Imanishi, *J. Org. Chem.* **2008**, *73*, 5360–5370.
[17] A. Abate, E. Brenna, C. Fuganti, L. Malpezzi, S. Serra, *Tetrahedron: Asymmetry* **2007**, *18*, 1145–1153.
[18] H. Kim, H. Lee, D. Lee, S. Kim, D. Kim, *J. Am. Chem. Soc.* **2007**, *129*, 2269–2274.
[19] B. Kim, T. Sohn, D. Kim, R. S. Paton, *Chem. – A Eur. J.* **2018**, *24*, 2634–2642.
[20] A. Fukuzawa, T. Masamune, *Tetrahedron Lett.* **1981**, *22*, 4081–4084.
[21] D. Berger, L. E. Overman, P. A. Renhowe, *J. Am. Chem. Soc.* **1997**, *119*, 2446–2452.
[22] H. Lee, H. Kim, T. Yoon, B. Kim, S. Kim, H.-D. Kim, D. Kim, *J. Org. Chem.* **2005**, *70*, 8723–8729.
[23] N. F. Langille, T. F. Jamison, *Org. Lett.* **2006**, *8*, 3761–3764.
[24] F. Le Bideau, F. Gilloir, Y. Nilsson, C. Aubert, M. Malacria, *Tetrahedron* **1996**, *52*, 7487–7510.
[25] H.-D. Xu, H. Wu, C. Jiang, P. Chen, M.-H. Shen, *Tetrahedron Lett.* **2016**, *57*, 2915–2918.
[26] S. S. Sohn, E. L. Rosen, J. W. Bode, *J. Am. Chem. Soc.* **2004**, *126*, 14370–14371.
[27] J. Cho, Y. M. Lee, D. Kim, S. Kim, *J. Org. Chem.* **2009**, *74*, 3900–3904.
[28] M. Suzuki, Y. Mizuno, Y. Matsuo, M. Masuda, *Phytochemistry* **1996**, *43*, 121–124.

7. NMR spectra

^{1}H-NMR ($CDCl_3$, 400 MHz)

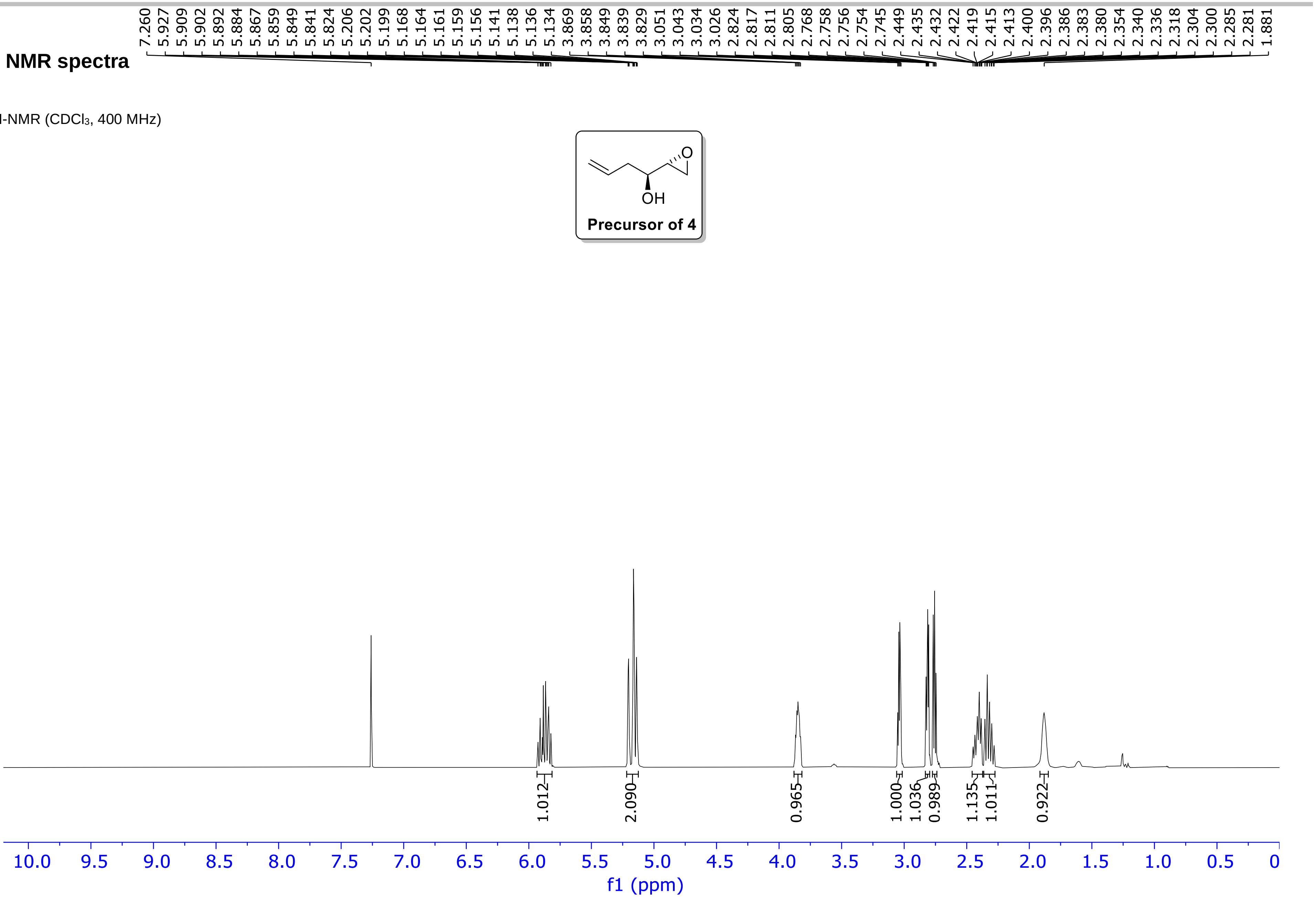

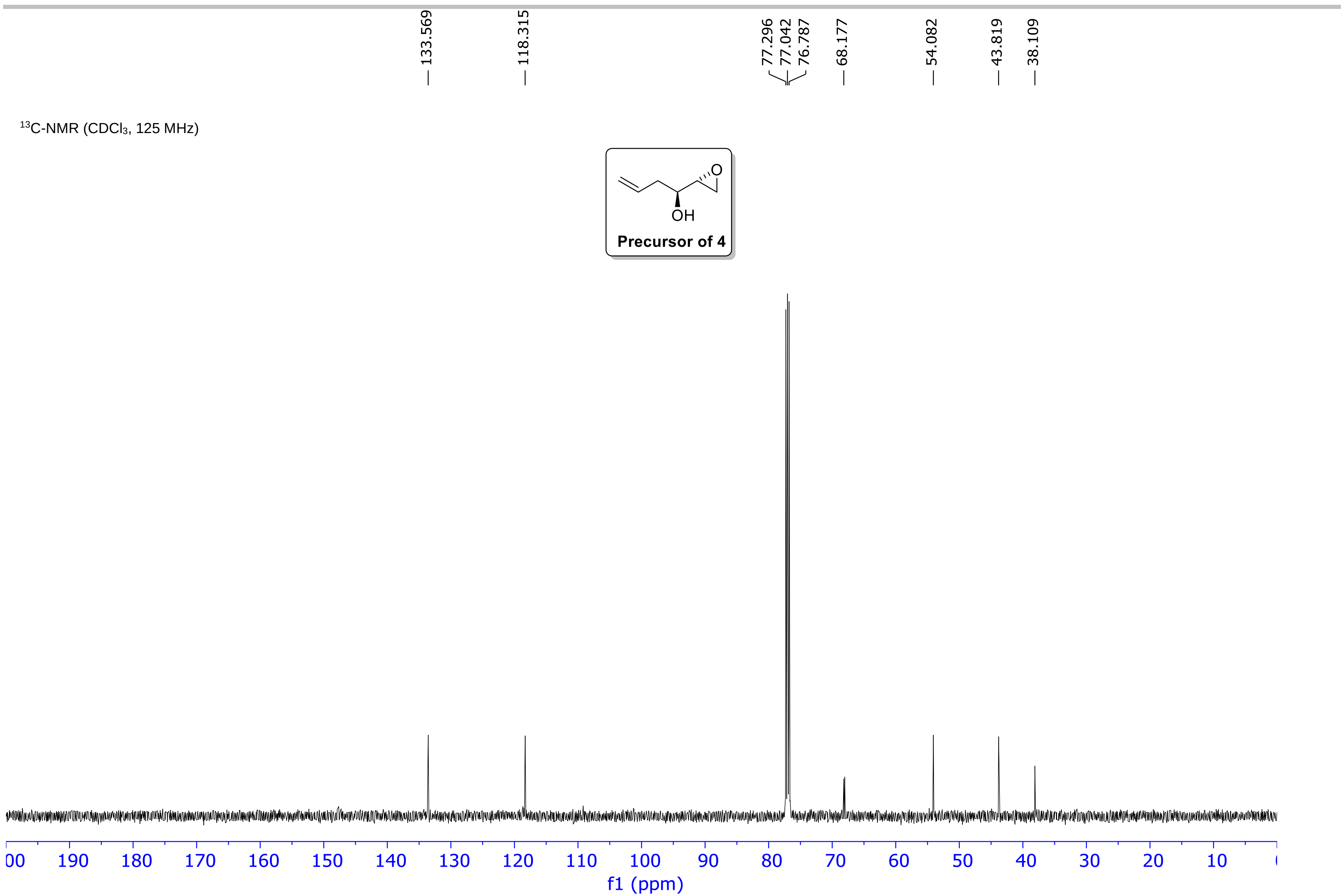
13C-NMR (CDCl3, 125 MHz)
133.569
118.315
77.296
77.042
76.787
68.177
54.082
43.819
38.109
OH
O
Precursor of 4
f1 (ppm)

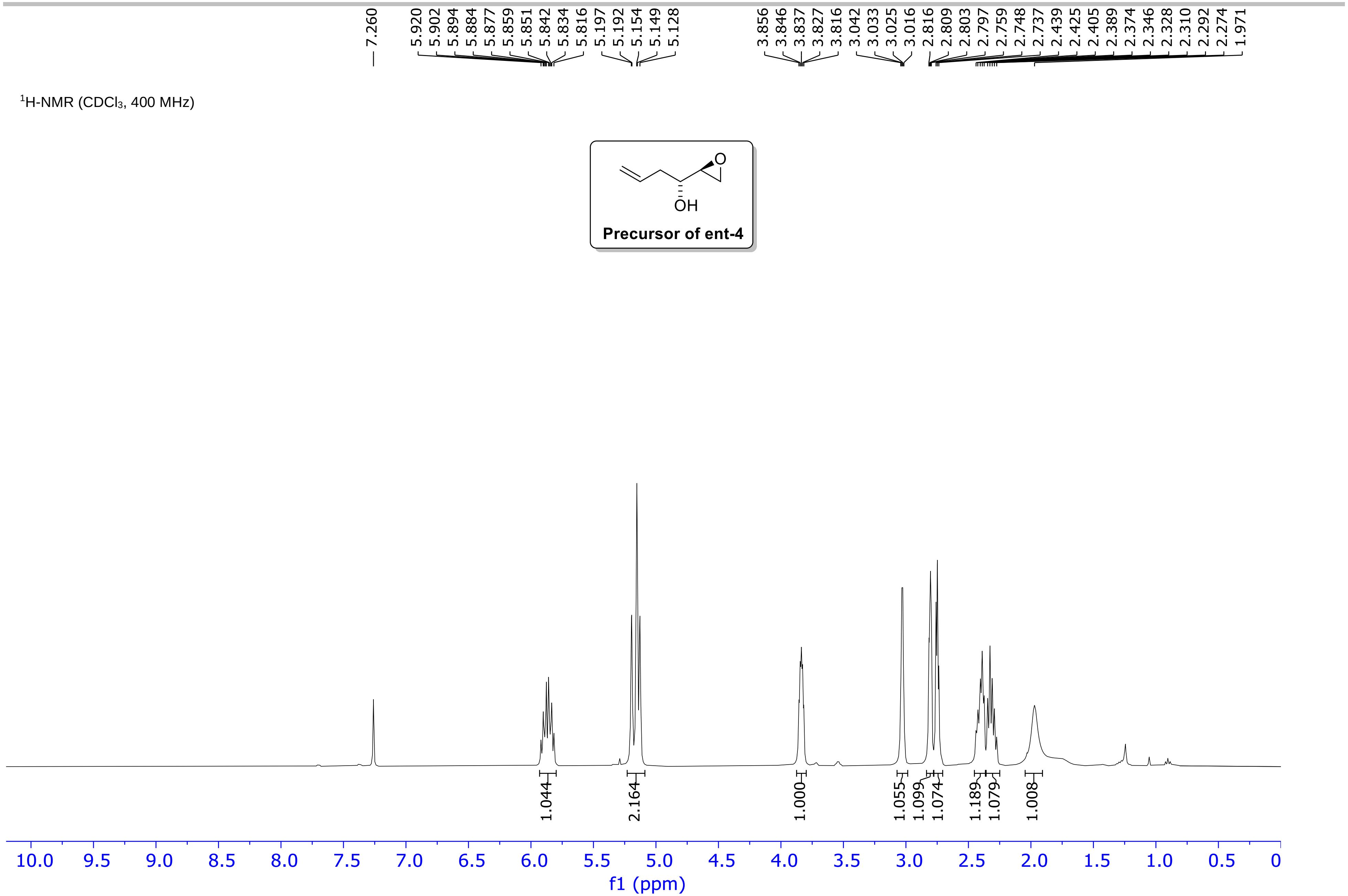

1H-NMR (CDCl3, 400 MHz)
OH
O
Precursor of ent-4
7.260
5.920 5.902 5.894 5.884 5.877 5.859 5.851 5.842 5.834 5.816
5.197 5.192 5.154 5.149 5.128
3.856 3.846 3.837 3.827 3.816
3.042 3.033 3.025 3.016
2.816 2.809 2.803 2.797 2.759 2.748 2.737
2.439 2.425 2.405 2.389 2.374 2.346 2.328 2.310 2.292 2.274
1.971
1.044 2.164 1.000 1.055 1.099 1.074 1.189 1.079 1.008
f1 (ppm)

^{13}C-NMR ($CDCl_3$, 100 MHz)

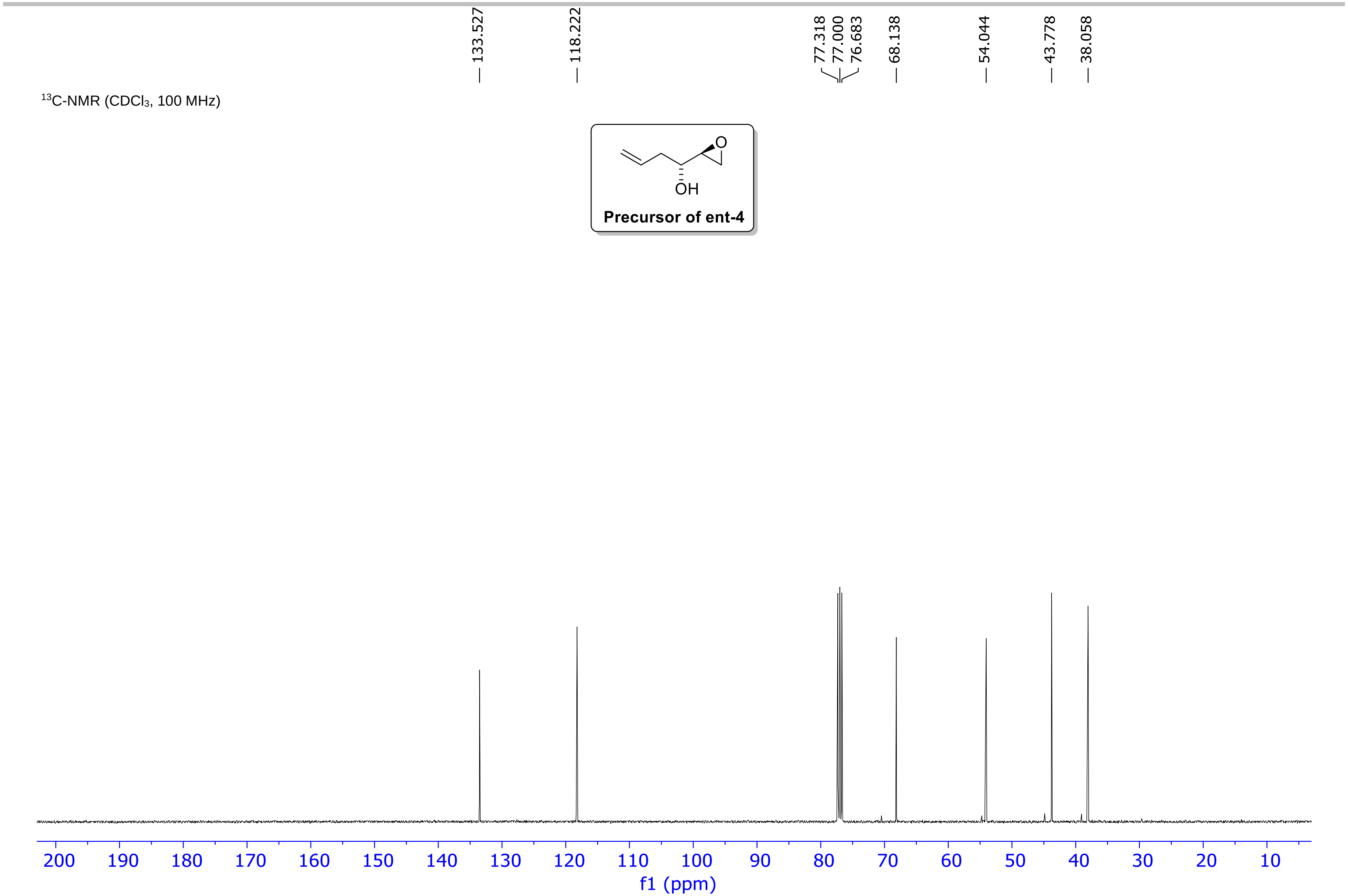

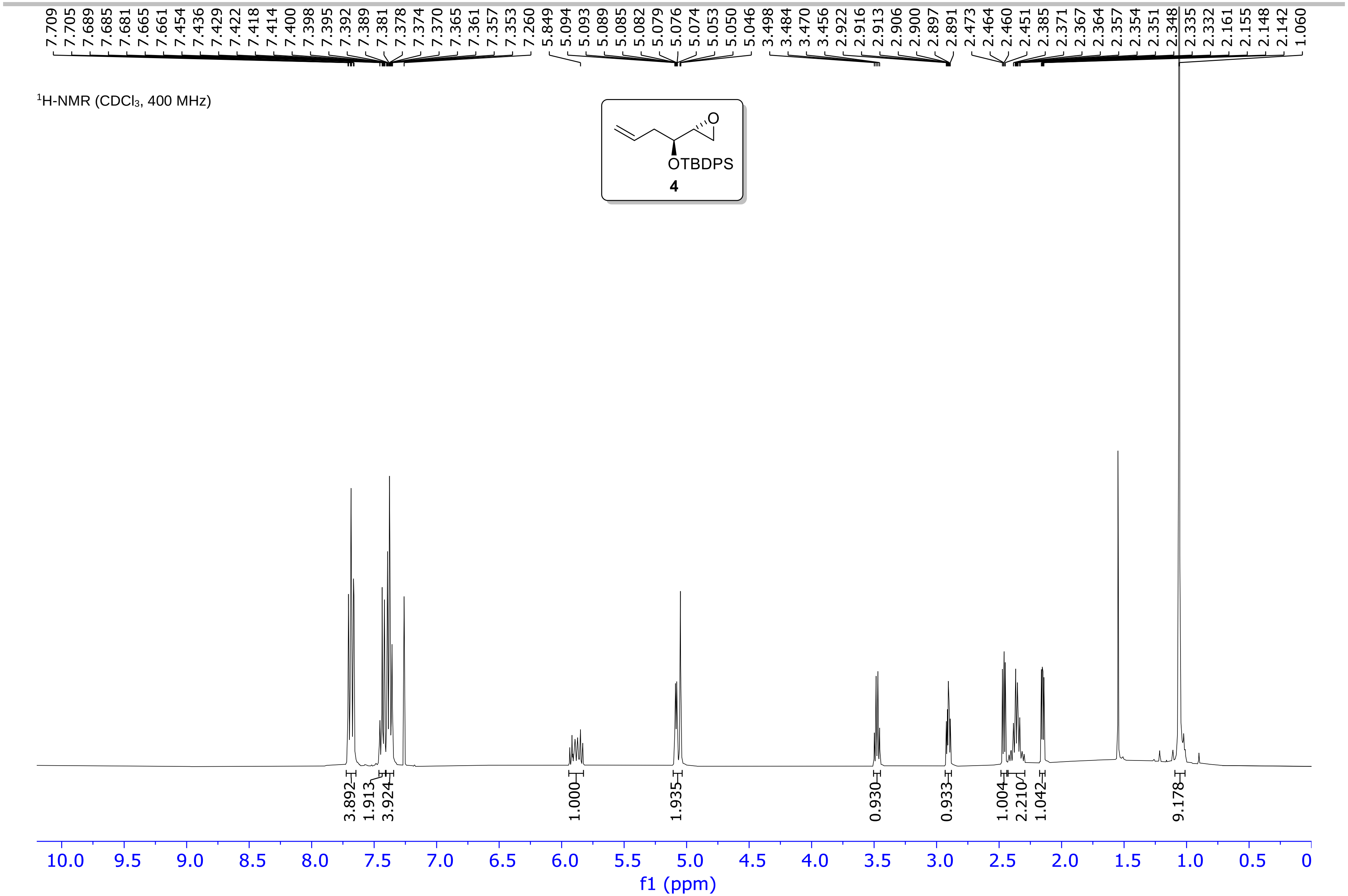

7.709 7.705 7.689 7.685 7.681 7.665 7.661 7.454 7.436 7.429 7.422 7.418 7.414 7.400 7.398 7.395 7.392 7.389 7.381 7.378 7.374 7.370 7.365 7.361 7.357 7.353 7.260 5.849 5.094 5.093 5.089 5.085 5.082 5.079 5.076 5.074 5.053 5.050 5.046 3.498 3.484 3.470 3.456 2.922 2.916 2.913 2.906 2.900 2.897 2.891 2.473 2.464 2.460 2.451 2.385 2.371 2.367 2.364 2.357 2.354 2.351 2.348 2.335 2.332 2.161 2.155 2.148 2.142 1.060
1H-NMR (CDCl3, 400 MHz)
O
OTBDPS
4
3.892
1.913
3.924
1.000
1.935
0.930
0.933
1.004
2.210
1.042
9.178
10.0 9.5 9.0 8.5 8.0 7.5 7.0 6.5 6.0 5.5 5.0 4.5 4.0 3.5 3.0 2.5 2.0 1.5 1.0 0.5 0
f1 (ppm)

^{13}C-NMR ($CDCl_3$, 100 MHz)

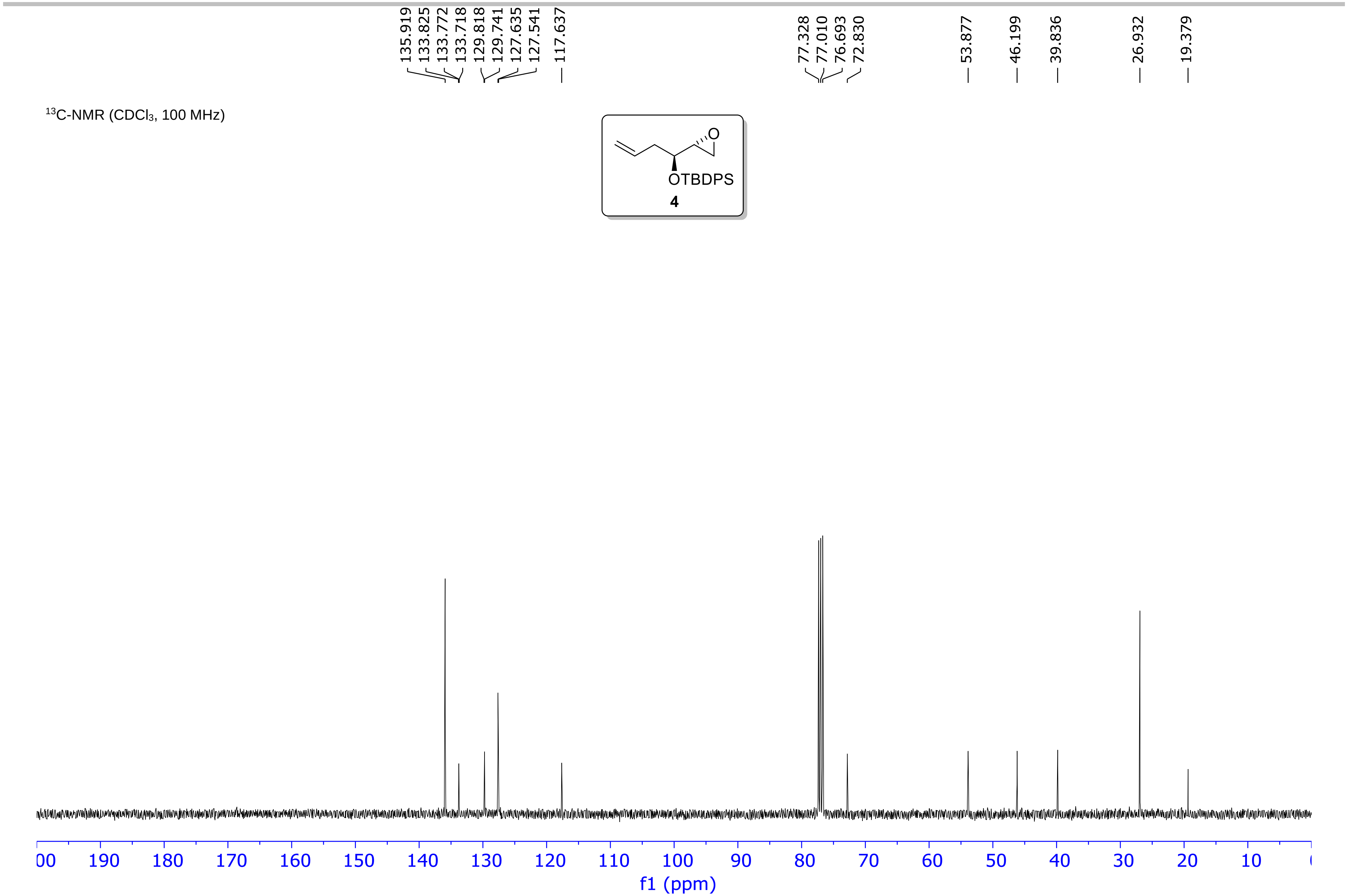

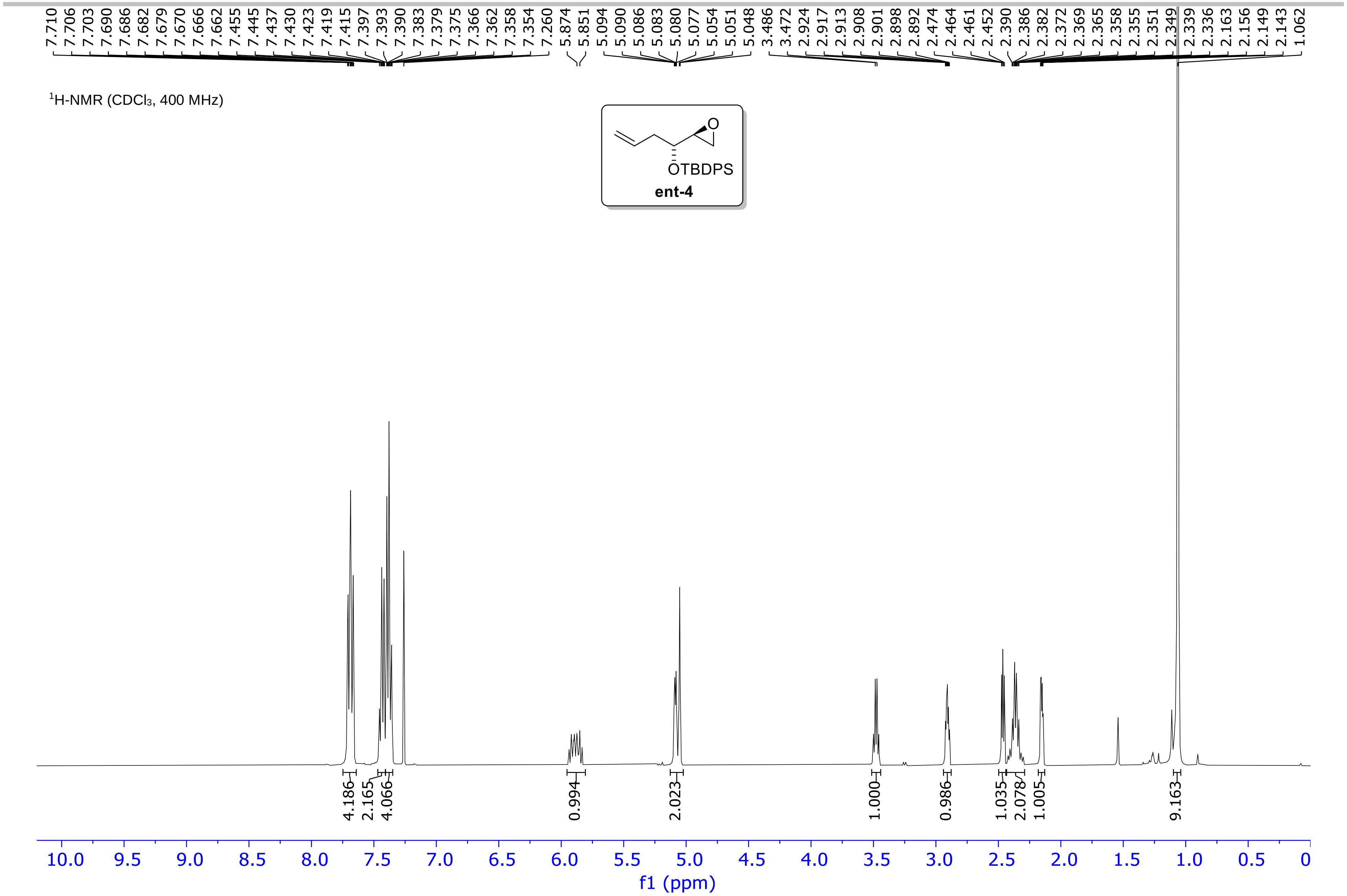
7.710 7.706 7.703 7.690 7.686 7.682 7.679 7.670 7.666 7.662 7.455 7.445 7.437 7.430 7.423 7.419 7.415 7.397 7.393 7.390 7.383 7.379 7.375 7.366 7.362 7.358 7.354 7.260 5.874 5.851 5.094 5.090 5.086 5.083 5.080 5.077 5.054 5.051 5.048 3.486 3.472 2.924 2.917 2.913 2.908 2.901 2.898 2.892 2.474 2.464 2.461 2.452 2.390 2.386 2.382 2.372 2.369 2.365 2.358 2.355 2.351 2.349 2.339 2.336 2.163 2.156 2.149 2.143 1.062
¹H-NMR (CDCl3, 400 MHz)
O
OTBDPS
ent-4
4.186 2.165 4.066 0.994 2.023 1.000 0.986 1.035 2.078 1.005 9.163
10.0 9.5 9.0 8.5 8.0 7.5 7.0 6.5 6.0 5.5 5.0 4.5 4.0 3.5 3.0 2.5 2.0 1.5 1.0 0.5 0
f1 (ppm)

^{13}C-NMR ($CDCl_3$, 100 MHz)

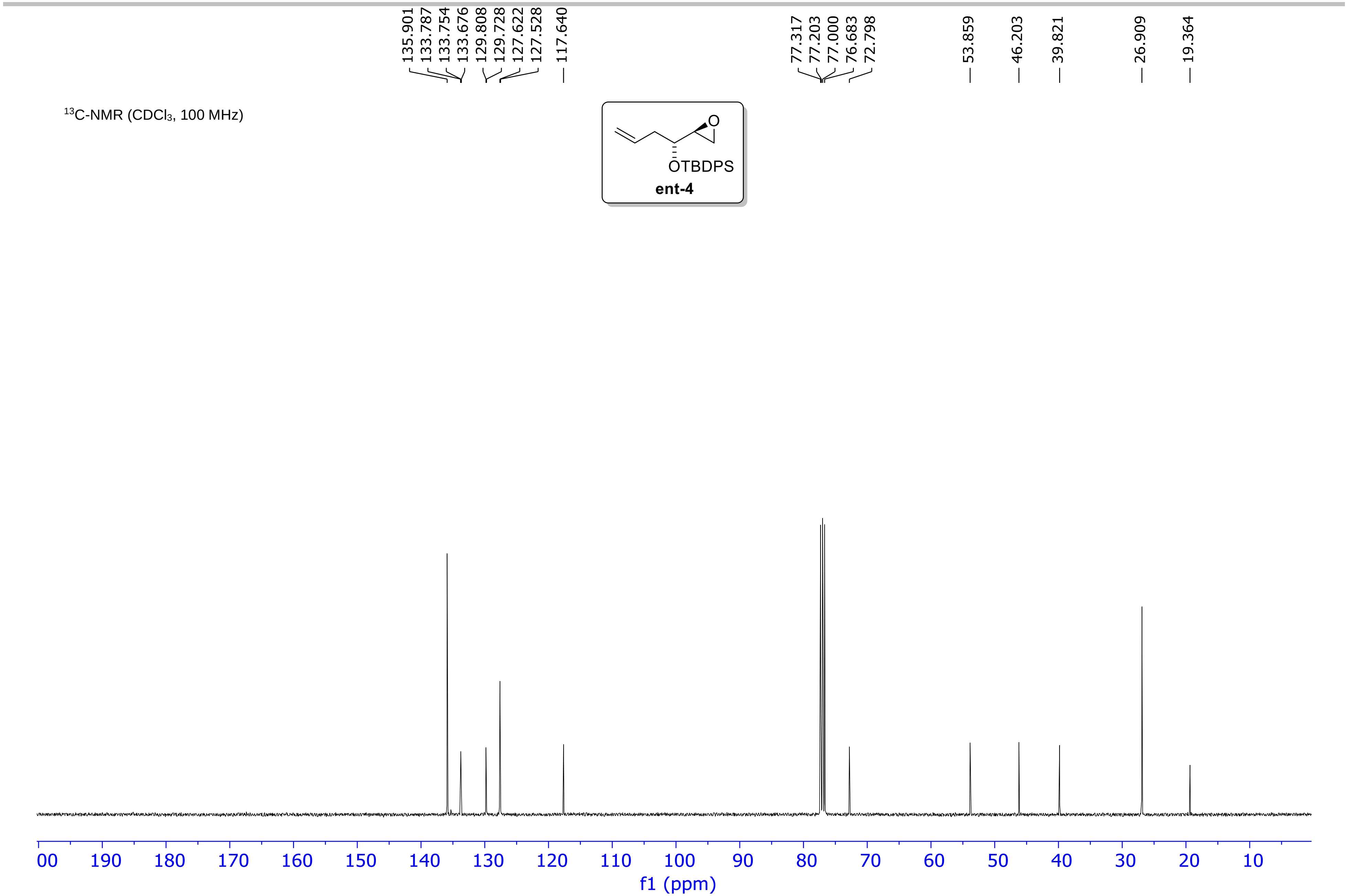

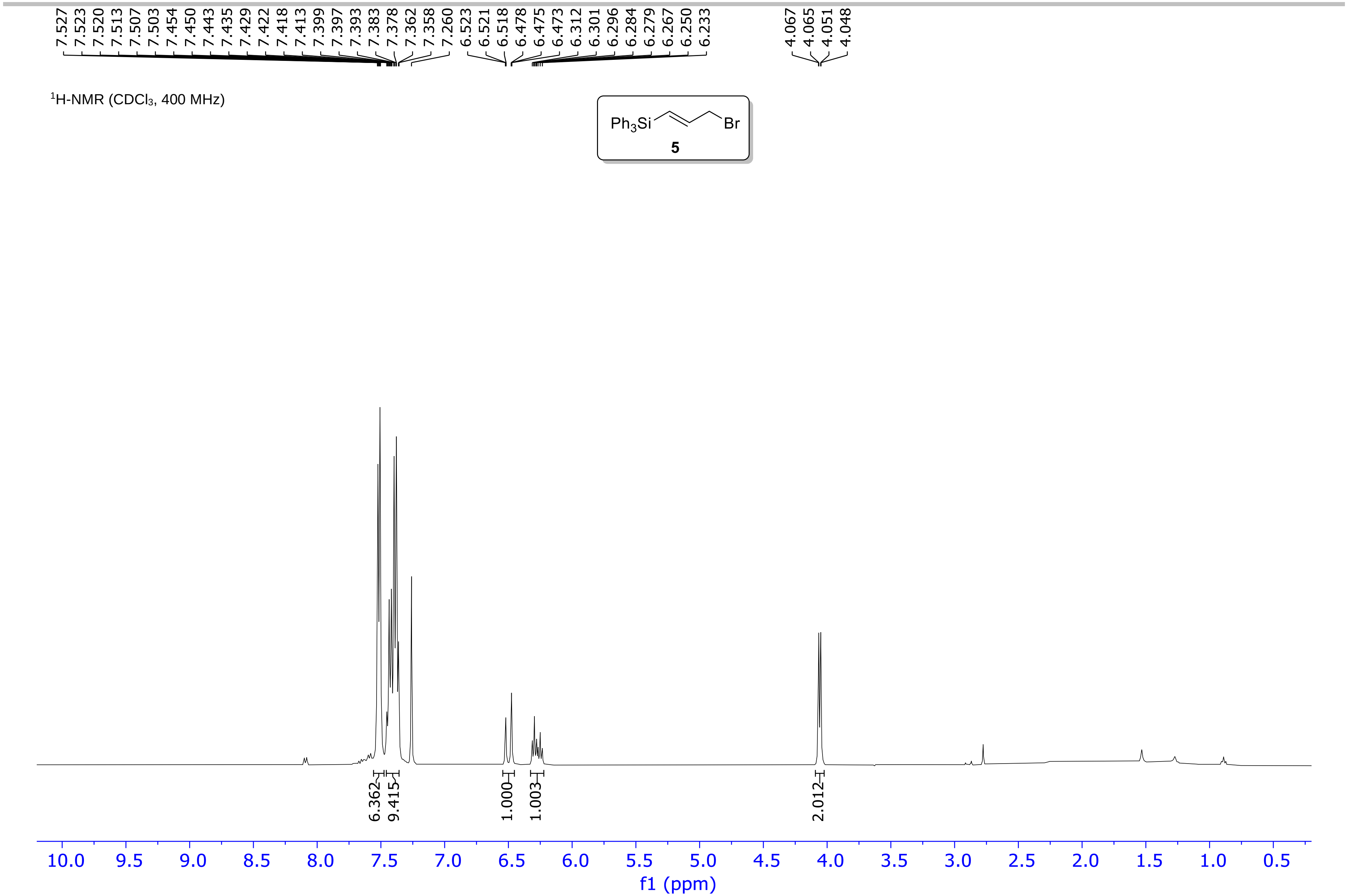

7.527
7.523
7.520
7.513
7.507
7.503
7.454
7.450
7.443
7.435
7.429
7.422
7.418
7.413
7.399
7.397
7.393
7.383
7.378
7.362
7.358
7.260
6.523
6.521
6.518
6.478
6.475
6.473
6.312
6.301
6.296
6.284
6.279
6.267
6.250
6.233
4.067
4.065
4.051
4.048
¹H-NMR (CDCl₃, 400 MHz)
Ph3Si
Br
5
6.362
9.415
1.000
1.003
2.012
10.0 9.5 9.0 8.5 8.0 7.5 7.0 6.5 6.0 5.5 5.0 4.5 4.0 3.5 3.0 2.5 2.0 1.5 1.0 0.5
f1 (ppm)

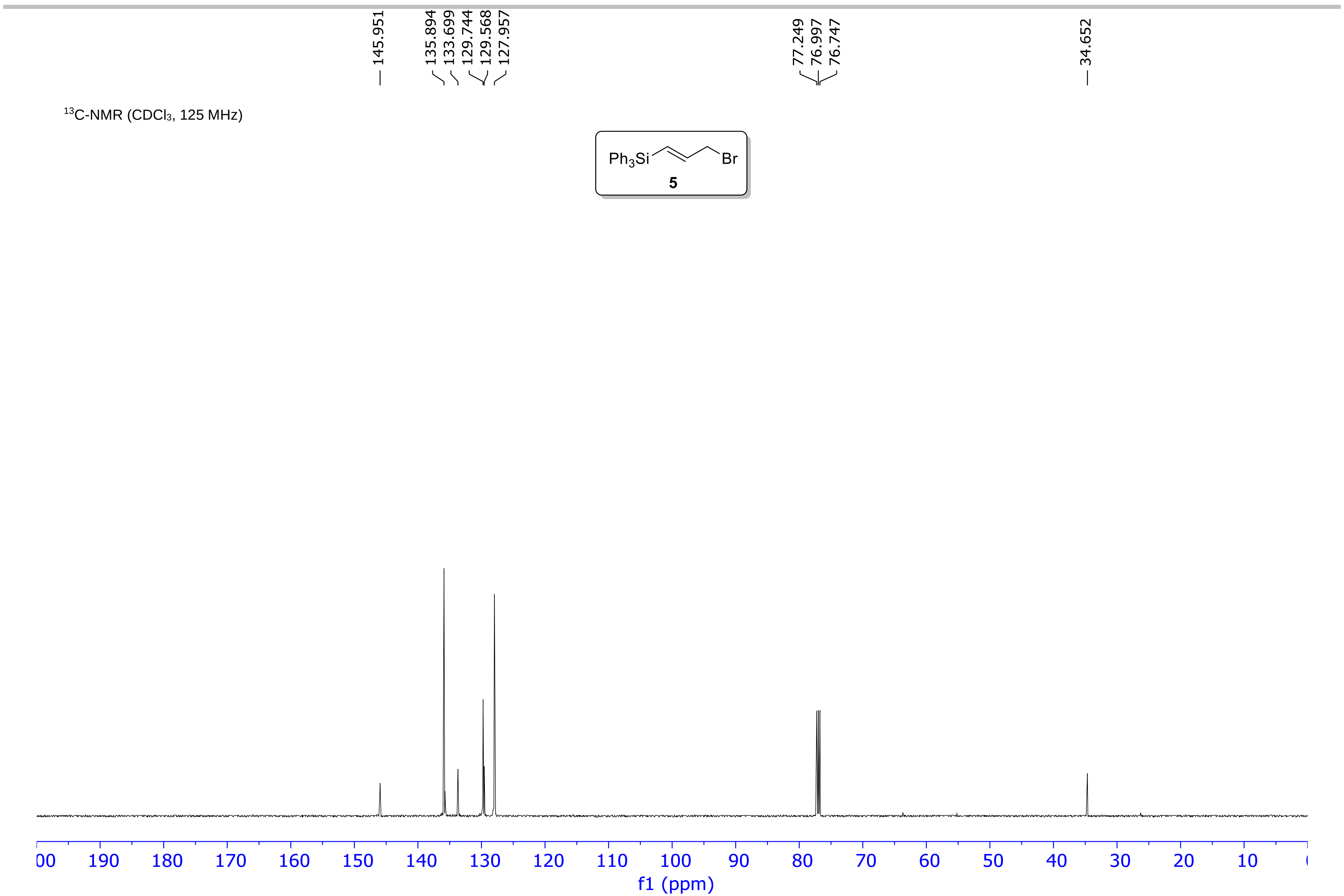
145.951
135.894
133.699
129.744
129.568
127.957
77.249
76.997
76.747
34.652
13C-NMR (CDCl3, 125 MHz)
Ph3Si
Br
5
00
190
180
170
160
150
140
130
120
110
100
90
80
70
60
50
40
30
20
10
f1 (ppm)

^{1}H-NMR ($CDCl_3$, 400 MHz)

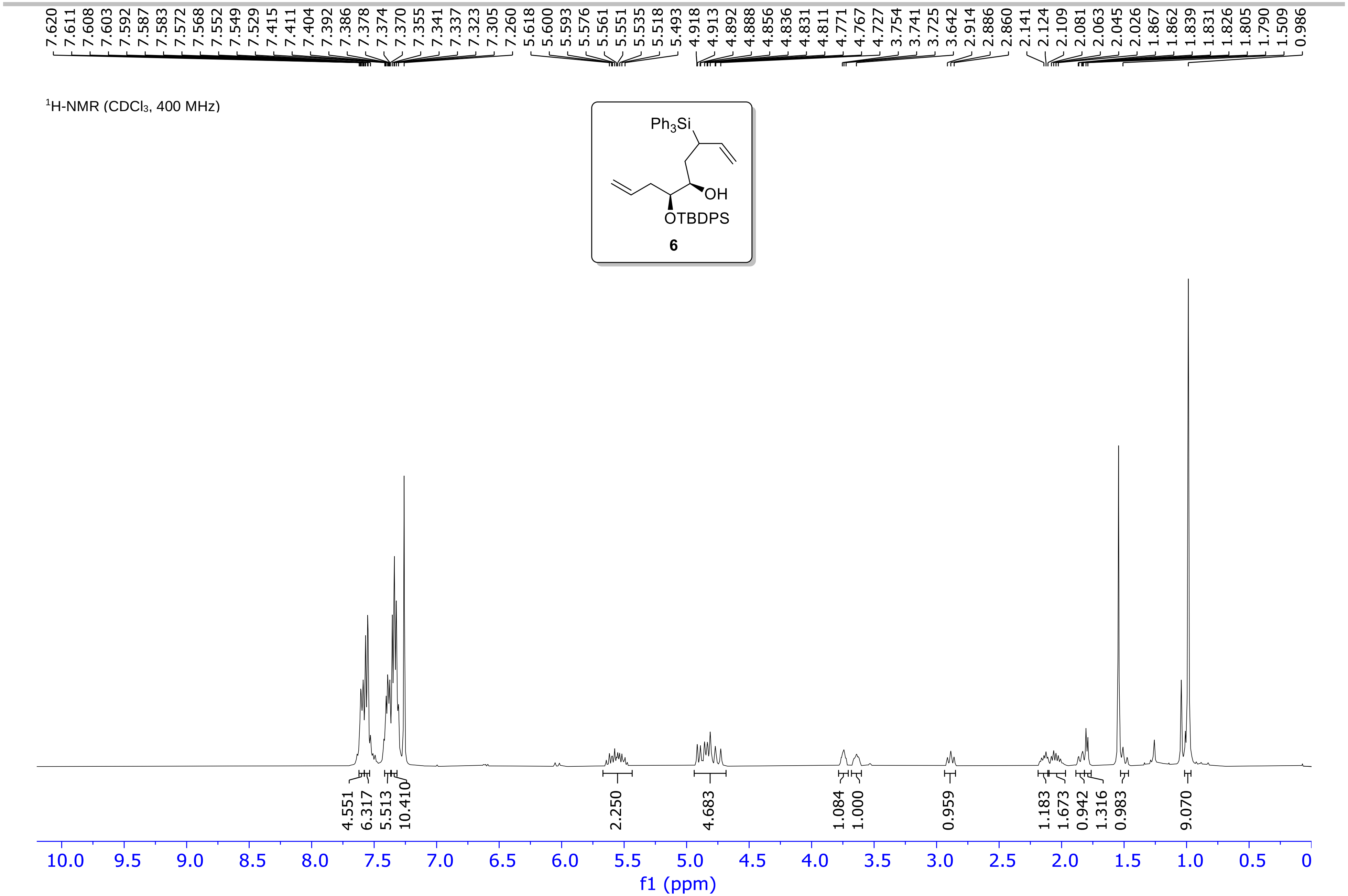

^{13}C-NMR (CDCl$_3$, 100 MHz)

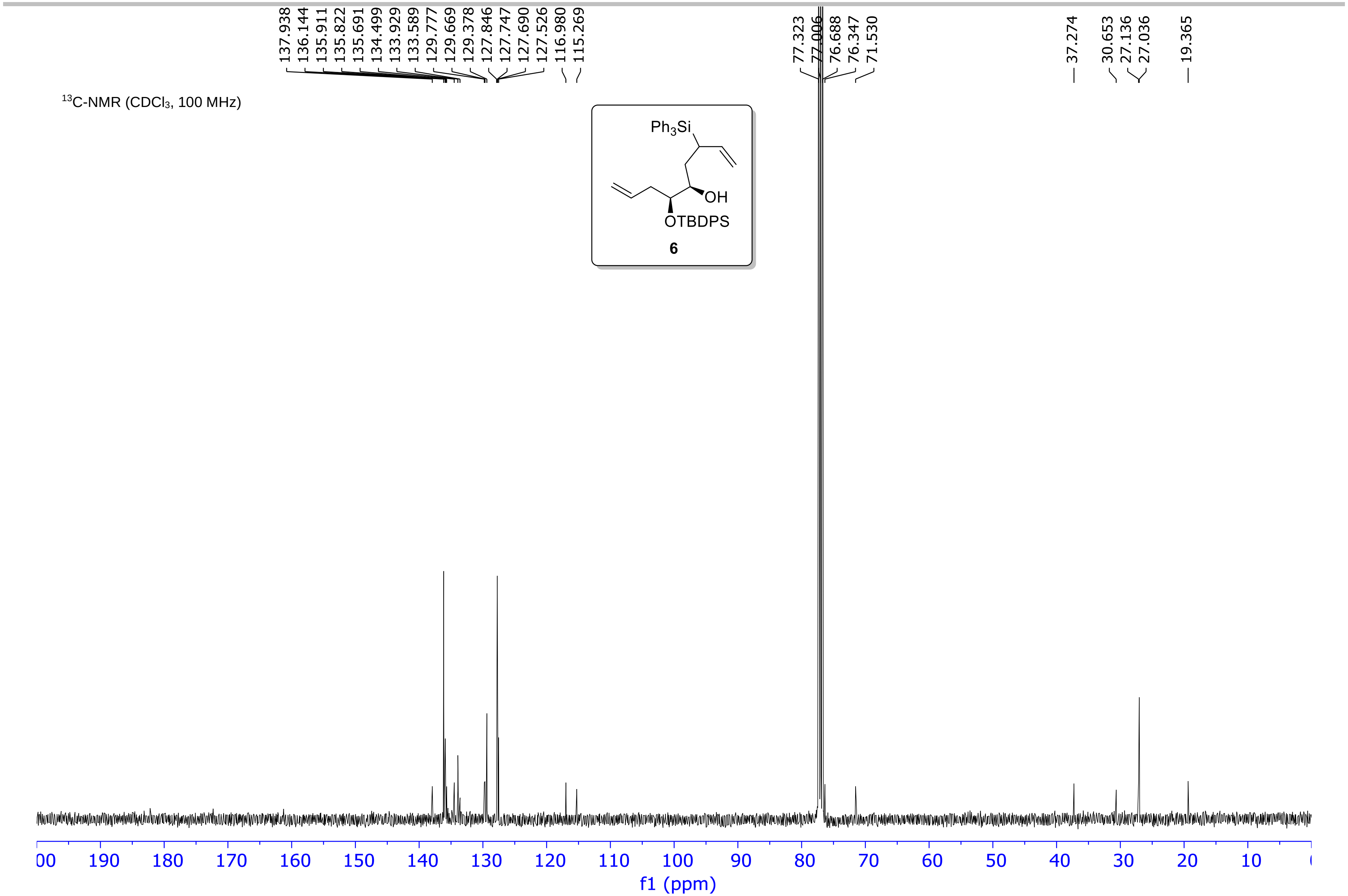

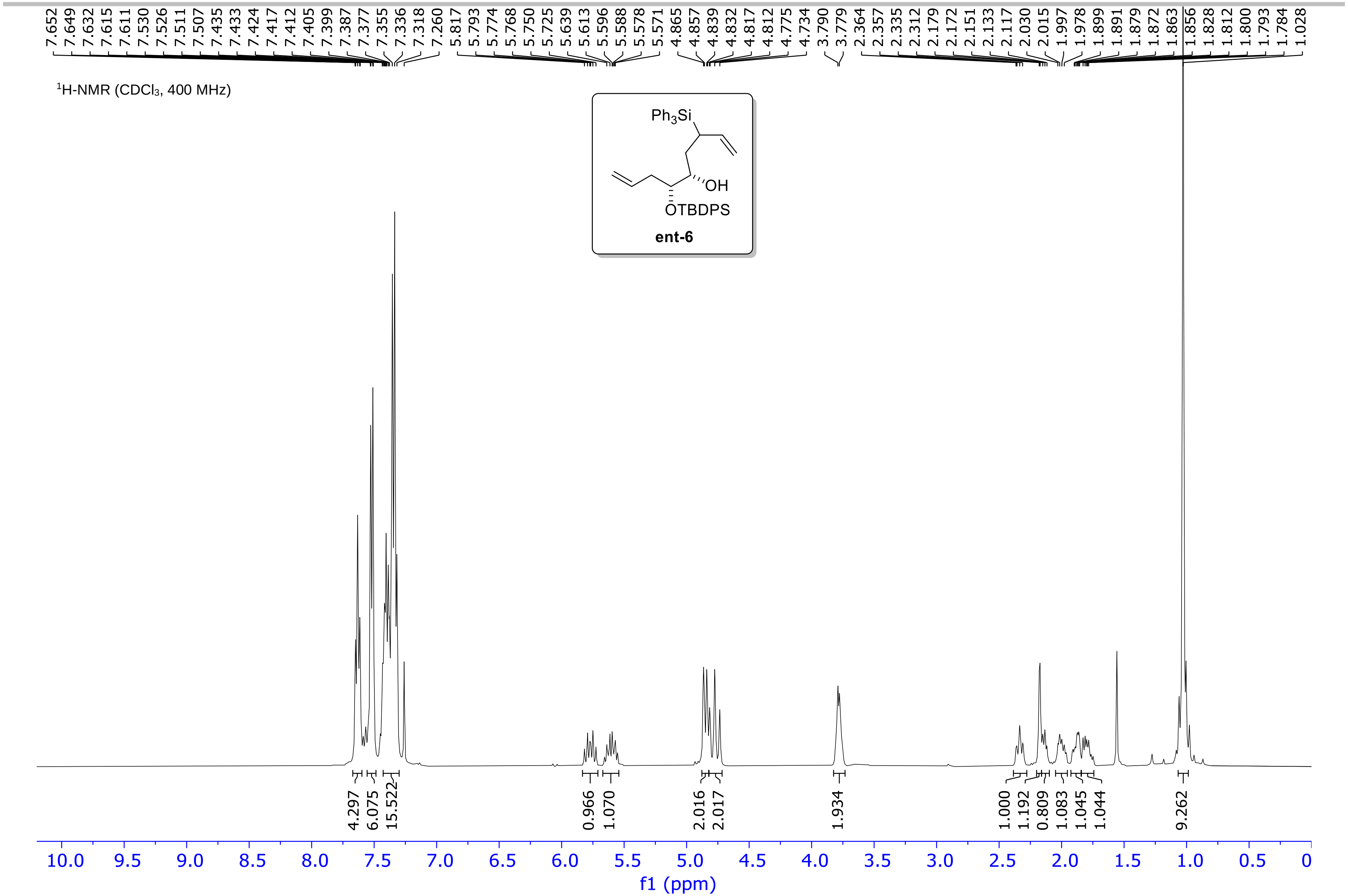

^{1}H-NMR (CDCl$_3$, 400 MHz)
Ph$_3$Si
OH
OTBDPS
ent-6
7.652 7.649 7.632 7.615 7.611 7.530 7.526 7.511 7.507 7.435 7.433 7.424 7.417 7.412 7.405 7.399 7.387 7.377 7.355 7.336 7.318 7.260 5.817 5.793 5.774 5.768 5.750 5.725 5.639 5.613 5.596 5.588 5.578 5.571 4.865 4.857 4.839 4.832 4.817 4.812 4.775 4.734 3.790 3.779 2.364 2.357 2.335 2.312 2.179 2.172 2.151 2.133 2.117 2.030 2.015 1.997 1.978 1.899 1.891 1.879 1.872 1.863 1.856 1.828 1.812 1.800 1.793 1.784 1.028
4.297 6.075 15.522 0.966 1.070 2.016 2.017 1.934 1.000 1.192 0.809 1.083 1.045 1.044 9.262
10.0 9.5 9.0 8.5 8.0 7.5 7.0 6.5 6.0 5.5 5.0 4.5 4.0 3.5 3.0 2.5 2.0 1.5 1.0 0.5 0
f1 (ppm)

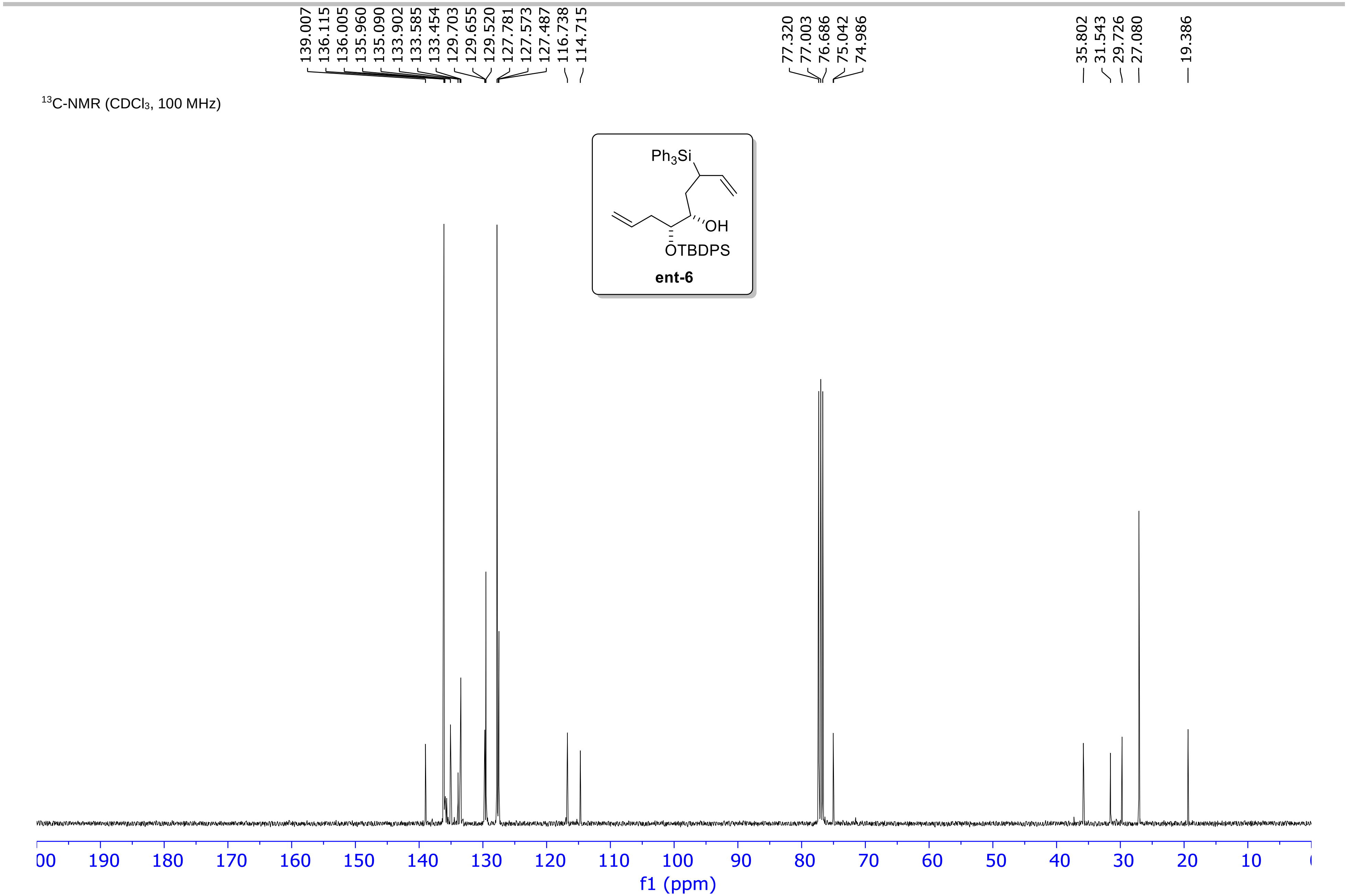

139.007
136.115
136.005
135.960
135.090
133.902
133.585
133.454
129.703
129.655
129.520
127.781
127.573
127.487
116.738
114.715
77.320
77.003
76.686
75.042
74.986
35.802
31.543
29.726
27.080
19.386
^{13}C-NMR ($CDCl_3$, 100 MHz)
Ph_3Si
OH
OTBDPS
ent-6
00
190
180
170
160
150
140
130
120
110
100
90
80
70
60
50
40
30
20
10
f1 (ppm)

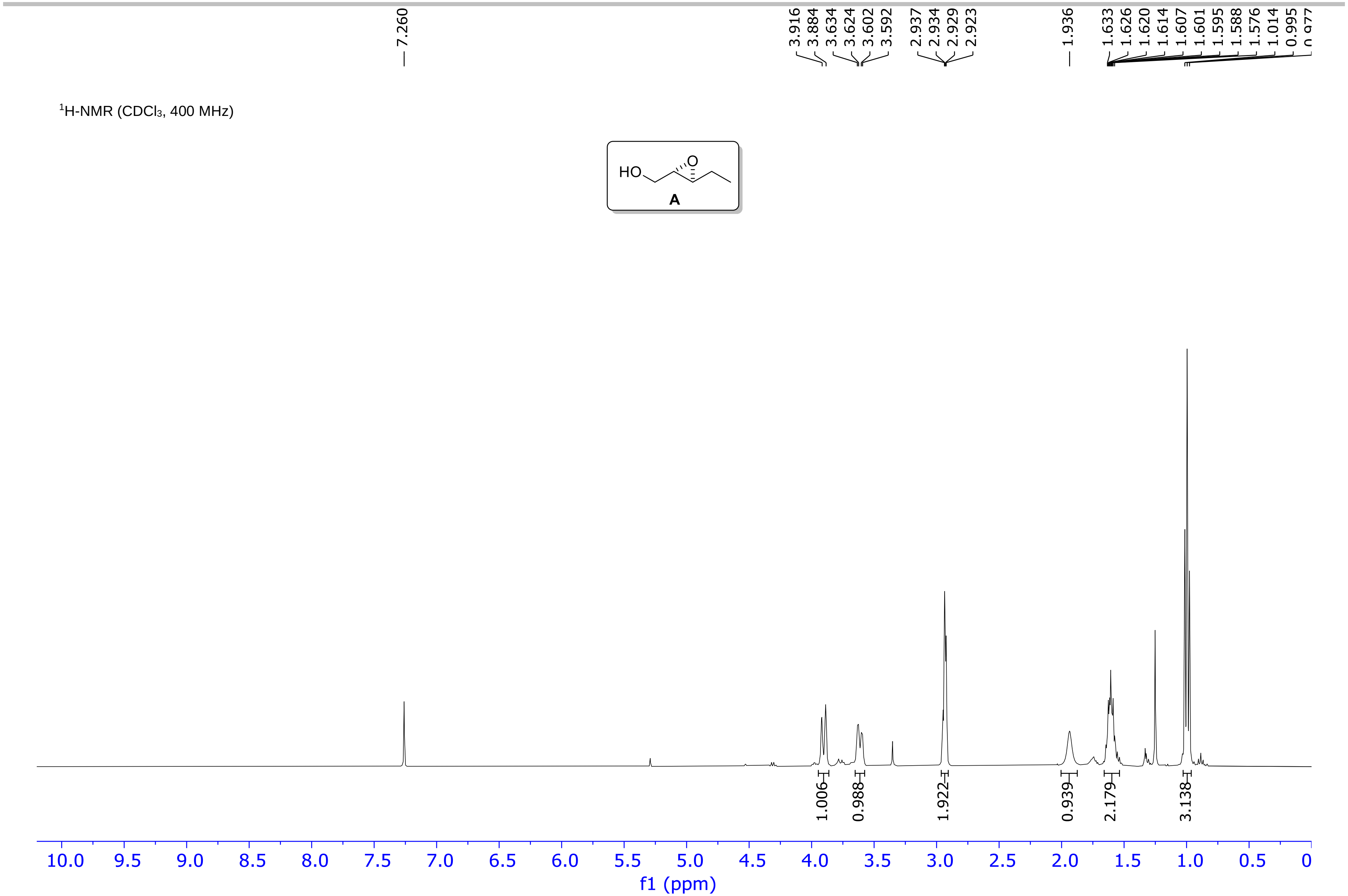
7.260
3.916
3.884
3.634
3.624
3.602
3.592
2.937
2.934
2.929
2.923
1.936
1.633
1.626
1.620
1.614
1.607
1.601
1.595
1.588
1.576
1.014
0.995
1H-NMR (CDCl3, 400 MHz)
HO
O
A
1.006
0.988
1.922
0.939
2.179
3.138
10.0
9.5
9.0
8.5
8.0
7.5
7.0
6.5
6.0
5.5
5.0
4.5
4.0
3.5
3.0
2.5
2.0
1.5
1.0
0.5
0
f1 (ppm)

^{13}C-NMR ($CDCl_3$, 100 MHz)

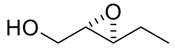

A

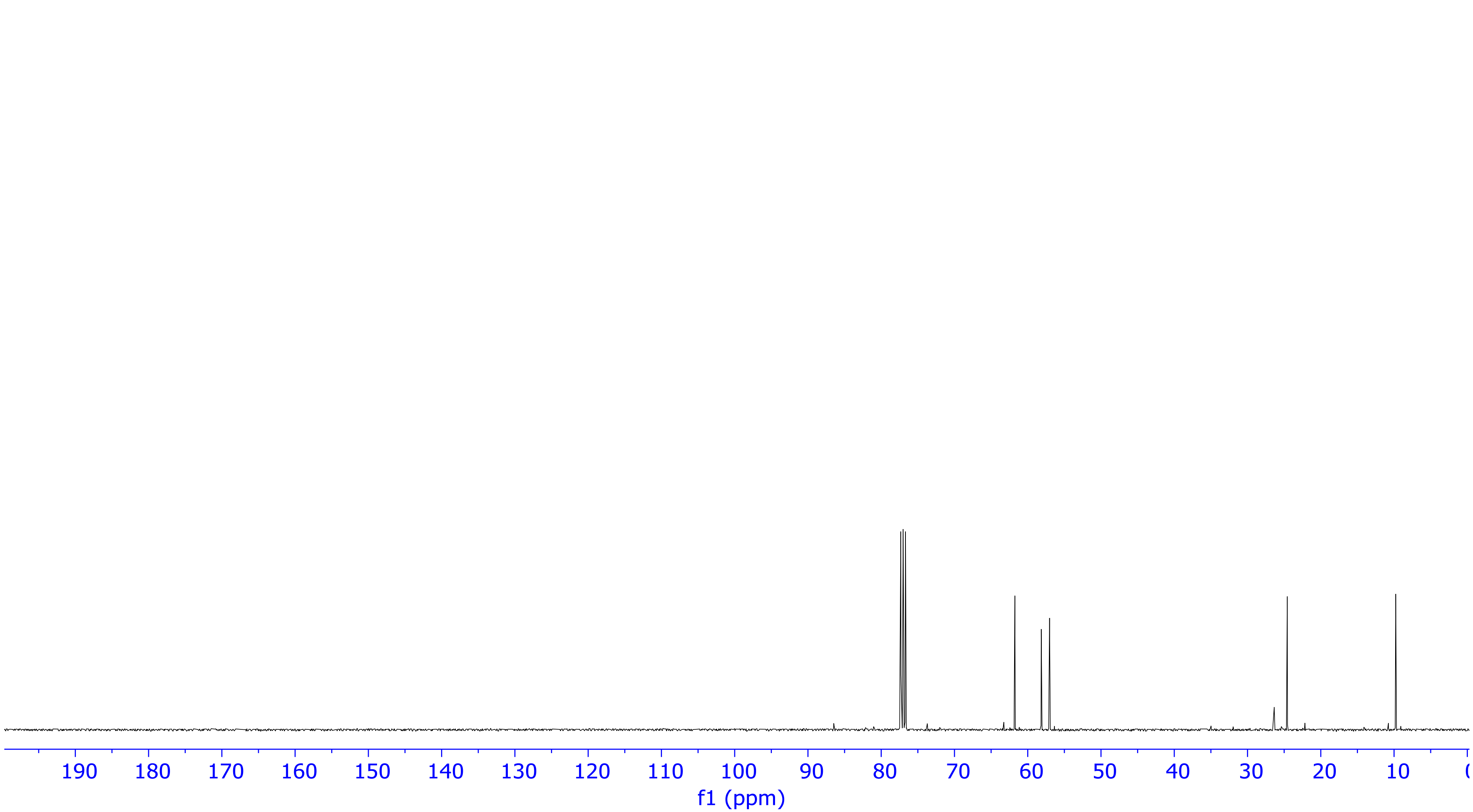

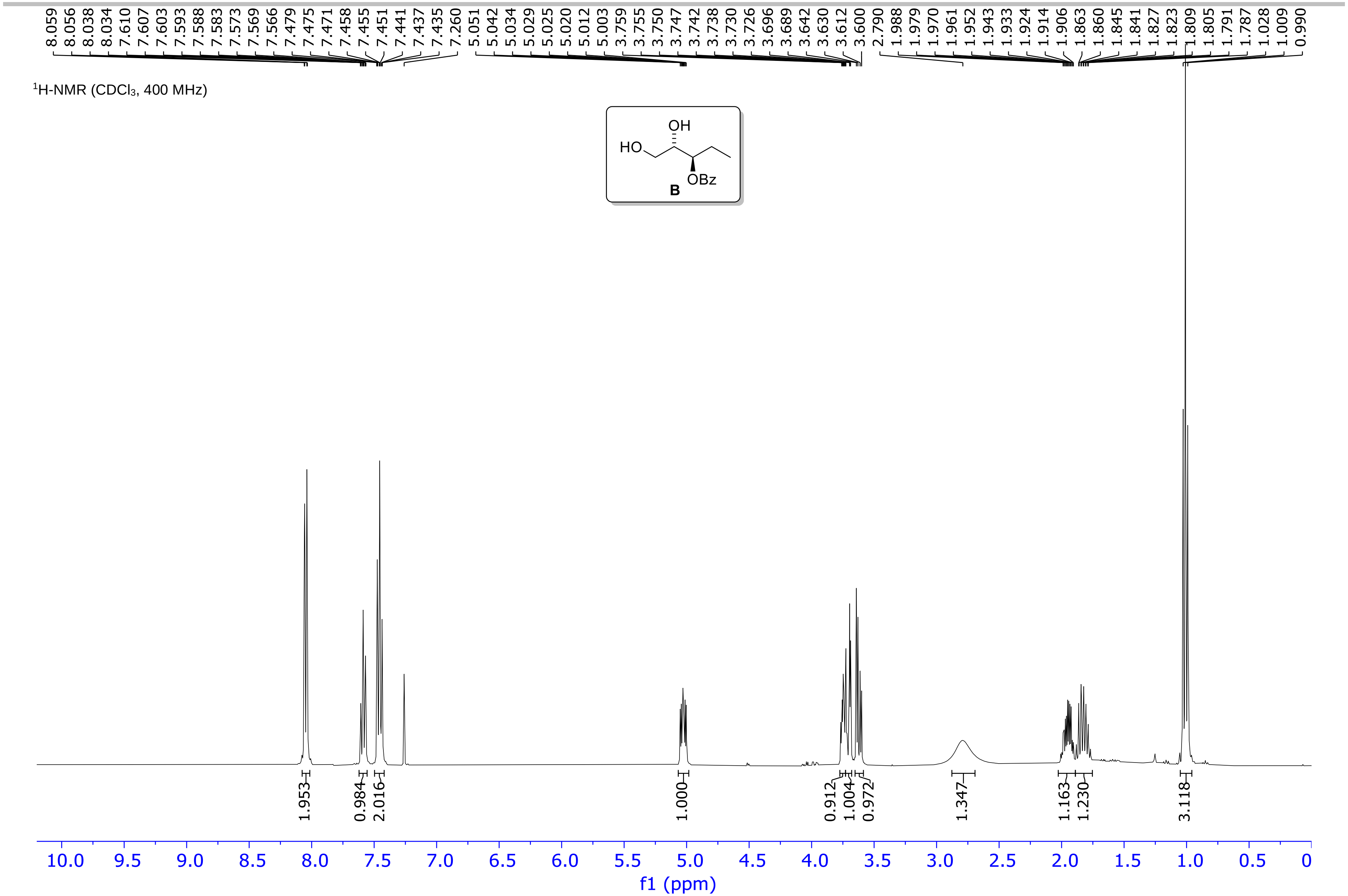
8.059
8.056
8.038
8.034
7.610
7.607
7.603
7.593
7.588
7.583
7.573
7.569
7.566
7.479
7.475
7.471
7.458
7.455
7.451
7.441
7.437
7.435
7.260
5.051
5.042
5.034
5.029
5.025
5.020
5.012
5.003
3.759
3.755
3.750
3.747
3.742
3.738
3.730
3.726
3.696
3.689
3.642
3.630
3.612
3.600
2.790
1.988
1.979
1.970
1.961
1.952
1.943
1.933
1.924
1.914
1.906
1.863
1.860
1.845
1.841
1.827
1.823
1.809
1.805
1.791
1.787
1.028
1.009
0.990
1H-NMR (CDCl3, 400 MHz)
OH
HO
OBz
B
1.953
0.984
2.016
1.000
0.912
1.004
0.972
1.347
1.163
1.230
3.118
10.0
9.5
9.0
8.5
8.0
7.5
7.0
6.5
6.0
5.5
5.0
4.5
4.0
3.5
3.0
2.5
2.0
1.5
1.0
0.5
0
f1 (ppm)

^{13}C-NMR (CDCl$_3$, 100 MHz)

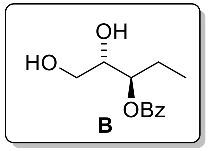

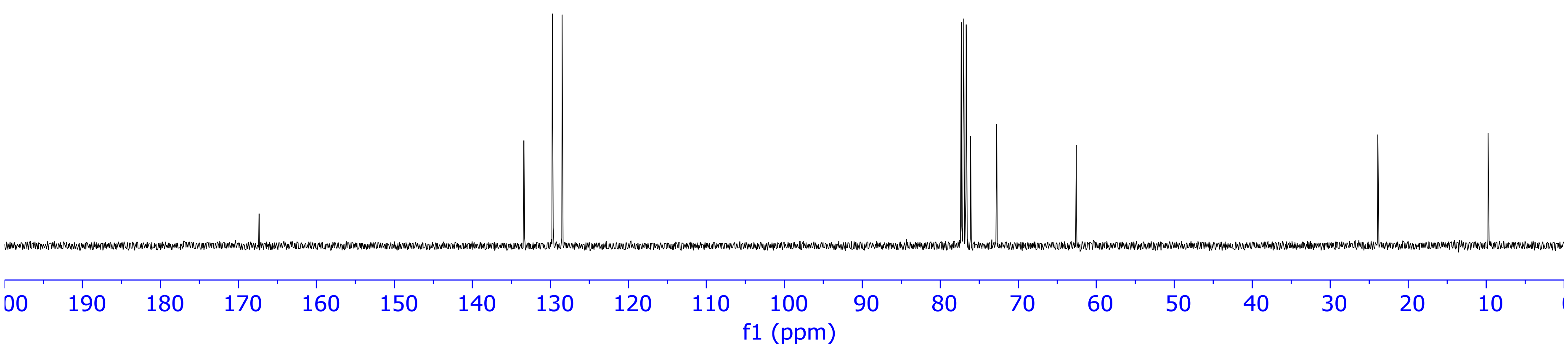

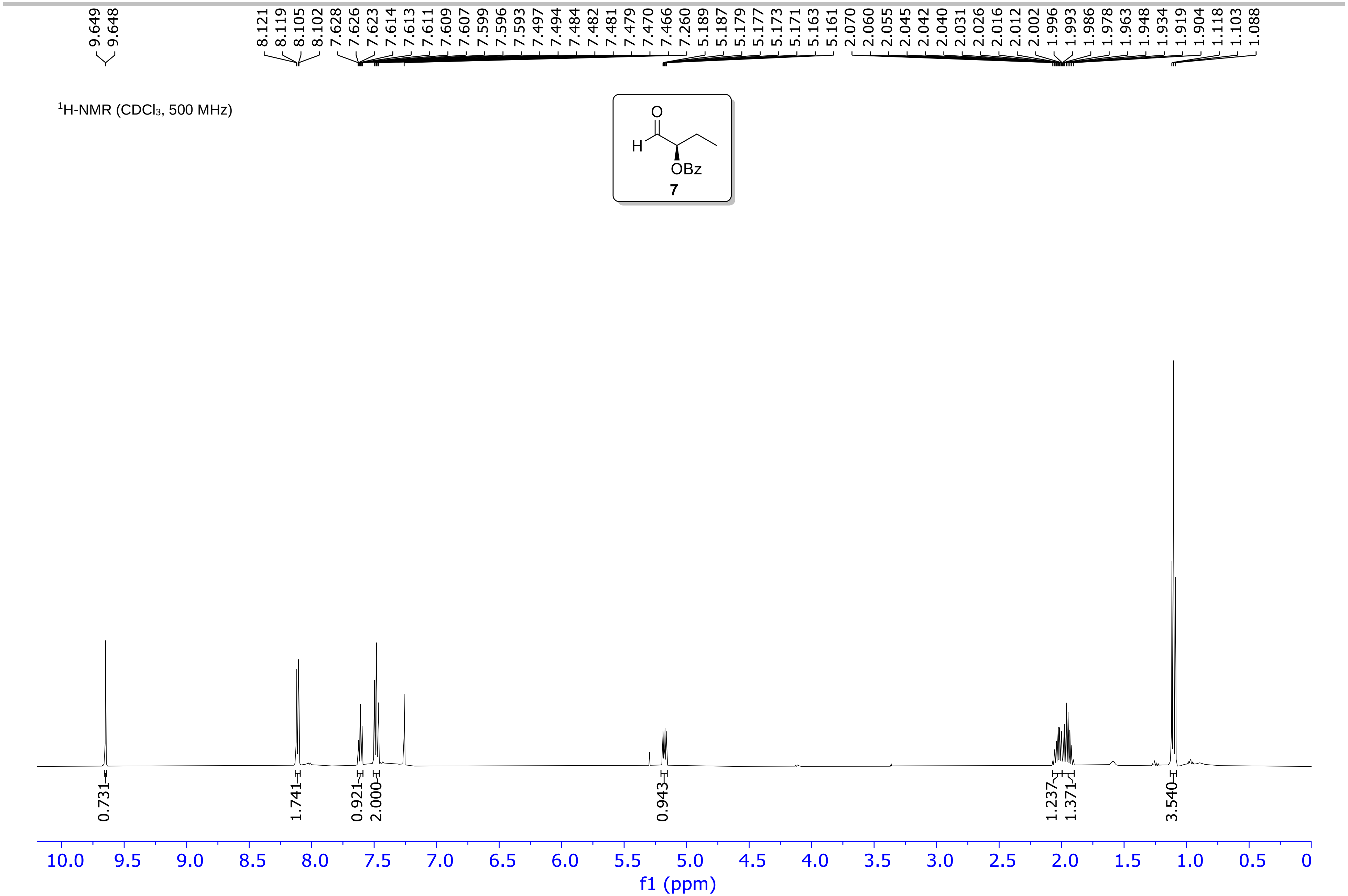
9.649
9.648
8.121
8.119
8.105
8.102
7.628
7.626
7.623
7.614
7.613
7.611
7.609
7.607
7.599
7.596
7.593
7.497
7.494
7.484
7.482
7.481
7.479
7.470
7.466
7.260
5.189
5.187
5.179
5.177
5.173
5.171
5.163
5.161
2.070
2.060
2.055
2.045
2.042
2.040
2.031
2.026
2.016
2.012
2.002
1.996
1.993
1.986
1.978
1.963
1.948
1.934
1.919
1.904
1.118
1.103
1.088
1H-NMR (CDCl3, 500 MHz)
O
H
OBz
7
0.731
1.741
0.921
2.000
0.943
1.237
1.371
3.540
10.0
9.5
9.0
8.5
8.0
7.5
7.0
6.5
6.0
5.5
5.0
4.5
4.0
3.5
3.0
2.5
2.0
1.5
1.0
0.5
0
f1 (ppm)

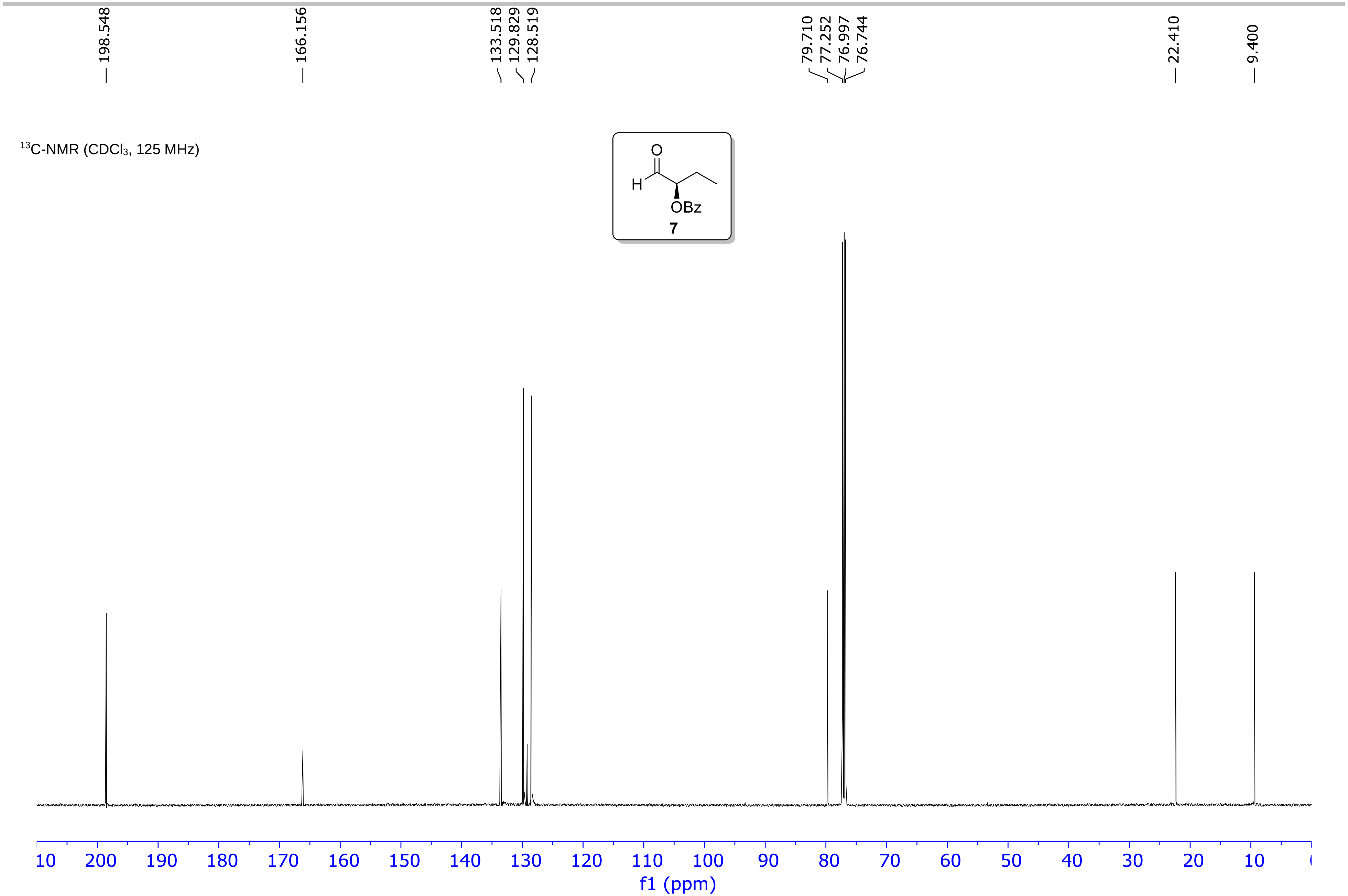
198.548
166.156
133.518
129.829
128.519
79.710
77.252
76.997
76.744
22.410
9.400
13C-NMR (CDCl3, 125 MHz)
O
H
OBz
7
f1 (ppm)

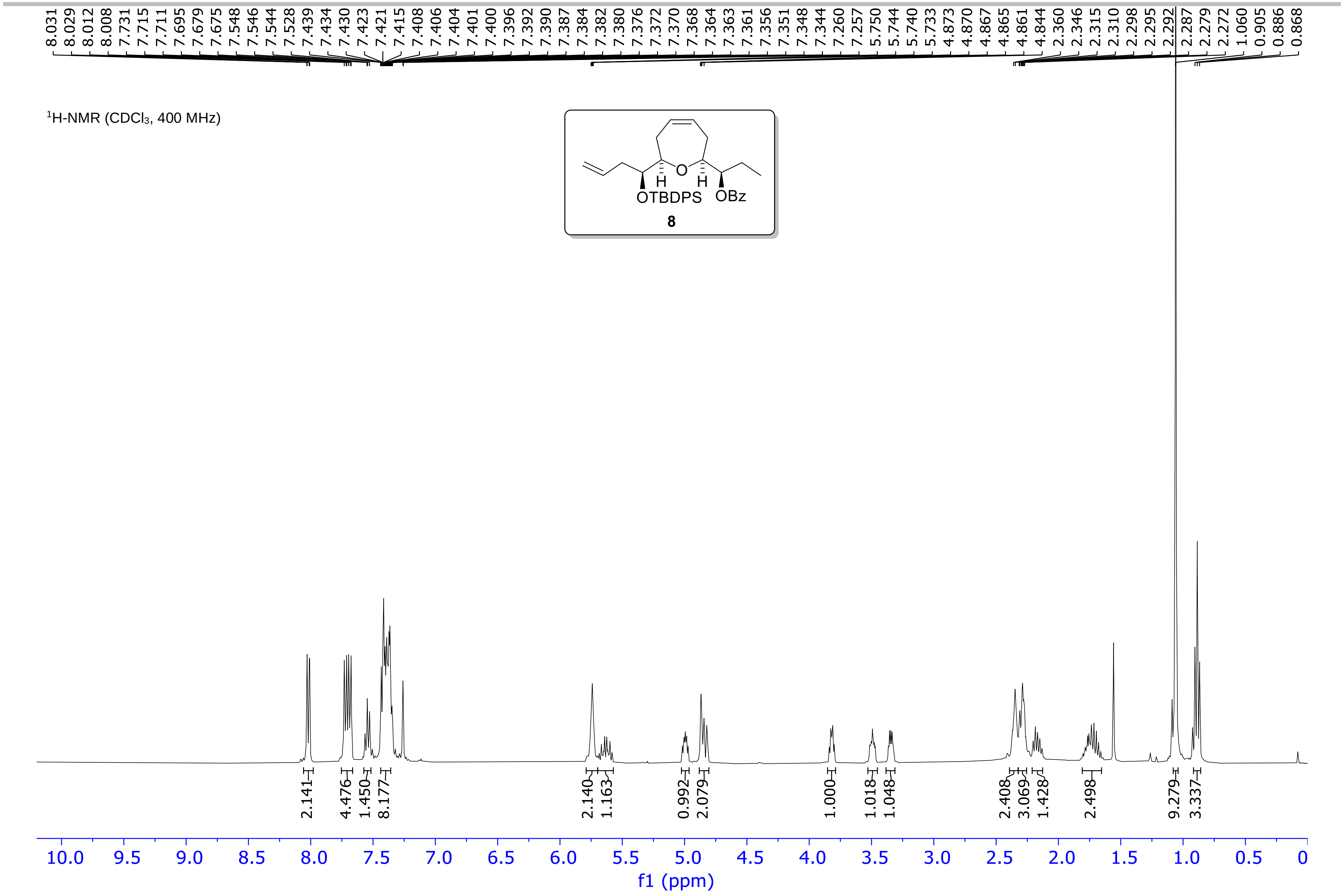

8.031 8.029 8.012 8.008 7.731 7.715 7.711 7.695 7.679 7.675 7.548 7.546 7.544 7.528 7.439 7.434 7.430 7.423 7.421 7.415 7.408 7.406 7.404 7.401 7.400 7.396 7.392 7.390 7.387 7.384 7.382 7.380 7.376 7.372 7.370 7.368 7.364 7.363 7.361 7.356 7.351 7.348 7.344 7.260 7.257 5.750 5.744 5.740 5.733 4.873 4.870 4.867 4.865 4.861 4.844 2.360 2.346 2.315 2.310 2.298 2.295 2.292 2.287 2.279 2.272 1.060 0.905 0.886 0.868
^{1}H-NMR (CDCl$_3$, 400 MHz)
H
O
H
OTBDPS
OBz
8
2.141 4.476 1.450 8.177 2.140 1.163 0.992 2.079 1.000 1.018 1.048 2.408 3.069 1.428 2.498 9.279 3.337
10.0 9.5 9.0 8.5 8.0 7.5 7.0 6.5 6.0 5.5 5.0 4.5 4.0 3.5 3.0 2.5 2.0 1.5 1.0 0.5 0
f1 (ppm)

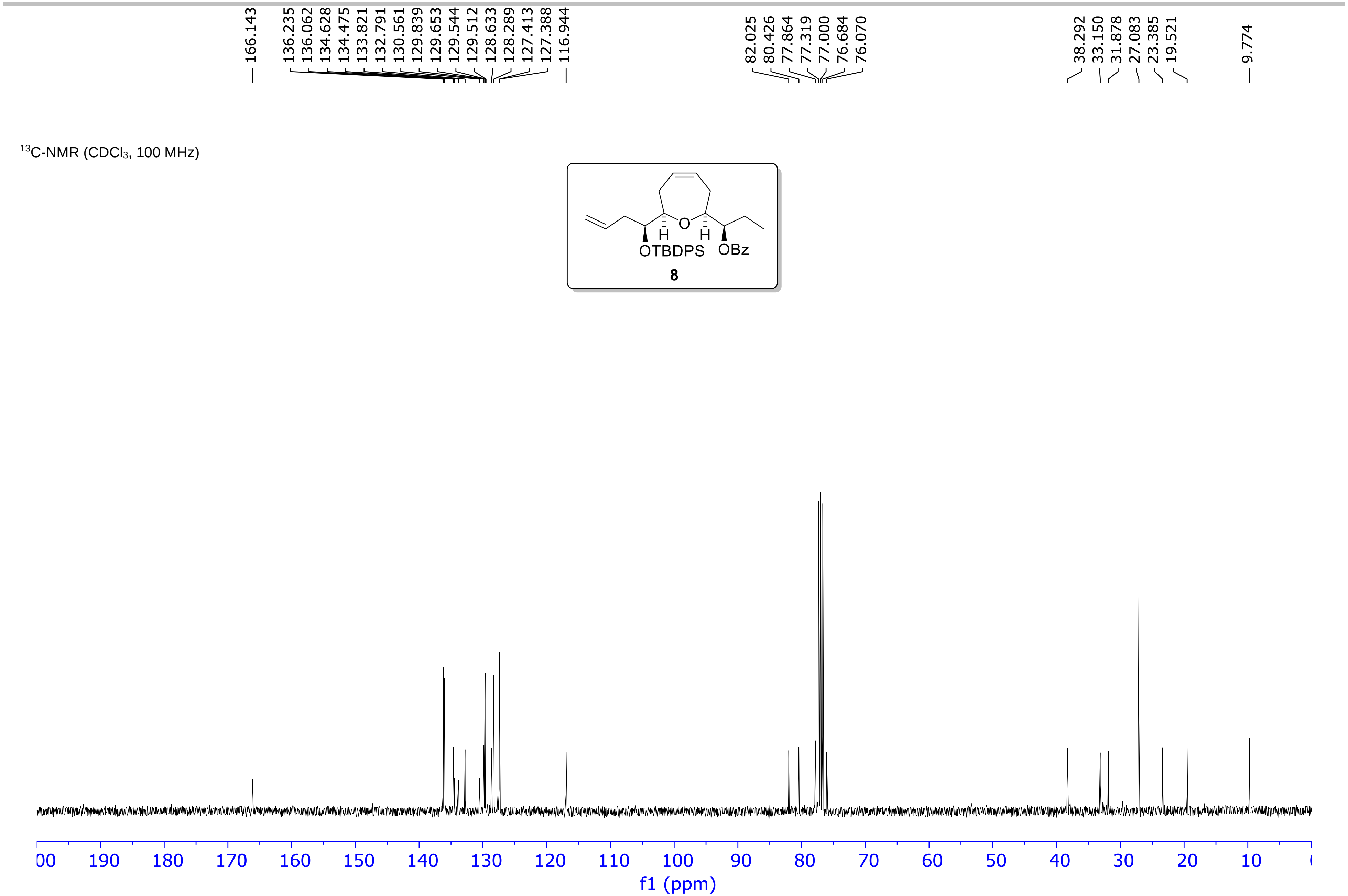
13C-NMR (CDCl3, 100 MHz)
166.143
136.235
136.062
134.628
134.475
133.821
132.791
130.561
129.839
129.653
129.544
129.512
128.633
128.289
127.413
127.388
116.944
82.025
80.426
77.864
77.319
77.000
76.684
76.070
38.292
33.150
31.878
27.083
23.385
19.521
9.774
O
H
H
OTBDPS
OBz
8
00
190
180
170
160
150
140
130
120
110
100
90
80
70
60
50
40
30
20
10
f1 (ppm)

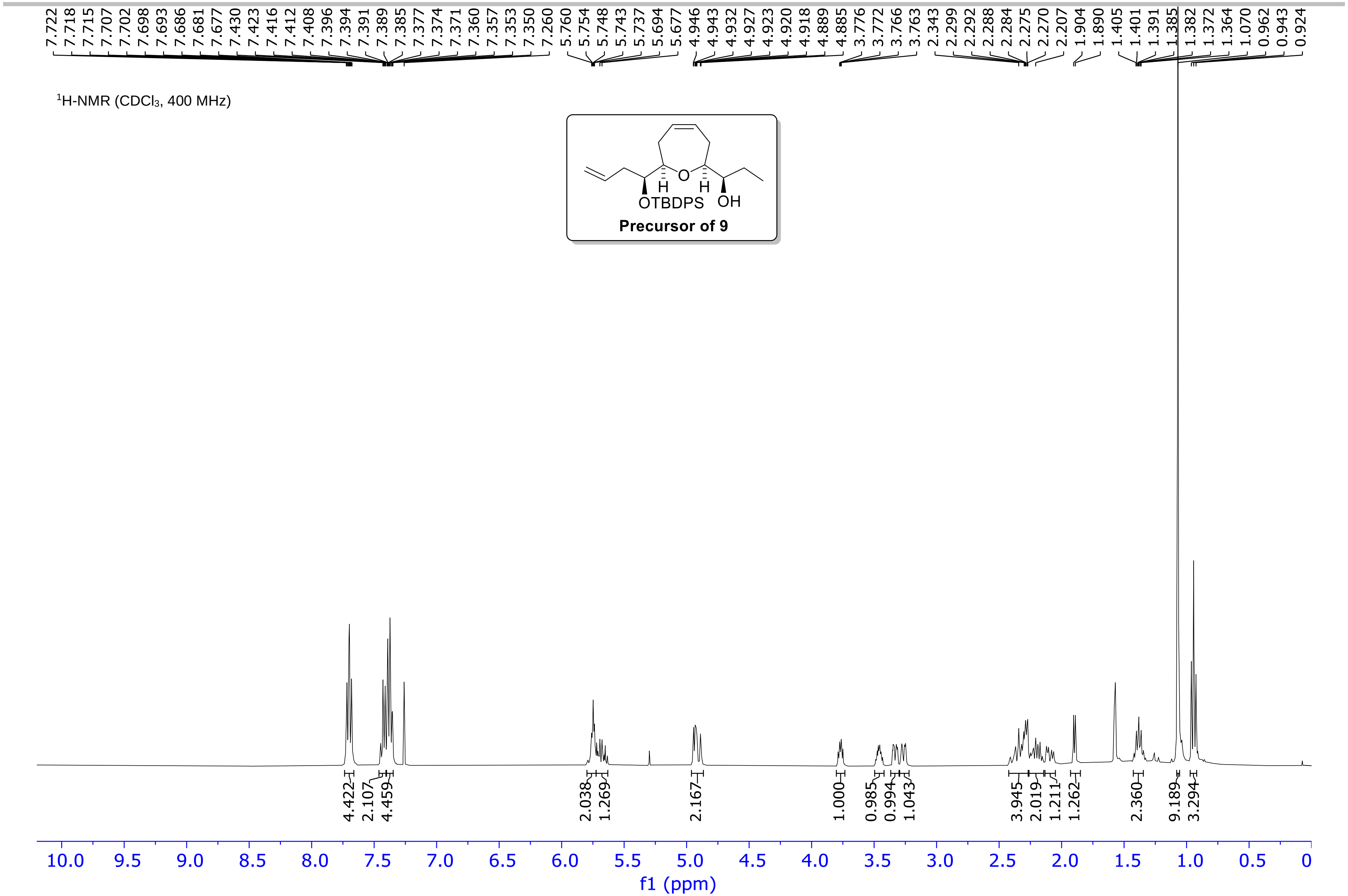
7.722 7.718 7.715 7.707 7.702 7.698 7.693 7.686 7.681 7.677 7.430 7.423 7.416 7.412 7.408 7.396 7.394 7.391 7.389 7.385 7.377 7.374 7.371 7.360 7.357 7.353 7.350 7.260 5.760 5.754 5.748 5.743 5.737 5.694 5.677 4.946 4.943 4.932 4.927 4.923 4.920 4.918 4.889 4.885 3.776 3.772 3.766 3.763 2.343 2.299 2.292 2.288 2.284 2.275 2.270 2.207 1.904 1.890 1.405 1.401 1.391 1.385 1.382 1.372 1.364 1.070 0.962 0.943 0.924
^{1}H-NMR (CDCl$_3$, 400 MHz)
H
O
H
OTBDPS
OH
Precursor of 9
4.422 2.107 4.459 2.038 1.269 2.167 1.000 0.985 0.994 1.043 3.945 2.019 1.211 1.262 2.360 9.189 3.294
10.0 9.5 9.0 8.5 8.0 7.5 7.0 6.5 6.0 5.5 5.0 4.5 4.0 3.5 3.0 2.5 2.0 1.5 1.0 0.5 0
f1 (ppm)

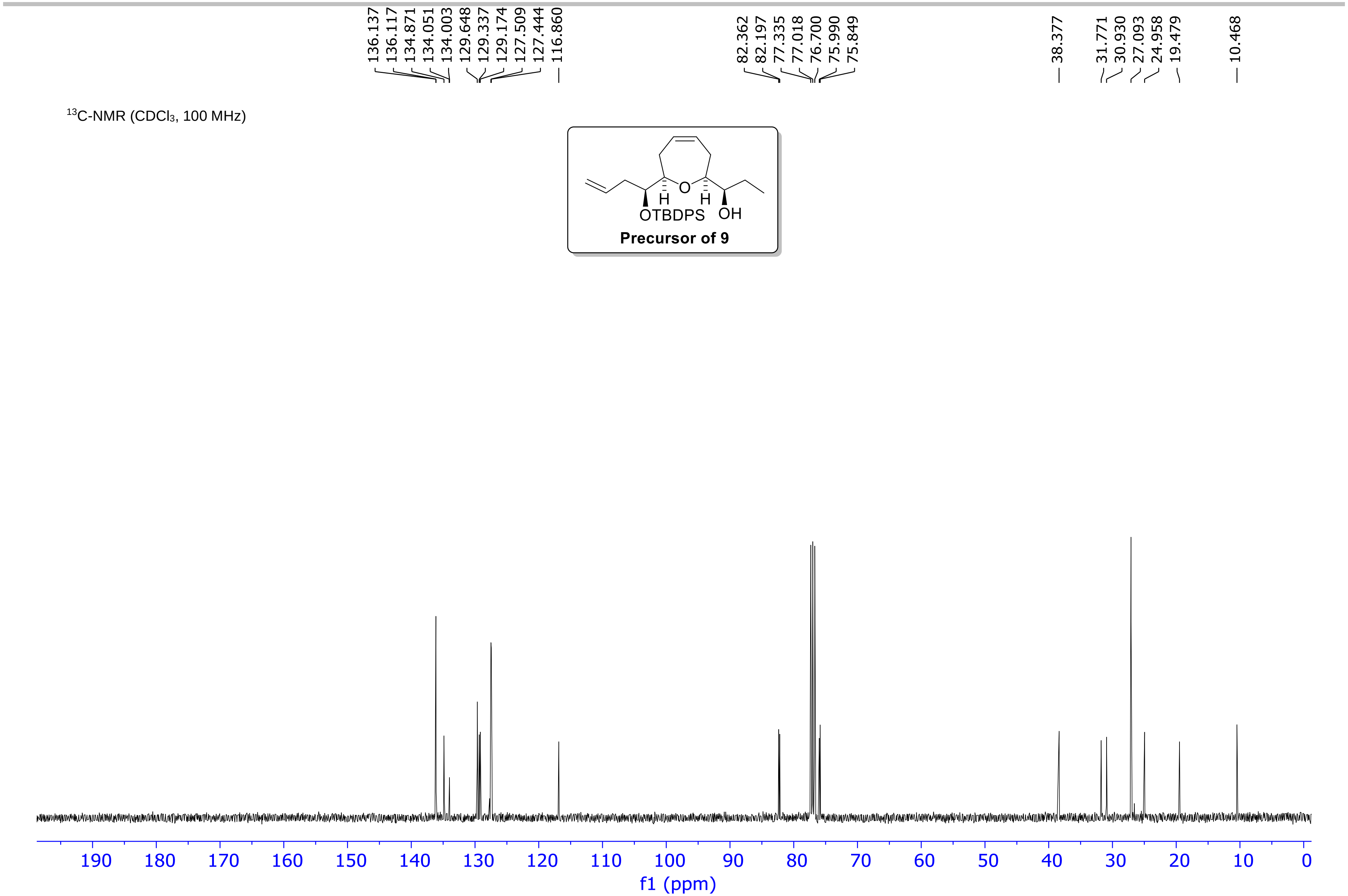
136.137
136.117
134.871
134.051
134.003
129.648
129.337
129.174
127.509
127.444
116.860
82.362
82.197
77.335
77.018
76.700
75.990
75.849
38.377
31.771
30.930
27.093
24.958
19.479
10.468
^{13}C-NMR (CDCl$_3$, 100 MHz)
H
O
H
OTBDPS
OH
Precursor of 9
190 180 170 160 150 140 130 120 110 100 90 80 70 60 50 40 30 20 10 0
f1 (ppm)

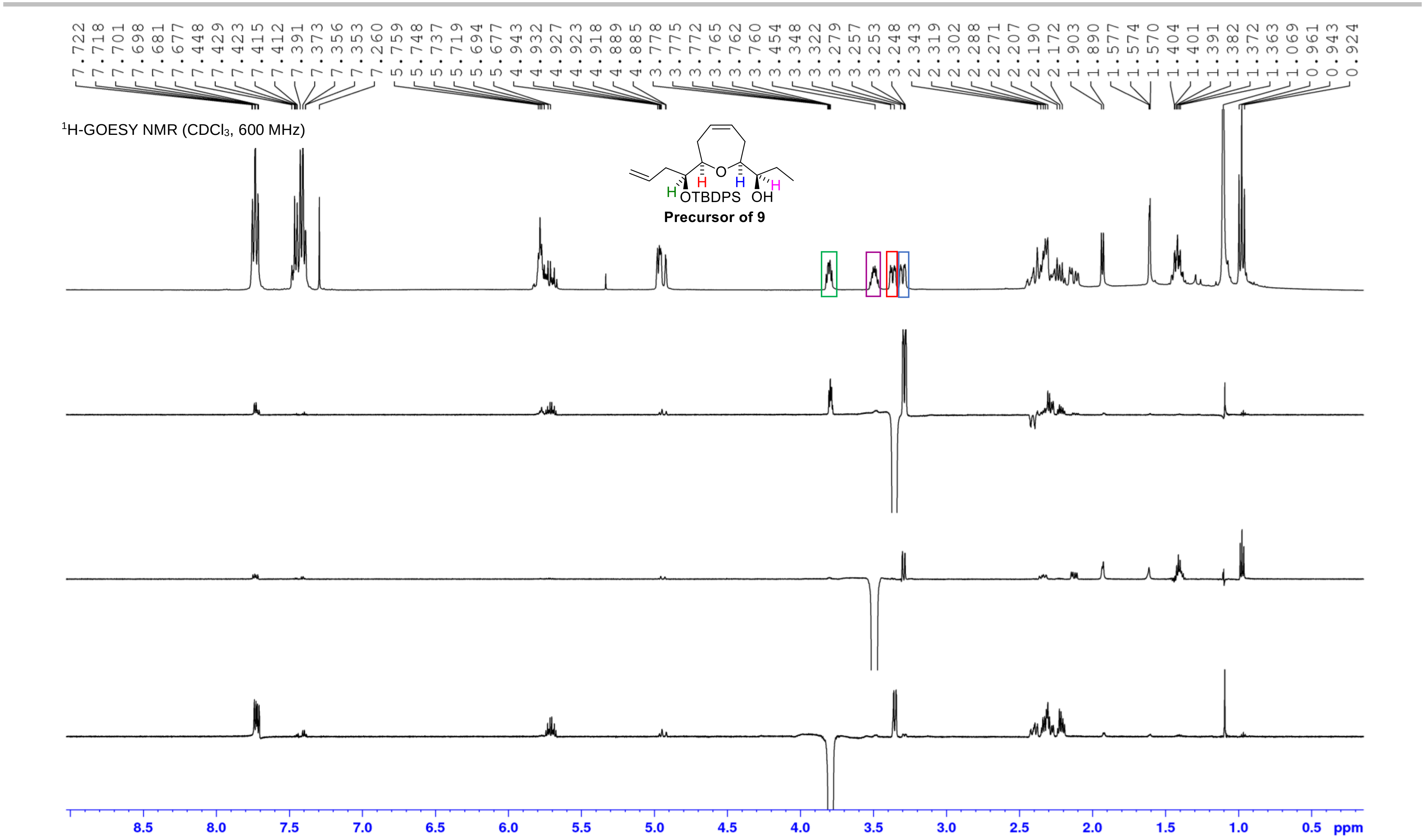

^{1}H-GOESY NMR ($CDCl_3$, 600 MHz)
H OTBDPS O H H OH H
Precursor of 9
8.5 8.0 7.5 7.0 6.5 6.0 5.5 5.0 4.5 4.0 3.5 3.0 2.5 2.0 1.5 1.0 0.5 ppm

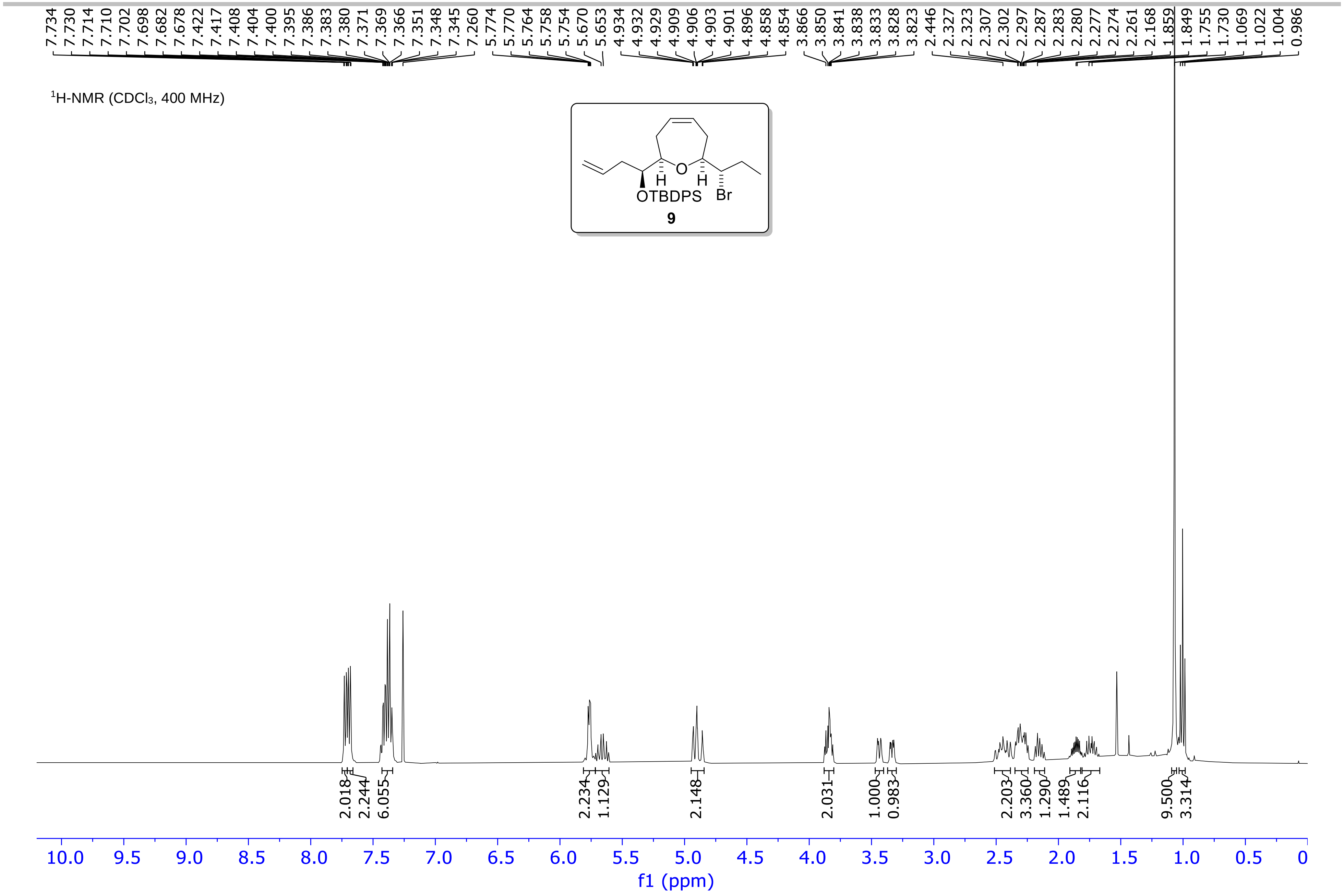

7.734 7.730 7.714 7.710 7.702 7.698 7.682 7.678 7.422 7.417 7.408 7.404 7.400 7.395 7.386 7.383 7.380 7.371 7.369 7.366 7.351 7.348 7.345 7.260 5.774 5.770 5.764 5.758 5.754 5.670 5.653 4.934 4.932 4.929 4.909 4.906 4.903 4.901 4.896 4.858 4.854 3.866 3.850 3.841 3.838 3.833 3.828 3.823 2.446 2.327 2.323 2.307 2.302 2.297 2.287 2.283 2.280 2.277 2.274 2.261 2.168 1.859 1.849 1.755 1.730 1.069 1.022 1.004 0.986
¹H-NMR (CDCl₃, 400 MHz)
OTBDPS
Br
O
H
H
9
2.018 2.244 6.055 2.234 1.129 2.148 2.031 1.000 0.983 2.203 3.360 1.290 1.489 2.116 9.500 3.314
10.0 9.5 9.0 8.5 8.0 7.5 7.0 6.5 6.0 5.5 5.0 4.5 4.0 3.5 3.0 2.5 2.0 1.5 1.0 0.5 0
f1 (ppm)

^{13}C-NMR ($CDCl_3$, 100 MHz)

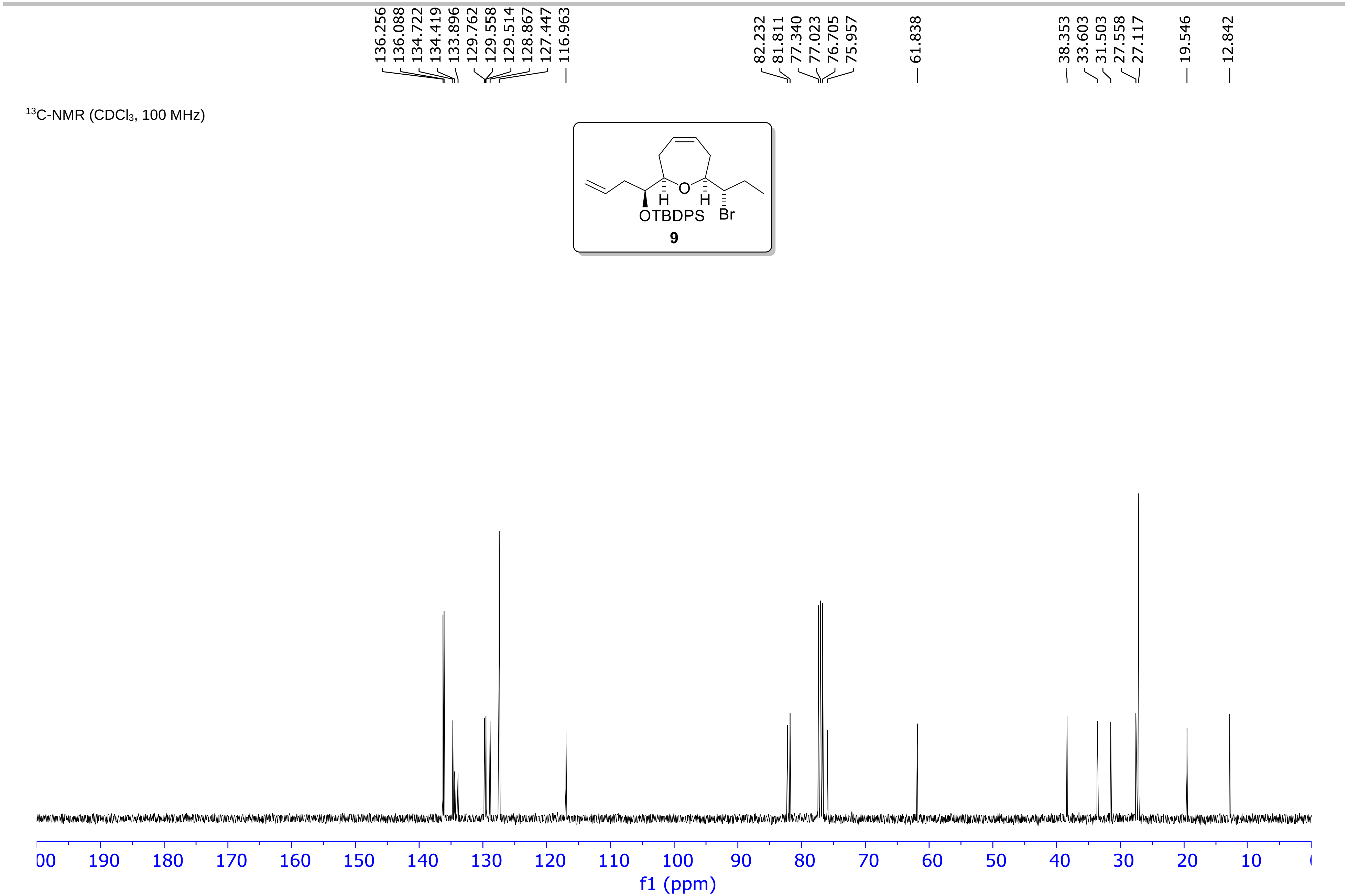

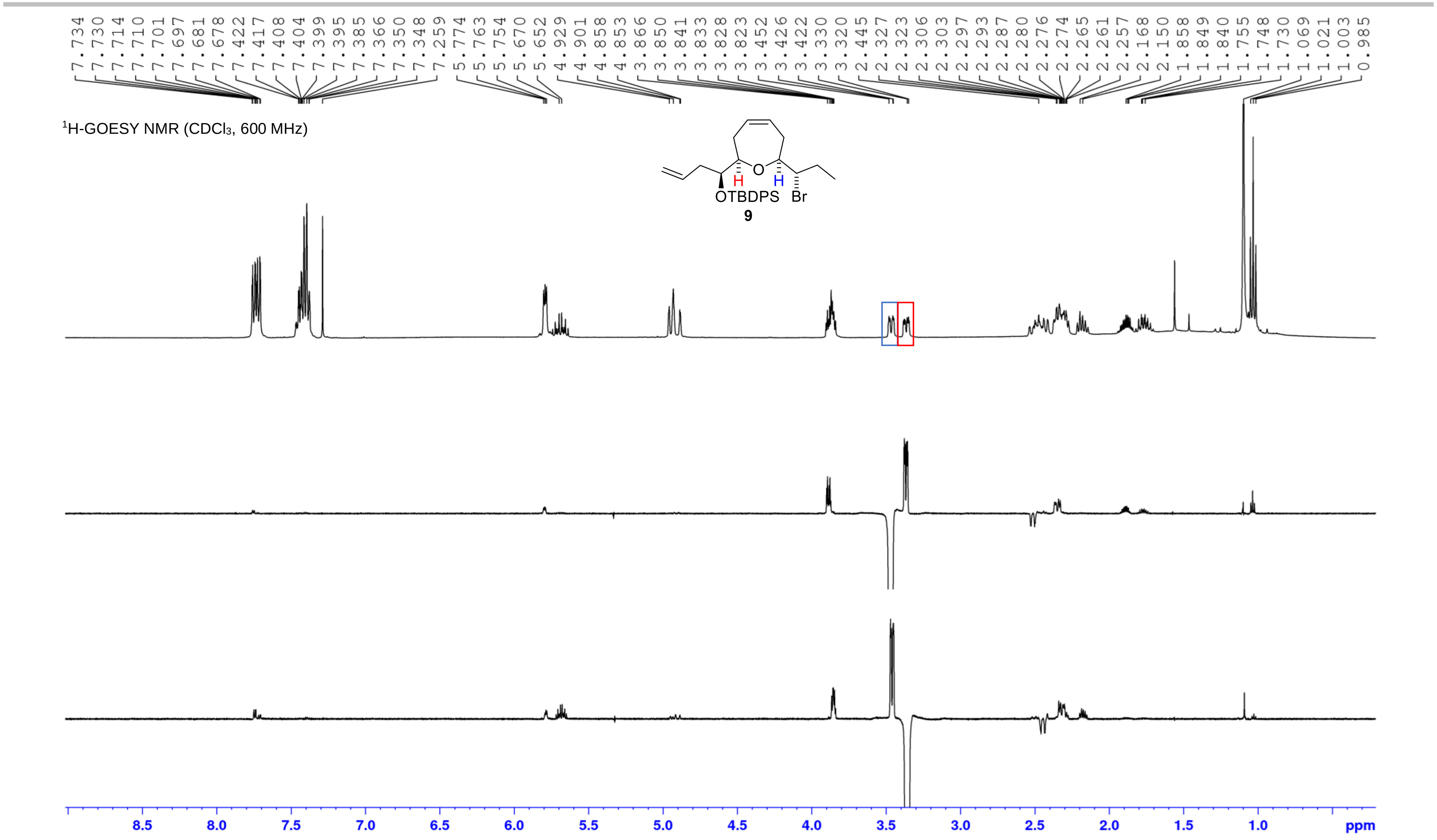
7.734
7.730
7.714
7.710
7.701
7.697
7.681
7.678
7.422
7.417
7.408
7.404
7.399
7.395
7.385
7.366
7.350
7.348
7.259
5.774
5.763
5.754
5.670
5.652
4.929
4.901
4.858
4.853
3.866
3.850
3.841
3.833
3.828
3.823
3.452
3.426
3.422
3.330
3.320
2.445
2.327
2.323
2.306
2.303
2.297
2.293
2.287
2.280
2.276
2.274
2.265
2.261
2.257
2.168
2.150
1.858
1.849
1.840
1.755
1.748
1.730
1.069
1.021
1.003
0.985
1H-GOESY NMR (CDCl3, 600 MHz)
H
O
H
OTBDPS
Br
9
8.5
8.0
7.5
7.0
6.5
6.0
5.5
5.0
4.5
4.0
3.5
3.0
2.5
2.0
1.5
1.0
ppm

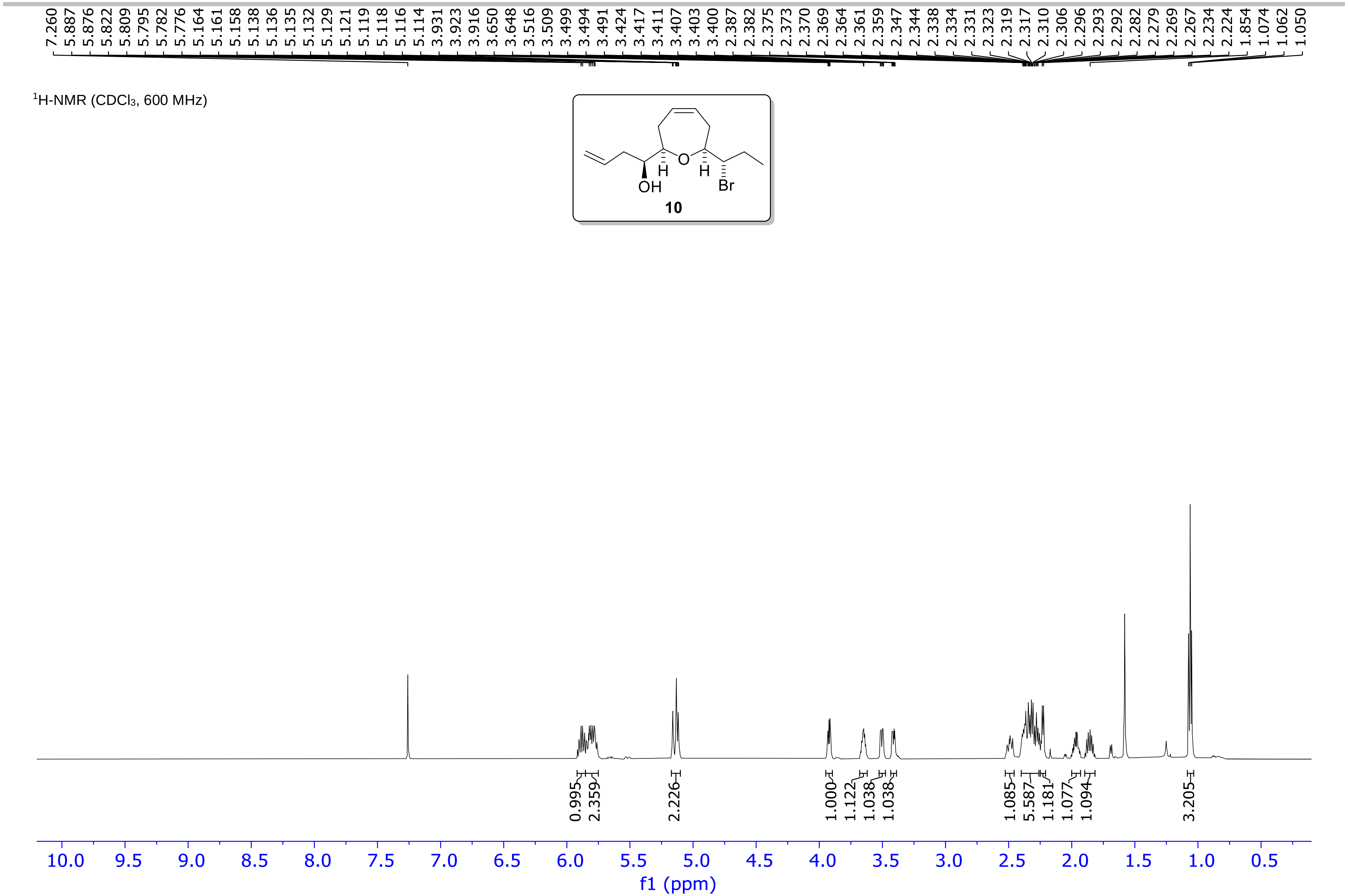

7.260 5.887 5.876 5.822 5.809 5.795 5.782 5.776 5.164 5.161 5.158 5.138 5.136 5.135 5.132 5.129 5.121 5.119 5.118 5.116 5.114 3.931 3.923 3.916 3.650 3.648 3.516 3.509 3.499 3.494 3.491 3.424 3.417 3.411 3.407 3.403 3.400 2.387 2.382 2.375 2.373 2.370 2.369 2.364 2.361 2.359 2.347 2.344 2.338 2.334 2.331 2.323 2.319 2.317 2.310 2.306 2.296 2.293 2.292 2.282 2.279 2.269 2.267 2.234 2.224 1.854 1.074 1.062 1.050
1H-NMR (CDCl3, 600 MHz)
OH
H
O
H
Br
10
0.995
2.359
2.226
1.000
1.122
1.038
1.038
1.085
5.587
1.181
1.077
1.094
3.205
10.0 9.5 9.0 8.5 8.0 7.5 7.0 6.5 6.0 5.5 5.0 4.5 4.0 3.5 3.0 2.5 2.0 1.5 1.0 0.5
f1 (ppm)

^{13}C-NMR ($CDCl_3$, 100 MHz)

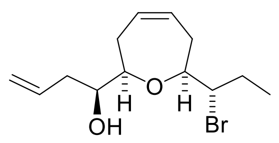

10

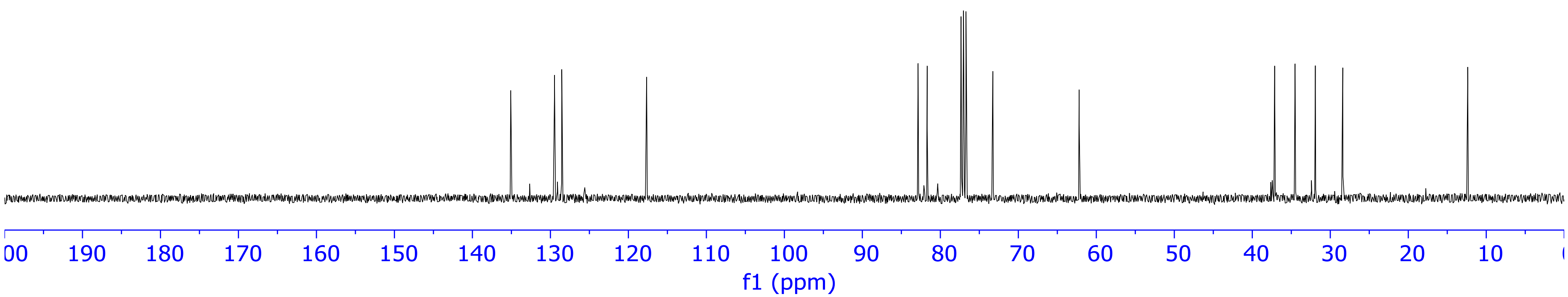

^{1}H-GOESY NMR ($CDCl_3$, 600 MHz)

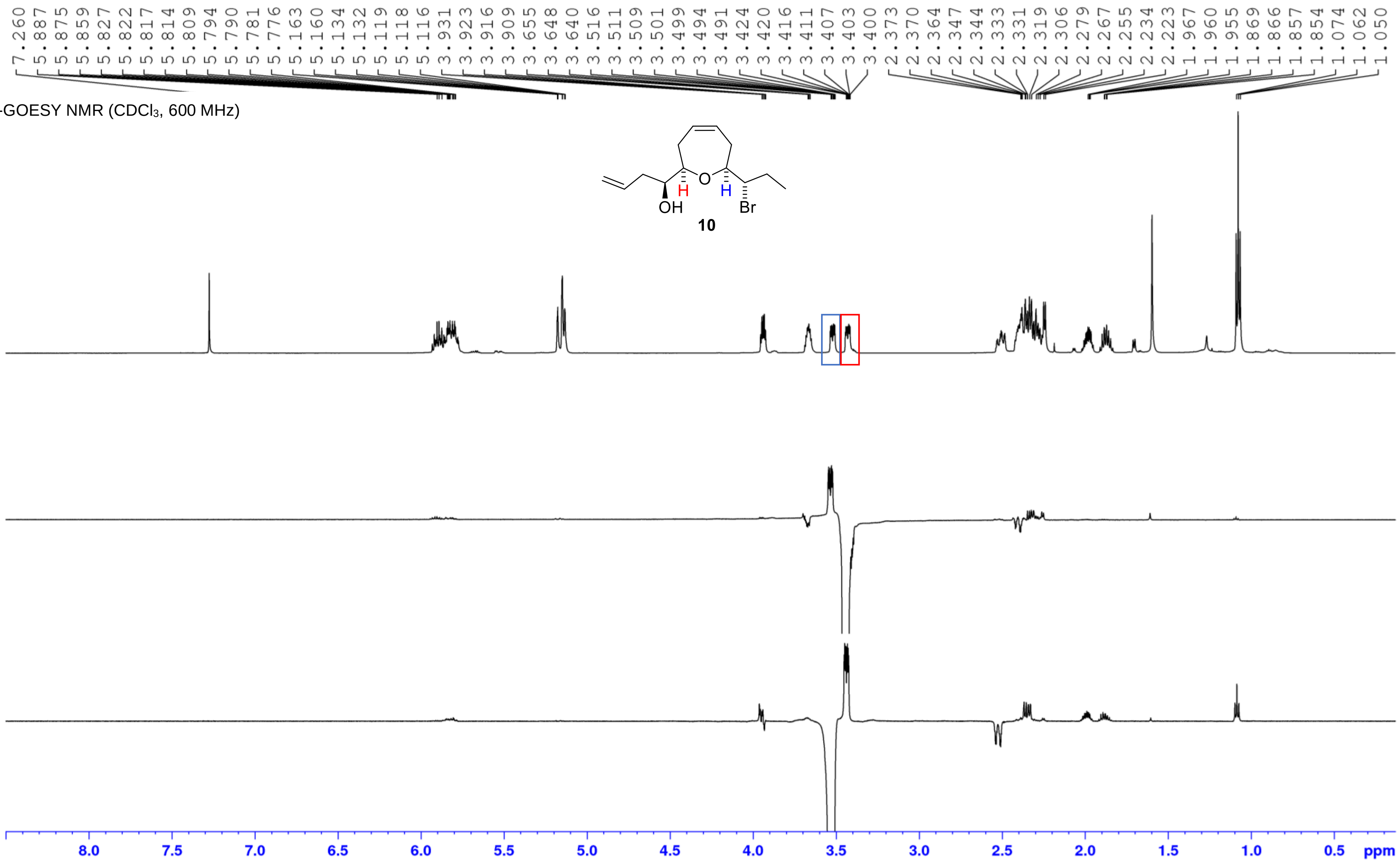

^{1}H-NMR (CDCl3, 400 MHz)

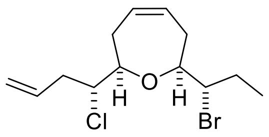

11

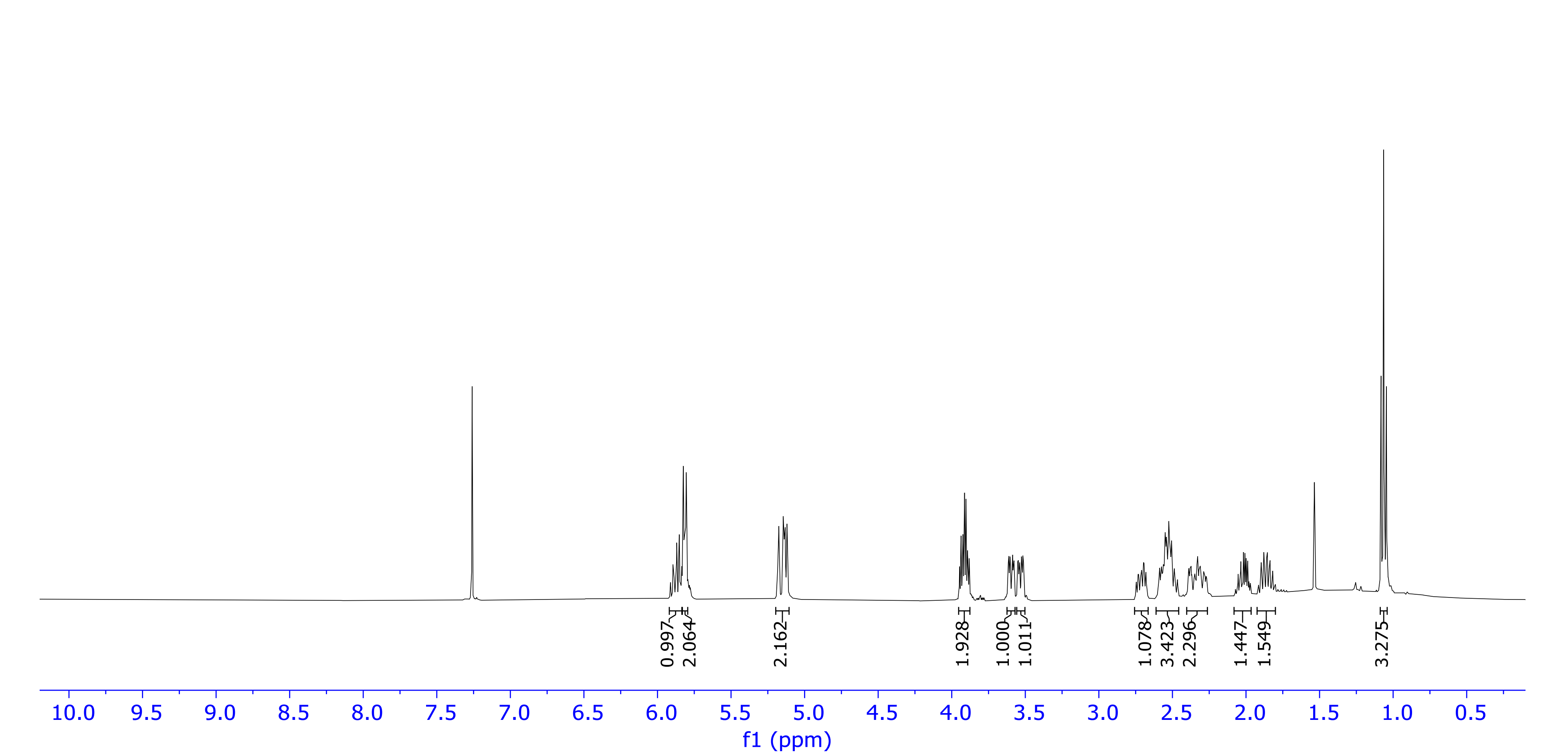

^{13}C-NMR ($CDCl_3$, 100 MHz)

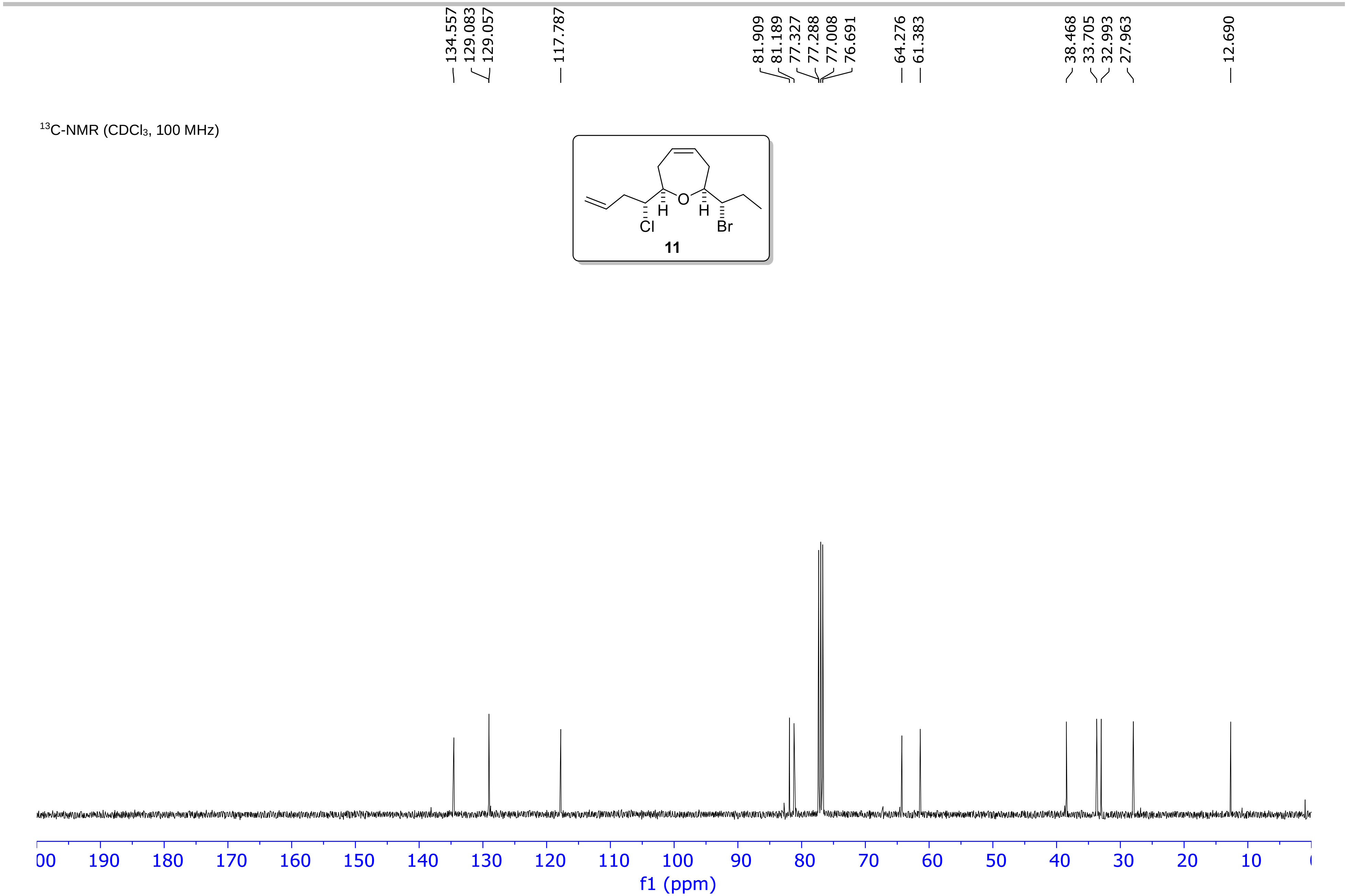

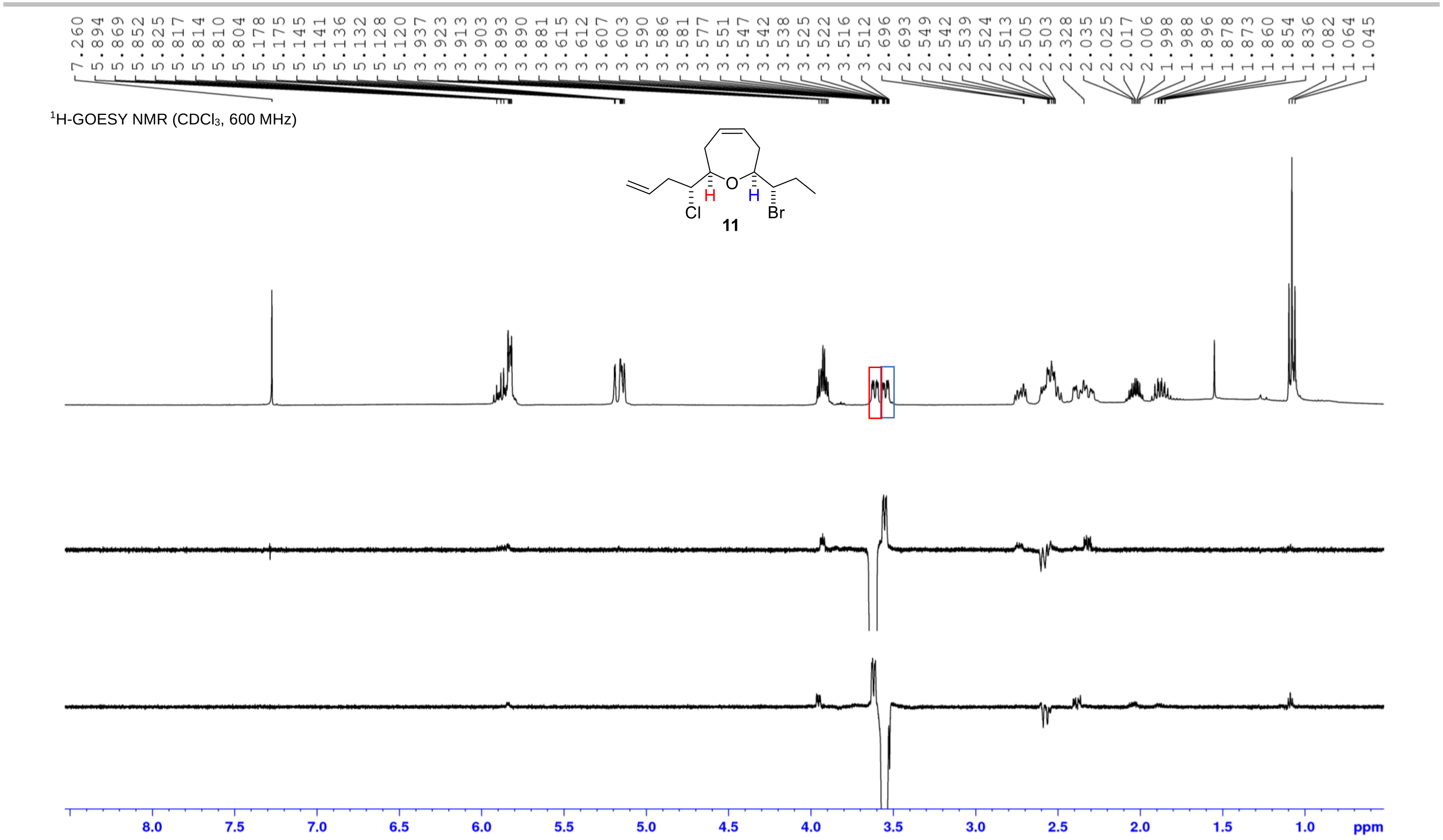
7.260 5.894 5.869 5.852 5.825 5.817 5.814 5.810 5.804 5.178 5.175 5.145 5.141 5.136 5.132 5.128 5.120 3.937 3.923 3.913 3.903 3.893 3.890 3.881 3.615 3.612 3.607 3.603 3.590 3.586 3.581 3.577 3.551 3.547 3.542 3.538 3.525 3.522 3.516 3.512 2.696 2.693 2.549 2.542 2.539 2.524 2.513 2.505 2.503 2.328 2.035 2.025 2.017 2.006 1.998 1.988 1.896 1.878 1.873 1.860 1.854 1.836 1.082 1.064 1.045
1H-GOESY NMR (CDCl3, 600 MHz)
O
H
H
Cl
Br
11
8.0 7.5 7.0 6.5 6.0 5.5 5.0 4.5 4.0 3.5 3.0 2.5 2.0 1.5 1.0 ppm

^{1}H-NMR ($CDCl_3$, 500 MHz)

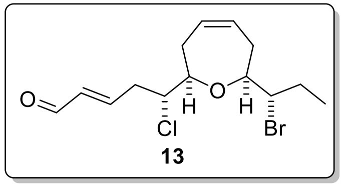

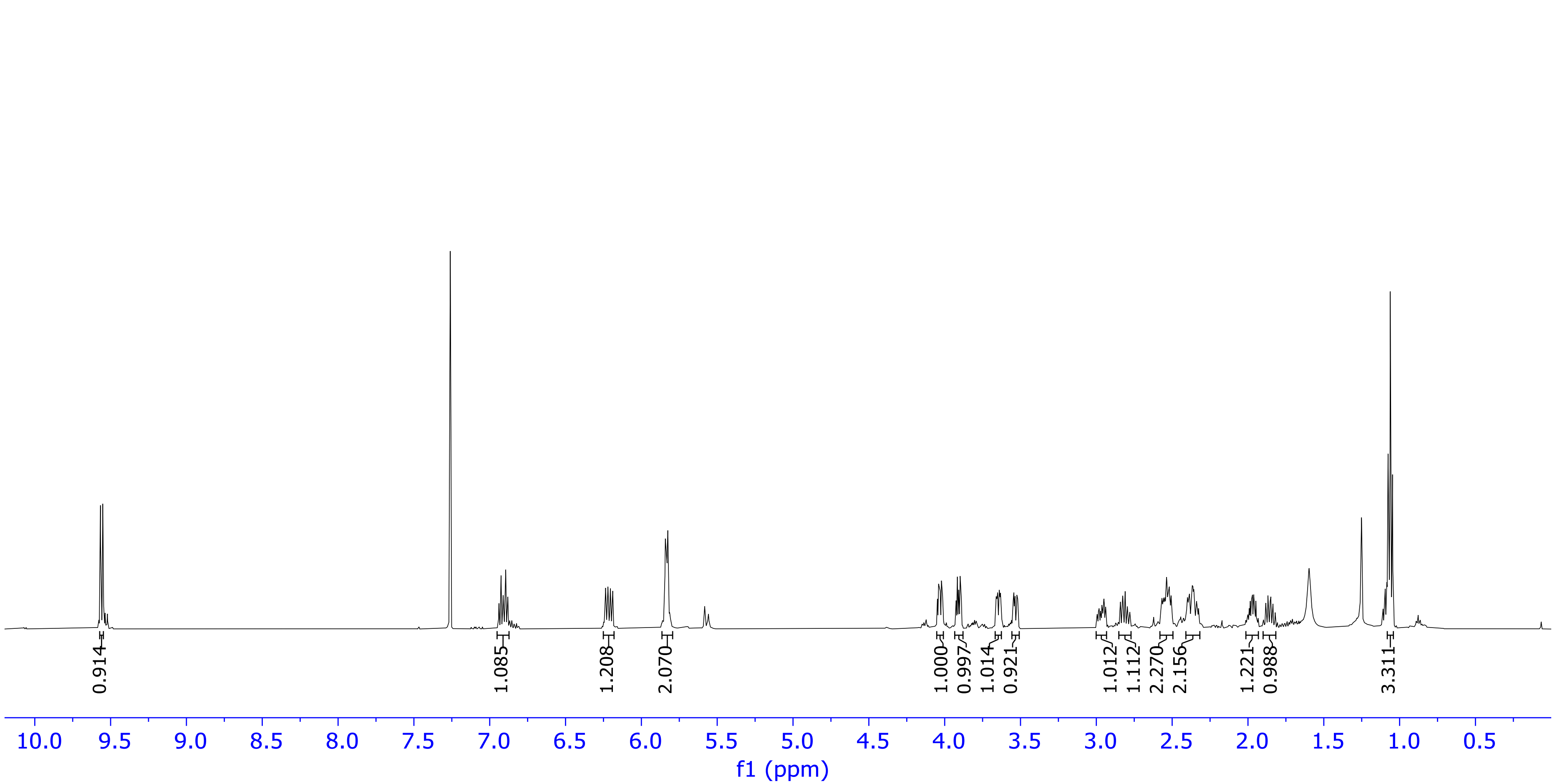

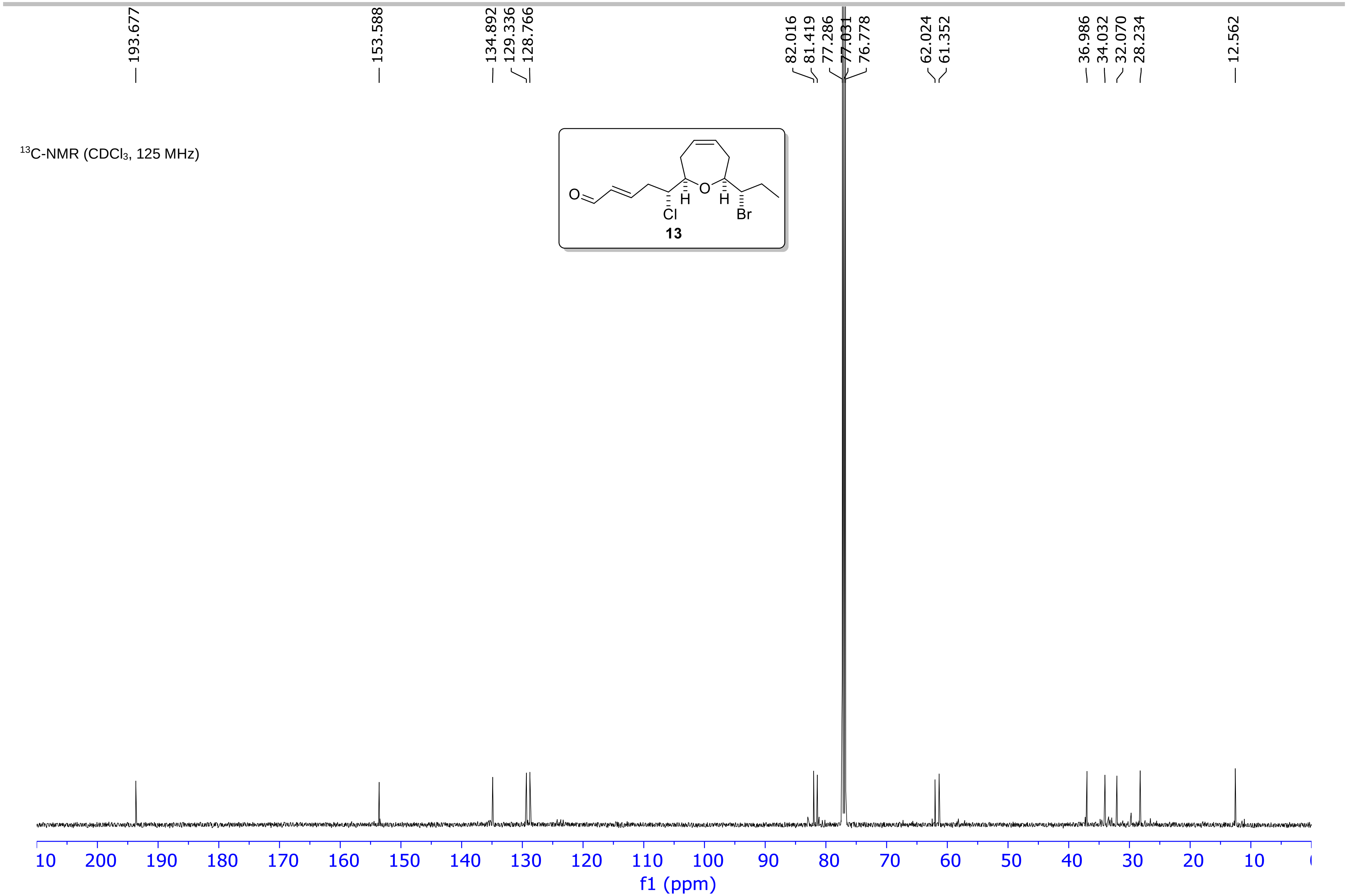

193.677
153.588
134.892
129.336
128.766
82.016
81.419
77.286
77.031
76.778
62.024
61.352
36.986
34.032
32.070
28.234
12.562
^{13}C-NMR ($CDCl_3$, 125 MHz)
O
Cl
H
O
H
Br
13
f1 (ppm)

^{1}H-NMR ($CDCl_3$, 500 MHz)

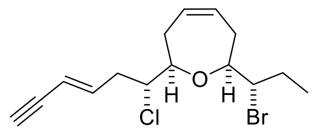

(+)-isolaurepinnacin (**1**)

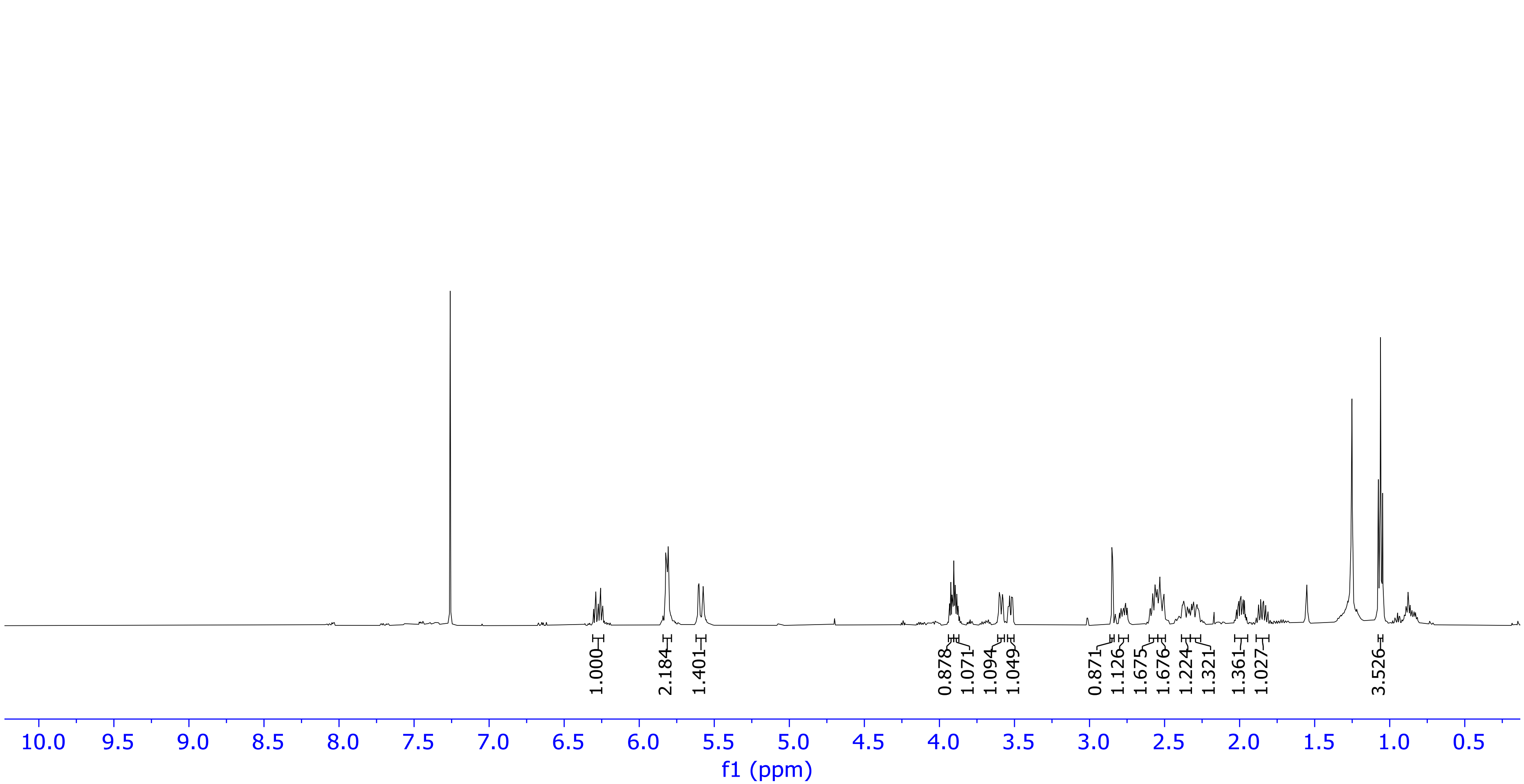

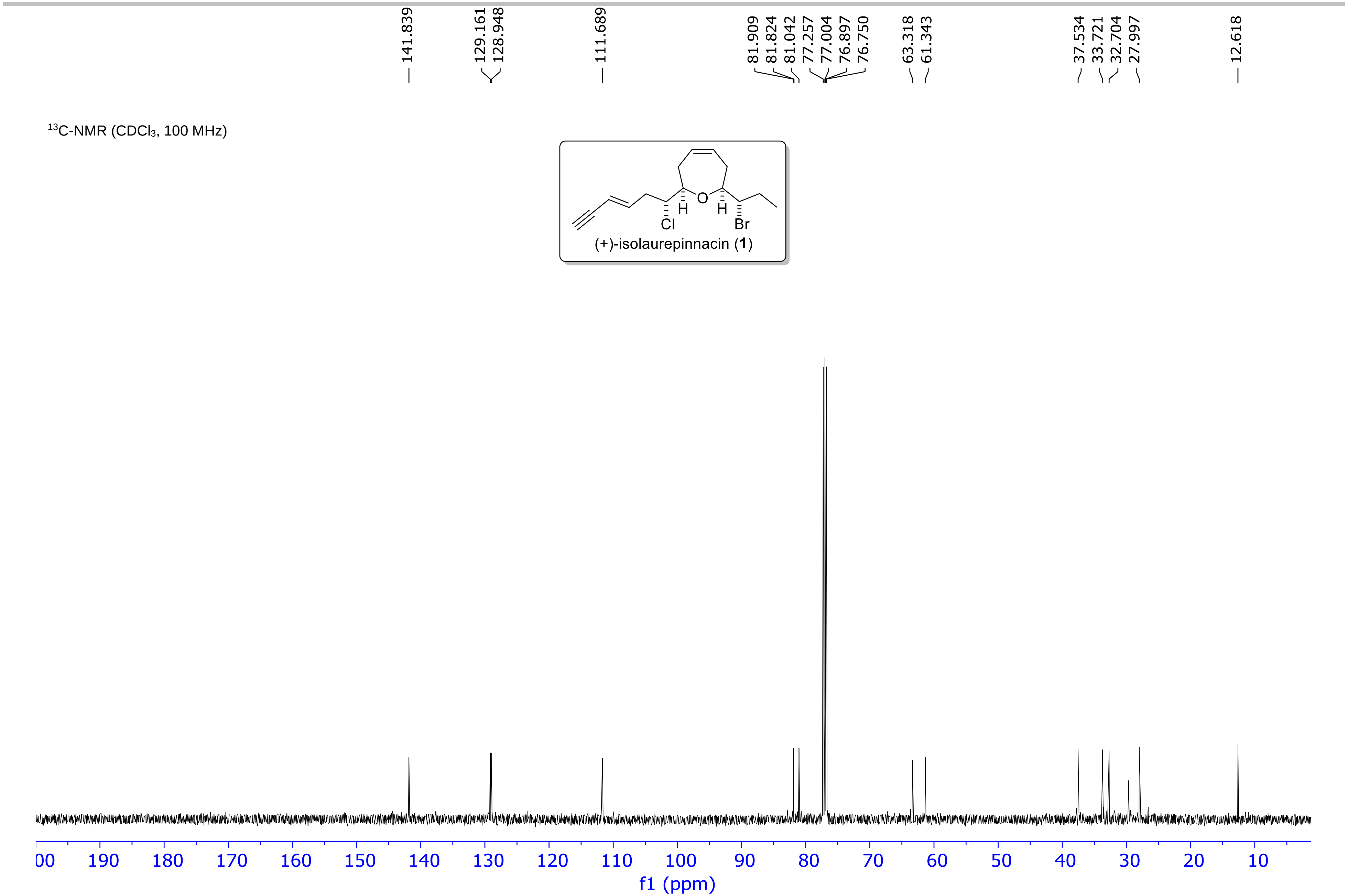
141.839
129.161
128.948
111.689
81.909
81.824
81.042
77.257
77.004
76.897
76.750
63.318
61.343
37.534
33.721
32.704
27.997
12.618
13C-NMR (CDCl3, 100 MHz)
O
H
H
Cl
Br
(+)-isolaurepinnacin (1)
00
190
180
170
160
150
140
130
120
110
100
90
80
70
60
50
40
30
20
10
f1 (ppm)

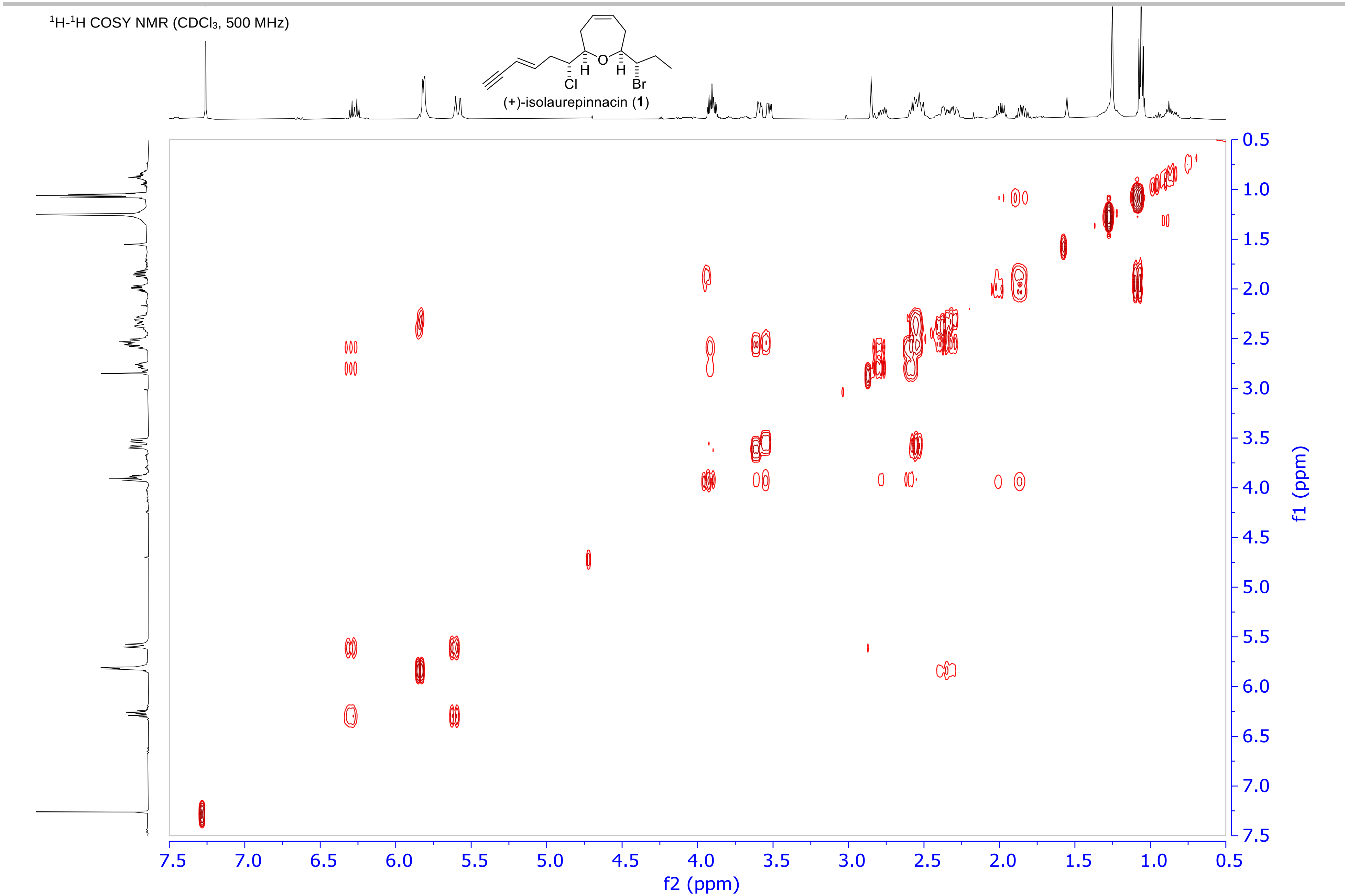

^{1}H-^{1}H COSY NMR (CDCl$_3$, 500 MHz)
H
O
H
Cl
Br
(+)-isolaurepinnacin (**1**)
f2 (ppm)
f1 (ppm)

^{1}H-^{13}C HSQC ed. NMR ($CDCl_3$, 500 MHz)

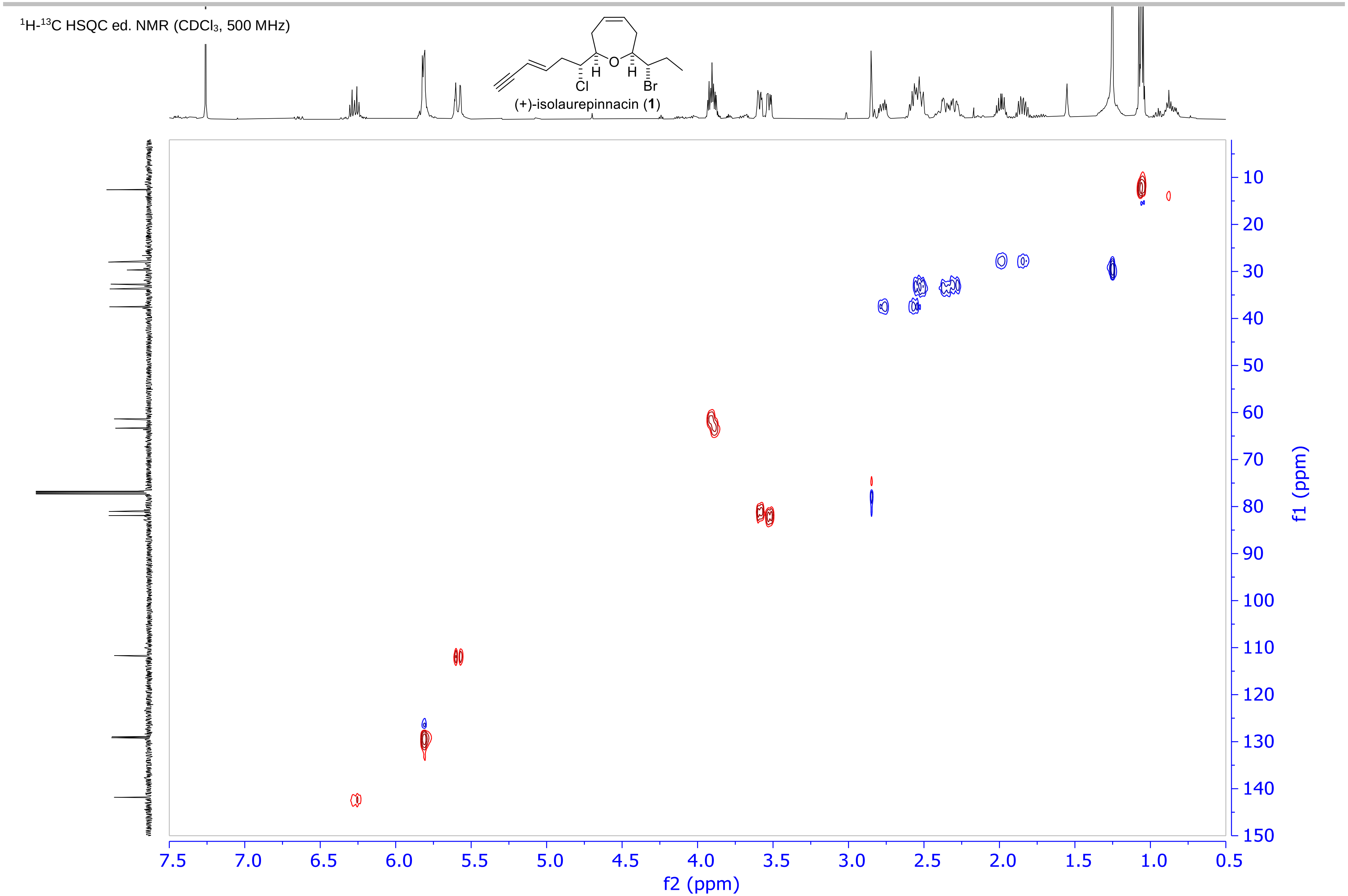

^{1}H-GOESY NMR (CDCl$_3$, 600 MHz)

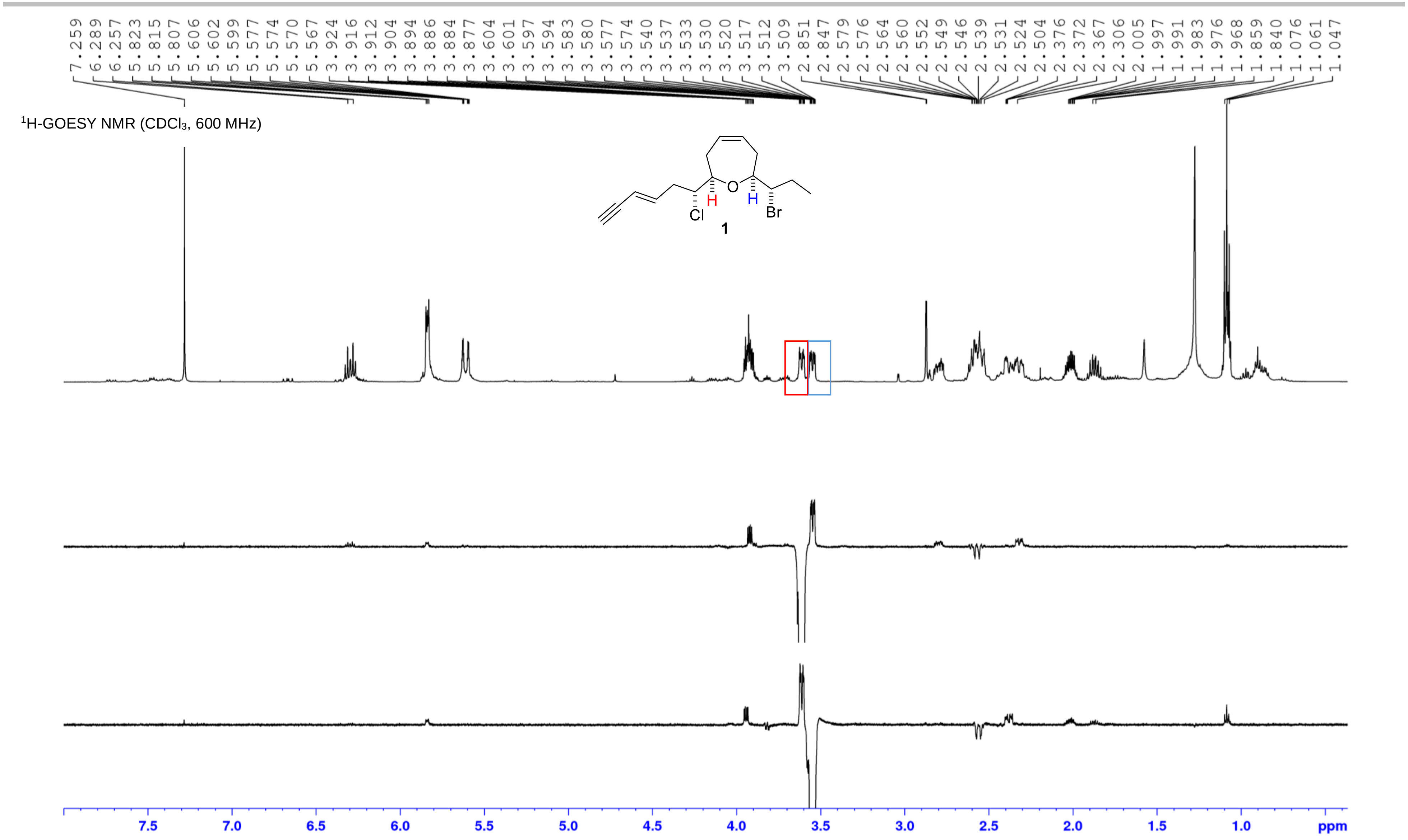

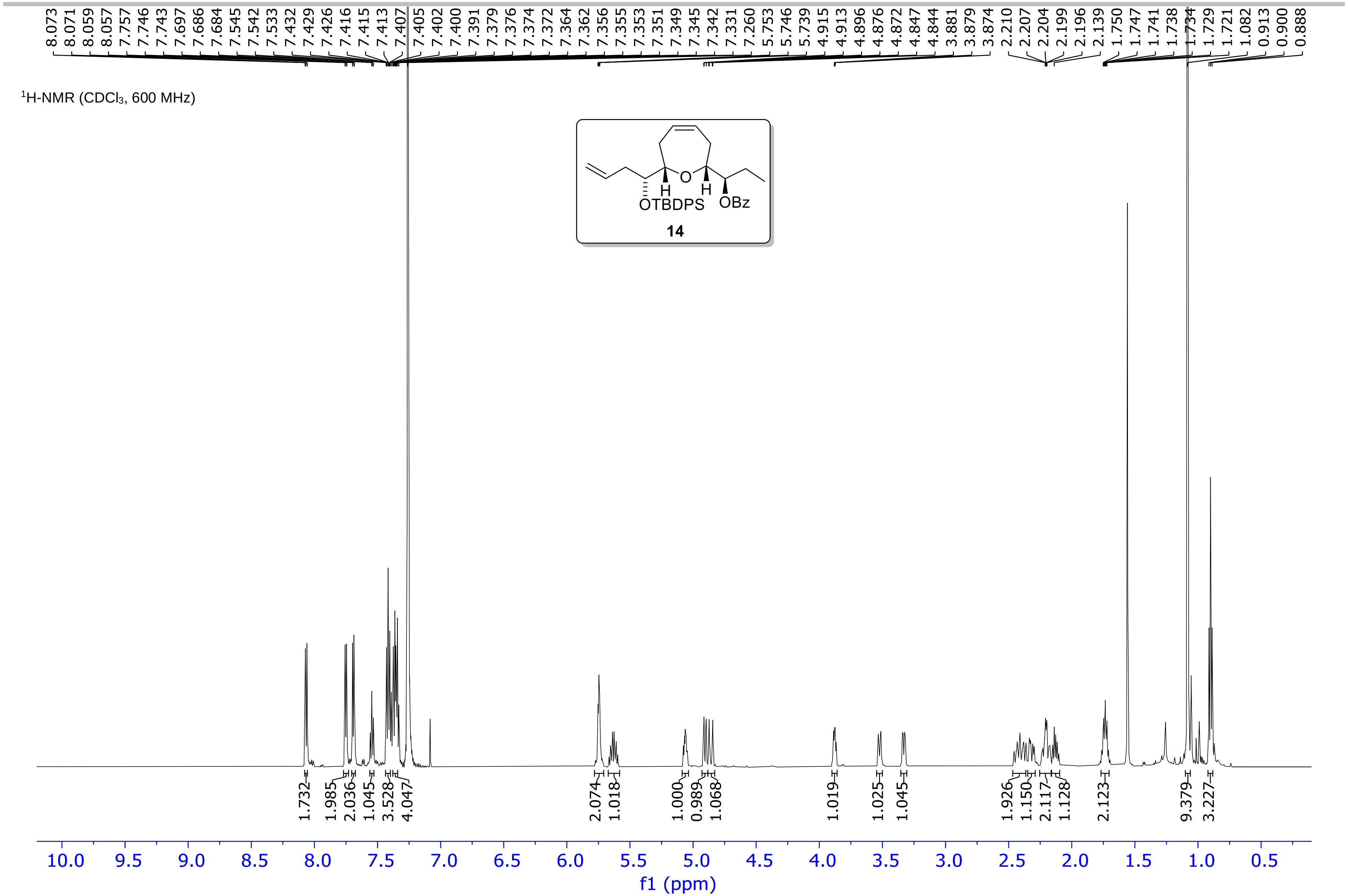

8.073 8.071 8.059 8.057 7.757 7.746 7.743 7.697 7.686 7.684 7.545 7.542 7.533 7.432 7.429 7.426 7.416 7.415 7.413 7.407 7.405 7.402 7.400 7.391 7.379 7.376 7.374 7.372 7.364 7.362 7.356 7.355 7.353 7.351 7.349 7.345 7.342 7.331 7.260 5.753 5.746 5.739 4.915 4.913 4.896 4.876 4.872 4.847 4.844 3.881 3.879 3.874 2.210 2.207 2.204 2.199 2.196 2.139 1.750 1.747 1.741 1.738 1.734 1.729 1.721 1.082 0.913 0.900 0.888
¹H-NMR (CDCl₃, 600 MHz)
OTBDPS
OBz
H
O
14
1.732 1.985 2.036 1.045 3.528 4.047 2.074 1.018 1.000 0.989 1.068 1.019 1.025 1.045 1.926 1.150 2.117 1.128 2.123 9.379 3.227
10.0 9.5 9.0 8.5 8.0 7.5 7.0 6.5 6.0 5.5 5.0 4.5 4.0 3.5 3.0 2.5 2.0 1.5 1.0 0.5
f1 (ppm)

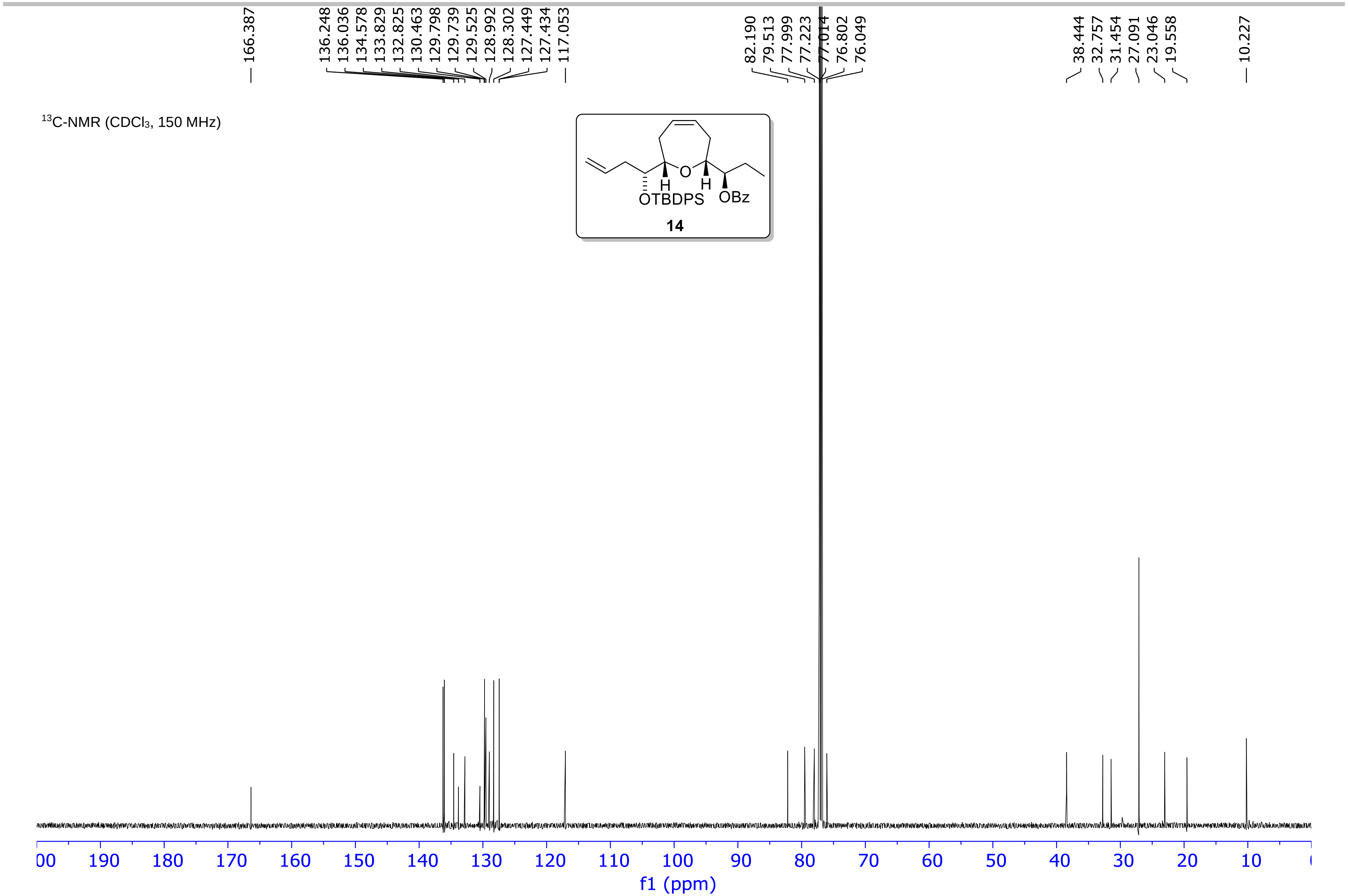
166.387
136.248
136.036
134.578
133.829
132.825
130.463
129.798
129.739
129.525
128.992
128.302
127.449
127.434
117.053
82.190
79.513
77.999
77.223
77.014
76.802
76.049
38.444
32.757
31.454
27.091
23.046
19.558
10.227
13C-NMR (CDCl3, 150 MHz)
O
H
H
OTBDPS
OBz
14
190
180
170
160
150
140
130
120
110
100
90
80
70
60
50
40
30
20
10
f1 (ppm)

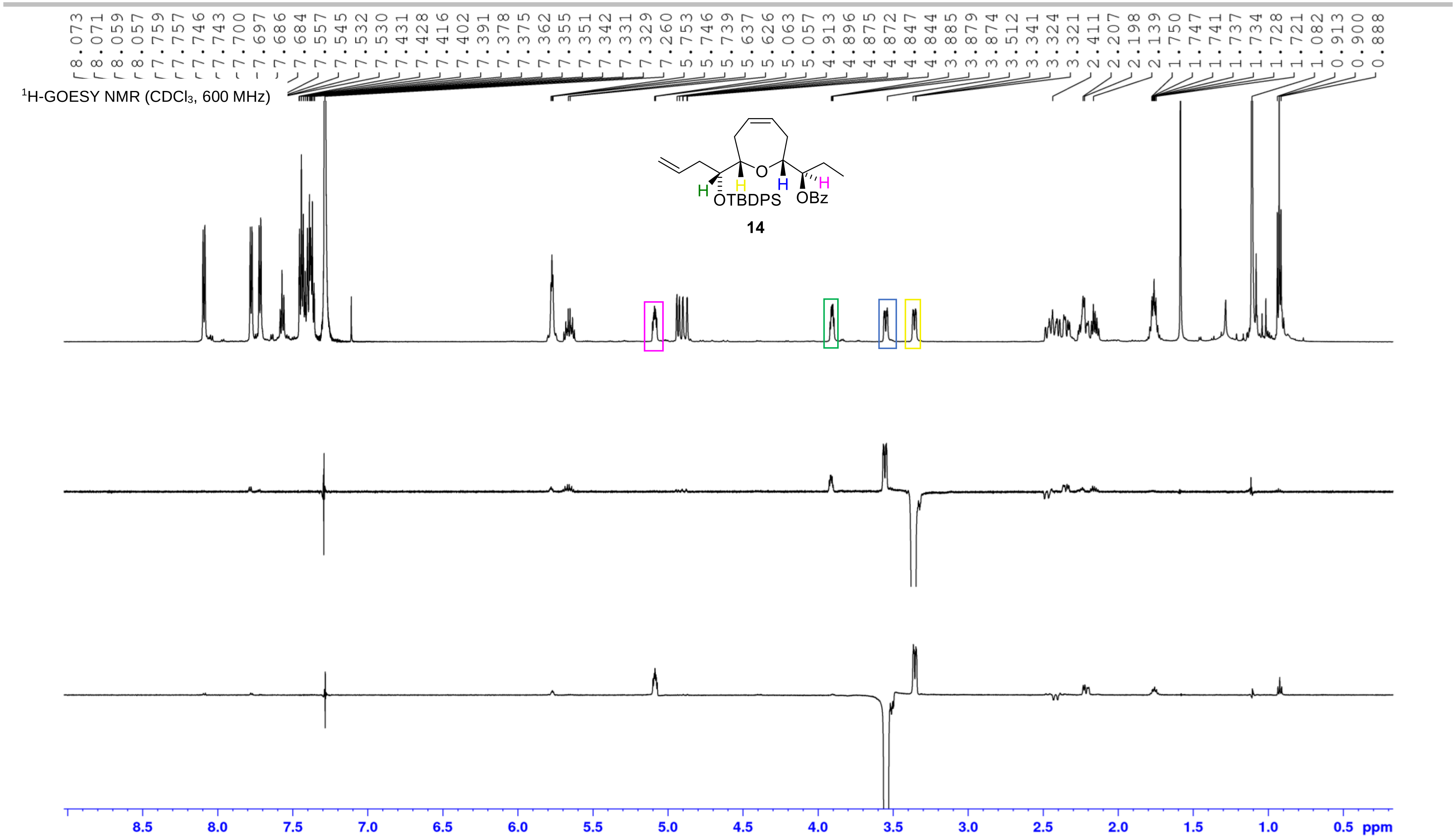
8.073 8.071 8.059 8.057 7.759 7.757 7.746 7.743 7.700 7.697 7.686 7.684 7.557 7.545 7.532 7.530 7.431 7.428 7.416 7.402 7.391 7.378 7.375 7.362 7.355 7.351 7.342 7.331 7.329 7.260 5.753 5.746 5.739 5.637 5.626 5.063 5.057 4.913 4.896 4.875 4.872 4.847 4.844 3.885 3.879 3.874 3.512 3.341 3.324 3.321 2.411 2.207 2.198 2.139 1.750 1.747 1.741 1.737 1.734 1.728 1.721 1.082 0.913 0.900 0.888
^{1}H-GOESY NMR (CDCl$_3$, 600 MHz)
H
H
O
H
H
OTBDPS
OBz
14
8.5 8.0 7.5 7.0 6.5 6.0 5.5 5.0 4.5 4.0 3.5 3.0 2.5 2.0 1.5 1.0 0.5 ppm

^{1}H-NMR (CDCl$_3$, 500 MHz)

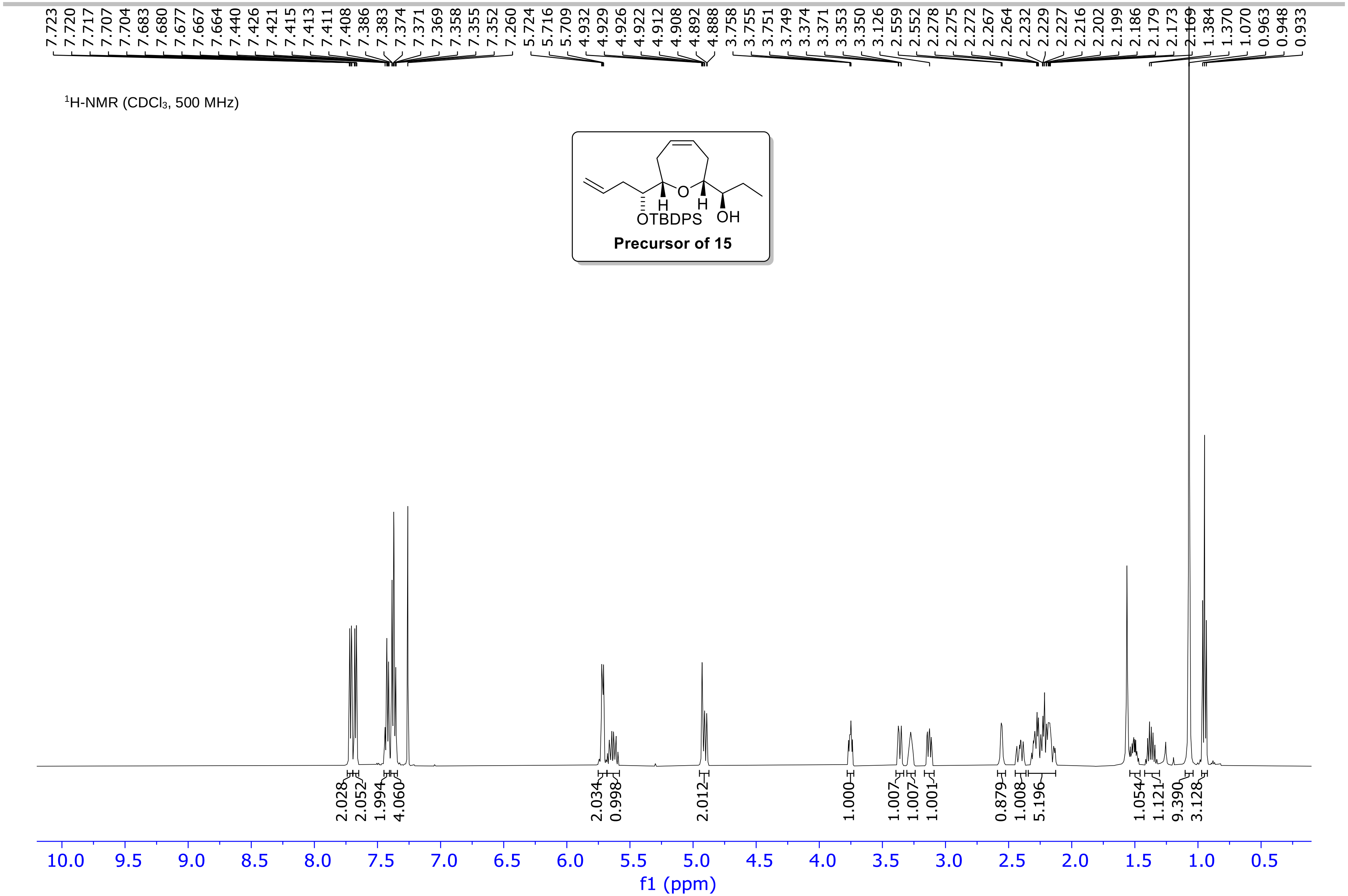

^{13}C-NMR ($CDCl_3$, 100 MHz)

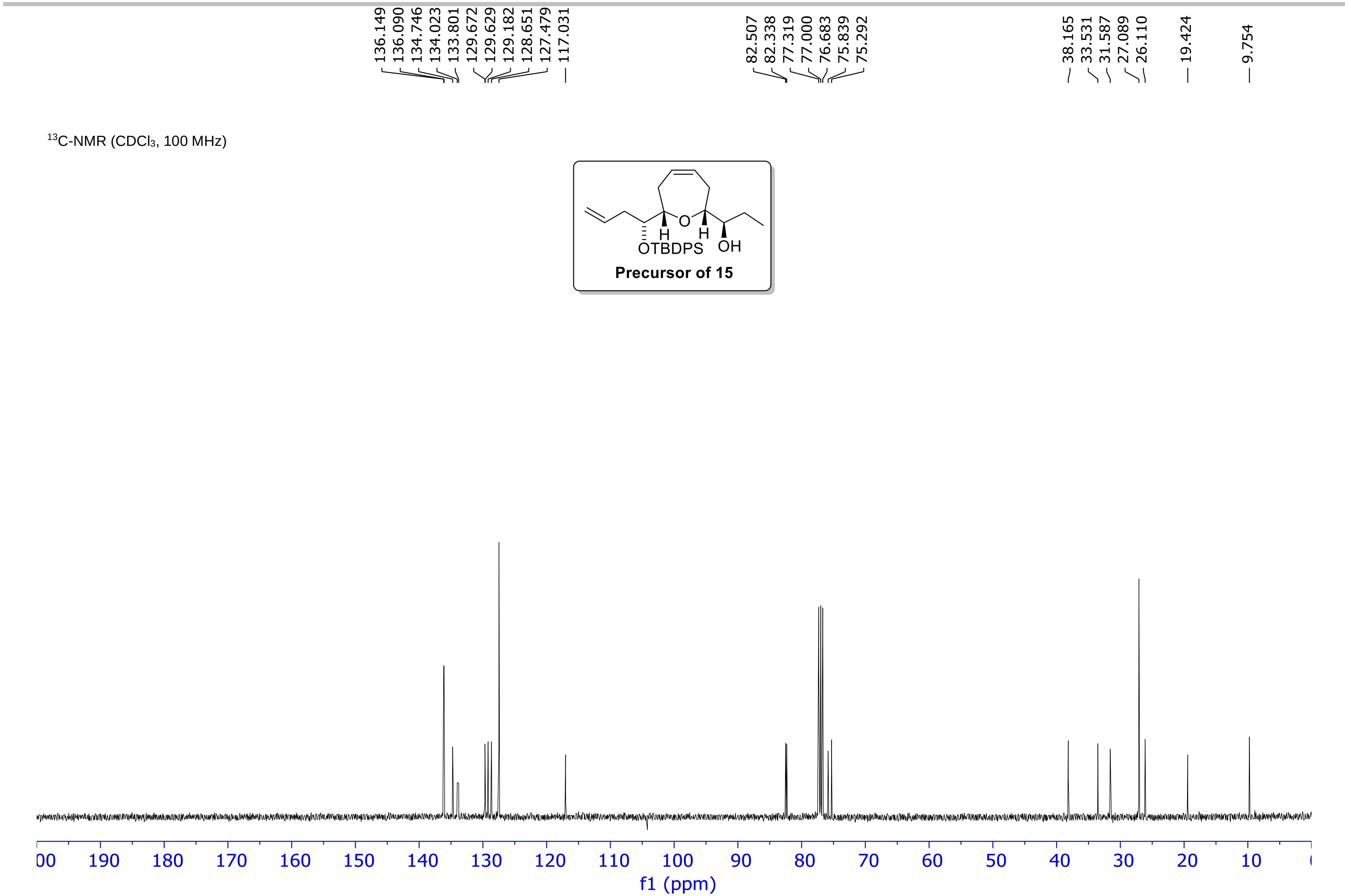

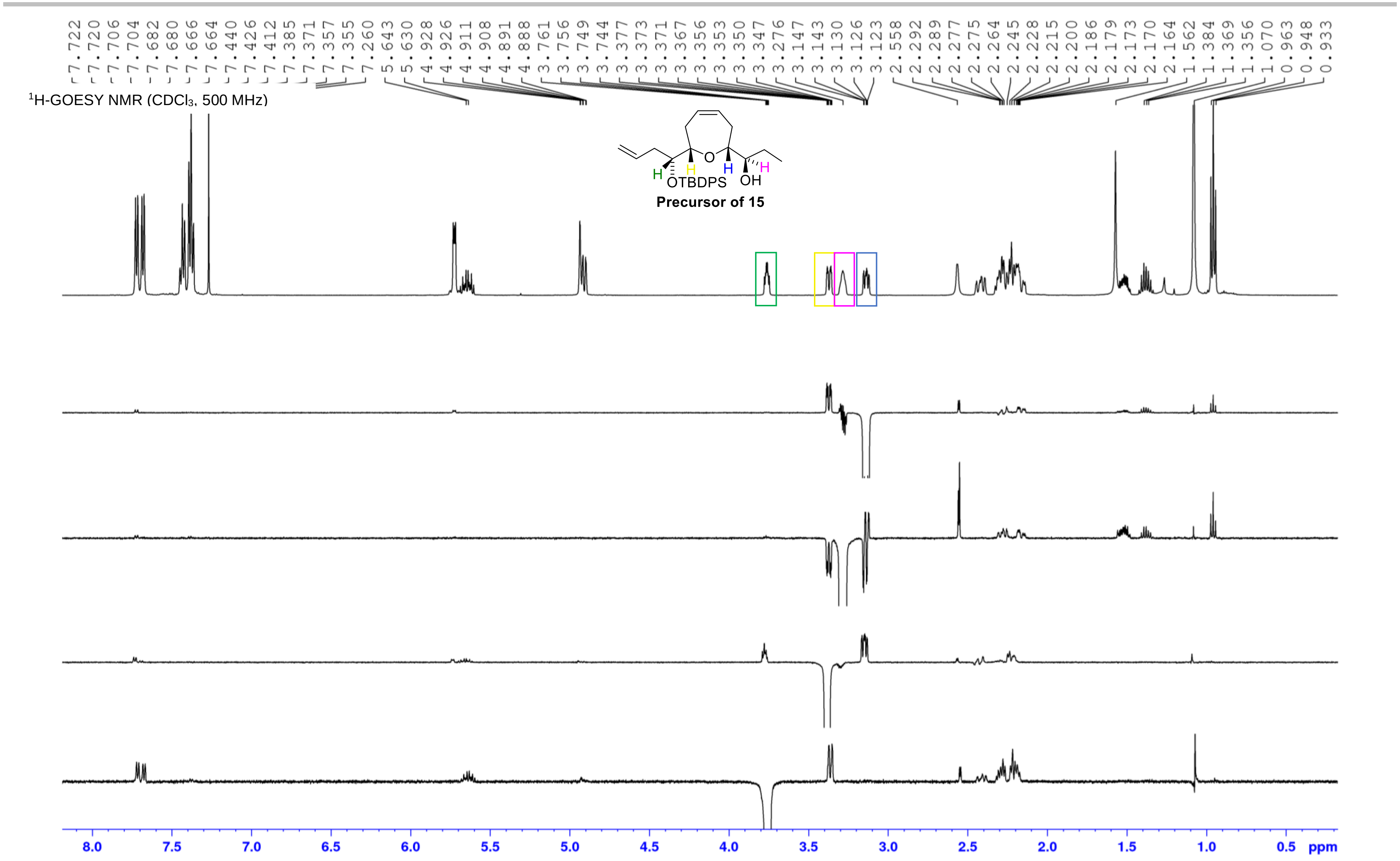

^{1}H-GOESY NMR (CDCl3, 500 MHz)
7.722 7.720 7.706 7.704 7.682 7.680 7.666 7.664 7.440 7.426 7.412 7.385 7.371 7.357 7.355 7.260 5.643 5.630 4.928 4.926 4.911 4.908 4.891 4.888 3.761 3.756 3.749 3.744 3.377 3.373 3.371 3.367 3.356 3.353 3.350 3.347 3.276 3.147 3.143 3.130 3.126 3.123 2.558 2.292 2.289 2.277 2.275 2.264 2.245 2.228 2.215 2.200 2.186 2.179 2.173 2.170 2.164 1.562 1.384 1.369 1.356 1.070 0.963 0.948 0.933
H
H
O
H
H
OTBDPS
OH
Precursor of 15
8.0 7.5 7.0 6.5 6.0 5.5 5.0 4.5 4.0 3.5 3.0 2.5 2.0 1.5 1.0 0.5 ppm

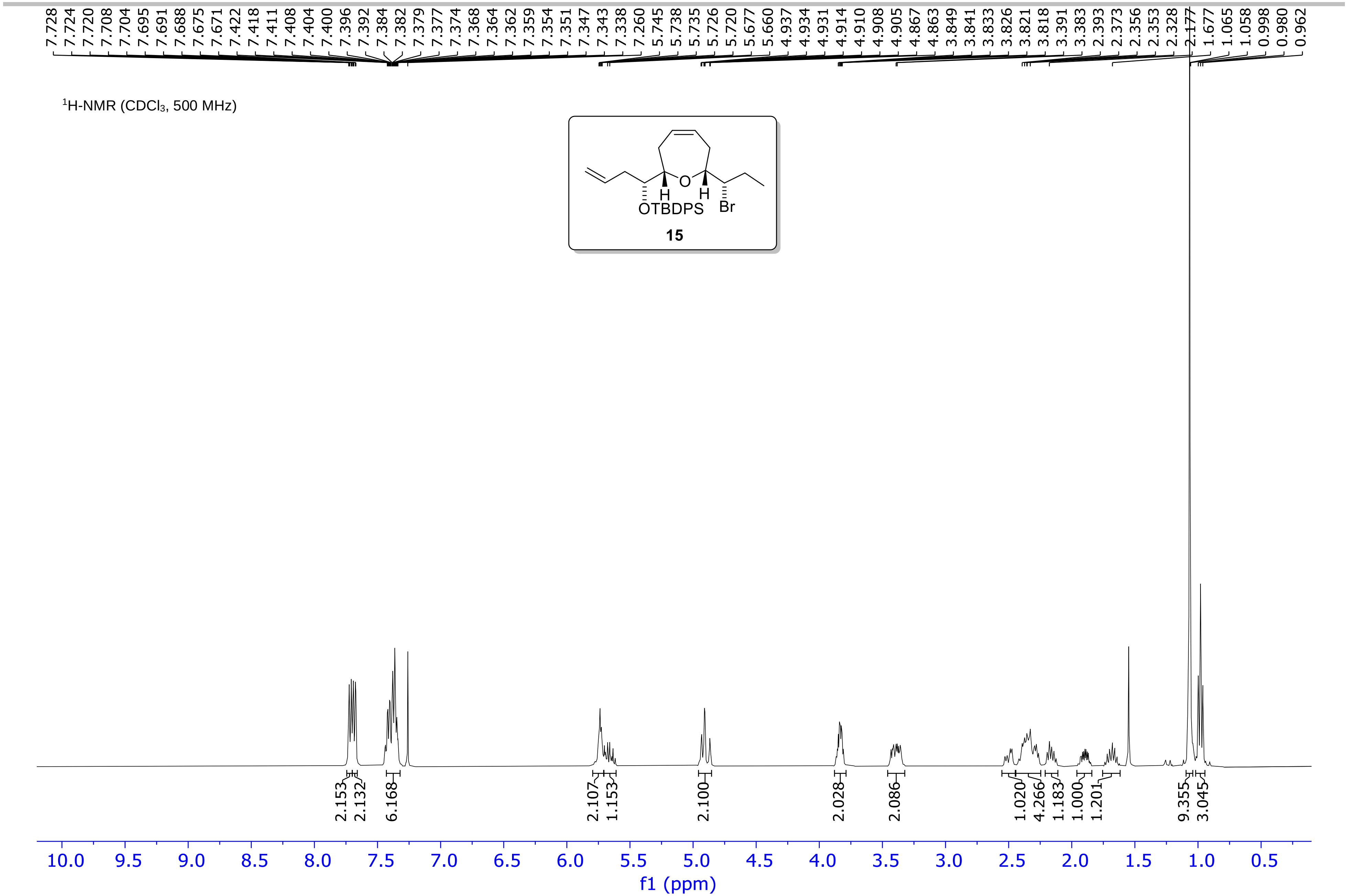
7.728 7.724 7.720 7.708 7.704 7.695 7.691 7.688 7.675 7.671 7.422 7.418 7.411 7.408 7.404 7.400 7.396 7.392 7.384 7.382 7.379 7.377 7.374 7.368 7.364 7.362 7.359 7.354 7.351 7.347 7.343 7.338 7.260 5.745 5.738 5.735 5.726 5.720 5.677 5.660 4.937 4.934 4.931 4.914 4.910 4.908 4.905 4.867 4.863 3.849 3.841 3.833 3.826 3.821 3.818 3.391 3.383 2.393 2.373 2.356 2.353 2.328 2.177 1.677 1.065 1.058 0.998 0.980 0.962
^{1}H-NMR (CDCl$_3$, 500 MHz)
H
O
H
OTBDPS
Br
15
2.153 2.132 6.168 2.107 1.153 2.100 2.028 2.086 1.020 4.266 1.183 1.000 1.201 9.355 3.045
10.0 9.5 9.0 8.5 8.0 7.5 7.0 6.5 6.0 5.5 5.0 4.5 4.0 3.5 3.0 2.5 2.0 1.5 1.0 0.5
f1 (ppm)

^{13}C-NMR ($CDCl_3$, 100 MHz)

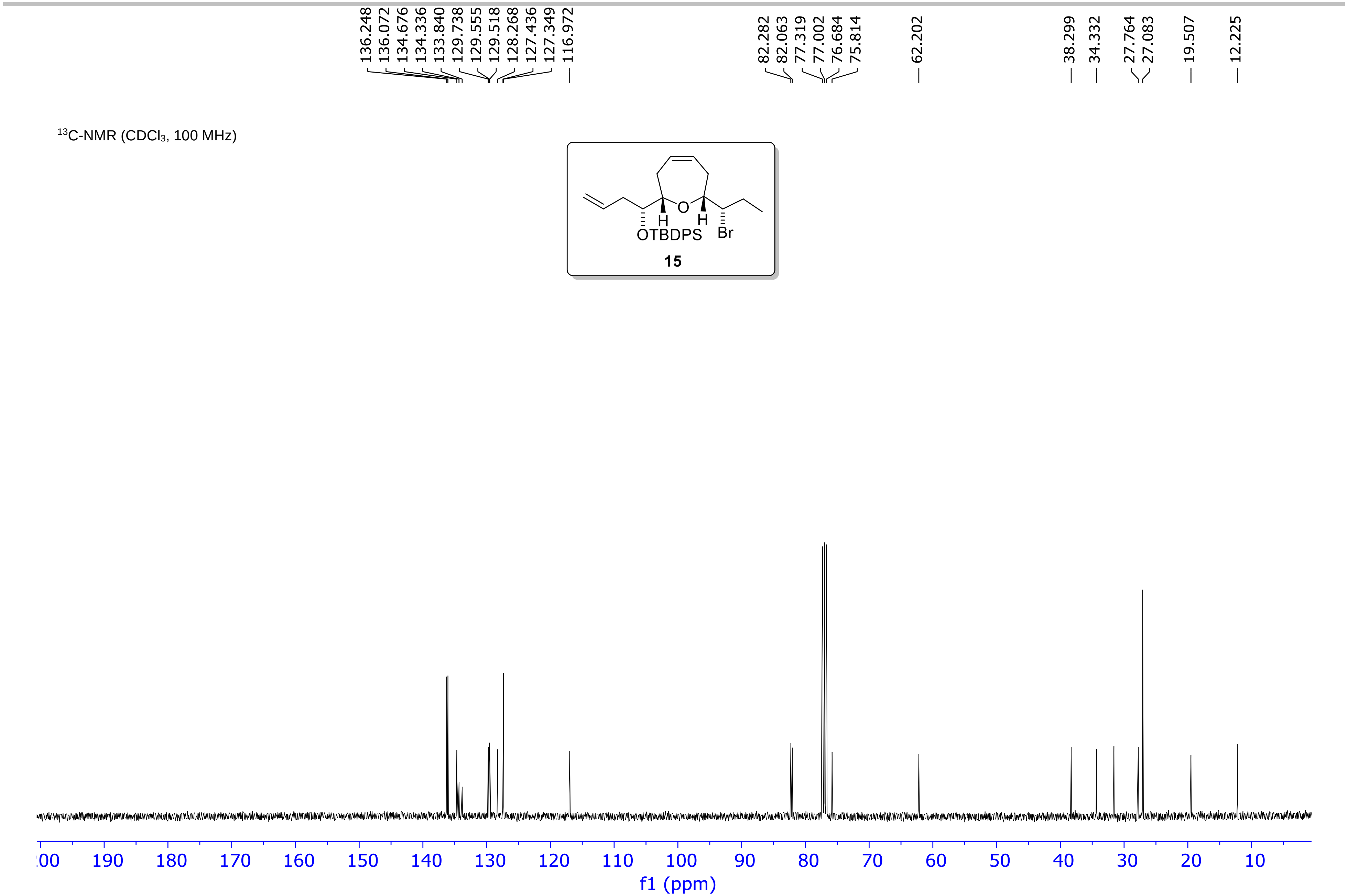

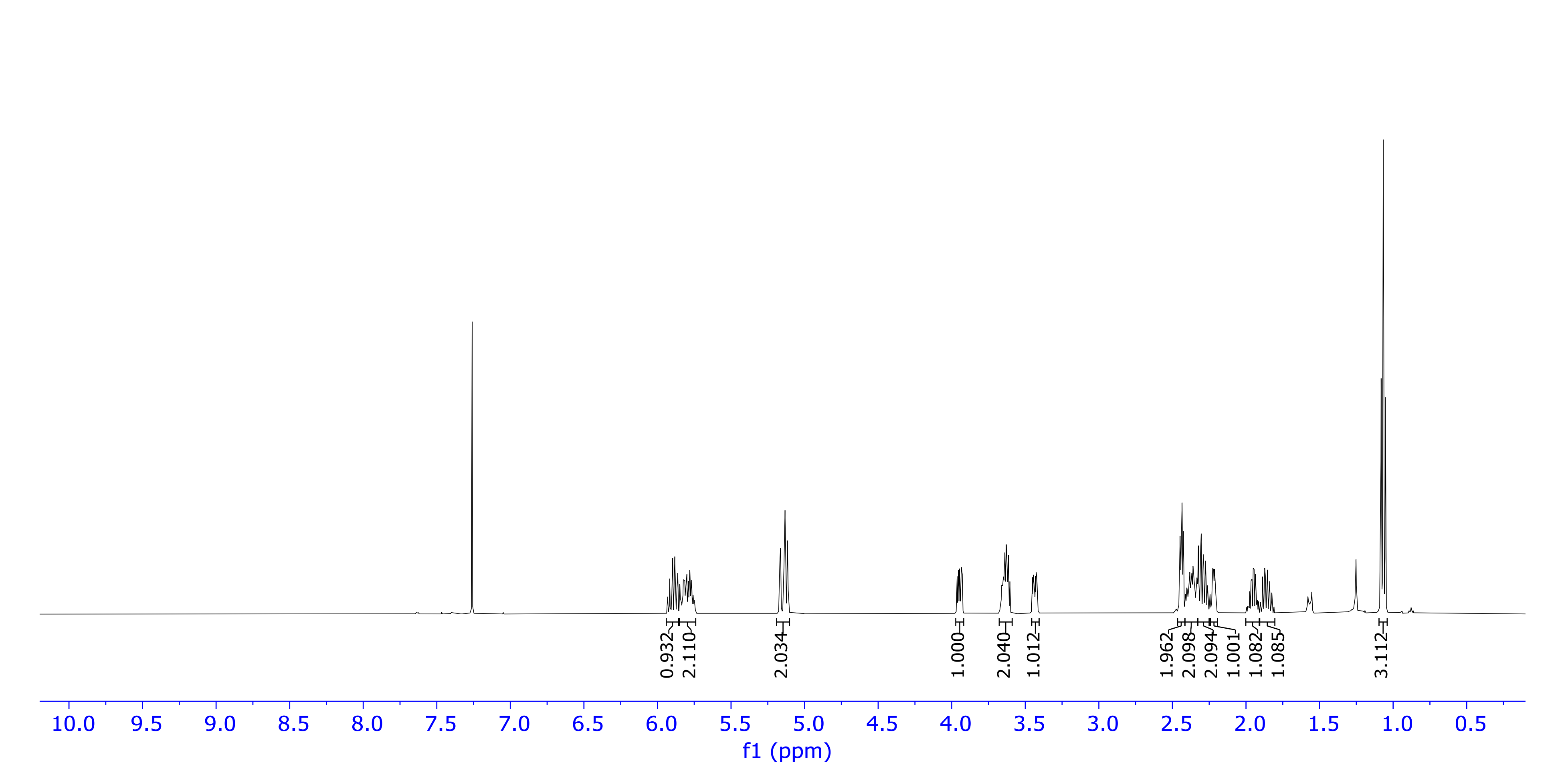
7.260 5.897 5.883 5.862 5.800 5.784 5.780 5.779 5.167 5.164 5.139 5.137 5.135 5.133 5.130 5.119 5.117 5.115 5.113 3.964 3.957 3.954 3.948 3.944 3.938 3.935 3.928 3.649 3.643 3.639 3.629 3.622 3.615 3.446 3.431 3.426 3.422 2.447 2.440 2.437 2.434 2.425 2.382 2.371 2.368 2.360 2.357 2.324 2.319 2.309 2.305 2.291 2.287 2.276 2.273 2.227 2.221 2.215 2.211 1.951 1.945 1.936 1.930 1.873 1.868 1.854 1.082 1.067 1.053
^{1}H-NMR (CDCl$_3$, 500 MHz)
0.932 2.110 2.034 1.000 2.040 1.012 1.962 2.098 2.094 1.001 1.082 1.085 3.112
10.0 9.5 9.0 8.5 8.0 7.5 7.0 6.5 6.0 5.5 5.0 4.5 4.0 3.5 3.0 2.5 2.0 1.5 1.0 0.5
f1 (ppm)

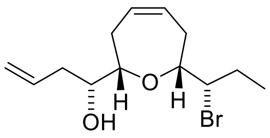
OH
H
O
H
Br

16

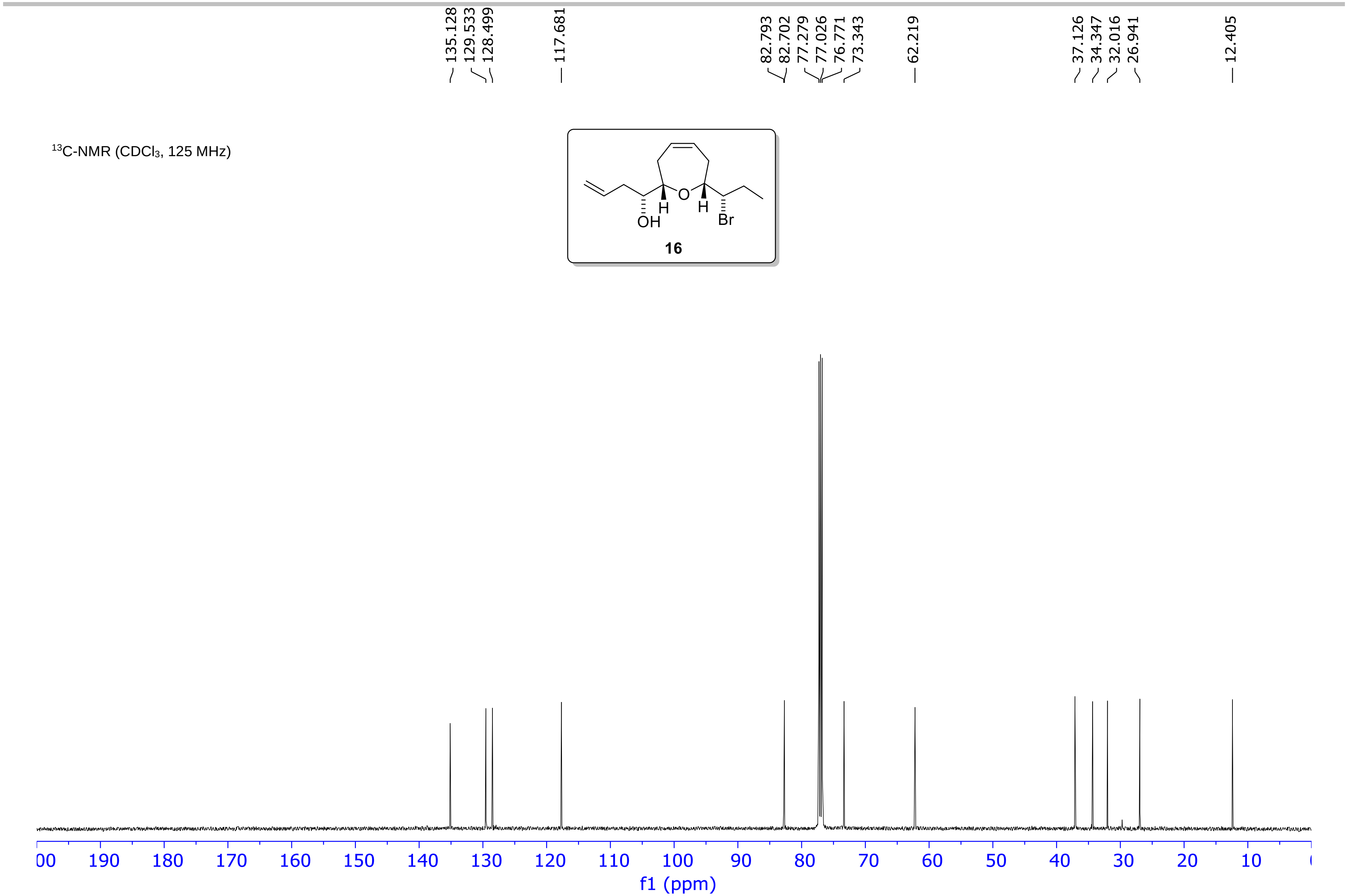
13C-NMR (CDCl3, 125 MHz)
135.128
129.533
128.499
117.681
82.793
82.702
77.279
77.026
76.771
73.343
62.219
37.126
34.347
32.016
26.941
12.405
OH
H
O
H
Br
16
190 180 170 160 150 140 130 120 110 100 90 80 70 60 50 40 30 20 10
f1 (ppm)

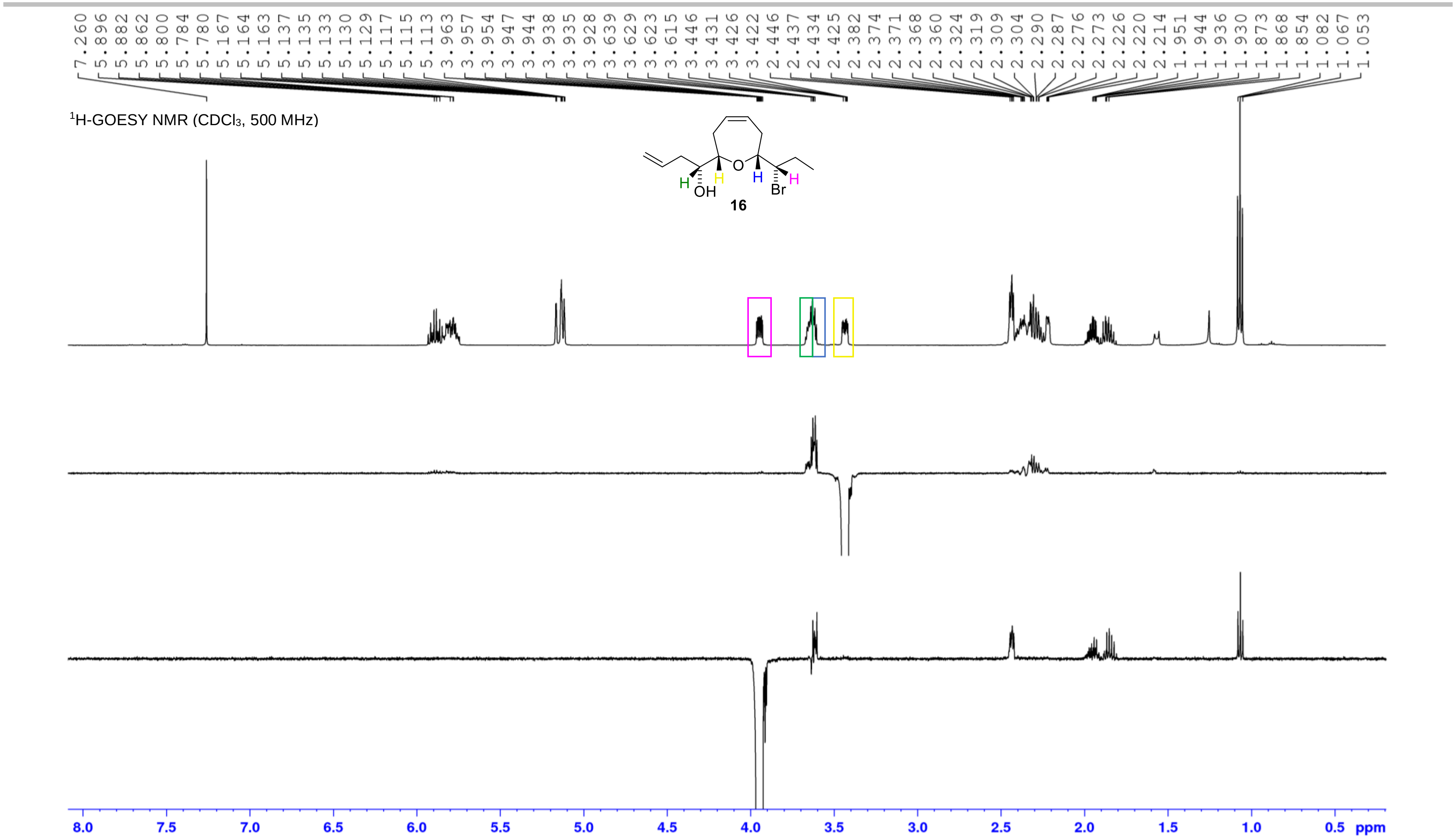
7.260
5.896
5.882
5.862
5.800
5.784
5.780
5.167
5.164
5.163
5.137
5.135
5.133
5.130
5.129
5.117
5.115
5.113
3.963
3.957
3.954
3.947
3.944
3.938
3.935
3.928
3.639
3.629
3.623
3.615
3.446
3.431
3.426
3.422
2.446
2.437
2.434
2.425
2.382
2.374
2.371
2.368
2.360
2.324
2.319
2.309
2.304
2.290
2.287
2.276
2.273
2.226
2.220
2.214
1.951
1.944
1.936
1.930
1.873
1.868
1.854
1.082
1.067
1.053
1H-GOESY NMR (CDCl3, 500 MHz)
H
H
OH
O
H
Br
H
16
8.0 7.5 7.0 6.5 6.0 5.5 5.0 4.5 4.0 3.5 3.0 2.5 2.0 1.5 1.0 0.5 ppm

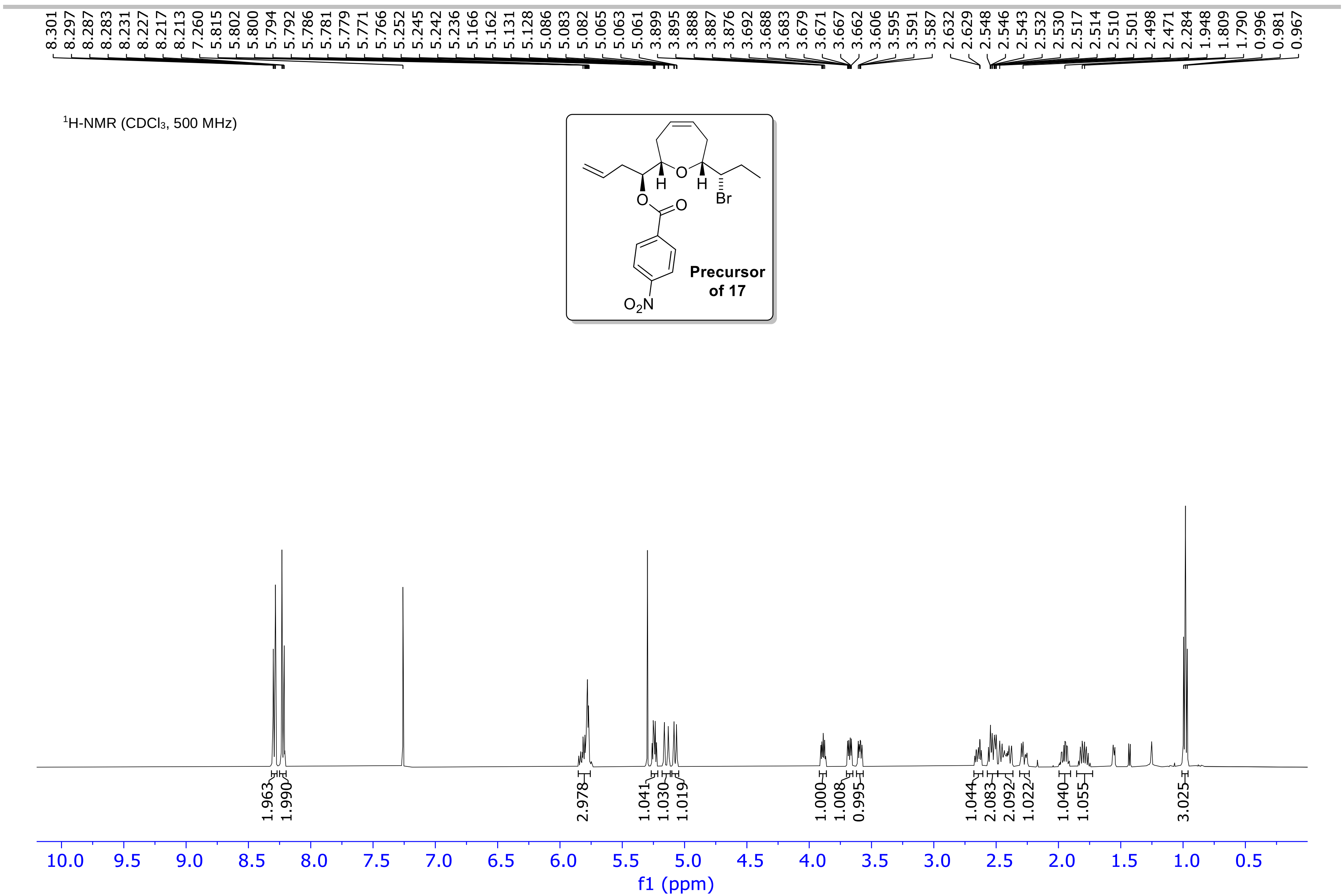

8.301 8.297 8.287 8.283 8.231 8.227 8.217 8.213 7.260 5.815 5.802 5.800 5.794 5.792 5.786 5.781 5.779 5.771 5.766 5.252 5.245 5.242 5.236 5.166 5.162 5.131 5.128 5.086 5.083 5.082 5.065 5.063 5.061 3.899 3.895 3.888 3.887 3.876 3.692 3.688 3.683 3.679 3.671 3.667 3.662 3.606 3.595 3.591 3.587 2.632 2.629 2.548 2.546 2.543 2.532 2.530 2.517 2.514 2.510 2.501 2.498 2.471 2.284 1.948 1.809 1.790 0.996 0.981 0.967
1H-NMR (CDCl3, 500 MHz)
O
H
H
Br
O
O
O2N
Precursor of 17
1.963 1.990 2.978 1.041 1.030 1.019 1.000 1.008 0.995 1.044 2.083 2.092 1.022 1.040 1.055 3.025
10.0 9.5 9.0 8.5 8.0 7.5 7.0 6.5 6.0 5.5 5.0 4.5 4.0 3.5 3.0 2.5 2.0 1.5 1.0 0.5
f1 (ppm)

^{13}C-NMR ($CDCl_3$, 125 MHz)

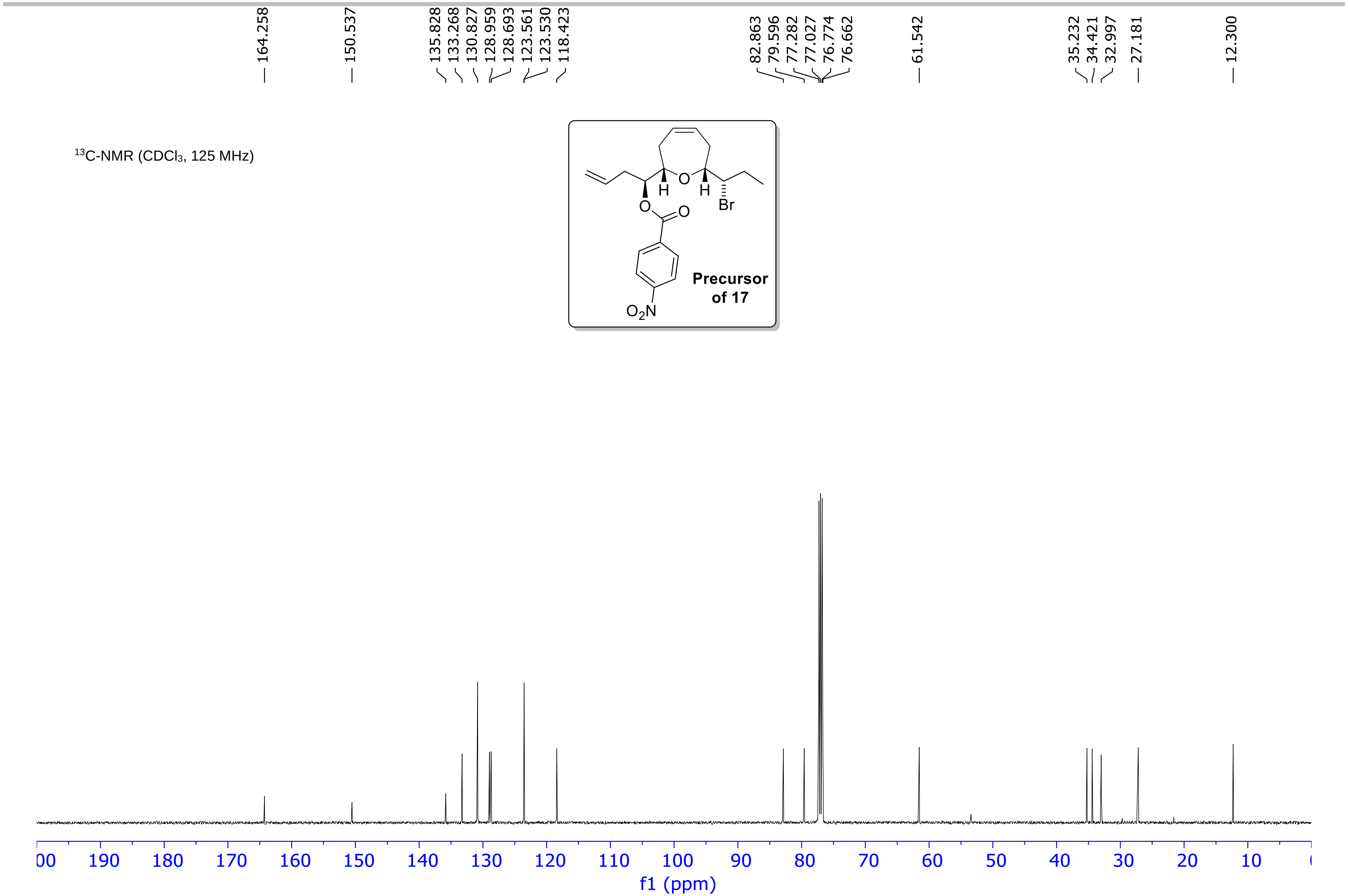

Precursor of 17

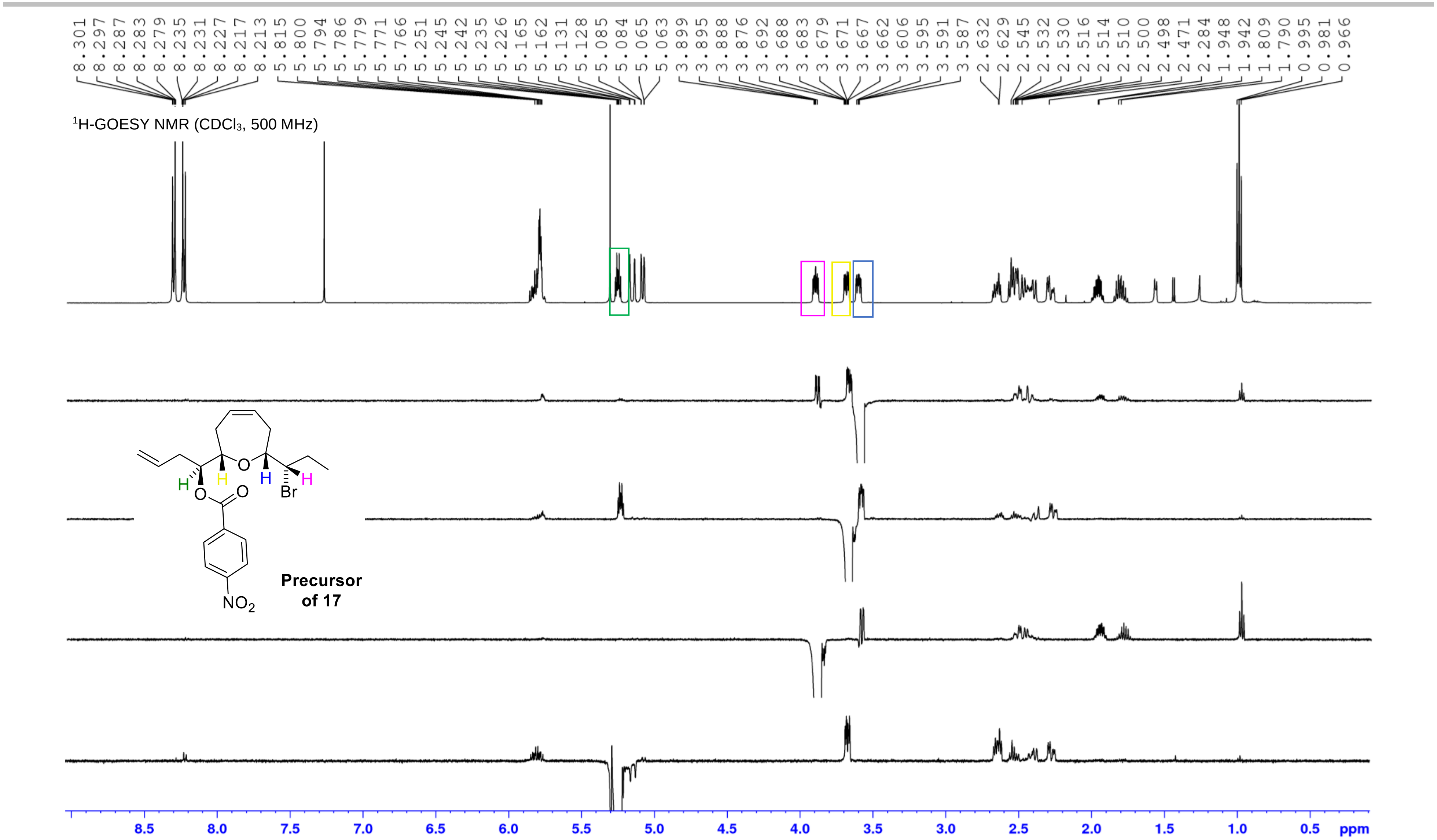

8.301 8.297 8.287 8.283 8.279 8.235 8.231 8.227 8.217 8.213 5.815 5.800 5.794 5.786 5.779 5.771 5.766 5.251 5.245 5.242 5.235 5.226 5.165 5.162 5.131 5.128 5.085 5.084 5.065 5.063 3.899 3.895 3.888 3.876 3.692 3.688 3.683 3.679 3.671 3.667 3.662 3.606 3.595 3.591 3.587 2.632 2.629 2.545 2.532 2.530 2.516 2.514 2.510 2.500 2.498 2.471 2.284 1.948 1.942 1.809 1.790 0.995 0.981 0.966
¹H-GOESY NMR (CDCl₃, 500 MHz)
Precursor of 17
8.5 8.0 7.5 7.0 6.5 6.0 5.5 5.0 4.5 4.0 3.5 3.0 2.5 2.0 1.5 1.0 0.5 ppm

^{1}H-NMR ($CDCl_3$, 500 MHz)

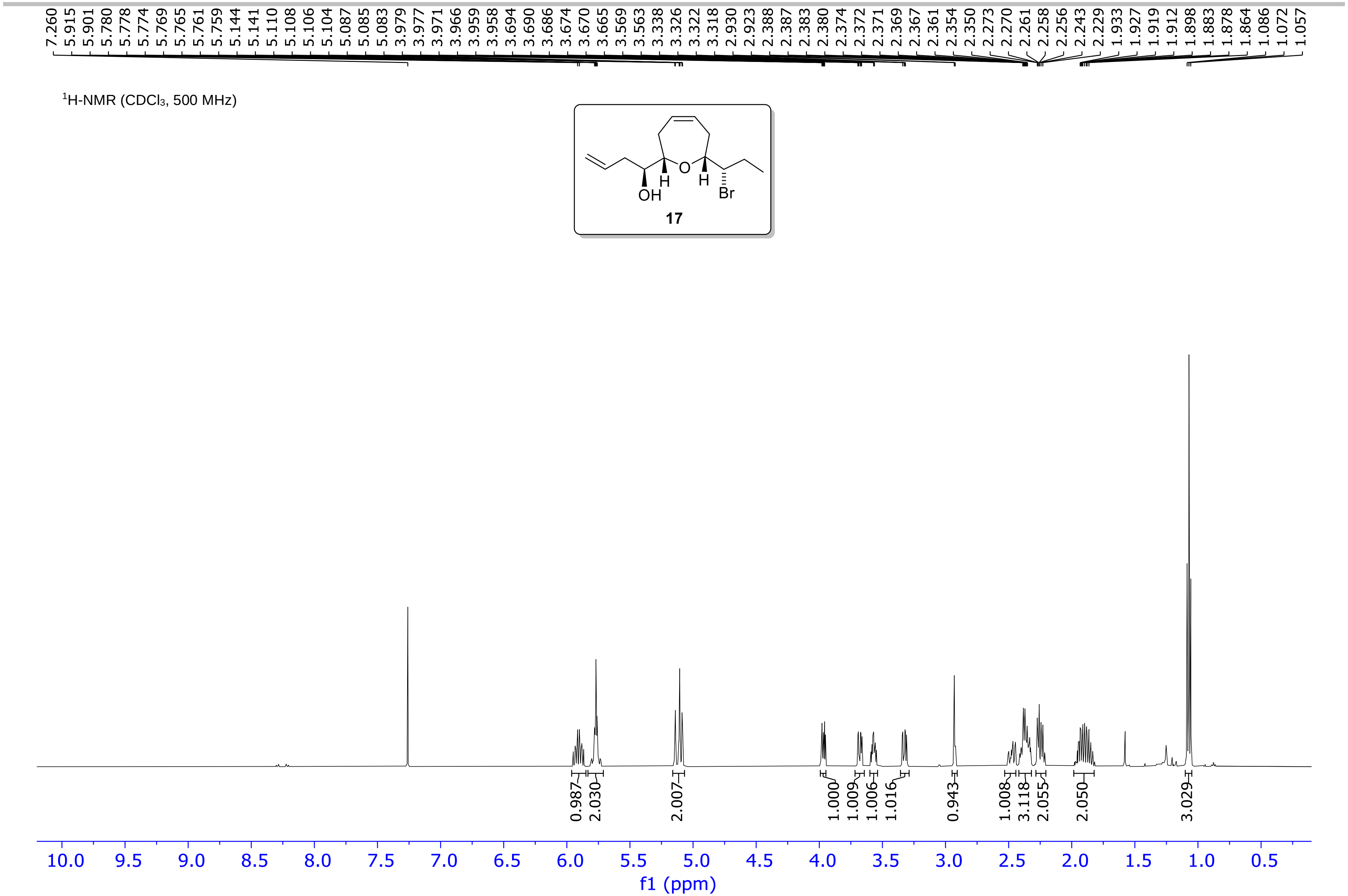

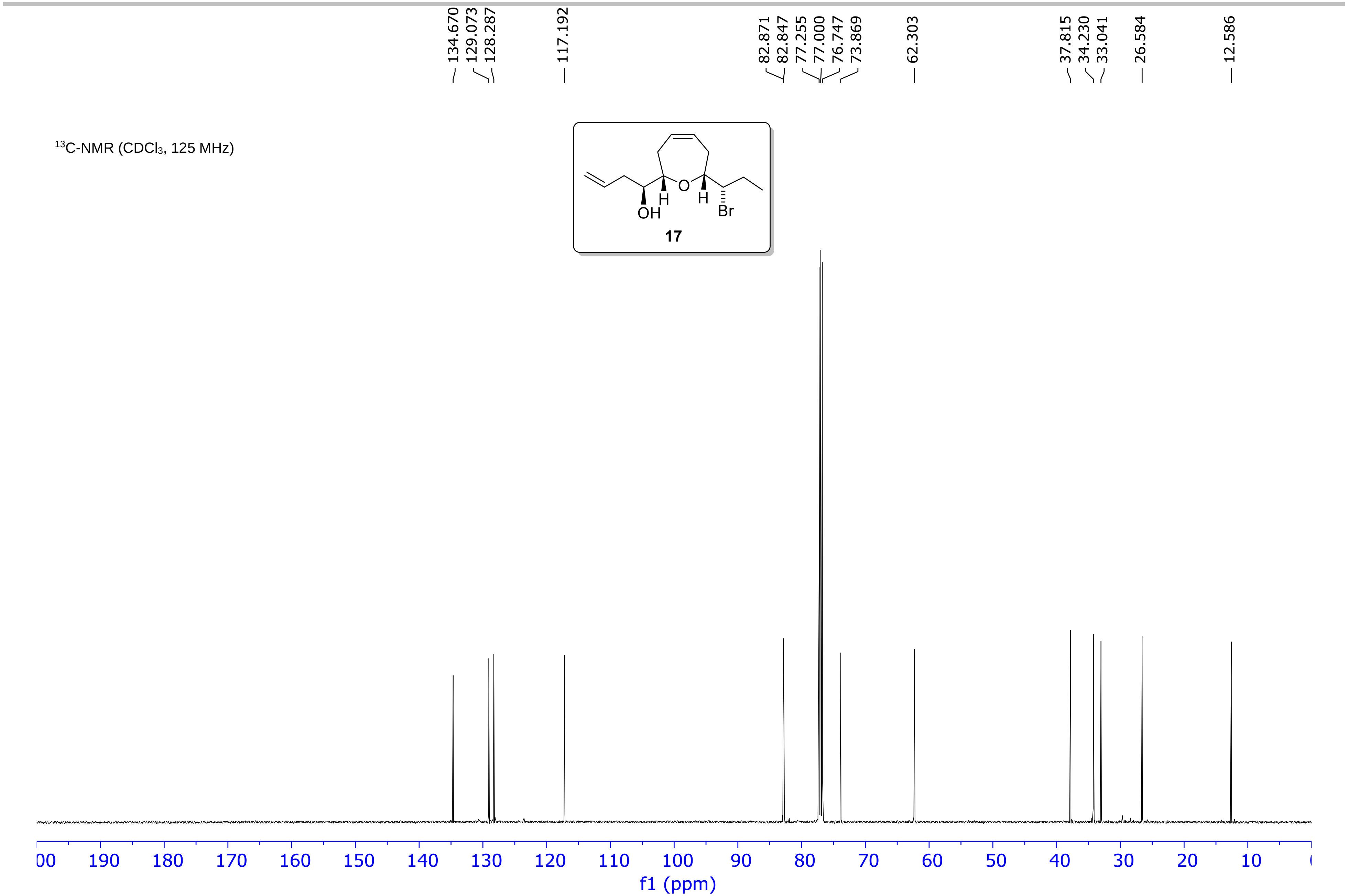
134.670
129.073
128.287
117.192
82.871
82.847
77.255
77.000
76.747
73.869
62.303
37.815
34.230
33.041
26.584
12.586
13C-NMR (CDCl3, 125 MHz)
OH
H
O
H
Br
17
00
190
180
170
160
150
140
130
120
110
100
90
80
70
60
50
40
30
20
10
f1 (ppm)

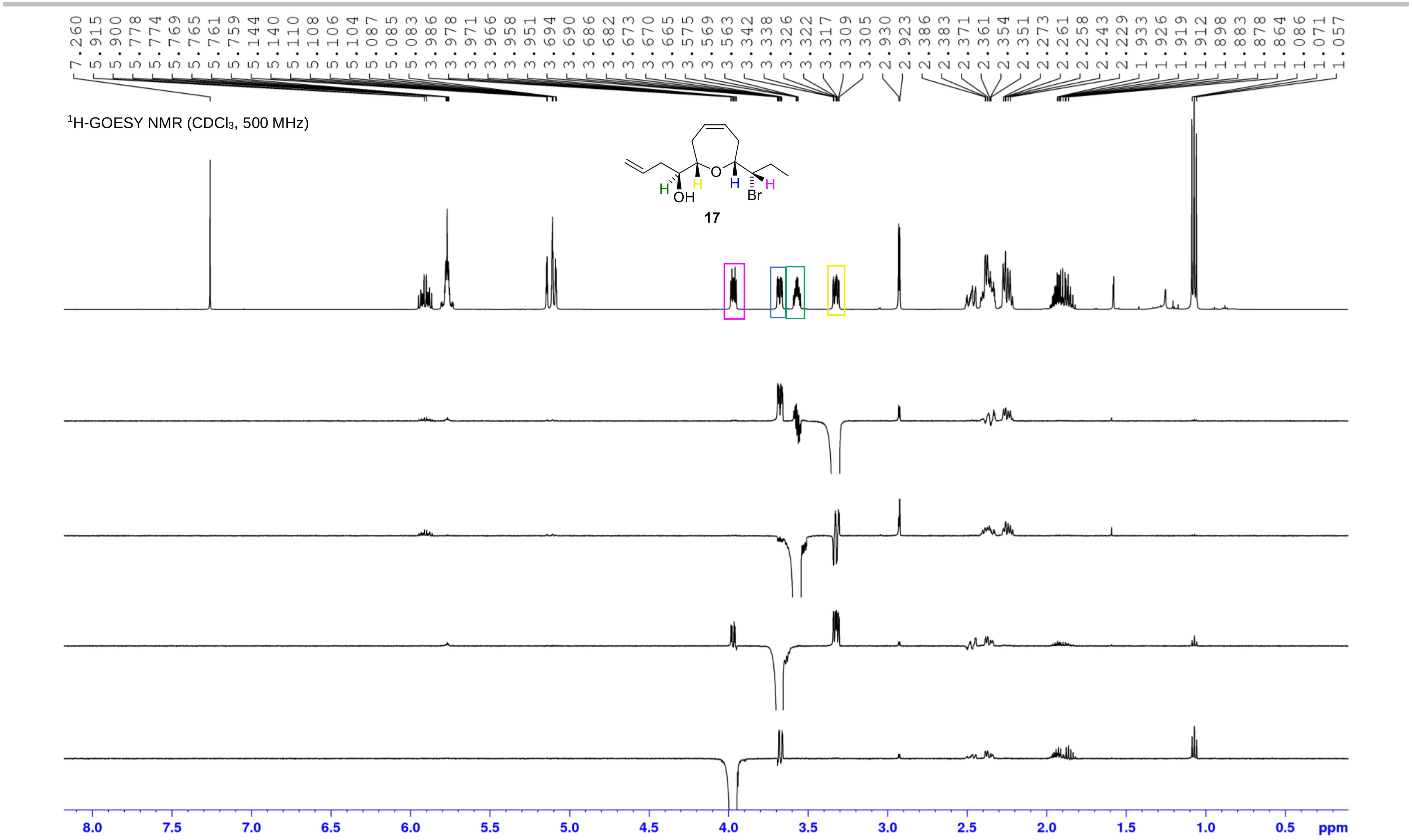

7.260
5.915
5.900
5.778
5.774
5.769
5.765
5.761
5.759
5.144
5.140
5.110
5.108
5.106
5.104
5.087
5.085
5.083
3.986
3.978
3.971
3.966
3.958
3.951
3.694
3.690
3.686
3.682
3.673
3.670
3.665
3.575
3.569
3.563
3.342
3.338
3.326
3.322
3.317
3.309
3.305
2.930
2.923
2.386
2.383
2.371
2.361
2.354
2.351
2.273
2.261
2.258
2.243
2.229
1.933
1.926
1.919
1.912
1.898
1.883
1.878
1.864
1.086
1.071
1.057
¹H-GOESY NMR (CDCl₃, 500 MHz)
O
H
H
H
H
OH
Br
17
8.0
7.5
7.0
6.5
6.0
5.5
5.0
4.5
4.0
3.5
3.0
2.5
2.0
1.5
1.0
0.5
ppm

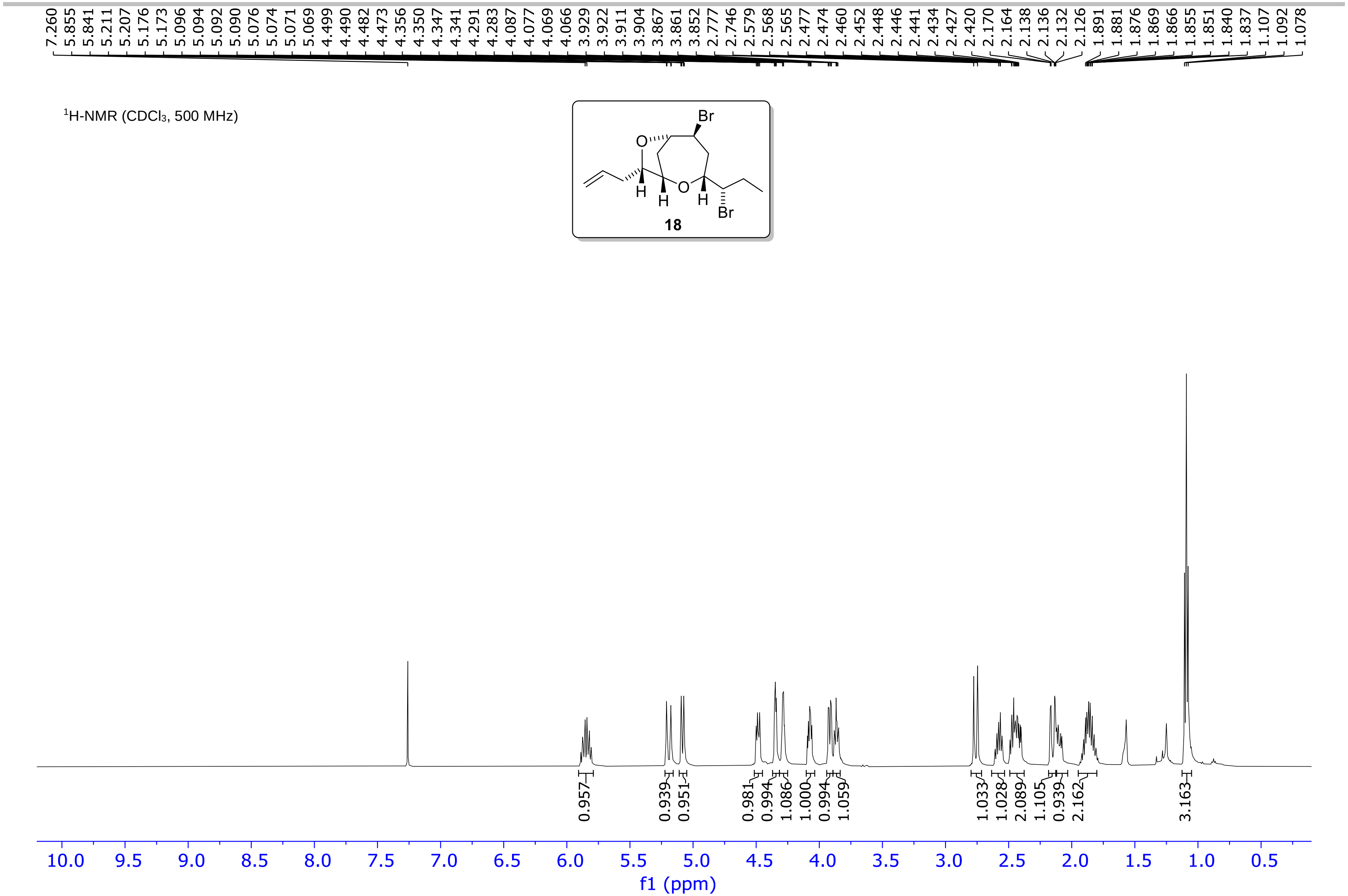

^{1}H-NMR ($CDCl_3$, 500 MHz)

^{13}C-NMR ($CDCl_3$, 125 MHz)

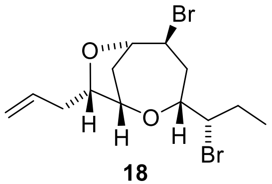

18

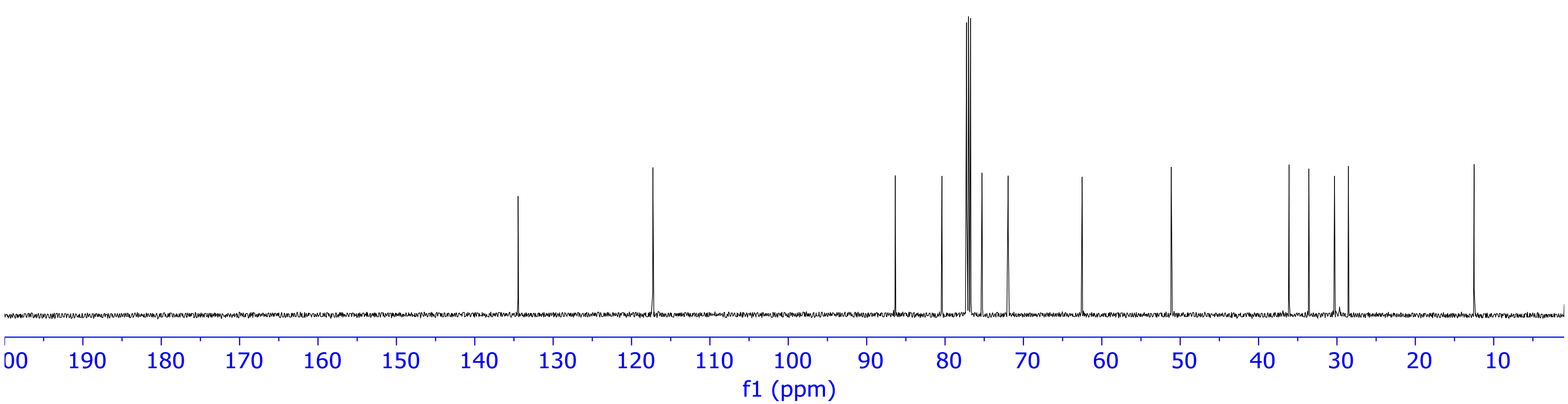

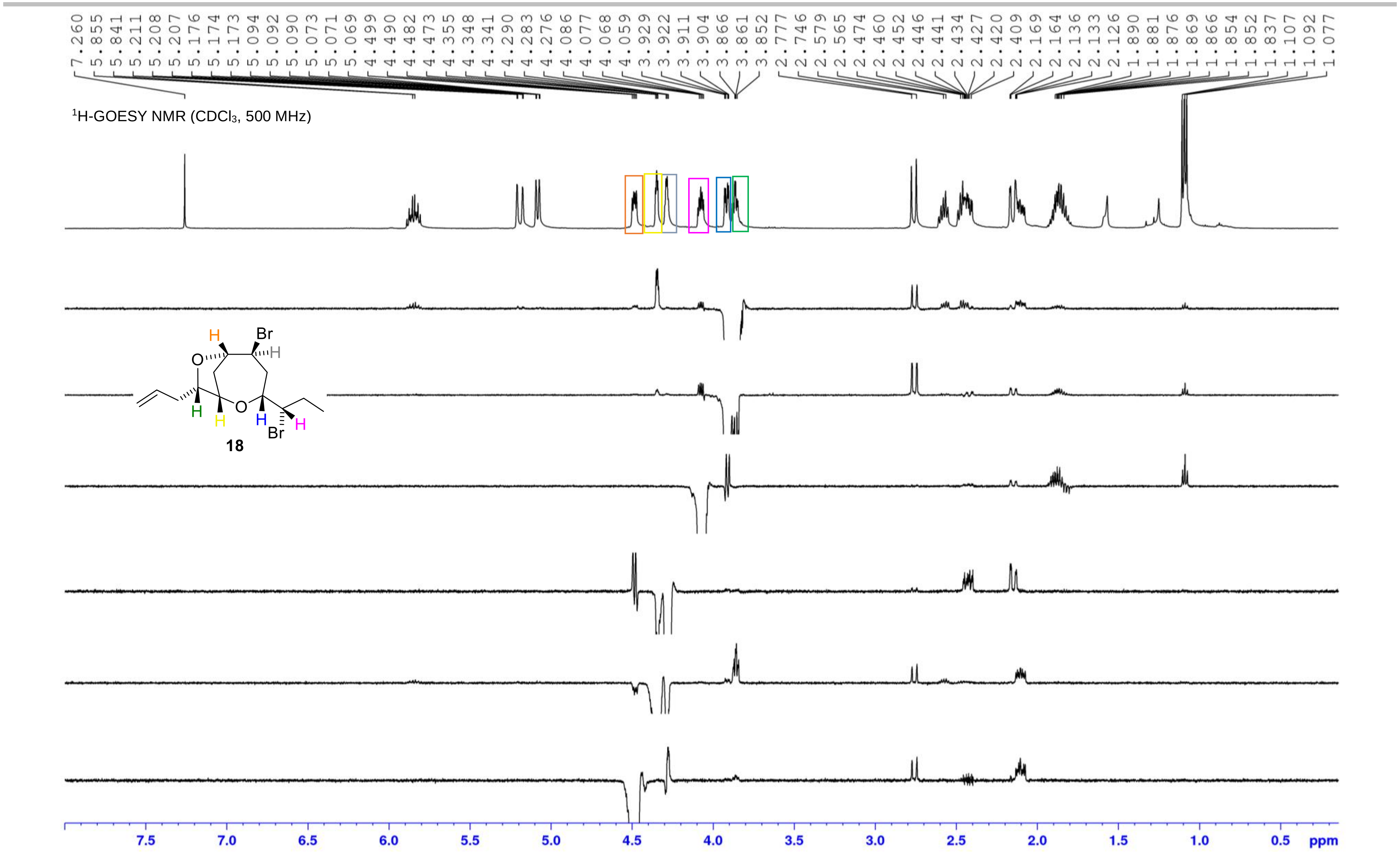
7.260 5.855 5.841 5.211 5.208 5.207 5.176 5.174 5.173 5.094 5.092 5.090 5.073 5.071 5.069 4.499 4.490 4.482 4.473 4.355 4.348 4.341 4.290 4.283 4.276 4.086 4.077 4.068 4.059 3.929 3.922 3.911 3.904 3.866 3.861 3.852 2.777 2.746 2.579 2.565 2.474 2.460 2.452 2.446 2.441 2.434 2.427 2.420 2.409 2.169 2.164 2.136 2.133 2.126 1.890 1.881 1.876 1.869 1.866 1.854 1.852 1.837 1.107 1.092 1.077
1H-GOESY NMR (CDCl3, 500 MHz)
H
Br
H
O
H
H
O
H
Br
H
18
7.5 7.0 6.5 6.0 5.5 5.0 4.5 4.0 3.5 3.0 2.5 2.0 1.5 1.0 0.5 ppm

^{1}H-NMR (CDCl$_3$, 400 MHz)

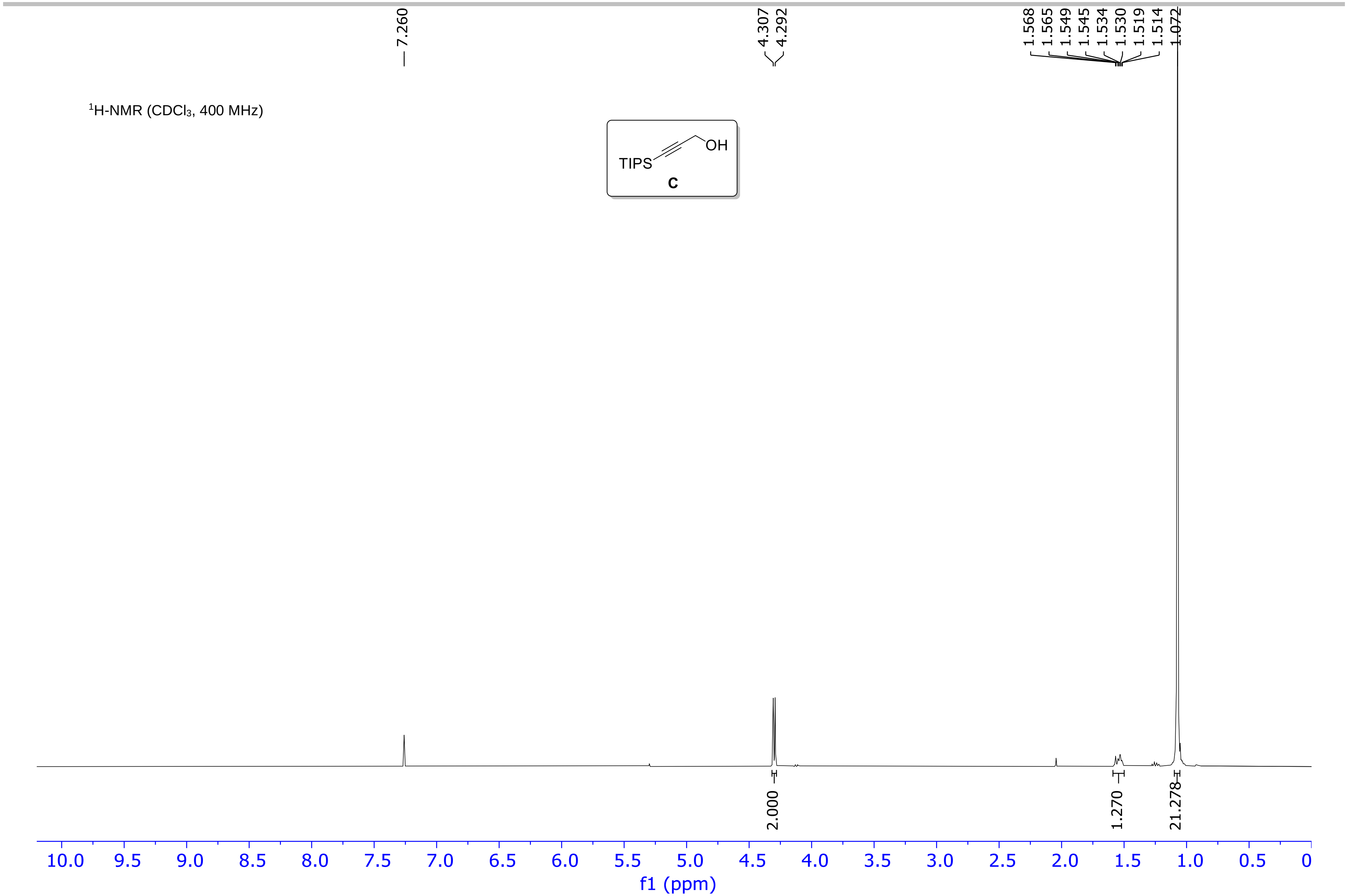

^{13}C-NMR ($CDCl_3$, 100 MHz)

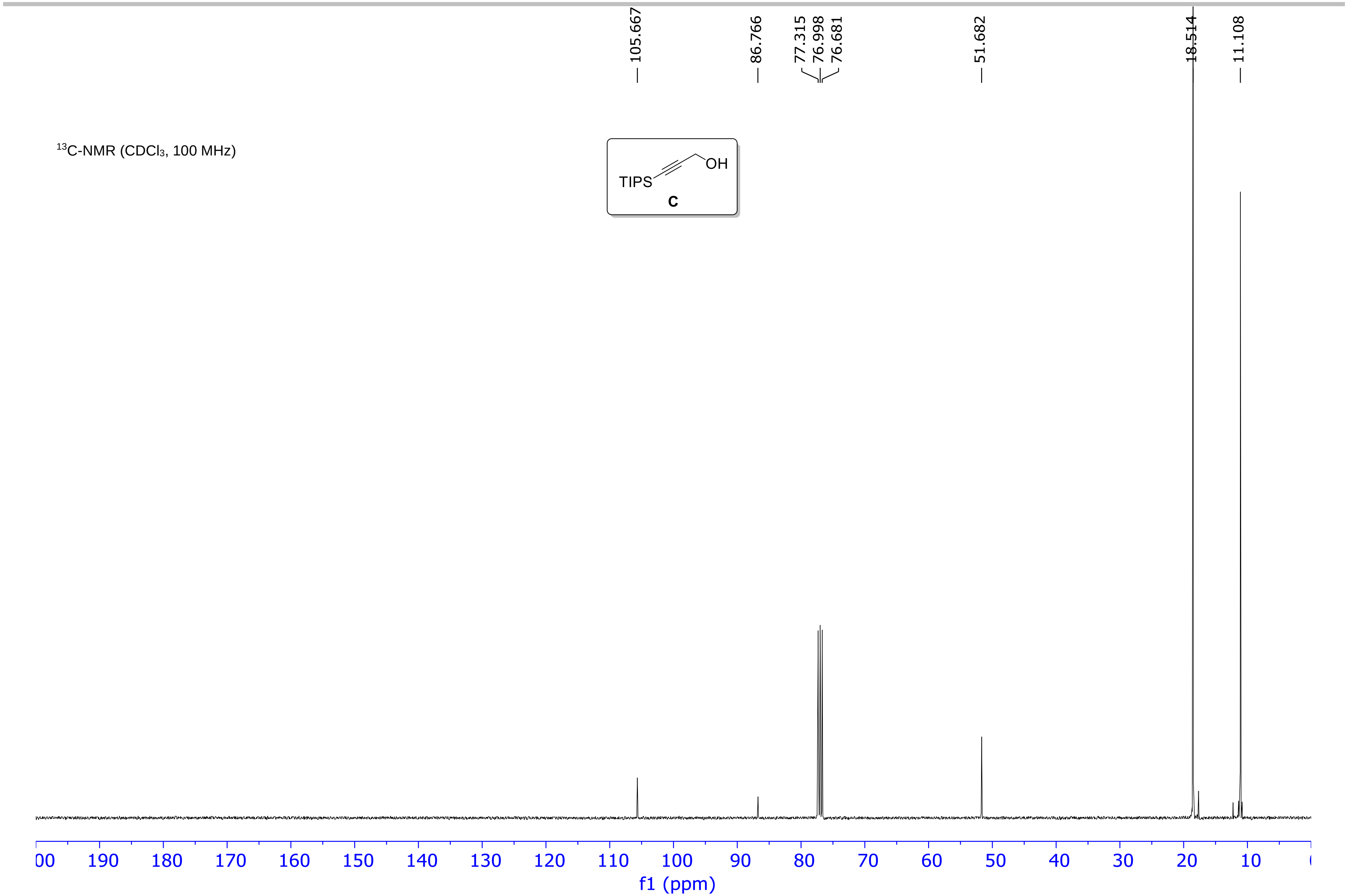

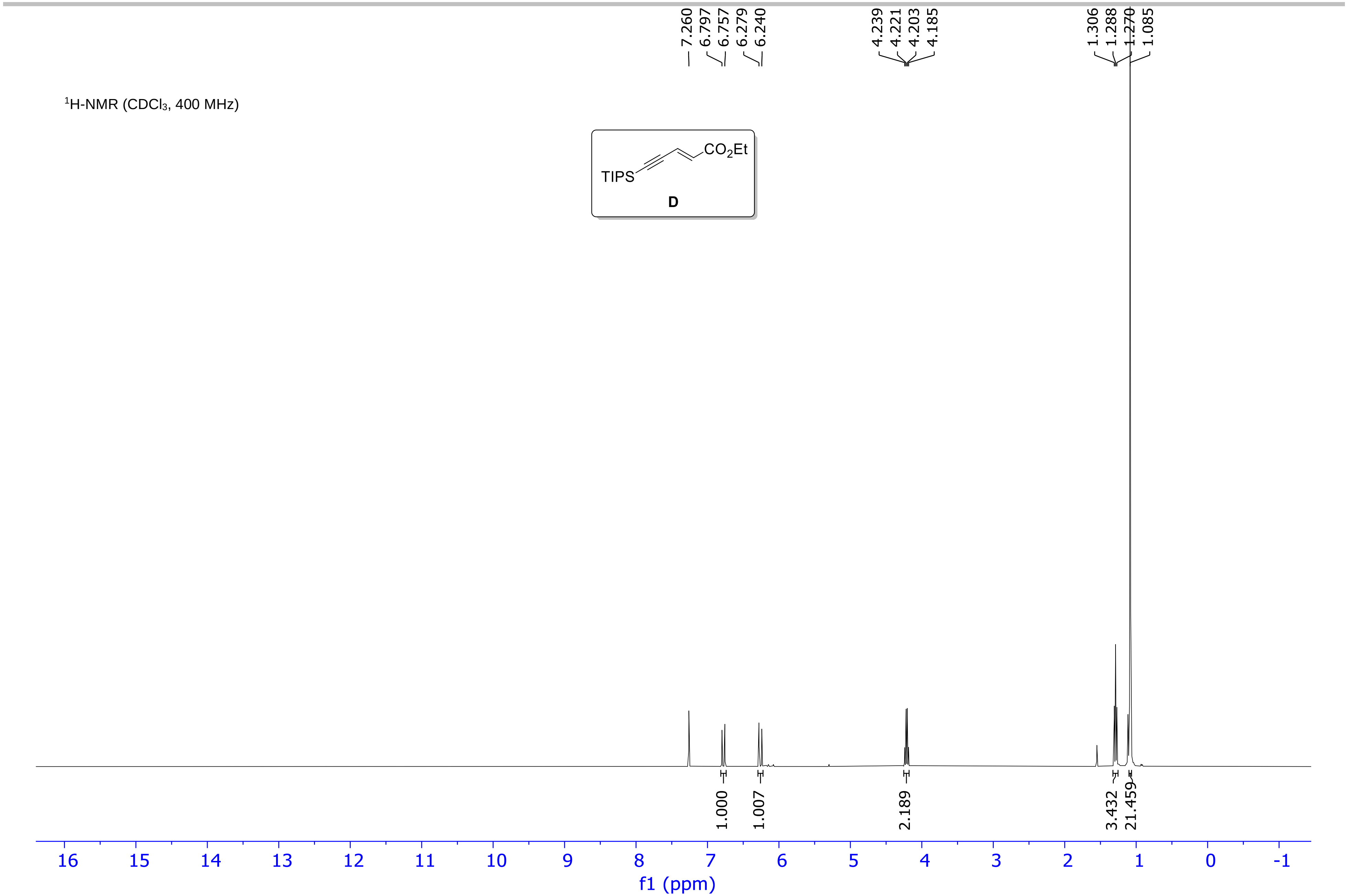

1H-NMR (CDCl3, 400 MHz)
TIPS
CO2Et
D
7.260
6.797
6.757
6.279
6.240
4.239
4.221
4.203
4.185
1.306
1.288
1.270
1.085
1.000
1.007
2.189
3.432
21.459
16 15 14 13 12 11 10 9 8 7 6 5 4 3 2 1 0 -1
f1 (ppm)

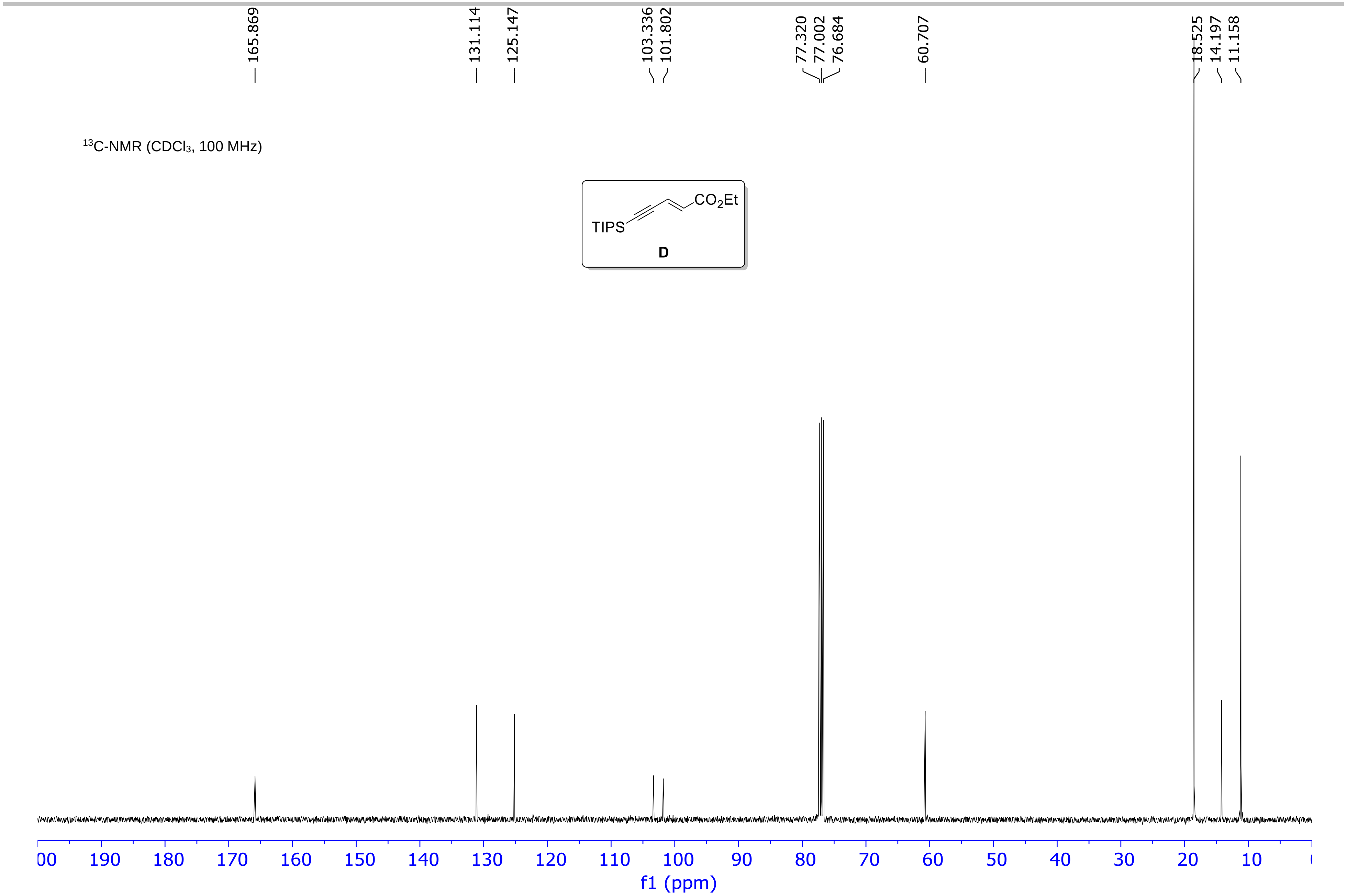
165.869
131.114
125.147
103.336
101.802
77.320
77.002
76.684
60.707
18.525
14.197
11.158
13C-NMR (CDCl3, 100 MHz)
CO2Et
TIPS
D
00
190
180
170
160
150
140
130
120
110
100
90
80
70
60
50
40
30
20
10
f1 (ppm)

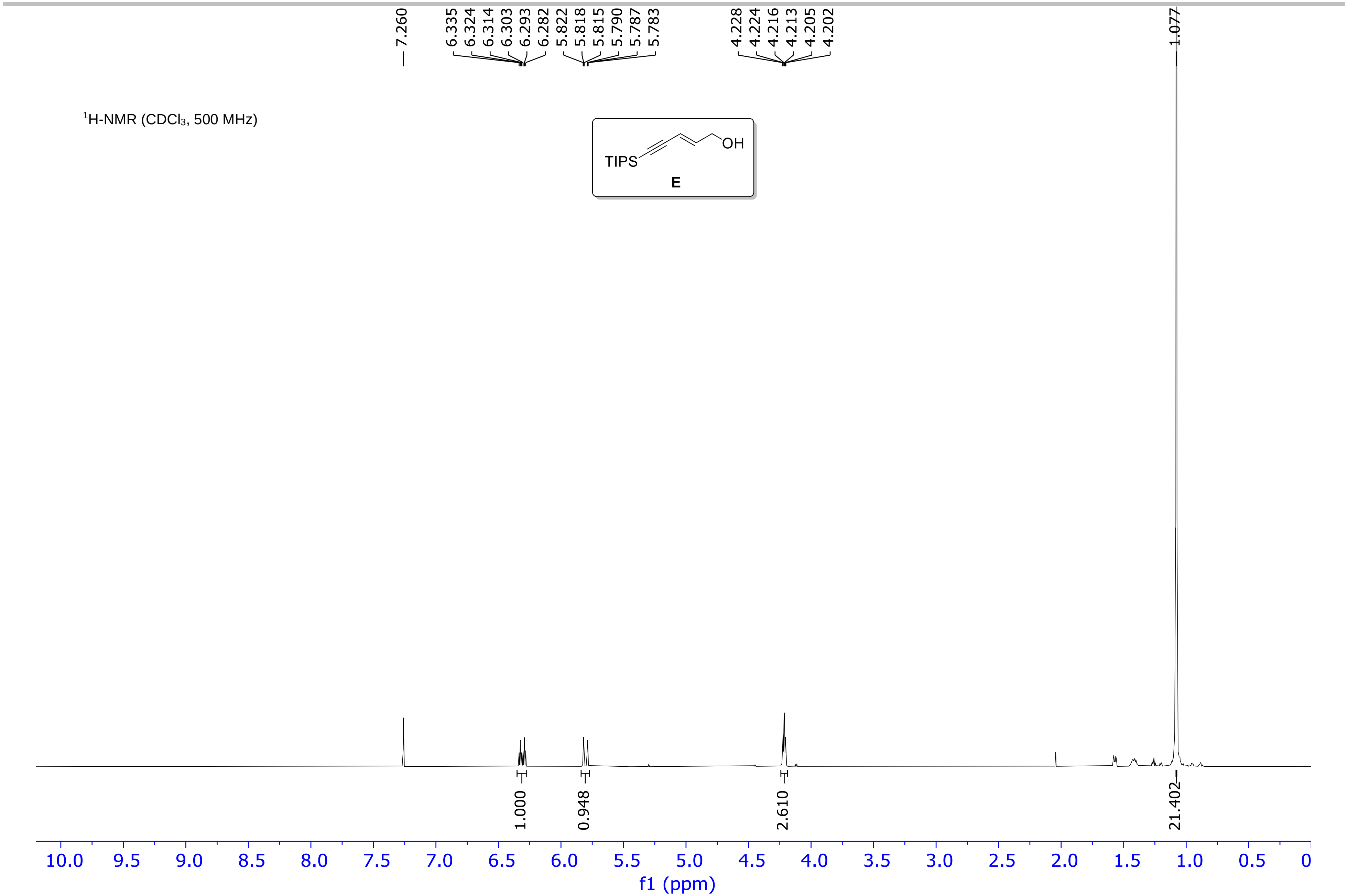

7.260
6.335
6.324
6.314
6.303
6.293
6.282
5.822
5.818
5.815
5.790
5.787
5.783
4.228
4.224
4.216
4.213
4.205
4.202
1.077
^{1}H-NMR ($CDCl_3$, 500 MHz)
TIPS
OH
E
1.000
0.948
2.610
21.402
10.0
9.5
9.0
8.5
8.0
7.5
7.0
6.5
6.0
5.5
5.0
4.5
4.0
3.5
3.0
2.5
2.0
1.5
1.0
0.5
0
f1 (ppm)

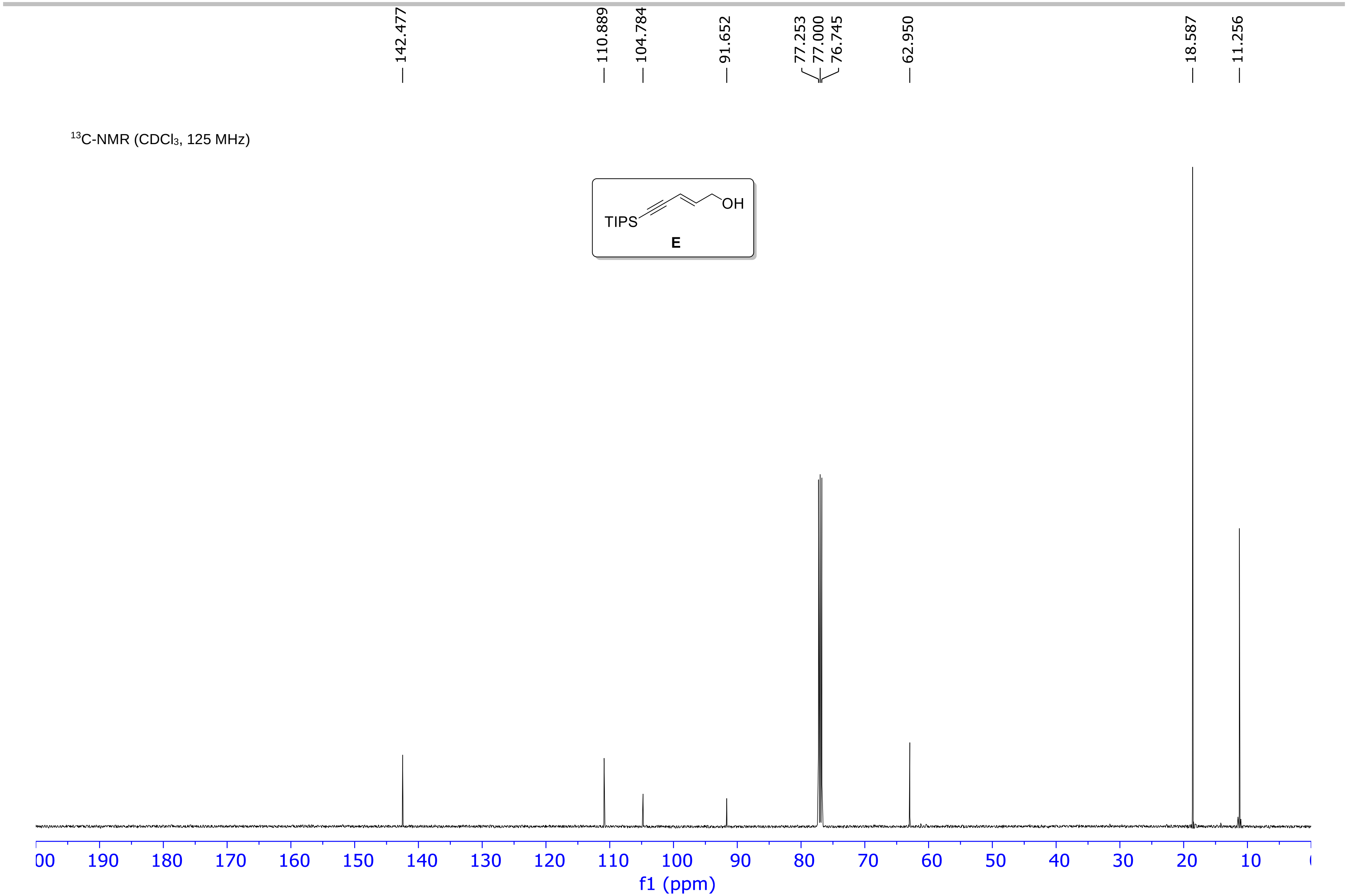
142.477
110.889
104.784
91.652
77.253
77.000
76.745
62.950
18.587
11.256
^{13}C-NMR (CDCl$_3$, 125 MHz)
TIPS
OH
E
00 190 180 170 160 150 140 130 120 110 100 90 80 70 60 50 40 30 20 10
f1 (ppm)

^{1}H-NMR (CDCl$_3$, 500 MHz)

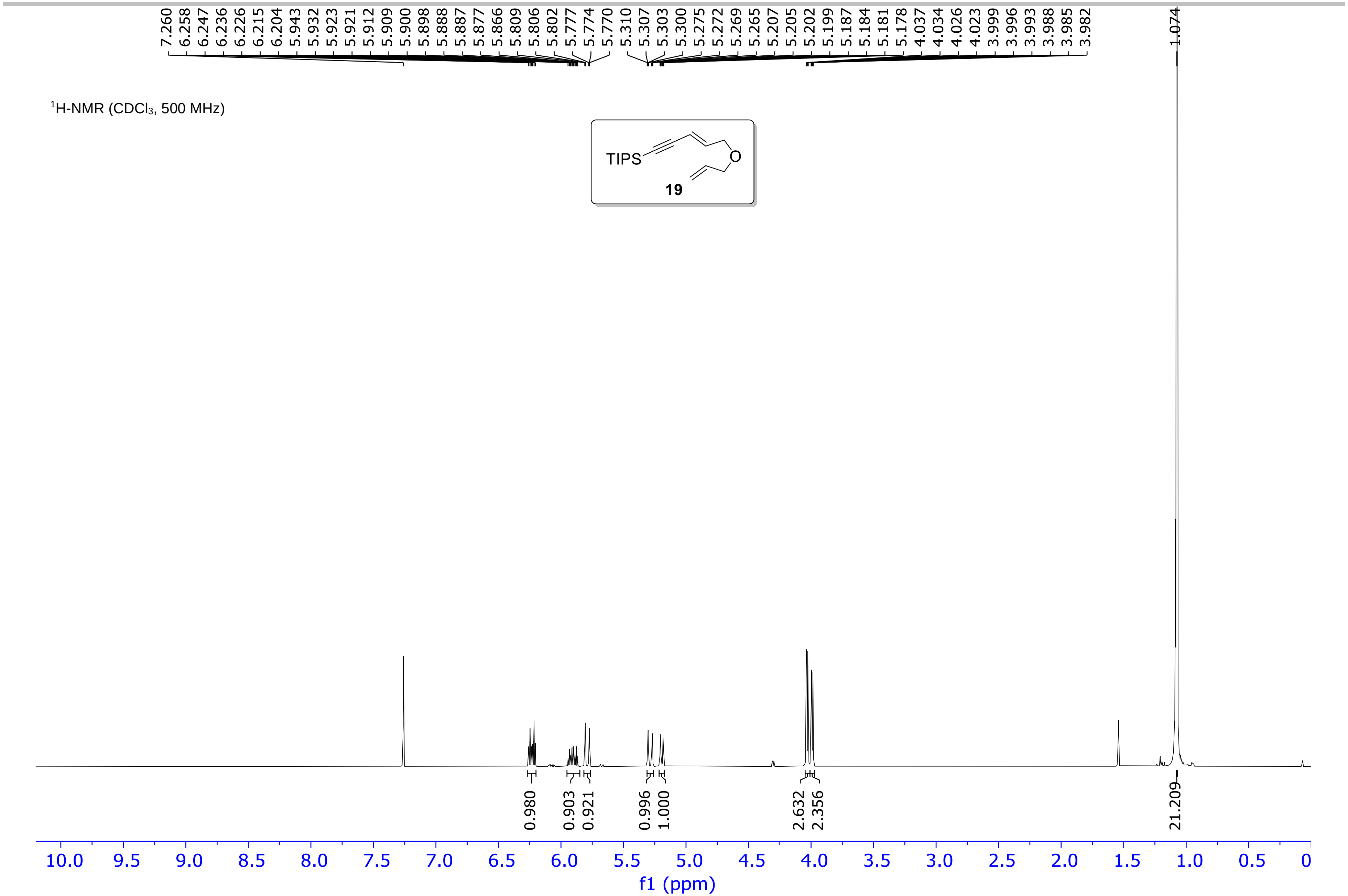

^{13}C-NMR (CDCl$_3$, 125 MHz)

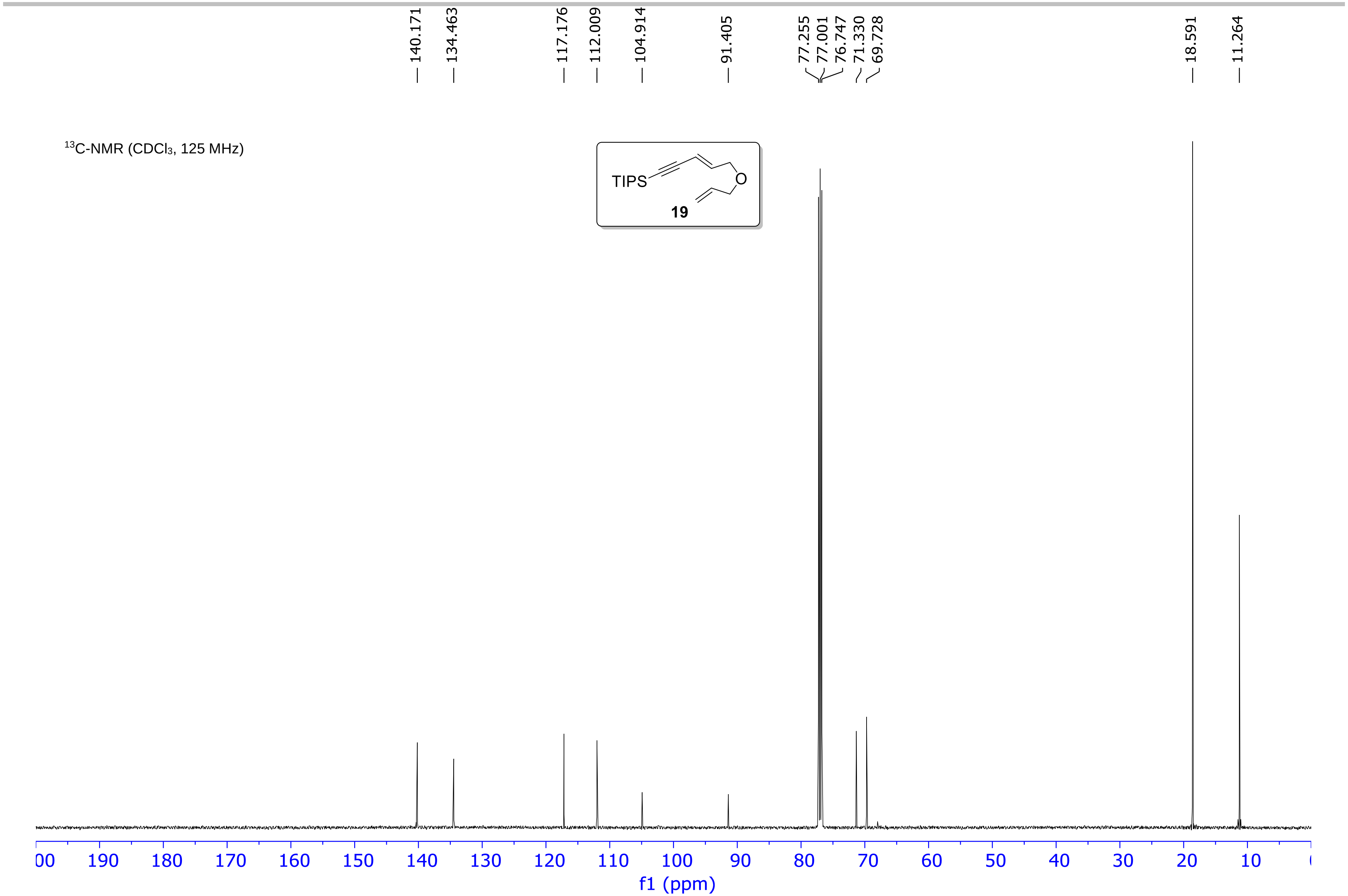

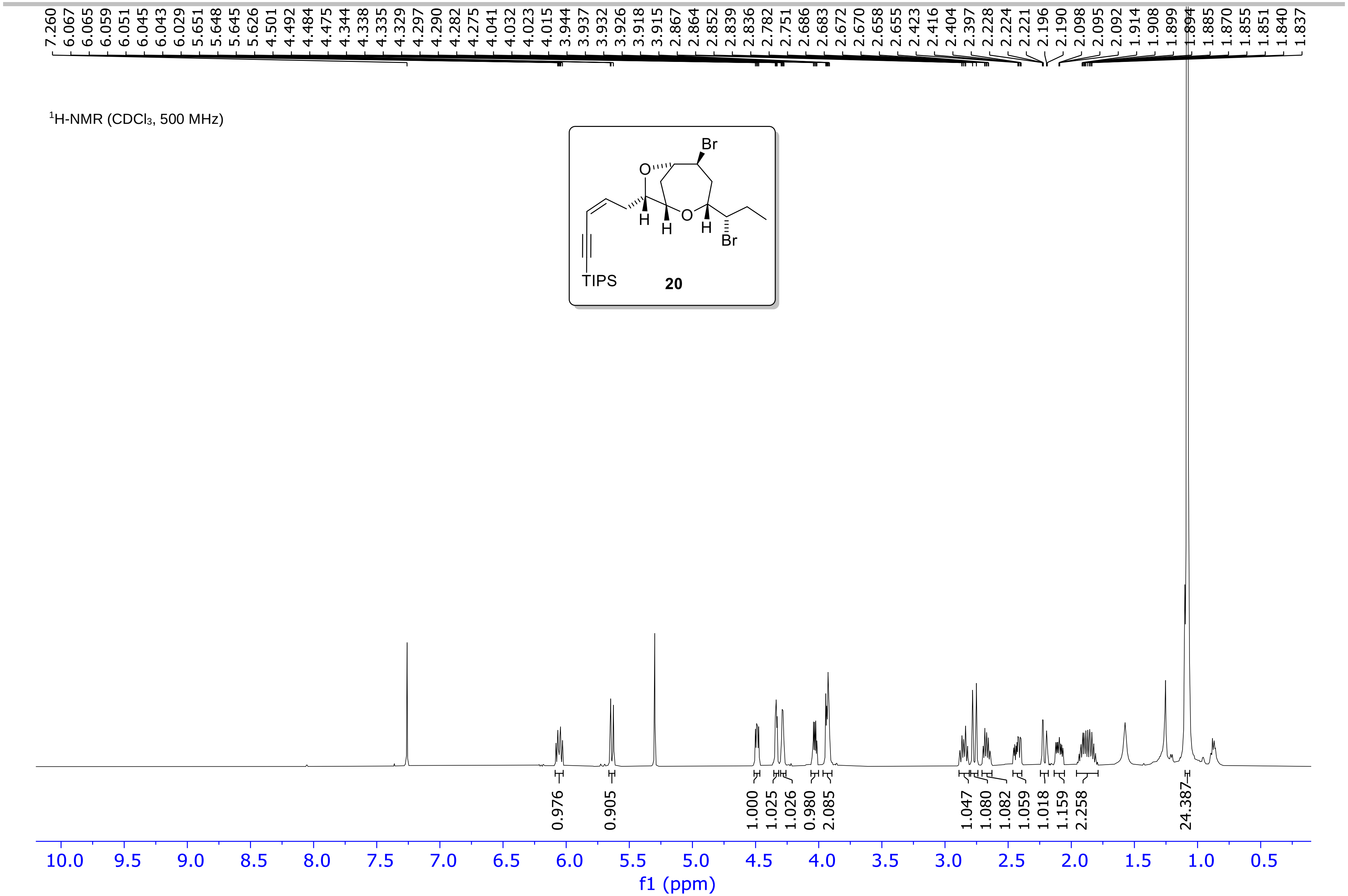
7.260 6.067 6.065 6.059 6.051 6.045 6.043 6.029 5.651 5.648 5.645 5.626 4.501 4.492 4.484 4.475 4.344 4.338 4.335 4.329 4.297 4.290 4.282 4.275 4.041 4.032 4.023 4.015 3.944 3.937 3.932 3.926 3.918 3.915 2.867 2.864 2.852 2.839 2.836 2.782 2.751 2.686 2.683 2.672 2.670 2.658 2.655 2.423 2.416 2.404 2.397 2.228 2.224 2.221 2.196 2.190 2.098 2.095 2.092 1.914 1.908 1.899 1.894 1.885 1.870 1.855 1.851 1.840 1.837
1H-NMR (CDCl3, 500 MHz)
Br
O
H
H
O
H
Br
TIPS
20
0.976 0.905 1.000 1.025 1.026 0.980 2.085 1.047 1.080 1.082 1.059 1.018 1.159 2.258 24.387
10.0 9.5 9.0 8.5 8.0 7.5 7.0 6.5 6.0 5.5 5.0 4.5 4.0 3.5 3.0 2.5 2.0 1.5 1.0 0.5
f1 (ppm)

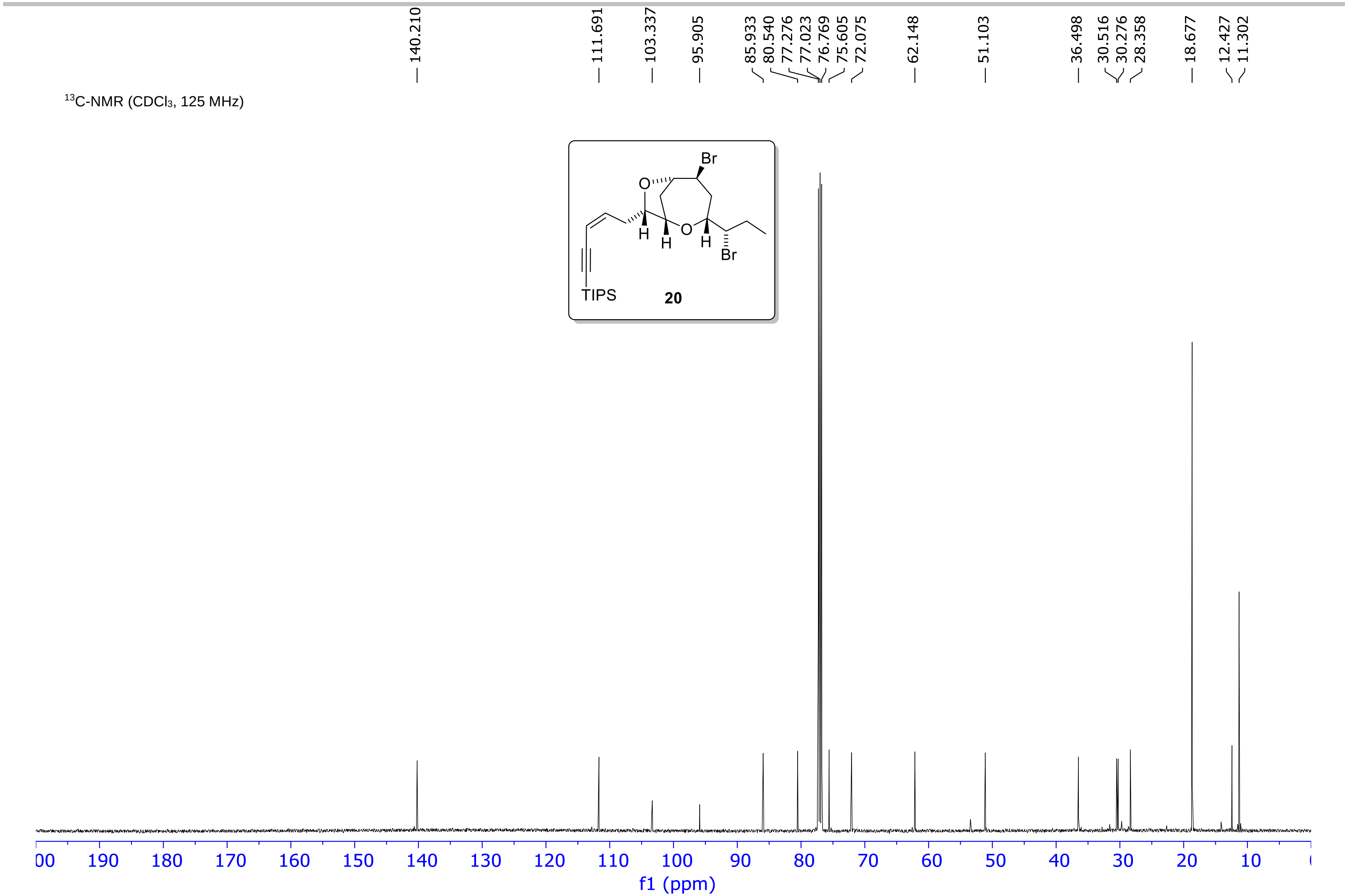
13C-NMR (CDCl3, 125 MHz)
140.210
111.691
103.337
95.905
85.933
80.540
77.276
77.023
76.769
75.605
72.075
62.148
51.103
36.498
30.516
30.276
28.358
18.677
12.427
11.302
Br
O
H
H
O
H
Br
TIPS
20
00
190
180
170
160
150
140
130
120
110
100
90
80
70
60
50
40
30
20
10
f1 (ppm)

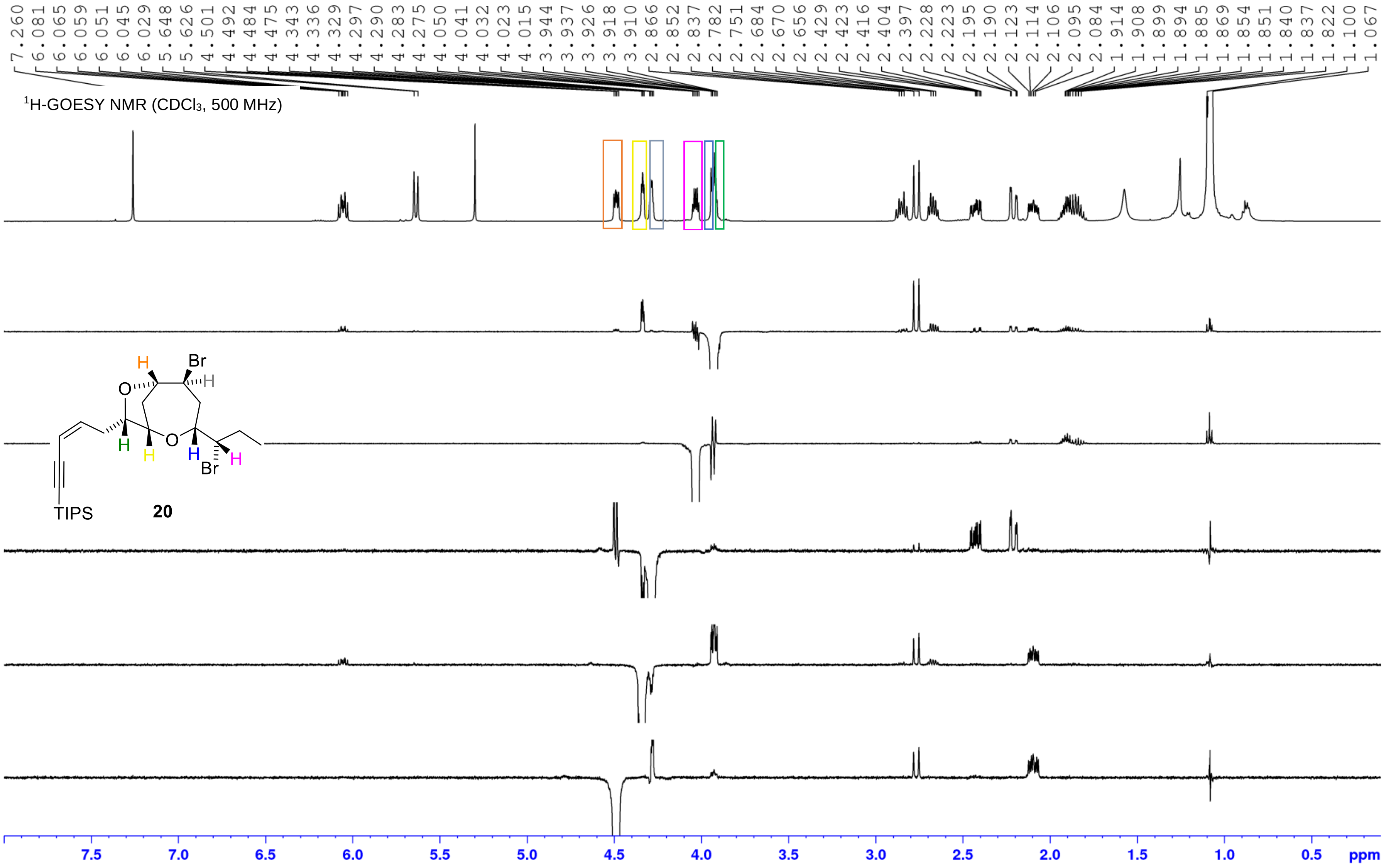

^{1}H-GOESY NMR (CDCl$_3$, 500 MHz)
7.260 6.081 6.065 6.059 6.051 6.045 6.029 5.648 5.626 4.501 4.492 4.484 4.475 4.343 4.336 4.329 4.297 4.290 4.283 4.275 4.050 4.041 4.032 4.023 4.015 3.944 3.937 3.926 3.918 3.910 2.866 2.852 2.837 2.782 2.751 2.684 2.670 2.656 2.429 2.423 2.416 2.404 2.397 2.228 2.223 2.195 2.190 2.123 2.114 2.106 2.095 2.084 1.914 1.908 1.899 1.894 1.885 1.869 1.854 1.851 1.840 1.837 1.822 1.100 1.067
Br
H
O
H
H
O
H
Br
H
H
TIPS
20
7.5
7.0
6.5
6.0
5.5
5.0
4.5
4.0
3.5
3.0
2.5
2.0
1.5
1.0
0.5
ppm

^{1}H-NMR (CDCl$_3$, 500 MHz)

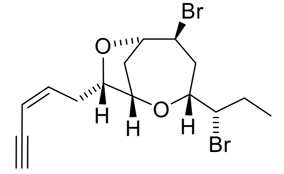

(+)-neoisoprelaurefucin (**2**)

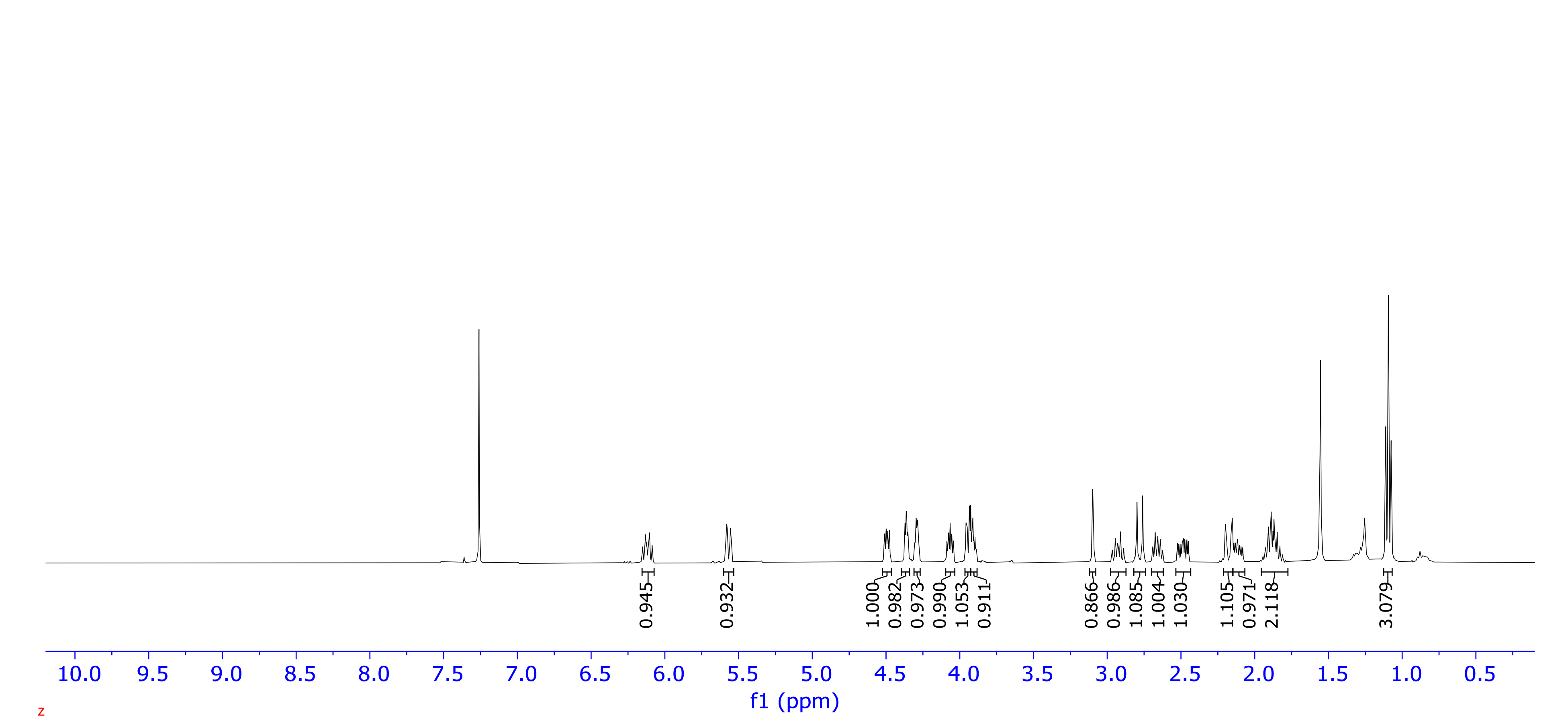

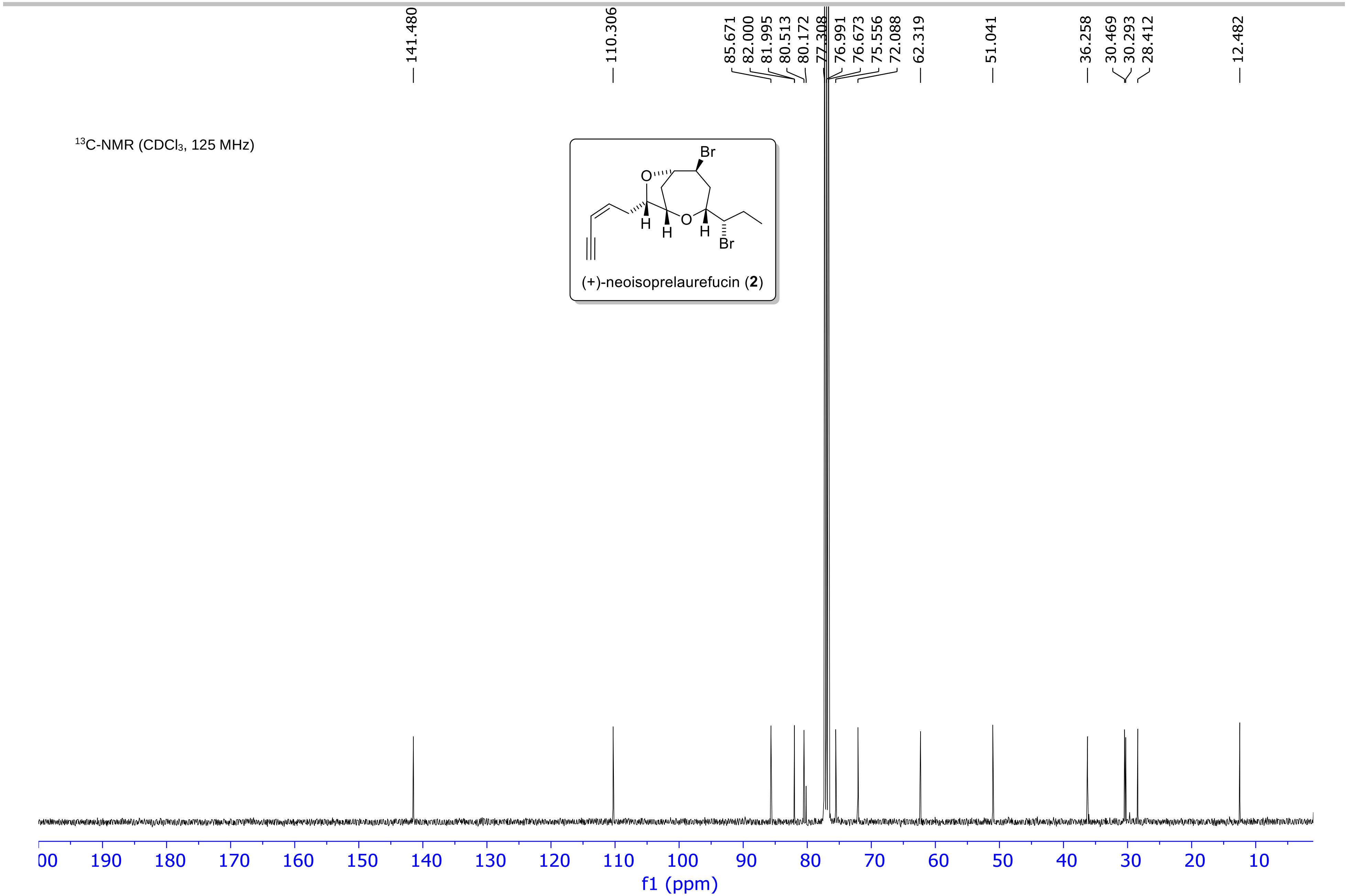

13C-NMR (CDCl3, 125 MHz)
141.480
110.306
85.671
82.000
81.995
80.513
80.172
77.308
76.991
76.673
75.556
72.088
62.319
51.041
36.258
30.469
30.293
28.412
12.482
Br
O
H
H
O
H
Br
(+)-neoisoprelaurefucin (2)
00
190
180
170
160
150
140
130
120
110
100
90
80
70
60
50
40
30
20
10
f1 (ppm)

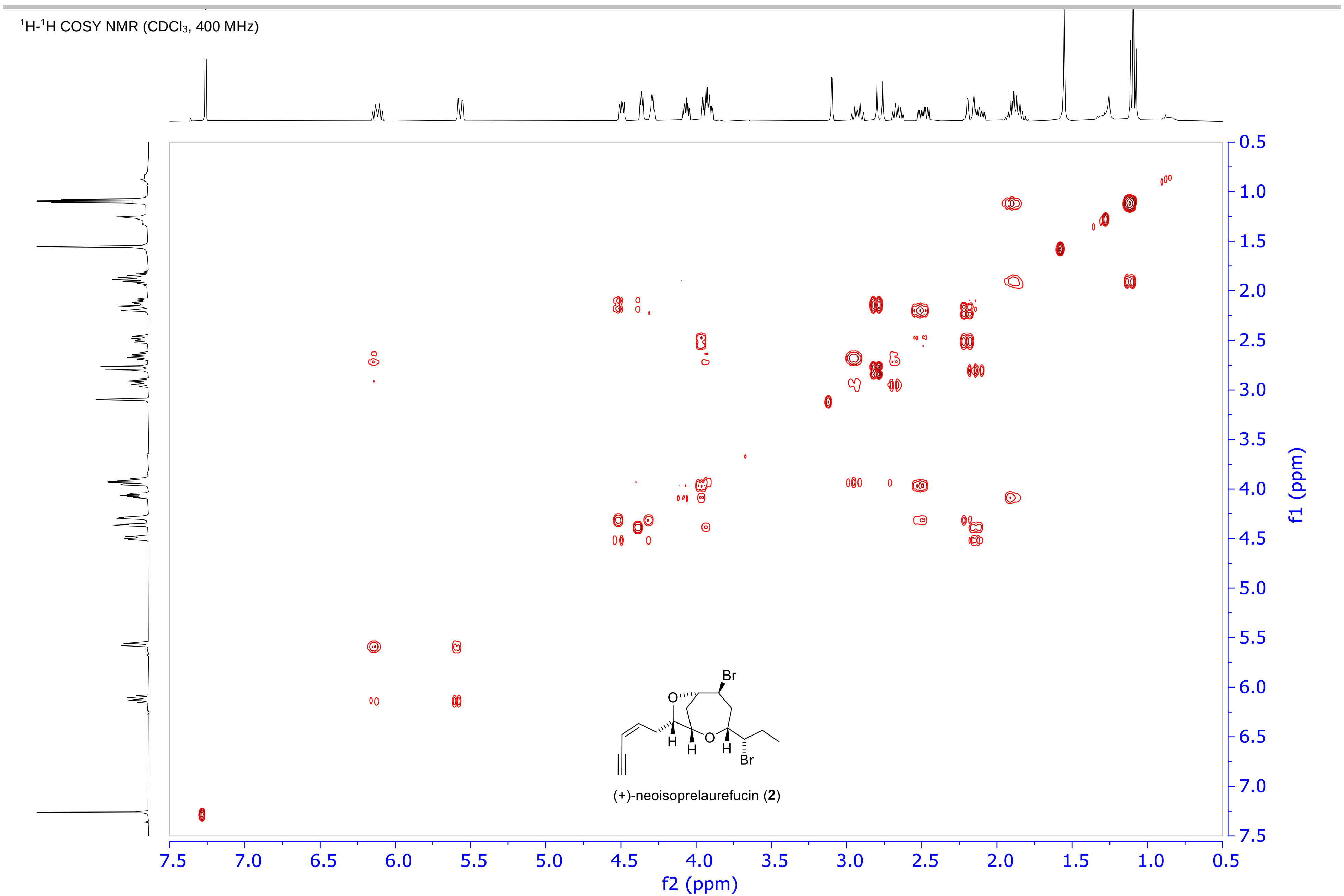

1H-1H COSY NMR (CDCl3, 400 MHz)
f2 (ppm)
f1 (ppm)
(+)-neoisoprelaurefucin (2)

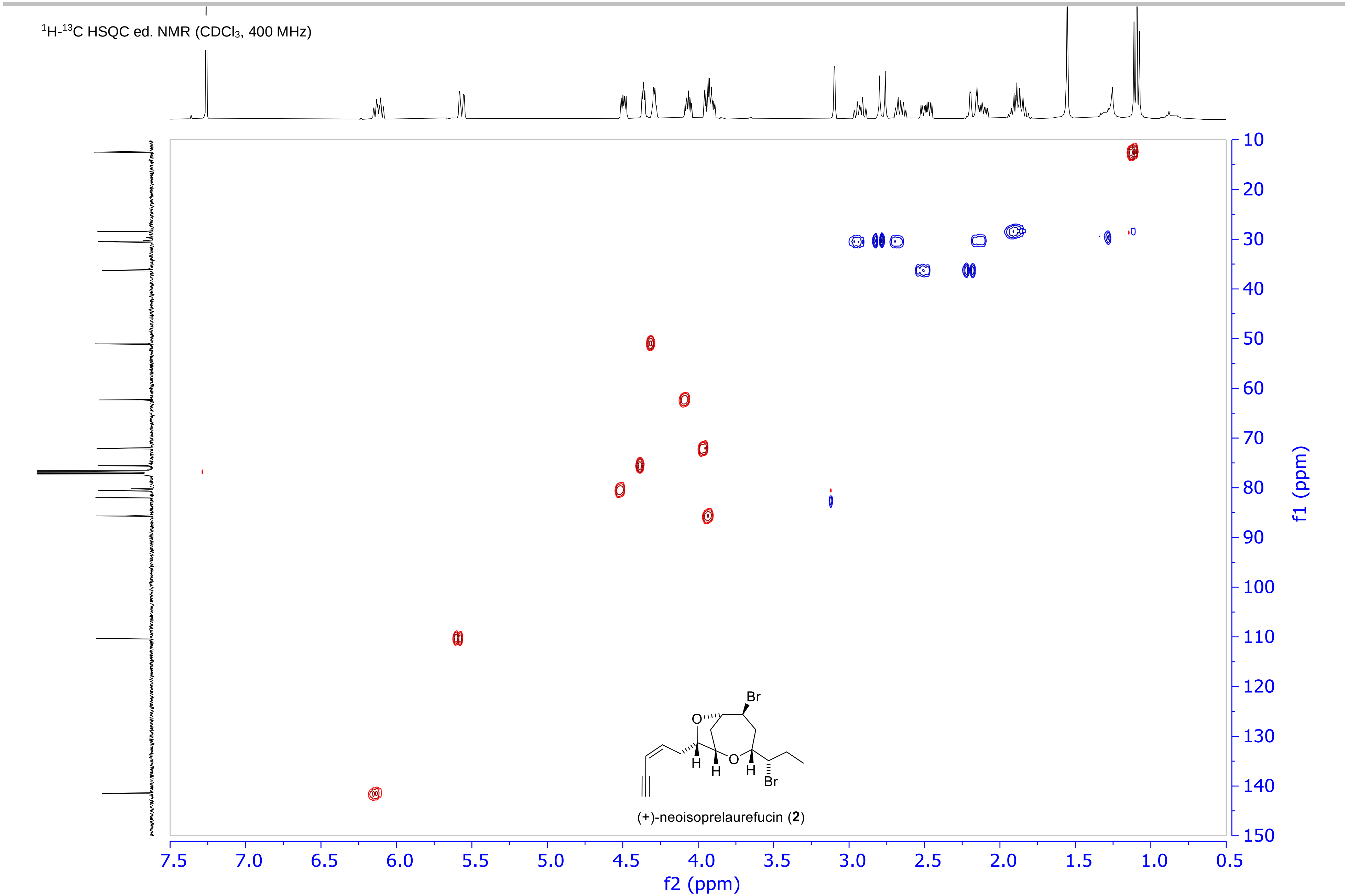
^{1}H-^{13}C HSQC ed. NMR (CDCl$_3$, 400 MHz)
f1 (ppm)
f2 (ppm)
Br
O
H
H
O
H
Br
(+)-neoisoprelaurefucin (**2**)

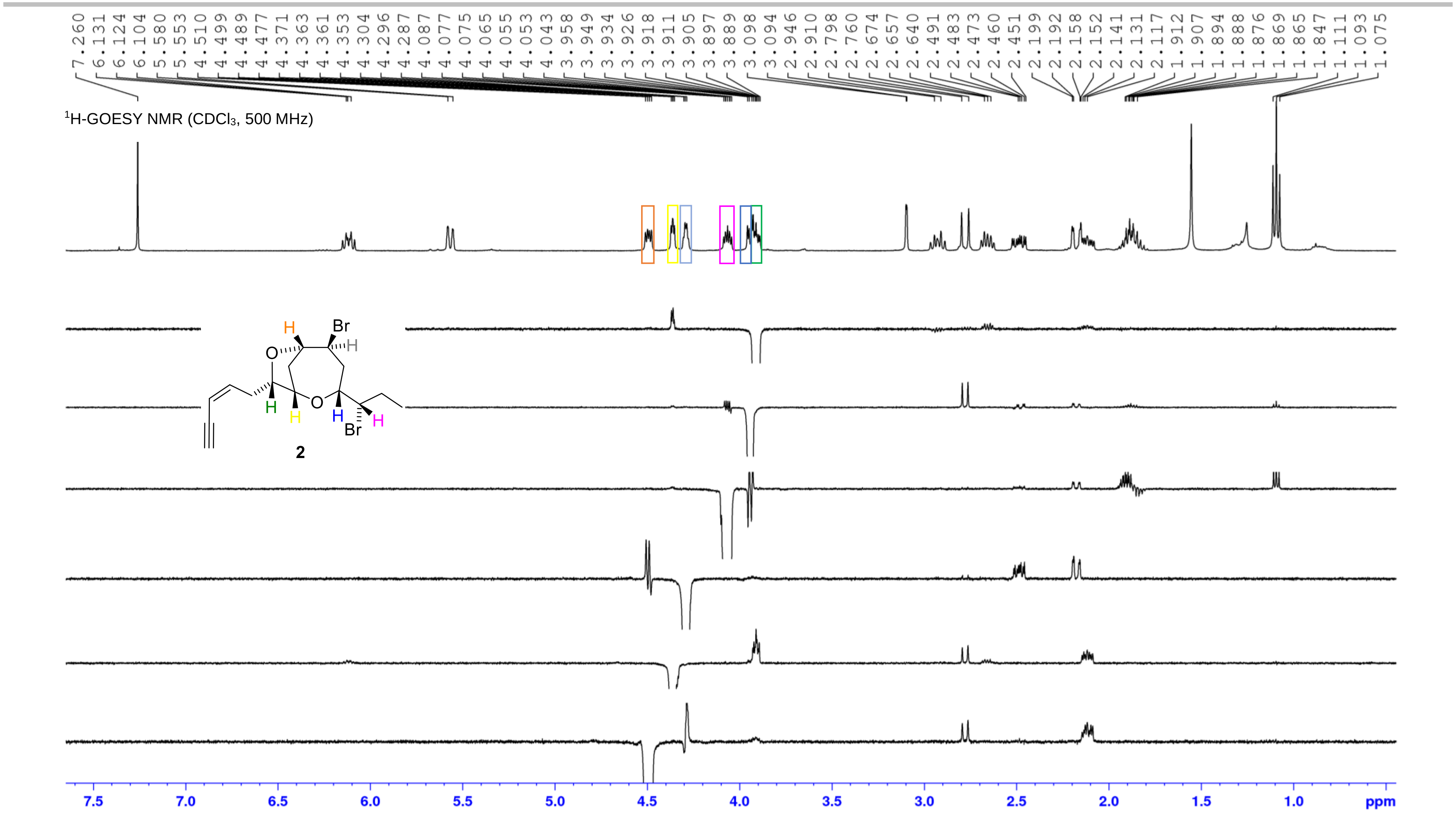
7.260 6.131 6.124 6.104 5.580 5.553 4.510 4.499 4.489 4.477 4.371 4.363 4.361 4.353 4.304 4.296 4.287 4.087 4.077 4.075 4.065 4.055 4.053 4.043 3.958 3.949 3.934 3.926 3.918 3.911 3.905 3.897 3.889 3.098 3.094 2.946 2.910 2.798 2.760 2.674 2.657 2.640 2.491 2.483 2.473 2.460 2.451 2.199 2.192 2.158 2.152 2.141 2.131 2.117 1.912 1.907 1.894 1.888 1.876 1.869 1.865 1.847 1.111 1.093 1.075
1H-GOESY NMR (CDCl3, 500 MHz)
H
Br
H
O
O
H
H
H
Br
H
2
7.5 7.0 6.5 6.0 5.5 5.0 4.5 4.0 3.5 3.0 2.5 2.0 1.5 1.0 ppm
